# Herpetological phylogeographic analyses support a Miocene focal point of Himalayan uplift and biological diversification

**DOI:** 10.1093/nsr/nwaa263

**Published:** 2020-10-21

**Authors:** Wei Xu, Wen-Jie Dong, Ting-Ting Fu, Wei Gao, Chen-Qi Lu, Fang Yan, Yun-He Wu, Ke Jiang, Jie-Qiong Jin, Hong-Man Chen, Ya-Ping Zhang, David M Hillis, Jing Che

**Affiliations:** State Key Laboratory of Genetic Resources and Evolution, Kunming Institute of Zoology, Chinese Academy of Sciences, Kunming 650223, China; Kunming College of Life Science, University of Chinese Academy of Sciences, Kunming 650204, China; State Key Laboratory of Genetic Resources and Evolution, Kunming Institute of Zoology, Chinese Academy of Sciences, Kunming 650223, China; Kunming College of Life Science, University of Chinese Academy of Sciences, Kunming 650204, China; State Key Laboratory of Genetic Resources and Evolution, Kunming Institute of Zoology, Chinese Academy of Sciences, Kunming 650223, China; Kunming College of Life Science, University of Chinese Academy of Sciences, Kunming 650204, China; State Key Laboratory of Genetic Resources and Evolution, Kunming Institute of Zoology, Chinese Academy of Sciences, Kunming 650223, China; Kunming College of Life Science, University of Chinese Academy of Sciences, Kunming 650204, China; State Key Laboratory of Genetic Resources and Evolution, Kunming Institute of Zoology, Chinese Academy of Sciences, Kunming 650223, China; Kunming College of Life Science, University of Chinese Academy of Sciences, Kunming 650204, China; State Key Laboratory of Genetic Resources and Evolution, Kunming Institute of Zoology, Chinese Academy of Sciences, Kunming 650223, China; State Key Laboratory of Genetic Resources and Evolution, Kunming Institute of Zoology, Chinese Academy of Sciences, Kunming 650223, China; Kunming College of Life Science, University of Chinese Academy of Sciences, Kunming 650204, China; State Key Laboratory of Genetic Resources and Evolution, Kunming Institute of Zoology, Chinese Academy of Sciences, Kunming 650223, China; State Key Laboratory of Genetic Resources and Evolution, Kunming Institute of Zoology, Chinese Academy of Sciences, Kunming 650223, China; State Key Laboratory of Genetic Resources and Evolution, Kunming Institute of Zoology, Chinese Academy of Sciences, Kunming 650223, China; State Key Laboratory of Genetic Resources and Evolution, Kunming Institute of Zoology, Chinese Academy of Sciences, Kunming 650223, China; Center for Excellence in Animal Evolution and Genetics, Chinese Academy of Sciences, Kunming 650223, China; Department of Integrative Biology and Biodiversity Center, University of Texas at Austin, Austin, TX 78712, USA; State Key Laboratory of Genetic Resources and Evolution, Kunming Institute of Zoology, Chinese Academy of Sciences, Kunming 650223, China; Center for Excellence in Animal Evolution and Genetics, Chinese Academy of Sciences, Kunming 650223, China

**Keywords:** orogenesis, monsoon system, biotic assembly, *in situ* diversification, amphibians and reptiles

## Abstract

The Himalaya are among the youngest and highest mountains in the world, but the exact timing of their uplift and origins of their biodiversity are still in debate. The Himalayan region is a relatively small area but with exceptional diversity and endemism. One common hypothesis to explain the rich montane diversity is uplift-driven diversification—that orogeny creates conditions favoring rapid *in situ* speciation of resident lineages. We test this hypothesis in the Himalayan region using amphibians and reptiles, two environmentally sensitive vertebrate groups. In addition, analysis of diversification of the herpetofauna provides an independent source of information to test competing geological hypotheses of Himalayan orogenesis. We conclude that the origins of the Himalayan herpetofauna date to the early Paleocene, but that diversification of most groups was concentrated in the Miocene. There was an increase in both rates and modes of diversification during the early to middle Miocene, together with regional interchange (dispersal) between the Himalaya and adjacent regions. Our analyses support a recently proposed stepwise geological model of Himalayan uplift beginning in the Paleocene, with a subsequent rapid increase of uplifting during the Miocene, finally giving rise to the intensification of the modern South Asian Monsoon.

## INTRODUCTION

In the past 40 million years, there has been a sharp increase in global tectonic activity and associated orogeny [1]. These geological processes, in turn, have resulted in many climatic and environmental changes, which have strongly influenced regional biological diversification [2]. Among the global biodiversity hotspots, mountains surrounding the Qinghai–Tibetan Plateau are enigmatic and unusual [3], especially the Himalaya—the highest mountains in the world.

**Figure 1.**
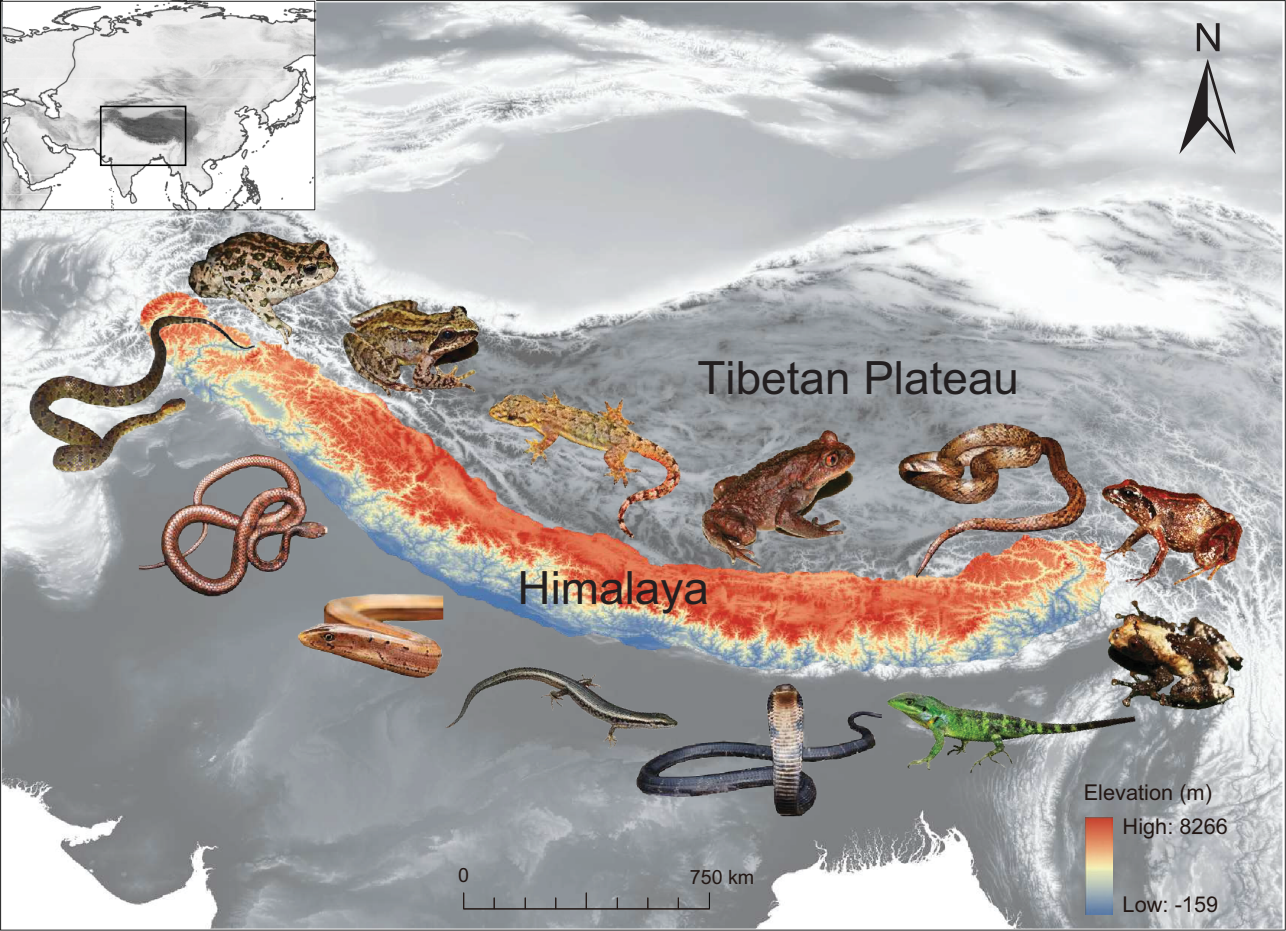
Map of the Himalaya geographic regions (highlighted by colors) used for analyses. The color scale on the bottom right indicates the elevations of the Himalaya. Representative species of the Himalaya are shown along the mountain range (from the upper left corner clockwise is *Bufotes zamdaensis*-Bufonidae, *Nanorana rostandi*-Dicroglossidae, *Hemidactylus platyurus*-Gekkonidae, *Scutiger wuguanfui*-Megophryidae, *Oligodon lipipengi*-Colubridae, *Liurana medogensis*-Ceratobatrachidae, *Theloderma baibungense*-Rhacophoridae, *Mictopholis austeniana*-Agamidae, *Ophiophagus hannah*-Elapidae, *Asymblepharus himalayanus*-Scincidae, *Dopasia gracilis*-Anguidae, *Pareas monticola*-Pareidae, *Protobothrops himalayanus*-Viperidae).

Geologically, the Himalaya are distinct from the Tibetan Plateau and the Hengduan Mountains (Fig. 1; definition of the Himalaya in the Supplementary Data), although all of these regions have often been considered as a greater and united Qinghai–Tibetan Plateau. The uplift of the Himalaya is suggested to be more important than the Tibetan Plateau in shaping Asian paleoclimate patterns [4]. This massive mountain range encompasses remarkable endemic diversity [5,6]. However, despite increasing interest, the overall biotic assembly remains poorly understood. In this study, we aim to better understand the origin of this remarkable biotic assembly in the Himalayan region. Time-calibrated analyses of patterns of diversification of biotic assembly can provide independent estimates or corroboration of the timing of geological processes such as mountain range formation and related climate changes.

**Figure 2.**
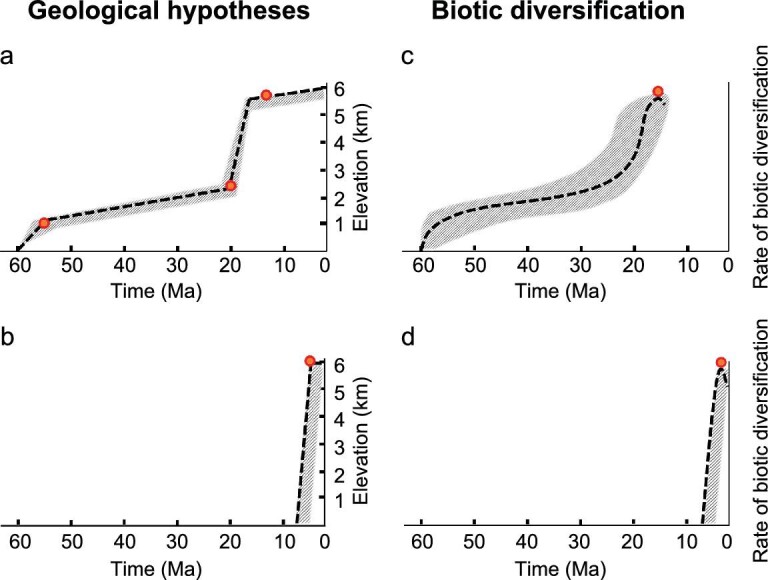
Schematic diagram of (a, b) two geological hypotheses regarding the uplift of the Himalaya to current elevations, and (c, d) the corresponding expected biotic assembly processes. The dashed lines show the general trends, with the range of consistent dates and elevations indicated by shading. The red solid circles indicate key time points. (a) The ‘Stepwise hypothesis’ refers to a model in which the Himalaya arose in a stepwise series of uplifts beginning in the Early Paleocene, but at a much faster rate during the Miocene (23–15 Ma). This diagram is adapted from Ding *et al.* [8]. (b) The ‘Late Orogeny hypothesis’ refers to a model in which the Himalaya started uplift during the Early Miocene and reached the current elevations much later (around 7.0–3.2 Ma). This diagram is drawn based on a synthesis of several studies [9,11]. (c) If the ‘Stepwise hypothesis’ is correct, the expected biotic diversification would begin in the Paleocene, but then exhibit a rapid increase during the early Miocene and hit the peak in the middle Miocene (ca. 15 Ma). (d) If the ‘Late Orogeny hypothesis’ is correct, the expected diversification of Himalayan biota would occur recently (after the Miocene), with a peak of diversification later than 7.0–3.2 Ma.

Geological studies about the Himalaya formation have made great progress recently, however, understanding the timing of the subsequent rise to current elevations has proved challenging and controversial [7]. Hypotheses about the geological history of the Himalaya largely differ in the timing and sequence of the uplift process (Fig. 2a and b). Ding *et al.* [8] proposed a stepwise model, which suggested that the Himalaya rose slowly from 1000–2500 m during the period of 56–23 Ma, with an additional rapid period of elevational increase to 4000 m from 23–19 Ma, and a final rise around 15 Ma to the current average elevations of about 5000 m. We label this model the ‘Stepwise hypothesis’ (Fig. 2a). Coincident with the rise of the highest elevations, the modern South Asian Monsoon began to intensify [4,8]. However, the discovery of fossil oaks (*Quercus semecarpifolia*) indicated that the Himalaya were the most recent component of the Tibet–Himalaya edifice to be elevated, and reached their current elevations during the Pliocene [9], although this has been questioned [7,10]. Recent hydrological and thermal evidence also supports that this region was probably not elevated to current elevations till the mid-Pliocene [11]. We label this view the ‘Late Orogeny hypothesis’ (Fig. 2b).

Orogenies create variable environmental conditions (such as varying climatic niches, new habitats and dispersal barriers) that increase the rate of speciation of organisms—a process termed uplift-driven diversification [12,13]. We thus expect an accelerated *in situ* diversification rate following the uplift of the Himalaya. Spicer [14] proposed that the rise of the Himalaya and the subsequent development of the South Asian Monsoon had major impacts on species diversification in this region and a recent study on the alpine flora diversification in the Himalaya supported this scenario [15]. We therefore expect a time-based record of biological processes to be informative about montane histories and environmental changes. Various hypotheses about Himalayan origins can be tested using phylogenetic information and estimates of the timing of biological speciation events. In Fig. 2, we show the predictions of biotic diversification that are associated with each of the two models discussed above (geological models: Fig. 2a and b; biotic diversification predictions, Fig. 2c and d). Trends of biotic predictions show the expectations of biological diversification under each model. We use ‘rate of biotic diversification’ in Fig. 2 to represent the net effect of biota assembly processes (e.g. all forms of diversification, minus extinction).

The effects of Himalayan orogeny and the subsequent monsoon system development on the biotic diversification rate are not clearly separable, because they may jointly provide ecological/evolutionary opportunity to accelerate the speciation rate. However, at larger scales, changes in the moisture load and strength of the monsoon systems during its intensification must have affected the availability of water throughout the Qinghai–Tibetan Plateau region, with the growing Himalayan rain shadow especially important [14]. Increased rains kept the south slope of the Himalaya wet and facilitated the establishment of rainforest, with a corridor connected with Southeast Asia [16]. Under the theoretical framework of phylogenetic niche conservatism, dispersal should be facilitated between similar environments inhabited by the source biota [17]. In contrast, by the time the Himalayan uplift reached almost 5000 m around 15 Ma [8], the aridity of central Asia and Tibet had been established [3]. Under this model, we could expect limited biotic interchanges between the Himalaya and either central Tibet or Central-West Asia, because of the reduced effectiveness of dispersal corridors between these areas.

Our understanding of the assembly processes of the Himalayan biota has been hindered by a lack of phylogenetic and diversification dating data. This lack of information results from the relative difficulty of sampling many Himalayan species. The Himalayan region encompasses multiple countries and has many access challenges, so sampling across the entire region is difficult, which has inhibited integrative studies of the origin of the Himalayan biota. Recently, new radiometric dates of paleontological data point to Himalayan high biodiversity originating in the Paleogene [14]. Another long-held view—the sink hypothesis—suggests that the Himalayan biota, due to its high connectivity, is largely comprised of elements from adjacent biotic realms, e.g. Western Asia, Southeast and East Asia [18,19]. Although there are a few existing phylogeographic studies on endemic Himalayan clades [20,21], a broad synthesis is lacking for most major groups, and there is little available information about broad biotic interchanges between the Himalaya and adjacent regions [3].

**Figure 3.**
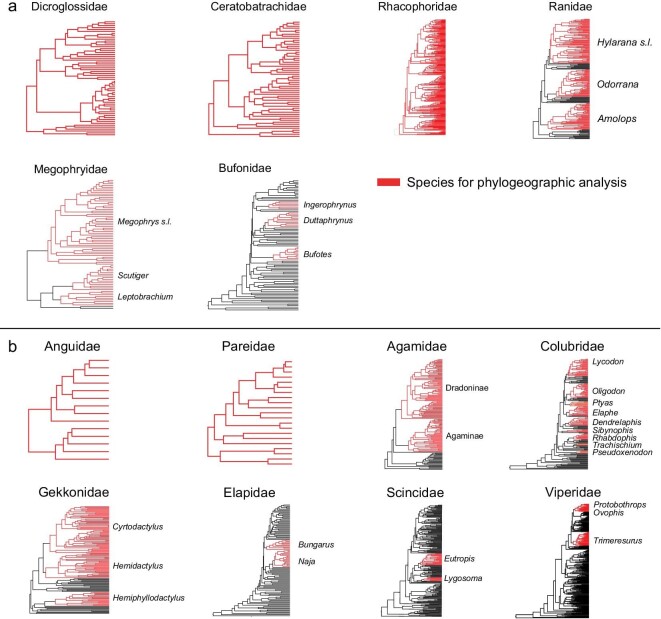
Overview of species used for our phylogeographic analysis. The 14 trees illustrate our family-level time trees: (a) for amphibians and (b) for reptiles.

Amphibians and reptiles are ideal organisms for studying biogeographic relationships and they generally retain high-resolution signals of historical responses to environmental changes [22]. They are often used to test geological and climate hypotheses [23,24]. In this study, we reconstructed 14 time-calibrated phylogenies of Himalayan-associated amphibian and reptile families, from which we analyzed 35 well-sampled subclades, to explore the spatiotemporal evolution of Himalayan amphibians (Fig. 3a) and reptiles (Fig. 3b) using a maximal number of observed diversification events (MDE) [25]. We then tested the major competing geological hypotheses (as shown in Fig. 2), and considered the effects of the South Asian Monsoon system on the Himalayan biota.

## RESULTS

### Relative rates and ages of biogeographic events

A total of 14 independent time-calibrated phylogenies of Himalayan-associated amphibian and reptile families (Fig. 3) involving 85 genera and 1628 species (Ranidae, Rhacophoridae, Dicroglossidae, Ceratobatrachidae, Bufonidae, Megophryidae, Agamidae, Anguidae, Pareidae, Colubridae, Elapidae, Gekkonidae, Scincidae and Viperidae; Supplementary Data S1) were reconstructed from MCMCTREE [26]. Among these, we estimated times of divergence among 183 species that occur in the Himalaya. The average ages of major biogeographic events are presented in Table 1 (see Supplementary Data S2 for evidence that our results were not strongly influenced by our choice of priors).

**Table 1.** Numbers and mean ages (with 95% confidence intervals) of different types of biogeographic events inferred in this study.

Biogeographic event type	Number	Mean age (95% confidence interval) (Ma)
*In situ* diversification^a^	126	17.28 (23.10–12.36)
Dispersal	87	19.18 (24.71–14.21)
Ambiguous events	17	28.21 (35.76–21.05)
From Southeast Asia into the Himalaya	49	19.95 (25.48–14.93)
From East Asia into the Himalaya	2	5.08 (7.77–3.13)
From South Asia into the Himalaya	8	19.99 (26.94–13.54)
From Central-West Asia into the Himalaya	2	47.8 (56.65–37.45)

^a^*In situ* diversification events and dispersal events were summarized based on the result of BioGeoBEARS analysis (Supplementary Data S3). Detailed dispersal events were summarized from results of the BioGeoBEARS analysis (Supplementary Data S4).

We identified 230 biogeographic events related to the Himalayan species based on the detailed biogeographic analysis of 14 families (Supplementary Table S1), including 126 *in situ* diversification events, 87 dispersal events and 17 ambiguous events (see definition of these biogeographic events in methods and materials in Supplementary Data and graphically shown in Supplementary Fig. S1). *In situ* diversification events contributed more than half of the events that gave rise to the Himalayan biota (126/230 = 54.78%). The mean age of these biogeographic events consistently clusters at ∼20 Ma (Table 1). In other words, diversification events in the Himalaya were most densely concentrated in the Miocene.

### Dynamics of *in situ* diversification and dispersal events

**Figure 4.**
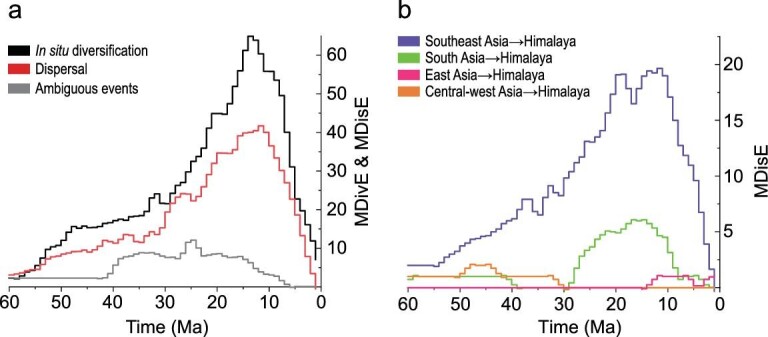
(a) The rates of *in situ* diversification and dispersal of the Himalayan herpetofauna through time (smoothed across 5 Ma windows). Dispersal indicates ‘dispersal into the Himalaya’. MDivE = maximal number of observed *in situ* diversification events per Ma. MDisE = maximal number of observed dispersal events per Ma. Ambiguous events are separately listed. (b) Dispersal events from adjacent regions into the Himalaya (smoothed across 5 Ma windows).

Our analysis revealed similar trends of *in situ* diversification and dispersal through time, estimated under an unconstrained biogeographic model (Fig. 4a). Both *in situ* diversification and dispersal events associated with the Himalaya began at approximately the same time around 70 Ma (Fig. 4a, Table 2). From 67–30 Ma (Paleocene to early Oligocene), the rate of both *in situ* diversification and dispersal was relatively low and increased slowly (Fig. 4a). During this period, the magnitude of *in situ* diversification was similar to dispersal. From 30 Ma (early Oligocene), *in situ* diversification and dispersal began to increase (Fig. 4a). Around 20 Ma (early Miocene), both processes (but especially *in situ* diversification) increased rapidly. Both rates peaked simultaneously around 13 Ma (middle Miocene) (Fig. 4a, Table 2). From each MDE curve, we detected two inflection points, indicating shifts in the diversification rate (Table 2). The dynamics of *in situ* diversification and dispersal rates remained essentially parallel across the Cenozoic. Both *in situ* diversification and dispersal rates showed a stepwise pattern of increases in the Paleocene and Miocene that best match the stepwise hypothesis for the formation of the Himalaya (Fig. 4a and Table 2). In contrast, our estimates of origination and peak diversification are not consistent with the late-uplift hypothesis.

**Table 2.** The empirical data and the predictions related to the biogeographic events under different geological models. 99% confidence intervals of the observation values are from 1000 bootstrap pseudoreplicates (see Supplementary Data S5).

		Predictions^a^ (Ma)	
Features of the diversification dynamics	Observations (99% confidence interval) (Ma)	Stepwise hypothesis	Late Orogeny hypothesis
Origination of MDivE	67 (67–54)		
Origination of MDisE	67 (67–54)		
		∼60	23
Peak of MDivE	13.5 (14–11)		
Peak of MDisE	12 (15–13)		
		∼15	5.3–2.5
Inflection points of MDivE	30 (35–11), 53 (56–27)		
Inflection points of MDisE	31 (39–11), 54 (58–27)		
		21–19, 56 (58–54)	None mentioned

^a^The prediction values are consistent with data used in Fig. 2c and d.

Focusing on the interchange between adjacent regions, Southeast Asia is the area with the highest frequency of interactions with the Himalaya (Fig. 4b, see details in Supplementary Table S2). Southeast Asia shared interchanges with the Himalaya in 49 dispersal events (49/62 = 79% of all dispersal events), whereas there were many fewer exchanges with other areas (Table 1). The Himalayan interchanges with Southeast Asia began by 67 Ma and then peaked at around 19 Ma (Fig. 4b).

An analysis that examined the effect of including ‘potential Himalayan species’ (Supplementary Fig. S2a) produced virtually identical results. We also repeated our analyses using BEAST [27] instead of MCMCTREE [26], again with virtually identical results (Supplementary Fig. S2b). Prior and posterior probabilities from BEAST and MCMCTREE are compared in Supplementary Data S2, and indicate that our sequence data were informative.

## DISCUSSION

### Testing geological models for the origin of the Himalaya and subsequent monsoon intensification

Mountain building has long been viewed as an important direct driver of speciation [28]. Rapid uplift results in widespread isolation and speciation of ancestral populations. In addition, the origin of montane areas can create a new source and sink for dispersal from other montane areas. In our analyses of the herpetofauna (Fig. 4), both the *in situ* diversification rate, as well as the dispersal rate into the Himalaya, fit the Stepwise model [8] for the origin of this mountain range. In this model (Fig. 2a), the initial uplift of the Himalaya began in the Paleocene (∼60 Ma), coinciding with the 99% range of start of biota assembly process (67–54 Ma for *in situ* diversification and dispersal, Table 2). The Himalaya then grew slowly and gradually at first, but then rapidly increased in the Miocene until approximately 13 Ma, when they reached their current elevations (∼6000 m and still rising). Correspondingly, we detected significant inflection points in the rates of *in situ* diversification and dispersal that are broadly consistent with the expectations under the Stepwise model (Table 2). The early gradual uplift of the Himalaya was accompanied by a gradual increase in the diversification of the herpetofauna, as well as a gradual increase of dispersal events into the Himalaya. We also found a rapid increase of the diversification rate in the Miocene, consistent with a pulse of uplift-driven diversification (Fig. 2c).

In contrast, our results are inconsistent with the Late Orogeny hypothesis for Himalayan origins shown in Fig. 2b. For the Late Orogeny hypothesis, the expected origination of Himalayan biota diversification (23 Ma) is much later than we observed (∼60 Ma, Table 2). Furthermore, the occurrence of several inflection points in the MDE suggested non-gradual mountain building, contrasting with the predictions of the Late Orogeny hypothesis (Table 2). Therefore, our data broadly support the Stepwise model of Ding *et al.* [8], but not the Late Orogeny hypothesis.

In contrast to our findings of a rapid increase in diversification rates of the Himalayan herpetofauna in the Miocene, Xing and Ree [13] found no signal for rapid diversification of the Himalayan flora, which indicated that the Himalayan uplift may have been gradual rather than episodic. Interestingly, a recent study by Ding *et al.* [15] did detect acceleration of *in situ* speciation on alpine flora in the Himalaya during the Miocene. Generally, compared with plants, amphibians and reptiles are more sensitive to environmental changes and geographic isolation. Furthermore, long-distance pollen dispersal is more likely to slow vicariance of plant populations relative to that of amphibian and reptile populations [29]. Birds are also well-sampled across the Himalaya. However, the timescale for the diversification of present-day bird communities is largely limited to the Late Miocene [25,30], so diversification of these communities does not cover the scope of the competing geological models tested here.

Geological, climatological and biological changes are highly correlated [14]. The combination of complex topography and varying climates produces a biodiversity hotspot in the Himalaya [14]. The rapid Himalayan uplift and associated intensified South Asian Monsoon not only promoted a pulse of uplift-driven *in situ* diversification, but also affected the rates of biotic interchange (Fig. 4). As the Himalaya rose, the northward flow of moist air from the Indian Ocean was blocked by the high Himalaya and/or deflected to the east [14], and may have also given rise to the modern South Asian Monsoon [4,14,31,32]. These changes resulted in the aridification of central Asia starting from the Early Miocene [3], and ecological barriers in the eastern Himalaya since the Miocene [33]. Consequently, these changes may have limited dispersal between the Himalaya and Central-West Asia, South Asia and East Asia. In support of this scenario, we found much lower rates of dispersal of amphibians and reptiles between the Himalayan region and Central-West Asia, South Asia and East Asia, compared to the relatively high rates of dispersal between the Himalaya and Southeast Asia (Fig. 4b). Biotic interchange was likely restricted by the lack of a moist environment required by many reptiles and amphibians. In contrast, an expanded tropical forest belt is thought to have persisted between the Himalaya and Southeast Asia since the middle Miocene [16], which likely accounts for the high dispersal rates between these two regions (Fig. 4b).

In addition to the uplift of the Himalaya and the development of the monsoon system discussed above, global cooling may have contributed to extinction, which would have lowered the net diversification rates [34]. Although assembly patterns of the Himalayan herpetofauna do not closely match the global cooling trend, global cooling may have affected the diversification patterns we observed. For example, simulations [35] have shown that extinction caused by global cooling can potentially lead to patterns similar to the Miocene burst of Himalayan herpetofauna we observed. Many fossils have been found near Lunpola, central Tibet, which indicate this area experienced dramatic changes from a wet environment to the current dry environment [7,36]. However, there is no similar evidence of such a shift for the Himalaya.

### How did the Himalayan fauna assemble?

Our analyses show a deep-rooted origin of Himalayan herpetofauna originating in the Paleocene, but with rapid diversification in the Miocene. These findings are broadly consistent with the hypothesis proposed by Spicer [14] to explain Himalayan biodiversity. In a recent critical review, Renner [37] argued that numerous phylogenetic studies incorrectly attributed relatively young ages (Miocene and later) for lineage divergence due to the uplift of the Qinghai–Tibetan Plateau. The references discussed by Renner [37] often combined different regions as the Qinghai–Tibetan Plateau and did not differentiate them. Following the advice of Renner [37], here we focus on the biota of Himalaya, which has a very different history from other parts of the Qinghai–Tibetan Plateau.

Our analyses quantify the relative contributions of *in situ* diversification and dispersal, which requires extensive sampling of both regions and taxa. Prior studies that focused on groups with high dispersal ability, or only on local endemics, have led to the conclusion that the Himalaya are a dispersal sink [18,19]. For example, cases of long-distance dispersal from the mountains of China–Indochina along the southern slope of the Himalayan chains have been reported, associated with very little *in situ* speciation [38]. However, the sink hypothesis is not sufficient to explain the origins of groups with lower dispersal ability, such as reptiles and amphibians. We found both *in situ* speciation and long-term dispersal were important processes for assembly of the herpetofauna. In groups with low vagility, *in situ* speciation is generally considered to be a more likely explanation for faunal assembly [20]. Our finding that most biotic dispersal of the Himalayan herpetofauna has been between the Himalaya and Southeast Asia has important conservation implications. Long-term maintenance of biotic diversity in the Himalaya likely depends on the preservation of a dispersal corridor between these two areas. Therefore, protection of this dispersal corridor should be an international conservation priority.

## MATERIALS AND METHODS

Please refer to the Supplementary data online.

## Supplementary Material

nwaa263_Supplemental_File
