# Supplementary material for: Herpetological phylogeographic analyses support a Miocene focal point of Himalayan uplift and biological diversification
Source: Natl Sci Rev. 2020 Oct 21;8(9):nwaa263. doi: 10.1093/nsr/nwaa263 (PMC8433089; doi:10.1093/nsr/nwaa263)
Supplement: nwaa263_Supplemental_File [file nwaa263_supplemental_file.pdf]

Supplementary Materials for

Herpetological Phylogeographic Analyses Support a Miocene Focal Point of Himalayan Uplift and Biological Diversification

Wei Xu[†,‡], Wen-Jie Dong[†,‡], Ting-Ting Fu[†,‡], Wei Gao[†,‡], Chen-Qi Lu[†,‡], Fang Yan[†], Yun-He Wu[†,‡], Ke Jiang[†], Jie-Qiong Jin[†], Hong-Man Chen[†], Ya-Ping Zhang[†,‖], David M. Hillis[§,*], and Jing Che[†,‖,*]

Correspondence to: chej@mail.kiz.ac.cn; dhillis@austin.utexas.edu.

This PDF file includes:

Methods and Materials

Supplementary Text

Fig. S1 to S2

Table S1 to S5

Data S1 to S8

References

Methods and Materials

Data collection

The complete dataset for phylogenetic analyses is presented in **DataS5**. Of the included 1,628 species, 184 are distributed in the Himalaya, and 127 are endemic (limited to the Himalaya). Those fourteen families met the following criteria, as suggested in Xing and Ree [1]: (1) the clade contains substantial numbers of species distributed in the Himalaya; (2) sufficient molecular data were available or obtainable to infer a phylogeny that covered majority taxonomic diversity and geographic range of this clade; and (3) sufficient fossil data were available for molecular clock calibration of the clade's diversification. Thirty-five well-resolved clades with Himalaya species of those families are selected for biogeographic analysis (**Fig. 3**). We integrated closelyrelated clades of the same family into a combined family-level tree, instead of building trees for each subgroup separately. This maximized use of shared fossil calibrations among genera; otherwise, further splitting of the trees would have reduced available fossil calibration points and thus increased the uncertainly of age estimates.

The DNA dataset for phylogenetic analysis included 29 loci with 8,156 sequences (**DataS5**). We collected 391 new sequences from 121 species, and downloaded the remaining sequences from GenBank (through August 2019). Total genomic DNA was extracted using a standard phenol/chloroform method [2] for the 121 species newly sampled in this study. Amplification consisted of an initial denaturation step at 95℃ for 5 min, followed by 35 cycles of denaturation at 95℃ for 1 min, annealing for 1 min, followed by extension at 72℃ for 1 min. The final extension was set at 72℃ for 10 min. PCR products were purified with a Gel Extraction Mini Kit (Watson BioTechnologies, Shanghai, China), and then sequenced in both directions with BigDye Terminator Cycle Sequencing Kit (v.2.0, Applied Biosystems, Foster City, CA, USA) using an ABI PRISM 3730 DNA Analyzer (Applied Biosystems). Primers and conditions for PCR amplification are listed in **Table S3**.

Our study was approved by the ethics committees of the Kunming Institute of Zoology, Chinese Academy of Sciences, China (permission for this study is BBCJ-2014-001).

Time calibrated phylogeny

All sequences were aligned using ClustalW with default settings as implemented in MEGA X [3]. Multiple loci were concatenated using SequenceMatrix [4]. The assembled supermatrices for

each family were used to construct phylogenetic trees under the maximum likelihood (ML) criterion, using IQTREE [5] in the IQTREE web server [6]. We used ModelFinder [7] to determine the best substitution model for each gene partition (**Table S4**). We calculated bootstrap proportions and other branch-support metrics (all based on 1,000 replicates) using the UFBoot [8] algorithm in IQTREE 1.5.5, UFBoot2 [9] in IQTREE 1.6.1, aLRT [10], and its nonparametric equivalent SH-aLRT [11]. The Bayesian measures of branch support reduce the bias of bootstrap support values in large datasets [12].

Fourteen time-calibrated trees were constructed using MCMCTREE [13]. The inferred IQTREE tree was used as the reference topology. We first used BASEML program (in PAML) to estimate the mean substitution rate under GTR + I substitution model, using a strict molecular clock. We assigned each tree a root age based on a published large-scale tree [14,15] or fossil records. The average estimated rates were then used to set the prior of overall substitution rate (*rgene gamma*) and rate-drift parameter (*sigma2 gamma*). We used 40 well-established fossil ages to calibrate the date of nodes (fossil details are presented in **Table S5** and graphically shown in **Data S6**). The minimum and maximum bounds of constraints were treated as soft, with a 2.5% probability that those bounds could be violated. The first 5,000,000 generations of the MCMC sample were treated as burn-in; we then then sampled every 1,000 generations for another 10,000,000 generations to create a sample of 10,000 solutions. We executed this analysis twice to check for convergence in the solution set, starting from randomly generated seeds.

Comparison between prior and posterior distributions are needed in any Bayesian analysis to determine whether signal is coming from the data *per se* or from the prior selection, especially for parameters of interest [16]. We compared time estimates of calibration nodes based on prior-only and data-informed distributions. Prior distributions were obtained with calibrations only, using no sequence data. Other parameters were the same in both analyses. All results were processed in R v4.0.1 [17] and were visualized using ggplot2 v3.3.1 [18].

Definition of biogeographic regions

We analyzed nine biogeographic regions.The Himalayan Mountain ranges region, following Boos and Kuang[19] and Ding et al. [20], is bounded to the west by Nanga Parbat, to the north by the northernmost bend of Indus river, and to the east by Namcha Barwa and the great bend of the Yarlung Tsangpo River. It is bordered on the northwest by the Karakoram and the Hindu

Kush ranges, and to the north the Himalaya are separated from the Tibetan Plateau by a 50−60 km wide tectonic valley called the Indus–Tsangpo Suture. To the south the arc of the Himalaya is ringed by the low Indo-Gangetic Plain. We included species distributed in northeast India, northern Myanmar and Ganges Delta in the Himalayan Mountain region because they are adjacent and have a similar mountain environment. Importantly, we strictly differentiate the Himalaya from high Tibetan Plateau due to their clear geological distinction in the spirit of Spicer [21]. The other regions we included were temperate/boreal East Asia (the eastern boreal part of Russia and temperate regions of eastern Asia, including Japan and Taiwan); Central-West Asia (including Pakistan, Uzbekistan, Tajikistan, and the Arabian Peninsula countries); Southeast Asia (including tropical regions of China, Malesia, and Papuasia); Australasia (Australia, New Zealand, and the southwestern Pacific); South Asia (south of the Himalaya, including Sri Lanka); Africa; Europe; and the Americas (North America, Central America, and South America).

Ancestral range reconstruction

We reconstructed ancestral ranges as follows. First, to infer the differences between the dynamics of *in situ* diversification versus dispersal-associated diversification, we classified species into either Himalayan (state "A") or non-Himalayan (state "B") regions, based on their current known distributions (species that occurs both inside and outside of the Himalayan region were coded "AB"). We then fit six models (DEC, DEC+j, DIVALIKE, DIVALIKE+j, BAYAREALIKE, BAYAREALIKE+j) implemented in BioGeoBEARS [22] using *model-test* function [23] to test which models should be the best based on AICc weight. The best model was applied to infer the probabilities for the ancestral range of each node in the tree as being Himalayan or non-Himalayan. We also classified species into the nine regions to provide additional information about dispersal between the Himalaya and other adjacent regions.

Phylogeographic analysis: Local diversification and dispersal

Biotic assembly for a given region is the process of species accumulation, which is composed of both colonization via regional dispersal as well as *in situ* lineage diversification. Although extinction may affect overall diversification rate, observed *in situ* lineage diversification is all that can be estimated from extant taxa.

Using the ancestral range analysis, we categorized several biogeographic events on the phylogenetic tree (**Fig. S1**)[24]. We identified "*in situ* diversification events" whenever the ancestral node for a group of descendant Himalayan species was reconstructed in the Himalaya; the date of that ancestral node was taken as the date of the *in situ* diversification events. In contrast, we identified of dispersal between areas if the range of a descendant node differed from the reconstructed range of the ancestral node. More specifically, a biogeographic event was identified as "dispersal into the Himalaya" in cases in which the ancestral range was reconstructed as other than the Himalaya, whereas the descendant node was in the Himalaya. In the case of biogeographic events associated with dispersal, the date of the dispersal event was recorded as the date of the older (ancestral) node. Some events could not be assigned unambiguously to either *in situ* diversification events or dispersal events because of ambiguity in the ancestral node assignments. In all cases, we refer to a node as being either a tip (that is, extant species) or an internal node (ancestral species). Some diversification events from clades outside our selected clades, but possibly related to the Himalaya, are separately defined as "ambiguous events" (see details in **Supplementary Text**).

We estimated the maximal number of observed dispersal events per Ma (MDisE) [25] or *in situ* diversification events per Ma (MDivE, extension of MDE) to infer general patterns of *in situ* diversification and biotic interchange. These MDE rates were calculated by summing potential dispersal or *in situ* diversification events over all data sets through time using time slices of one million years (based on the divergence time credibility intervals given in **Table S1** and **Table S2**). All dispersal and *in situ* diversification events (based on different nodes in the tree) were treated as independent. In addition to estimating events from the MCMCTREE results, we also conductedan MDE analysis based on results from BEAST to consider method sensitivity (see more details in **Supplementary Text**).

We estimated inflection points in the MDE curves from 67 Ma to the present to characterize major changes in diversification rates over time (using the *e.divisive* function of R-package 'ecp') [26, 27, 28]. We resampled 1,000 bootstrap pseudoreplicates from the raw credibility intervals (used for inferring the MDisE and MDivE rates above) using the *sample* function R 3.3.2 (**Supplementary Text**). We repeated the calculations to find the MDE origination, peaks, and inflection points for each bootstrap pseudosample, and used these samples to calculate

confidence intervals for the MDE curve attributes presented in **Table 2**(details see **Supplementary Text**).

Bootstrap analyses

The inflection points were identified using the *e.divisive* function in R-package *ecp* with the following parameters: maximum number of random permutations = 1,000, significance level = 0.001, k=2 (maximum possible inflection points are set to 2, corresponding with possible inflection points in those geological models), minimum number of observations between changes = 10 Ma. We estimated the origination, peak, and inflection points from each bootstrap pseudosample and repeated this calculation for 1,000 bootstrap pseudoreplicates (**Data S7**). We sorted these samples ascending and calculate the 99% confidence intervals of MDE curve attributes.

Testing the effect of "potential Himalayan species"

Some species are excluded from our analysis due to their questionable distribution in the Himalaya. Most of these are widespread species, with no molecular data published from Himalayan samples since their original description. We consider their presence in the Himalaya uncertain. Therefore, we considered them as non-Himalayan species in our biogeographic analysis, but labeled them "potential Himalayan species" (details see **Data S8**). It is possible that some of them might be confirmed as true Himalayan species in the future. Therefore, to examine the effects of their exclusion in our analyses, we used molecular data from regions outside the Himalaya and integrated these species into our MDE analysis to check if these exclusions changed any of our results.

Supplemental Text

Defining "ambiguous events" and effect

We selected well-resolved clades for analysis. Those clades with Himalayan species but poorly-resolved or with too few species (fewer than three species) were excluded (*Ophiophagus* for example, has only one species, *Ophiophagus hannah*), to minimize bias from extinction or poor sampling. Furthermore, Himalayan species in poorly-resolved clades (e.g., *Sphenomorphus*) have a highly uncertain phylogenetic position, so they were also excluded. The MDE trend of those

“ambiguous events” (**Fig. 4**) shows that they occupy only small fraction of all events. Generally, those events are likely dispersal events if those clades are truly monotypic taxa. Adding these events would lead to a similar trend but a somewhat earlier increased dispersal MDE curve.

Effect of “potential Himalayan species”

When “potential Himalayan species” were included in the analysis, the trends remain unchanged (**Fig. S2a**). The origination, peak, and time period of rapid increase were all unchanged. The integration of those species only changed the peak value of dispersal, which made it almost equivalent in scale with *in situ* diversification (**Fig. S2a**).

Repeat validation using BEAST

To test the robustness of the divergence time estimation, we added BEAST v 1.10.4 [29]. We used PartitionFinder2 [30] to find the best substitution model for each gene partition under BIC. The best-fit partitioning schemes determined by PartitionFinder2 was applied to the BEAST. We used the same calibration of MCMCTREE. BEAST analyses were run for 100 million generations, with samples taken every 10,000 generations under an uncorrelated lognormal relaxed clock and a Yule tree prior. Stationarity was assessed using TRACER 1.7.0 [31] with ESS values > 200 taken as evidence for convergence. Maximum clade credibility trees were obtained using TREEANNOTATOR 2.4.1 [32] applying a burn-in of 25%.

Biogeographic events detected from BEAST trees are generally similar to those obtained with MCMCTREE, including 133 *in situ* diversification events (mean age with 95% confidence interval: 16.15 Ma [21.90 Ma – 11.17 Ma]), 88 dispersal events (mean age with 95% confidence interval: 23.28 Ma [32.37 Ma – 16.12 Ma]), and 12 ambiguous events (mean age with 95% confidence interval: 30.29 Ma [42.17 Ma – 20.61 Ma]). A few questionable events are excluded due to their large 95% confidence intervals (some are more than 50 Ma). The MDE trend is also similar to the results with MCMCTREE (**Fig. 4**)

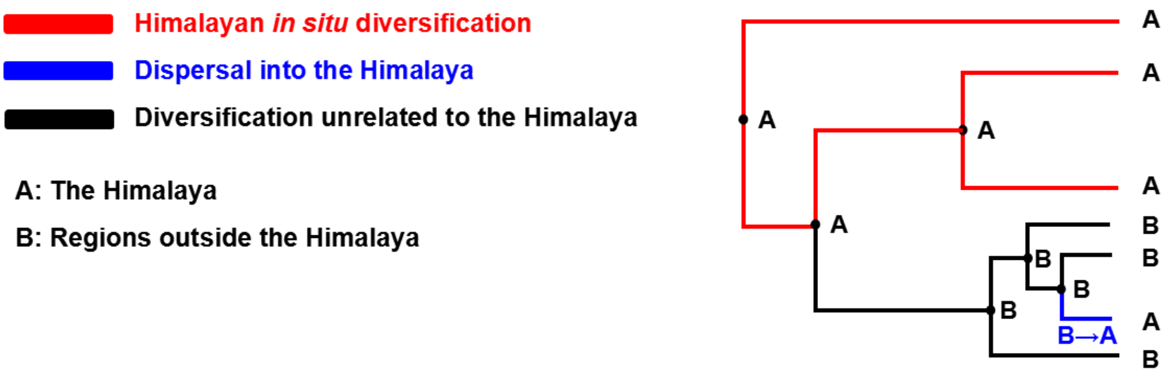

Fig. S1. Identification of *in situ* diversification events and dispersal events. An example phylogenetic tree is shown with the ancestral areas depicted for each node. A: the Himalaya; B: non-Himalayan regions. Red branches represent *in situ* diversification events, blue branches represent dispersal events, and black branches were not related to Himalayan diversification.

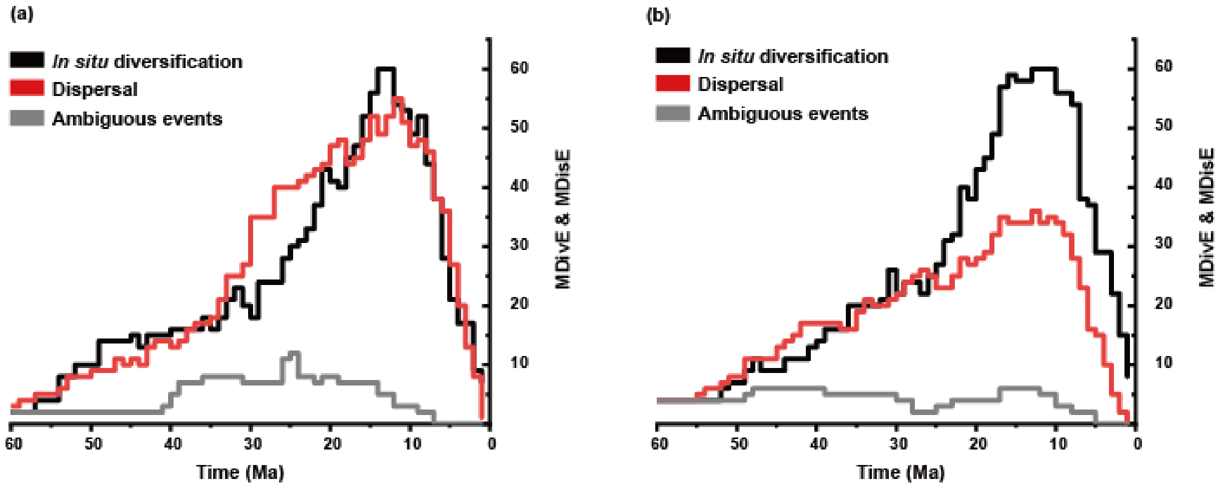

Fig. S2. (a): The rates of *in situ* diversification and dispersal of the Himalayan herpetofauna through time after adding those "potential Himalayan species." MDivE = maximal number of observed *in situ* diversification events per Ma. MDisE = maximal number of observed dispersal events per Ma. (b): The rates of *in situ* diversification and dispersal of the Himalayan herpetofauna through time from BEAST. MDivE = maximal number of observed *in situ* diversification events per Ma. MDisE = maximal number of observed dispersal events per Ma.

Table S1.

Inferred *in situ* diversification events and dispersal events related to the Himalaya summarized from **Data S3**. Respective clade, median, upper and lower bounds of the 95% intervals for the age.

Clades	Median (Myr)	Upper (Myr)	Lower (Myr)	Events
Gekkonidae	9.11	12.86	6.04	*In situ* diversification
Gekkonidae	9.11	12.86	6.04	*In situ* diversification
Gekkonidae	20.26	25.86	15.81	*In situ* diversification
Gekkonidae	20.26	25.86	15.81	*In situ* diversification
Gekkonidae	23.39	29.38	18.91	Dispersal
Gekkonidae	25.58	31.08	20.48	*In situ* diversification
Gekkonidae	25.58	31.08	20.48	*In situ* diversification
Gekkonidae	30.01	35.82	24.58	*In situ* diversification
Gekkonidae	30.01	35.82	24.58	*In situ* diversification
Gekkonidae	31.49	37.28	26.08	*In situ* diversification
Gekkonidae	31.49	37.28	26.08	*In situ* diversification
Gekkonidae	22.87	28.25	18.07	*In situ* diversification
Gekkonidae	22.87	28.25	18.07	*In situ* diversification
Gekkonidae	18.94	23.8	14.65	*In situ* diversification
Gekkonidae	18.94	23.8	14.65	*In situ* diversification
Gekkonidae	9.18	14	5.34	*In situ* diversification
Gekkonidae	16.19	20.9	12.08	*In situ* diversification
Gekkonidae	16.19	20.9	12.08	*In situ* diversification
Gekkonidae	6.2	10.89	2.71	*In situ* diversification
Gekkonidae	6.2	10.89	2.71	*In situ* diversification
Gekkonidae	33.61	39.63	27.83	*In situ* diversification
Gekkonidae	36.49	42.83	30.42	*In situ* diversification
Gekkonidae	36.49	42.83	30.42	*In situ* diversification
Gekkonidae	3.64	5.93	2.02	*In situ* diversification
Gekkonidae	3.64	5.93	2.02	*In situ* diversification
Gekkonidae	41.81	48.53	35.29	*In situ* diversification
Gekkonidae	41.81	48.53	35.29	*In situ* diversification
Gekkonidae	37.76	44.67	31.11	*In situ* diversification
Gekkonidae	26.05	32.91	19.55	*In situ* diversification
Gekkonidae	17.13	23.38	11.83	*In situ* diversification
Gekkonidae	44.87	53.52	38.96	*In situ* diversification
Gekkonidae	44.87	53.52	38.96	*In situ* diversification
Gekkonidae	55.41	66.68	44.65	*In situ* diversification
Gekkonidae	55.41	66.68	44.65	*In situ* diversification

Gekkonidae	23.31	32.9	15.45	*In situ* diversification
Gekkonidae	23.31	32.9	15.45	*In situ* diversification
Gekkonidae	21.56	28.31	16.09	*In situ* diversification
Gekkonidae	21.56	28.31	16.09	*In situ* diversification
Gekkonidae	17.67	24.34	12.25	*In situ* diversification
Gekkonidae	17.67	24.34	12.25	*In situ* diversification
Gekkonidae	18.35	25.04	12.7	*In situ* diversification
Gekkonidae	18.35	25.04	12.7	*In situ* diversification
Gekkonidae	23.65	29.72	18.08	Dispersal
Gekkonidae	15.42	22.4	9.49	Dispersal
Gekkonidae	35.27	42.79	28.2	Dispersal
Gekkonidae	14.04	21.89	7.8	Dispersal
Gekkonidae	32.26	40.69	24.52	Ambiguous events
Gekkonidae	49.54	60.03	40.56	Ambiguous events
Gekkonidae	55.08	65.99	45.65	Ambiguous events
Gekkonidae	14.98	20.55	10.19	Dispersal
Dicroglossidae	11.19	16.66	6.74	Dispersal
Dicroglossidae	19.4	27.58	11.79	Dispersal
Dicroglossidae	12.09	16.54	8.53	Dispersal
Dicroglossidae	9.47	14.06	5.59	Dispersal
Dicroglossidae	9.47	14.06	5.59	Dispersal
Dicroglossidae	24.22	30.22	18.78	Dispersal
Dicroglossidae	0.45	1.12	0.08	*In situ* diversification
Dicroglossidae	0.45	1.12	0.08	*In situ* diversification
Dicroglossidae	3.4	5.25	2.03	Dispersal
Dicroglossidae	3.4	5.25	2.03	*In situ* diversification
Dicroglossidae	6.36	8.63	4.48	*In situ* diversification
Dicroglossidae	6.36	8.63	4.48	*In situ* diversification
Dicroglossidae	4.55	6.77	2.79	*In situ* diversification
Dicroglossidae	4.55	6.77	2.79	*In situ* diversification
Dicroglossidae	8.54	11.1	6.34	*In situ* diversification
Dicroglossidae	8.54	11.1	6.34	*In situ* diversification
Dicroglossidae	1.67	3.27	0.62	*In situ* diversification
Dicroglossidae	4.51	6.97	2.54	*In situ* diversification
Dicroglossidae	14.35	17.57	11.63	Dispersal
Dicroglossidae	45.83	53.34	37.33	Dispersal
Bufonidae	13.38	20.38	7.56	Dispersal
Bufonidae	6.71	10.42	3.9	Dispersal
Bufonidae	14.25	18.62	10.59	Dispersal
Bufonidae	14.85	19.77	10.27	Dispersal
Bufonidae	16.69	24.28	9.23	Ambiguous events

Bufonidae	1.74	3.6	0.65	*In situ* diversification
Bufonidae	1.74	3.6	0.65	*In situ* diversification
Bufonidae	7.53	12.06	4.32	Dispersal
Bufonidae	27.82	35.18	19.97	Ambiguous events
Pareidae	51.32	66.28	37.72	Dispersal
Megophryidae	25.73	29.68	21.2	Dispersal
Megophryidae	12.95	21.07	6.09	*In situ* diversification
Megophryidae	12.95	21.07	6.09	*In situ* diversification
Megophryidae	8.52	12.57	5.25	Dispersal
Megophryidae	10.26	14.45	6.69	*In situ* diversification
Megophryidae	11.63	15.78	8.06	*In situ* diversification
Megophryidae	11.63	15.78	8.06	*In situ* diversification
Megophryidae	8.63	13.31	4.23	*In situ* diversification
Megophryidae	13.31	17.69	9.56	*In situ* diversification
Megophryidae	13.31	17.69	9.56	*In situ* diversification
Megophryidae	11.98	16.58	8.01	*In situ* diversification
Megophryidae	11.98	16.58	8.01	*In situ* diversification
Megophryidae	9.86	14.34	5.98	*In situ* diversification
Megophryidae	9.86	14.34	5.98	*In situ* diversification
Megophryidae	4.82	8.97	2	*In situ* diversification
Megophryidae	4.82	8.97	2	*In situ* diversification
Megophryidae	16.16	20.85	11.69	*In situ* diversification
Megophryidae	16.16	20.85	11.69	*In situ* diversification
Megophryidae	20.48	24.77	16.15	*In situ* diversification
Megophryidae	20.48	24.77	16.15	*In situ* diversification
Megophryidae	16.48	21.24	11.93	*In situ* diversification
Megophryidae	16.48	21.24	11.93	*In situ* diversification
Megophryidae	12.9	17.65	8.64	*In situ* diversification
Megophryidae	12.9	17.65	8.64	*In situ* diversification
Megophryidae	10.12	15.06	5.71	*In situ* diversification
Megophryidae	10.12	15.06	5.71	*In situ* diversification
Megophryidae	10.59	15.52	6.15	*In situ* diversification
Megophryidae	10.59	15.52	6.15	*In situ* diversification
Megophryidae	22.6	26.81	18.17	*In situ* diversification
Megophryidae	25.85	29.62	21.55	Dispersal
Megophryidae	5.14	8.22	2.96	Dispersal
Megophryidae	11.17	15.84	7.5	*In situ* diversification
Megophryidae	12.74	17.45	9.03	*In situ* diversification
Megophryidae	12.74	17.45	9.03	*In situ* diversification
Megophryidae	11.14	15.99	7.78	*In situ* diversification
Megophryidae	11.14	15.99	7.78	*In situ* diversification

Megophryidae	10.43	14.91	6.81	*In situ* diversification
Megophryidae	10.43	14.91	6.81	*In situ* diversification
Megophryidae	7.47	11.54	4.29	*In situ* diversification
Megophryidae	7.47	11.54	4.29	*In situ* diversification
Megophryidae	5.07	8.72	2.43	*In situ* diversification
Megophryidae	14.27	19.49	10.18	Dispersal
Megophryidae	19.77	26.03	14.11	Dispersal
Megophryidae	18.12	24.42	11.92	Dispersal
Viperidae	3.73	6.16	2	Dispersal
Viperidae	15.37	20.32	10.91	Dispersal
Viperidae	24.86	29.43	20.49	Dispersal
Viperidae	9.75	12.41	7.49	Dispersal
Viperidae	18.51	22.32	15.08	Dispersal
Viperidae	22.68	26.94	18.74	Dispersal
Agamidae	13.88	22.51	7.76	*In situ* diversification
Agamidae	13.88	22.51	7.76	*In situ* diversification
Agamidae	51.25	59.79	40.79	Dispersal
Agamidae	34.2	42.93	25.99	Dispersal
Agamidae	12.87	17.36	9.18	Dispersal
Agamidae	31.86	38.17	26.06	Dispersal
Agamidae	54.67	62.68	46.62	Dispersal
Agamidae	43.83	56.1	31.97	*In situ* diversification
Agamidae	43.83	56.1	31.97	*In situ* diversification
Agamidae	37.67	51.35	25.64	*In situ* diversification
Agamidae	37.67	51.35	25.64	*In situ* diversification
Agamidae	40.63	53.48	29	*In situ* diversification
Agamidae	40.63	53.48	29	*In situ* diversification
Agamidae	34.77	48.74	23.56	*In situ* diversification
Agamidae	34.77	48.74	23.56	*In situ* diversification
Agamidae	38.66	52.33	26.49	Dispersal
Agamidae	9.04	12.97	5.9	Dispersal
Agamidae	22.01	33.17	12.63	*In situ* diversification
Agamidae	22.01	33.17	12.63	*In situ* diversification
Anguidae	27.97	37.27	19.92	Dispersal
Scincidae	38.51	44.75	31.65	Ambiguous events
Scincidae	32.27	39.52	24.87	Ambiguous events
Scincidae	32.27	39.52	24.87	Ambiguous events
Scincidae	51.42	56.62	44.32	Dispersal
Scincidae	19.81	26.46	13.57	Dispersal
Scincidae	30.49	36.42	24.97	Dispersal
Scincidae	26.62	33.98	18.79	Dispersal

Scincidae	30.49	38.71	21.96	Ambiguous events
Scincidae	25.32	32.66	18.61	Dispersal
Rhacophoridae	5.48	9.36	2.64	Dispersal
Rhacophoridae	41.45	46.72	35.66	Dispersal
Rhacophoridae	17.99	27.88	10.57	*In situ* diversification
Rhacophoridae	17.99	27.88	10.57	*In situ* diversification
Rhacophoridae	6.61	9.59	4	Dispersal
Rhacophoridae	8.34	11.69	4.87	Dispersal
Rhacophoridae	12.99	17.29	8.8	Dispersal
Rhacophoridae	9.21	14.69	5.01	Dispersal
Rhacophoridae	23.34	29.64	17.42	Dispersal
Rhacophoridae	5.02	7.27	3.25	Dispersal
Rhacophoridae	5.02	7.27	3.25	Dispersal
Rhacophoridae	14.4	19.98	9.14	Dispersal
Rhacophoridae	26.97	33.33	20.29	Dispersal
Rhacophoridae	43.42	49.03	36.74	Dispersal
Rhacophoridae	9.16	13.39	5.29	*In situ* diversification
Rhacophoridae	9.16	13.39	5.29	*In situ* diversification
Rhacophoridae	11.98	15.9	8.33	Dispersal
Rhacophoridae	15.79	21.03	10.33	Dispersal
Ranidae	14.49	20.45	9.08	Dispersal
Ranidae	27.57	32.88	21.7	Dispersal
Ranidae	5.46	10.92	1.8	Dispersal
Ranidae	36.76	41.27	32.36	Dispersal
Ranidae	7.66	14.04	2.74	*In situ* diversification
Ranidae	12.05	17.64	7.16	*In situ* diversification
Ranidae	12.05	17.64	7.16	*In situ* diversification
Ranidae	7.1	10.73	4.25	*In situ* diversification
Ranidae	7.1	10.73	4.25	*In situ* diversification
Ranidae	9.44	13.42	6.33	*In situ* diversification
Ranidae	9.44	13.42	6.33	*In situ* diversification
Ranidae	10.64	14.78	7.28	*In situ* diversification
Ranidae	10.64	14.78	7.28	*In situ* diversification
Ranidae	21.42	26.82	16.62	Dispersal
Ranidae	14.78	21.79	8.7	Dispersal
Ceratobatrachidae	19.9	28.49	12.83	*In situ* diversification
Ceratobatrachidae	19.9	28.49	12.83	*In situ* diversification
Ceratobatrachidae	13.79	20.82	8.12	*In situ* diversification
Ceratobatrachidae	13.79	20.82	8.12	*In situ* diversification
Ceratobatrachidae	3.26	8.19	0.5	*In situ* diversification
Ceratobatrachidae	3.26	8.19	0.5	*In situ* diversification

Elapidae	17.97	23.62	13.04	Dispersal
Elapidae	12.76	18.54	7.92	Dispersal
Elapidae	9.81	19.88	3.47	Dispersal
Elapidae	11.47	16.7	7.52	Dispersal
Elapidae	31.65	39.12	22.2	Ambiguous events
Colubridae	7.91	12.59	4.38	Dispersal
Colubridae	11.32	15.26	8.01	Dispersal
Colubridae	19.02	24.32	14.01	Dispersal
Colubridae	15.81	20.21	11.82	Dispersal
Colubridae	10.82	16.57	6.65	Dispersal
Colubridae	5.84	11.07	2.49	Dispersal
Colubridae	21.18	28.78	13.82	Dispersal
Colubridae	31.08	37.01	25.39	Dispersal
Colubridae	31.77	38.51	24.19	Ambiguous events
Colubridae	13.91	19.65	8.78	Dispersal
Colubridae	4.17	7.05	2.26	Dispersal
Colubridae	9.29	14.7	5.29	Dispersal
Colubridae	5.64	8.69	3.32	Dispersal
Colubridae	17.98	22.69	13.59	Dispersal
Colubridae	31.92	37.42	26.76	Dispersal
Colubridae	19.44	25.24	14.04	Ambiguous events
Colubridae	19.44	25.24	14.04	Ambiguous events
Colubridae	38.81	44.8	32.94	Dispersal
Colubridae	15.93	22.27	10.71	*In situ* diversification
Colubridae	15.93	22.27	10.71	*In situ* diversification
Colubridae	19.01	25.49	13.51	*In situ* diversification
Colubridae	19.01	25.49	13.51	*In situ* diversification
Colubridae	13.33	20.07	7.54	Ambiguous events
Colubridae	13.33	20.07	7.54	Ambiguous events
Colubridae	18.7	25.53	12.51	Ambiguous events
Colubridae	18.7	25.53	12.51	Ambiguous events
Colubridae	10.59	16.55	6.3	Dispersal

Table S2.

Inferred dispersal events between the Himalaya and surrounding regions summarized from **Data S4**. Respective clade, median, upper and lower bounds of the 95% intervals for the age of events in million years before present (Ma), as used for the calculation of the maximal number of dispersal (MDisE) per Ma. The arrows indicate dispersal direction.

Clades	Median (Myr)	Upper (Myr)	Lower (Myr)	Events
Gekkonidae	15.42	22.4	9.49	South Asia → The Himalaya
Gekkonidae	14.98	20.55	10.19	Southeast Asia → The Himalaya
Dicroglossidae	19.4	27.58	11.79	South Asia → The Himalaya
Dicroglossidae	6.37	9.83	3.82	South Asia → The Himalaya
Dicroglossidae	18.11	27.84	10.43	South Asia → The Himalaya
Bufonidae	13.38	20.38	7.56	Southeast Asia → The Himalaya
Bufonidae	16.69	24.28	9.23	South Asia → The Himalaya
Bufonidae	7.53	12.06	4.32	Europe → The Himalaya
Pareidae	51.32	66.28	37.72	Southeast Asia → The Himalaya
Megophryidae	25.73	29.68	21.2	Southeast Asia → The Himalaya
Megophryidae	22.6	26.81	18.17	Southeast Asia → The Himalaya
Megophryidae	1.12	2.56	0.35	East Asia → The Himalaya
Megophryidae	29.59	33.6	24.8	Southeast Asia → The Himalaya
Megophryidae	18.12	24.42	11.92	Southeast Asia → The Himalaya
Viperidae	15.37	20.32	10.91	Southeast Asia → The Himalaya
Viperidae	12.94	17.62	8.73	Southeast Asia → The Himalaya
Viperidae	17.27	21.87	13.07	Southeast Asia → The Himalaya
Viperidae	9.75	12.41	7.49	Southeast Asia → The Himalaya
Viperidae	12.65	16.81	9.1	Southeast Asia → The Himalaya
Viperidae	22.68	26.94	18.74	Southeast Asia → The Himalaya
Agamidae	51.25	59.79	40.79	South Asia → The Himalaya
Agamidae	16.34	23.39	10.3	Southeast Asia → The Himalaya
Agamidae	12.87	17.36	9.18	South Asia → The Himalaya
Agamidae	54.67	62.68	46.62	Southeast Asia → The Himalaya
Agamidae	38.66	52.33	26.49	Southeast Asia → The Himalaya
Agamidae	9.04	12.97	5.9	East Asia → The Himalaya
Agamidae	55.25	64.82	43.6	Central-West Asia → The Himalaya
Anguidae	27.97	37.27	19.92	Southeast Asia → The Himalaya
Scincidae	40.35	48.48	31.3	Central-West Asia → The Himalaya
Scincidae	19.81	26.46	13.57	South Asia → The Himalaya

Scincidae	30.49	36.42	24.97	Southeast Asia → The Himalaya
Scincidae	26.62	33.98	18.79	Southeast Asia → The Himalaya
Scincidae	25.32	32.66	18.61	Southeast Asia → The Himalaya
Rhacophoridae	5.48	9.36	2.64	Southeast Asia → The Himalaya
Rhacophoridae	41.45	46.72	35.66	Southeast Asia → The Himalaya
Rhacophoridae	12.99	17.29	8.8	Southeast Asia → The Himalaya
Rhacophoridae	9.21	14.69	5.01	Southeast Asia → The Himalaya
Rhacophoridae	23.34	29.64	17.42	Southeast Asia → The Himalaya
Rhacophoridae	2.89	5.34	1.05	Southeast Asia → The Himalaya
Rhacophoridae	5.02	7.27	3.25	Southeast Asia → The Himalaya
Rhacophoridae	14.4	19.98	9.14	Southeast Asia → The Himalaya
Rhacophoridae	43.42	49.03	36.74	Southeast Asia → The Himalaya
Rhacophoridae	11.98	15.9	8.33	Southeast Asia → The Himalaya
Rhacophoridae	15.79	21.03	10.33	Southeast Asia → The Himalaya
Ranidae	27.57	32.88	21.7	Southeast Asia → The Himalaya
Ranidae	36.76	41.27	32.36	Southeast Asia → The Himalaya
Ranidae	7.51	11.42	4.01	Southeast Asia → The Himalaya
Ranidae	33.4	38.58	28.32	Southeast Asia → The Himalaya
Ranidae	21.42	26.82	16.62	Southeast Asia → The Himalaya
Elapidae	12.78	18.31	8.32	Southeast Asia → The Himalaya
Elapidae	9.81	19.88	3.47	Southeast Asia → The Himalaya
Colubridae	7.91	12.59	4.38	Southeast Asia → The Himalaya
Colubridae	9.78	14.49	5.8	Southeast Asia → The Himalaya
Colubridae	19.02	24.32	14.01	Southeast Asia → The Himalaya
Colubridae	14.19	18.78	9.99	Southeast Asia → The Himalaya
Colubridae	5.54	9.57	2.71	Southeast Asia → The Himalaya
Colubridae	4.17	7.05	2.26	Southeast Asia → The Himalaya
Colubridae	9.29	14.7	5.29	Southeast Asia → The Himalaya
Colubridae	5.64	8.69	3.32	Southeast Asia → The Himalaya
Colubridae	17.98	22.69	13.59	Southeast Asia → The Himalaya
Colubridae	23.49	28.77	18.64	Southeast Asia → The Himalaya
Colubridae	38.81	44.8	32.94	Southeast Asia → The Himalaya

Table S3.

PCR details used in this study.

Clades	Gene	Primer name	Primer sequence	Annealing temperature	Source
Amolops	16S rRNA	16SAR	CGCCTGTTTAYCAAAAACAT	55℃	[33]
		16SBR	CCGGTYTGAACTCAGATCAYGT		[33]
	ND2	Met-LND2	CAATGTTGGTTAAAATCCTTCC	49℃	[34]
		Trp-HND2	AGGCTTTGAAGGCCTTTGGTC		[34]
	Tyr	Tyr1G	TGCTGGGCRTCTCTCCARTCCCA	50℃	[35]
		Tyr1B	AGGTCCTCYTRAGGAAGGAATG		[35]
		Tyr1D	TCCTCCGTGGGCACCCARTTCCC		[35]
		Tyr1F	TCATCTCCCGYCAYCTTCTGGAT		[35]
	RAG1	L-RAG1Ran	CTGGTCGTCAGATCTTTTCAGC	50℃	[36]
		H-RAG1Ran	GCAAAACGTTGAGAGTGATAAC		[36]
		L-RAG1Ranint	GGAAATTGGTGGAATCCTCAG		[36]
		H-RAG1Ranint	ATATAGATAGAGCCTGAGGC		[36]
Feihyla	16S rRNA	16SAR	CGCCTGTTTAYCAAAAACAT	55℃	[33]
		16SBR	CCGGTYTGAACT		[33]

			CAGATCAYGT		
Gracixalus	16S rRNA	16SAR	CGCCTGTTTAYCAAAAACAT	55℃	[33]
		16SBR	CCGGTYTGAACTCAGATCAYGT		[33]
Kurixalus	12–16S rRNA	L2519	AAACTGGATTAGATACCCCACTAT	55℃	[33]
		16SBR	CCGGTYTGAACTCAGATCAYGT		[33]
		H3296	GCTAGACCATKATGCAAAAGGTA		[33]
		16SAR	CGCCTGTTTAYCAAAAACAT		[33]
		12/16S-696F	TATARCAATAGTACCGCAAG		**This study**
		12/16S-1563R	CGGTTAGGATACCGCGGCCGT		**This study**
Polypedates	12–16S rRNA	L2519	AAACTGGATTAGATACCCCACTAT	55℃	[33]
		16SBR	CCGGTYTGAACTCAGATCAYGT		[33]
		H3296	GCTAGACCATKATGCAAAAGGTA		[33]
		16SAR	CGCCTGTTTAYCAAAAACAT		[33]
		12/16S-696F	TATARCAATAGTACCGCAAG		**This study**
		12/16S-1563R	CGGTTAGGATAC		**This study**

			CGCGGCCGT		
Theloderma	12–16S rRNA	L2519	AAACTGGGATTAGATACCCCACTAT	55℃	[33]
		16SBR	CCGGTYTGAACTCAGATCAYGT		[33]
		H3296	GCTAGACCATKATGCAAAAGGTA		[33]
		16SAR	CGCCTGTTTAYCAAAAACAT		[33]
		12/16S-696F	TATARCAATAGTACCGCAAG		**This study**
		12/16S-1563R	CGGTTAGGATACCGCGGCCGT		**This study**
Raorchestes	16S rRNA	16SAR	CGCCTGTTTAYCAAAAACAT	55℃	[33]
		16SBR	CCGGTYTGAACTCAGATCAYGT		[33]
	ND1	ND1-F	TCTGGRTGAGCATCAAACTCT	50℃	**This study**
		ND1-R	TATCTTTCGGGCACATTTCCAT		**This study**
Rhacophorus	12–16S rRNA	L2519	AAACTGGGATTAGATACCCCACTAT	55℃	[33]
		16SBR	CCGGTYTGAACTCAGATCAYGT		[33]
		H3296	GCTAGACCATKATGCAAAAGGTA		[33]
		16SAR	CGCCTGTTTAYC		[33]

		12/16S-696F	AAAAACATTATARCAATAGTACCGCAAG		**This study**
		12/16S-1563R	CGGTTAGGATACCGCGGCCGT		**This study**
		L-RAG1Ran	CTGGTCGTCAGATCTTTCAGC	50℃	[36]
		H-RAG1Ran	GCAAAACGTTGAGAGTGATAAC		[36]
	RAG1	L-RAG1Ranint	GGAAATTGGTGGAATCCTCAG		[36]
		H-RAG1Ranint	ATATAGATAGAGCCTGAGGC		[36]
		CJ-L001	GACCTACTGTCACCAA	55℃	**This study**
	RAG2	CJ-H004	CTGGTTTGTTGAGTTCTGTGA		**This study**
Nanorana		CJ-L002	CATACCCTTGAAAGTAACA		**This study**
		CJ-H003	AYCACCCATATYRCTACCAAACC		**This study**
	Rod	Rhod1A	ACCATGAACGGAACAGAAGYCC	50℃	[35]
		Rhod1D	GTAGCGAAGAARCCTTCAAMGTA		[35]
		Tyr1G	TGCTGGGCRTCTCTCCARTCCCA	50℃	[35]
	Tyr	Tyr1B	AGGTCCTCYTRAGGAAGGAATG		[35]
		Tyr1D	TCCTCCGTGGGC		[35]

			ACCCARTTCCC		
		Tyr1F	TCATCTCCCGYC AYCTTCTGGAT		[35]
	Rod	Rhod1A	ACCATGAACGGA ACAGAAGGYCC	50℃	[35]
		Rhod1D	TAGCGAAGAARC CTTCAAMGTA		[35]
	Tyr	Tyr1G	TGCTGGGCRTCT CTCCARTCCCA	50℃	[35]
		Tyr1B	AGGTCCTCYTRA GGAAGGAATG		[35]
Liurana		Tyr1D	TCCTCCGTGGGC ACCCARTTCCC		[35]
		Tyr1F	TCATCTCCCGYC AYCTTCTGGAT		[35]
	RAG1	L-RAG1Ran	CTGGTCGTCAGA TCTTTCAGC	50℃	[36]
		H-RAG1Ran	GCAAAACGTTGA GAGTGATAAC		[36]
		L-RAG1Ranint	GGAAATTGGTGG AATCCTCAG		[36]
		H-RAG1Ranint	ATATAGATAGAG CCTGAGGC		[36]
Bufotes	D-loop	CytbA-L	GAATYGGRGGW CAACCAGTAGAA GACCC	55℃	[37]
		ControlB-H	GTCCATTGGAGG TTAAGATCTACC A		[37]
	ND1–	L3878	GCCCCATTTGAC	50℃	[38]

	ND2	H4980	CTCACAGAAGG<br>ATTTTTCGTAGT<br>TGGGTTTGRTT		[39]
		L4437	AAGCTTTCGGGC<br>CCATACC		[39]
		L4221	AAGGACCTCCTT<br>GATAGGGA		[38]
		H4419	GGTATGGGCCCA<br>AAAGCTT		[38]
		L2519	AAACTGGGATTA<br>GATACCCCACTA<br>T	55℃	[33]
		16SBR	CCGGTYTGAACT<br>CAGATCAYGT		[33]
Duttaphrynus	12–<br>16S<br>rRNA	H3296	GCTAGACCATKA<br>TGCAAAAGGTA		[33]
		16SAR	CGCCTGTTTAYC<br>AAAAACAT		[33]
		12/16S-696F	TATARCAATAGT<br>ACCGCAAG		**This study**
		12/16S-1563R	CGGTTAGGATAC<br>CGCGGCCGT		**This study**
	COI	Chmf4	TYTCWACWAAY<br>CAYAAAGAYAT<br>CGG	46℃	[40]
Scutiger		Chmr4	ACYTCRGGRTGR<br>CCRAARAATCA		[40]
	Cytb	L141841	CTTCCATCCAAC<br>ATCTCAGCATGA<br>TGAAA	50℃	[41]

		SCU5	ACAAGACCAATGCTTTAGTTAAGCTAC		[41]
		L2519	AAACTGGGATTAGATACCCCACTAT	55℃	[33]
		16SBR	CCGGTYTGAACTCAGATCAYGT		[33]
Leptobrachium	12–16S rRNA	H3296	GCTAGACCATKATGCAAAAGGTA		[33]
		16SAR	CGCCTGTTTAYCAAAAACAT		[33]
		12/16S-696F	TATARCAATAGTACCGCAAG		**This study**
		12/16S-1563R	CGGTTAGGATACCGCGGCCGT		**This study**
		HERP691	CAGAAGCGTGCCCGAAYA		**This study**
Calotes	ND1–ND2	HERP692	CCTCTGGATTGGGYGTTC	54℃	**This study**
		HERP693	AATGCCTGAYTTACYGGA		**This study**
		HERP694	CTGGAAGTCAGAAGTGGG		**This study**
		PTF	AGGACTYGAACCTCYACA		**This study**
Japalura sensu lato (Ptyctolaemus, Diploderma, Japalura sensu stricto, Mictopholis, Pseudocalotes)	ND1–ND2	PTR	GGTTGTGKCGTCATTTTA	54℃	**This study**
		PTNF	TAACSGGTARCTGAGCCA		**This study**

		PTNR	TTTGATTTATTCCGCCYC		**This study**
	12S rRNA	L2519	AAACTGGGATTAGATACCCCACTAT	55℃	[33]
		H3296	GCTAGACCATKATGCAAAGGTA		[33]
	16S rRNA	16SAR	CGCCTGTTTAYCAAAAACAT	55℃	[33]
		16SBR	CCGGTYTGAACTCAGATCAYGT		[33]
Naja	Cytb	L14910	GACCTGTGATMTGAAAACCAYCGTTGT	55℃	[42]
		H16064	CTTTGGTTTACAAGAACAATGCTTTA		[42]
	ND4	ND4	CACCTATGACTACCAAAAGCTCATGTAGAAGC	55℃	[43]
		LEU	ACCACGTTTAGGTTCATTTTCATTAC		[43]
Dopasia	COI	Chmf4	TYTCWACWAAYCAYAAAGAYATCGG	46℃	[40]
		Chmr4	ACYTCRGGRTGRCCRAARAATCA		[40]
Pareas	Cytb	L14910	GACCTGTGATMTGAAAACCAYCGT	55℃	[42]

			TGT		
		H16064	CTTTGGTTTACAAGAACAATGCTTTA		[42]
	C-mos	S77	CATGGACTGGATCAGTTATG	55℃	[44]
		S78	CCTTGGGTGTGATTTTCTCACCT		[44]
		Trpr3a	TTTAGGGCTTTGAAGGC		[45]
Asymblepharus	ND1−ND2	LJXND1-ND2R	AGTAGYAGGYGRCAGGTTGT	54℃	**This study**
		LJXND1-ND2F	AACTTCCTCCCWCTMACCYT		**This study**
		H4980	ATTTTTCGTAGTTGGGTTTGRTT		[39]
		L4437	AAGCTTTCGGGCCCATACC		[39]
	ND2	L4437 *	AAGCTTTCGGGCCCATACC	50℃	[39]
		SphenoR	TAGGYGGCAGGTTGTAGCCC		[46]
Sphenomorphus& *Scincella*		ND2sphR	CTCTTDTTTGTRGCTTTGAAGGC		[46]
	12S rRNA	12S.H1478	GAGGGTGACGGGCGGTGTGT	50℃	[33]
		12S.L1091	AAACTGGGATTAGATACCCCACTAT		[33]
	16S	16SF.SKINK	TGTTTACCAAAA	50℃	[47]

	rRNA		ACATAGCCTTTAGC		
		16SR.SKINK	TAGATAGAAACCGACCTGGATT		[47]
Altiphylax	ND2	HERP654	AAGCAGTTGGGCCCATACC	49℃	**This study**
		HERP665	AGRGTGCCAATGTCTTTGTGRTT		**This study**
	RAG1	Rag1yff	AAYAATGATAAYGGGGAAAT	56℃	**This study**
		Rag1yfr	ACAGCCAAAGTRTTGAGTAT		**This study**
	PDC	PHOF2	AGATGAGCATGCAGGAGTATGA	55℃	[48]
		PHOR1	TCCACATCCACAGCAAAAAACTCCT		[48]
Cyrtodactylus	ND2	HERP654	AAGCAGTTGGGCCCATACC	49℃	**This study**
		HERP665	AGRGTGCCAATGTCTTTGTGRTT		**This study**
	RAG1	Rag1yff	AAYAATGATAAYGGGGAAAT	56°	**This study**
		Rag1yfr	ACAGCCAAAGTRTTGAGTAT		**This study**
	PDC	PHOF2	AGATGAGCATGCAGGAGTATGA	55°	[48]
		PHOR1	TCCACATCCACAGCAAAAAACTCCT		[48]

Hemidactylus	PDC	PHOF2	AGATGAGCATGCAGGAGTATGA	55℃	[48]
		PHOR1	TCCACATCCACAGCAAAAAACTCCT		[48]
	RAG1	Rag1yff	AAYAATGATAAYGGGGAAAT	56℃	**This study**
		Rag1yfr	ACAGCCAAAGTRTTGAGTAT		**This study**
Hemiphyllodactylus	ND2	HERP654	AAGCAGTTGGGCCCATACC	49℃	**This study**
		HERP665	AGRGTGCCAATGTCTTTGTGRTT		**This study**
Ovophis	12S rRNA	L2519	AAACTGGGATTAGATACCCCACTAT	55℃	[33]
		H3296	GCTAGACCATKATGCAAAAGGTA		[33]
	16S rRNA	16SAR	CGCCTGTTTAYCAAAAACAT	55℃	[33]
		16SBR	CCGGTYTGAACTCAGATCAYGT		[33]
	Cytb	L14910	GACCTGTGATMTGAAAACCAYCGTTGT	55℃	[42]
		H16064	CTTTGGTTTACAAGAACAATGCTTTA		[42]
	ND4	ND4	CACCTATGACTA	55℃	[43]

		Leu	CCAAAAGCTCATGTAGAAGCCATTACTTTTACTTGGATTTGCACCA		[43]
	12S rRNA	L2519	AAACTGGGATTAGATACCCCACTAT	55℃	[33]
		H3296	GCTAGACCATKATGCAAAAGGTA		[33]
	16S rRNA	16SAR	CGCCTGTTTAYCAAAAACAT	55℃	[33]
		16SBR	CCGGTYTGAACTCAGATCAYGT		[33]
Protobothrops	Cytb	L14910	GACCTGTGATMTGAAAACCAYCGTTGT	55℃	[42]
		H16064	CTTTGGTTTACAAGAACAATGCTTTA		[42]
	C-mos	S77	CATGGACTGGGATCAGTTATG	55℃	[44]
		S78	CCTTGGGTGTGATTTTCTCACCT		[44]
Trimeresurus	12S rRNA	L2519	AAACTGGGATTAGATACCCCACTAT	55℃	[33]
		H3296	GCTAGACCATKATGCAAAAGGTA		[33]
	16S	16SAR	CGCCTGTTTAYC	55℃	[33]

rRNA		AAAAACAT		
	16SBR	CCGGTYTGAACT CAGATCAYGT		[33]
Cytb	L14910	GACCTGTGATMT GAAAACCAYCGT TGT	55℃	[42]
	H16064	CTTTGGTTTACA AGAACAATGCTT TA		[42]

Table S4.

Gene partition models inferred by IQTREE.

Family	Gene	Nucleotide substitution model
Agamidae	12s−16s	TIM2+F+I+G4
	BDNF	TIM3e+I+G4
	C-mos	K2P+G4
	R35	TIM3+F+G4
	Rag1	TIM+F+I+G4
	Cytb	GTR+F+I+G4
	ND1−ND2	GTR+F+I+G4
	ND4	GTR+F+I+G4
Anguidae	*AKAP9*	HKY+F
	ATPSb	HKY+F
	BACH1	HKY+F
	COI	TPM2u+F+I+G4
	EXPH5	TIM2+F
	NKTR	HKY+F
	SELT	HKY+F
	UBN1	TN+F+G4
	Cytb	TPM2u+F+I+G4
	ND2	TVM+F+I+G4
	ND4	TPM2u+F+I+G4
Bufonidae	12s−16s	GTR+F+I+G4
	CXCR4	TPM3u+F+I+G4
	NXC1	TIM3+F+I+G4
	POMC	TIM2+F+I+G4
	Rag1	TIM2+F+I+G4
	ND2	K2P+G4

Ceratobatrachidae	12s−16s	GTR+F+I+G4
	POMC	TN+F+G4
	Rag1	HKY+F+G4
	Tyr	TPM3u+F+I+G4
Colubridae	12S	TIM2+F+I+G4
	C-mos	K3Pu+F+G4
	Cytb	GTR+F+I+G4
	ND2	GTR+F+I+G4
	ND4	GTR+F+I+G4
Dicroglossidae	12s−16s	GTR+F+I+G4
	Rag1	HKY+F+I+G4
	Rag2	TIM3+F+I+G4
	Rhod	K2P+I+G4
	Tyr	TIM2e+I+G4
Elapidae	12s−16s	GTR+F+I+G4
	C-mos	TPM3u+F+G4
	Cytb	GTR+F+I+G4
	ND4	GTR+F+I+G4
	Rag1	TPM3u+F+G4
Geckkonidae	*ND2*	K3Pu+F+I+G4
	Rag1	TN+F+G4
	PDC	TIM2e+G4
Megophryidae	16s	GTR+F+I+G4
	BDNF	K2P+G4
	COI	TVM+F+I+G4
	Rag1	TIMe+G4
	Rhod	K2P+G4
Pareidae	*C-mos*	K2P+G4
	Cytb	GTR+F+I+G4

	ND4	GTR+F+I+G4
	12s−16s	GTR+F+I+G4
	BDNF	K2P+I
	CMYC	K3P+I
	COI	TPM2+F+I+G4
	CXCR4	TPM2u+F+I+G4
	Cytb	TPM2+F+I+G4
	NCX1	HKY+F+I
Ranidae	*ND2*	GTR+F+I+G4
	ND3	TIM2+F+I+G4
	POMC	HKY+F+G4
	Rag1	TN+F+G4
	Rag2	HKY+F+G4
	RH1	K2P+I
	Rhod	K2P+I+G4
	SLC8A3	HKY+F+I
	Tyr	TIM2e+I+G4
	12s−16s	GTR+F+I+G4
	BDNF	TIM2e+I+G4
	COI	TIM2+F+I+G4
	ND1	TPM2+F+I+G4
Rhacophoridae	*POMC*	TN+F+G4
	Rag1	TIM3+F+I+G4
	Rhod	TVM+F+I+G4
	Tyr	TIM2e+I+G4
	Cytb	TPM2u+F+I+G4
	12s−16s	GTR+F+I+G4
Scincidae	*BDNF*	K3P+I+G4
	C-mos	TPM2u+F+I+G4

	Cytb	GTR+F+I+G4
	ND2	GTR+F+I+G4
	ND4	GTR+F+I+G4
	R35	TVM+F+G4
	Rag1	GTR+F+G4
	12s−16s	TIM2+F+I+G4
	Cytb	GTR+F+I+G4
	ND4	GTR+F+I+G4
	BDNF	TIM2e+I+G4
Viperidae	*C-mos*	TPM3u+F+G4
	jun	HKY+F+G4
	nt3	GTR+F+I+G4
	Rag1	TPM3+F+G4

Table S5.

The details of each calibration used in this study. The calibration nodes numbers correspond with **Data S6**.

Calibration nodes no.	Description	Type	References
1	Second calibration for Natatanura from 59.1 Ma to 69.8 Ma	Second calibration	[14]
2	Stem of *Pelophylax* at least 32 Ma	fossil	[49]
3	Stem of *Rana temporaria* group at least 19 Ma	fossil	[50]
4	Stem of *Rana catesbeiana* group at least 15 Ma	fossil	[51]
5	Stem of *Rana pipiens* group at least 18 Ma	fossil	[52, 53]
6	Stem-Alethinophidia, 95% set around 145 Ma	fossil	[54, 55, 56, 57]
7	Stem-Colubroidea, 95% set around 145 Ma	fossil	[54, 55, 56, 57]
8	Stem-Boinae, 95% set around 145 Ma	fossil	[54, 55, 56, 57]

9	Stem-Elapidae, 95% set around 145 Ma	fossil	[54, 55, 56, 57]
10	Stem-Viperidae, 95% set around 61.5 Ma	fossil	[56]
11	Stem-*Sistrurus*, 95% set around 47.4 Ma	fossil	[56]
12	Oldest '*Bufo*', 55 Ma (L. Palaeocene)	fossil	[57]
13	*Rhinella marinus* (M), Mie Miocene (11 Ma)	fossil	[58]
14	A minimum age of 18 Ma for the stem origin of toads belonging to the *Bufo viridis* group	fossil	[49, 59, 60]
15	A minimum age of 9.6 Ma for the origin of toads belonging to the *Bufo bufo* group	fossil	[49]
16	Second calibration for Megophryidae from 115.6 Ma to 141.5 Ma	Second calibration	[61]
17	MRCA for the *Spea*-*Scaphiopus* split at 33.3 Ma based on the oldest fossil scaphiopodid originally assigned with considerable confidence to the genus *Scaphiopus* (and not *Spea*), *S. skinneri* Estes, 1970, from the early Oligocene (Rupelian stage, 33.9−28.1 Ma, more specifically the Orellan, 33.9−33.3 Ma).	fossil	[62]
18	MRCA for the Pelodytidae-Pelobatidae split at 50.3 Ma based on the recently described, earliest known fossil pelodytid, *Aerugoamnis paulus* from the lower Eocene	fossil	[63]

	(Wasatchian,55.4−50.3 Ma) of the Green River formation,Wyoming, which was characterized based on a single almost complete, and mostly articulated specimen		
19	MRCA for the Pelobatidae-Megophryidae split at 46.2 Ma, based on the recently described oldest unequivocally assigned pelobatid species, *Eopelobates deani*, from the early middle Eocene (or more specifically the Bridgerian, 50.3−46.2 Ma) of the Green River Formation, Wyoming	fossil	[64]
20	The oldest known anguid, with the most complete fossil record of any lizard, is *Odaxosaurus*, from the late Campanian of Canada, approximately 75 Ma.	fossil	[65]
21	The earliest record of *Ophisaurus* in Europe is a parietal of *Ophisaurus* sp. from the late Eocene Newton Bone Bed (Osborne Member, ca. 35 Ma)	fossil	[66]
22	The fossil and associated fossil material were found in the Split Rock Formation in the Granite Mountains of central Wyoming. The minimum chronostratigraphic age of *Elgaria* is 16.7 Ma	fossil	[67]
23	root age: 123 Ma (124.5−121.4 Ma)	Second calibration	[68]
24	stem age of *Sceloporus*: at least 16 Ma (20.4−16 Ma)	fossil	[69]
25	Crown age of Phrynosomatinae (Phrynosoma, *Callisaurus*, Uma):at least 33.3 Ma	fossil	[70]

26	Crown-group age of Phrynosomatidae: at least 33.9 Ma	fossil	[70]
27	Crown age of *Anolis*: at least 15 Ma	fossil	[71]
28	Stem age of *Polychrus*: at least 33.9 Ma	fossil	[70]
29	Crown age of Iguanidae (*sensu stricto*): at least 16 Ma	fossil	[72, 73]
30	Crown age of Acrodonta(stem age of Chamaeleonidae): at least 48.6 Ma	fossil	[72, 73, 74]
31	Crown age of Agamidae (stem age of Uromastyx): at least 33.9 Ma	fossil	[73, 76]
32	Stem age of *Istiurus lesuerii*: at least 21 Ma	fossil	[73, 76]
33	Crown age Pleurodonta:at least 70 Ma	fossil	[77]
34	Crown age of Gekkotan at least 54 Ma	fossil	[78]
35	Stem of Scincidae at least 70 Ma	fossil	[80, 81]
36	Stem mrca of Xantusiidae, Gerrhosauridae and Cordylidae at least 65.2 Ma	fossil	[81]
37	Upper value of 95% interval of node of the mrca of Gekkota	Second calibration	[82]
38	Divergence between *Sphaerodactylus elegans* and *S. roosevelti* at 20−15Ma	fossil	[83]
39	The divergence between *Woodworthia maculata* and *Oedura marmorata* at 19−16Ma	fossil	[83]
40	The most-recent common ancestor of Gekkota at 110−97 Ma	fossil	[84]

Data S1.

Calibrated divergence time tree files analyzed by MCMCTREE of 14 families (in newick format).

Agamidae

(((((((((((((((((((Diploderma_brevipes:0.074229,Diploderma_luei:0.074229):0.029452,Diploderma_makii:0.103681):0.04265,Diploderma_polygonata:0.146331):0.03795,Diploderma_swinhonis:0.184281):0.043784,(Diploderma_flaviceps:0.157142,Diploderma_splendida:0.157142):0.070922):0.015732,((Diploderma_dymondi:0.110586,Diploderma_varcoae:0.110586):0.064689,(Diploderma_micangshanensis:0.13571,Diploderma_zhaoermii:0.13571):0.039565):0.068522):0.050489,Diploderma_iadinum:0.294286):0.027585,((((Diploderma_vela:0.081203,Diploderma_batangensis:0.081203):0.025091,Diploderma_yulongense:0.106294):0.140567,(Diploderma_laeviventris:0.170443,Diploderma_slowinskii:0.170443):0.076417):0.023941,(Diploderma_chapaensis:0.079921,Diploderma_yunnanense:0.079921):0.190879):0.051069):0.084967,(Pseudocalotes_bapoensis:0.254563,Pseudocalotes_brevipes:0.254563):0.152274):0.124952,(Acanthosaura_armata:0.264828,Acanthosaura_lepidogaster:0.264828):0.266961):0.03714,((Cristidorsa_planidorsata:0.13881,Cristidorsa_otai:0.13881):0.37372,Salea_horsfieldii:0.51253):0.056399):0.035155,(((((((Calotes_calotes:0.182358,Calotes_htunwini:0.182358):0.05367,Calotes_ceylonensis:0.236027):0.03484,(Calotes_versicolor:0.079723,Calotes_irawadi:0.079723):0.191144):0.066547,((Calotes_jerdoni:0.163372,Calotes_medogensis:0.163372):0.108575,Calotes_minor:0.271947):0.065467):0.047453,((Calotes_emma:0.026353,Calotes_chincollium:0.026353):0.315693,Calotes_mystaceus:0.342046):0.042821):0.140073,((((((Sitana_laticeps:0.074378,Sitana_spinaecephalus:0.074378):0.054275,Sitana_sivalensis:0.128653):0.035155,Sitana_marudhamneydhal:0.163808):0.021143,(Sitana_ponticeriana:0.0820570013473034,Sitana_visiri:0.082057):0.102895):0.087281,Sarada_deccanensis:0.272233):0.150218,Otocryptis_beddomei:0.422451):0.102489):0.047832,(((Aphaniotis_fusca:0.273888,Coryphophylax_subcristatus:0.273888):0.151658,(Bronchocela_cristatella:0.314953,Gonocephalus_grandis:0.314953):0.110593):0.07911,(Lyriocephalus_scutatus:0.327699,Ceratophora_aspera:0.327699):0.176957):0.068116):0.031312):0.070299,((((((((((Draco_obscurus:0.10198,Draco_taeniopterus:0.10198):0.085852,Draco_blanfordii:0.187832):0.025104,(Draco_haematopogon:0.167935,Draco_melanopogon:0.167935):0.045001):0.03119,Draco_quinquefasciatus:0.244125):0.020618,Draco_maximus:0.264744):0.053891,Draco_maculatus:0.318635):0.055314,(((Draco_cornutus:0.167187,Draco_volans:0.167187):0.095257,Draco_bimaculatus:0.262444):0.049308,Draco_biaro:0.311752):0.062197):0.036828,Draco_dussumieri:0.410777):0.025923,Draco_lineatus:0.4367):0.10996,(((Japalura_variegata:0.347727,Pseudocalotes_austeniana:0.347727):0.058576,Japalura_andersoniana:0.406303):0.03201,(Japalura_tricarinata:0.376687,Japalura_kumaonensis:0.376687):0.061625):0.108348):0.031404,(Ptyctolaemus_collicristatus:0.386566,Ptyctolaemus_gularis:0.386566):0.191498):0.096319):0.109088,Mantheyus_phuwuanensis:0.783472):0.106602,((((((((((((Phrynocephalus_raddei:0.083081,Phrynocephalus_reticulatus:0.083081):0.07153,Phrynocephalus_rossikowi:0.154611):0.024651,Phrynocephalus_strauchi:0.179262):0.045876,(Phrynocephalus_helioscopus:0.161231,Phrynocephalus_persicus:0.161231):0.063907):0.015151,((((Phrynocephalus_przewalskii:0.042475,Phrynocephalus_frontalis:0.042475):0.020857,Phrynocephalus_versicolor:0.063332):0.060514,Phrynocephalus_guttatus:0.123846):0.07904,Phrynocephalus_axillaris:0.202886):0.037404):0.023118,Phrynocephalus_mystaceus:0.263407):0.021181,((((Phrynocephalus_vlangalii:0.042869,Phrynocephalus_erythrurus:0.042869):0.047565,Phrynocephalus_theobaldi:0.090434):0.027167,(Phrynocephalus_putjatia:0.036538,Phrynocephalus_guinanensis:0.036538):0.081063):0.021183,Phrynocephalus_forsythii:0.138785):0.145803):0.043853,(((Phrynocephalus_arabicus:0.154113,Phrynocephalus_maculatus:0.154113):0.103894,Phrynocephalus_scutellatus:0.258007):0.034803,(Phrynocephalus_interscapularis:0.212901,Phrynocephalus_ornatus:0.212901):0.07991):0.035

63):0.194001,Stellagama_stellio:0.522441):0.034133,((Paralaudakia_himalayana:0.204435,Paralaudakia_lehmanni:0.204435):0.061123,Paralaudakia_caucasia:0.265558):0.291016):0.046346,((((Acanthocercus_atricollis:0.17171,Xenagama_taylori:0.17171):0.176662,Pseudotrapelus_sinaitus:0.348373):0.156682,(Agama_agama:0.245285,Agama_anchietae:0.245285):0.25977):0.030672,((Trapelus_agilis:0.093841,Trapelus_mutabilis:0.093841):0.222546,Bufoniceps_laungwalaensis:0.316387):0.21934):0.067193):0.046354,(((Laudakia_tuberculata:0.220084,Laudakia_papenfussi:0.220084):0.247137,(Laudakia_sacra:0.073726,Laudakia_wui:0.073726):0.393494):0.085232,(Laudakia_nupta:0.448327,Laudakia_melanura:0.448327):0.104125):0.096822):0.240799):0.048057,Hydrosaurus_amboinensis:0.938131):0.026518,((((((((((Tympanocryptis_tetraporophora:0.127828,Tympanocryptis_lineata:0.127828):0.066744,Pogona_vitticeps:0.194572):0.020892,(Diporiphora_nobbi:0.158108,Diporiphora_winneckei:0.158108):0.057355):0.020825,Rankinia_diemensis:0.236289):0.020718,Gowidon_temporalis:0.257007):0.018083,(Chlamydosaurus_kingii:0.165121,Amphibolurus_muricatus:0.165121):0.109968):0.04146,(Ctenophorus_adelaidensis:0.247188,Ctenophorus_isolepis:0.247188):0.069362):0.037741,Intellagama_lesueurii:0.354291):0.021452,((Chelosania_brunnea:0.279199,Moloch_horridus:0.279199):0.059767,Hypsilurus_boydii:0.338966):0.036777):0.123379,Physignathus_cocincinus:0.499122):0.465527):0.085557,(Leiolepis_belliana:0.259154,Leiolepis_guttata:0.259154):0.791052):0.049546,((Uromastyx_acanthinura:0.251193,Uromastyx_aegyptia:0.251193):0.182597,Saara_hardwickii:0.433791):0.66596);

Anguidae

(((((((Dopasia_hainanensis:0.15496,Dopasia_sp:0.15496):0.034935,Dopasia_harti:0.189895):0.089765,Dopasia_gracilis:0.27966):0.091846,(Ophisaurus_ventralis:0.289145,Ophisaurus_attenuatus:0.289145):0.082361):0.044481,(Anguis_fragilis:0.27204,Pseudopus_apodus:0.27204):0.143948):0.041828,Hyalosaurus_koellikeri:0.457815):0.179639,((((Abronia_graminea:0.293464,Mesaspis_monticola:0.293464):0.032031,Barisia_imbricata:0.325494):0.035709,Gerrhonotus_parvus:0.361203):0.062683,Elgaria_kingii:0.423886):0.213568);

Bufonidae

(((((((((((((((((((Bufo_aspinius:0.034532,Bufo_cryptotympanicus:0.034532):0.026912,Bufo_tuberospinius:0.061444):0.042135,Bufo_tuberculatus:0.103579002618313):0.021686,((Bufo_japonicus:0.080281,Bufo_torrenticola:0.080281):0.021912,Bufo_stejnegeri:0.102193):0.023072):0.015181,(Bufo_bankorensis:0.026894,Bufo_gargarizans:0.026894):0.113552):0.066838,(Bufo_bufo:0.022319,Bufo_verrucosissimus:0.022319):0.184965):0.087301,((Barbarophryne_brongersmai:0.252178,Epidalea_calamita:0.252178):0.028778,Sabahphrynus_maculatus:0.280956):0.01363):0.014149,(((((Ingerophrynus_biporcatus:0.134079,Ingerophrynus_parvus:0.134079):0.022972,Ingerophrynus_philippinicus:0.157052):0.019457,Ingerophrynus_divergens:0.176509):0.027184,(Ingerophrynus_galeatus:0.133799,Ingerophrynus_macrotis:0.133799):0.069894):0.085938,(Didynamipus_sjostedti:0.261679,Schismaderma_carens:0.261679):0.027952):0.019103):0.00998,(((((((((((Duttaphrynus_brevirostris:0.067146,Duttaphrynus_melanostictus:0.067146):0.015038,Duttaphrynus_parietalis:0.082185):0.048002,(Duttaphrynus_atukoralei:0.048006,Duttaphrynus_scaber:0.048006):0.08218):0.01228,Duttaphrynus_himalayanus:0.142466):0.028046,(Duttaphrynus_crocus:0.148453,Duttaphrynus_stuarti:0.148453):0.022059):0.038558,((Duttaphrynus_hololius:0.086715,Duttaphrynus_stomaticus:0.086715):0.033945,Duttaphrynus_dhufarensis:0.12066):0.08841):0.022401,(Bufoides_meghalayanus:0.166909,Xanthophryne_koynayensis:0.166909):0.064562):0.009916,Adenomus

_kelaartii:0.241387):0.022926,Pedostibes_tuberculosus:0.264313):0.036941,((Phrynoidis_asper:0.123844,Phrynoidis_juxtasper:0.123844):0.113195,Pedostibes_hosii:0.237039):0.064215):0.007345,Leptophryne_borbonica:0.308599):0.010116):0.00339,(((Ansonia_hanitschi:0.216217,Ansonia_leptopus:0.216217):0.051805,(Pelophryne_misera:0.151099,Pelophryne_signata:0.151099):0.116923):0.042513,(Ansonia_ornata:0.281423,Strauchbufo_raddei:0.281423):0.029112):0.011569):0.002563,(((((Bufotes_baturae:0.0174239997397661,Bufotes_zamdaensis:0.017424):0.05791,(Bufotes_boulengeri:0.029109,Bufotes_siculus:0.029109):0.046225):0.014583,Bufotes_balearicus:0.089917):0.037274,((Bufotes_oblongus:0.015243,Bufotes_pewzowi:0.015243):0.060226,Bufotes_variabilis:0.075468):0.051722):0.04714,Bufotes_viridis:0.174331):0.150336):0.00496,((Nectophrynoides_tornieri:0.111337,Nectophrynoides_viviparus:0.111337):0.105278,Churamiti_maridadi:0.216614):0.113013):0.007554,(((((Mertensophryne_micranotis:0.166108,Mertensophryne_uzunguensis:0.166108):0.054421,Poyntonophrynus_dombensis:0.220529):0.067291,(Sclerophrys_brauni:0.18704,Sclerophrys_garmani:0.18704):0.10078):0.022984,((Capensibufo_rosei:0.102099,Capensibufo_tradouwi:0.102099):0.157731,Vandijkophrynus_amatolicus:0.25983):0.050974):0.017133,((Nectophryne_afra:0.240898,Werneria_mertensiana:0.240898):0.035759,Wolterstorffina_parvipalmata:0.276657):0.05128):0.009244):0.023192,((((Anaxyrus_americanus:0.032126,Anaxyrus_terrestris:0.032126):0.134079,Anaxyrus_quercicus:0.166204):0.139675,((Incilius_alvarius:0.239402,Incilius_coccifer:0.239402):0.038774,Bufotes_latastii:0.278176):0.027703):0.032174,((Rhinella_marina:0.025028,Rhinella_schneideri:0.025028):0.232684,Rhinella_nattereri:0.257712):0.080341):0.02232):0.030105,(Peltophryne_lemur:0.32656,Rhaebo_haematiticus:0.32656):0.063918):0.037936,(Nannophryne_apolobambica:0.301935,Nannophryne_cophotis:0.301935):0.126478):0.053582,Dendrophryniscus_minutus:0.481996):0.049585,((Atelopus_peruensis:0.25548,Atelopus_spumarius:0.25548):0.203261,Osornophryne_guacamayo:0.458741):0.072839):0.070374,(Melanophryniscus_klappenbachi:0.071871,Melanophryniscus_stelzneri:0.071871):0.530082);

Ceratobatrachidae

((((((((((Platymantis_indeprensus:0.029158,Platymantis_mimulus:0.029158):0.033786,Platymantis_naomii:0.062944):0.019221,Platymantis_pseudodorsalis:0.082165):0.015645,(Platymantis_cagayanensis:0.019479,Platymantis_taylori:0.019479):0.078332):0.015872,(Platymantis_dorsalis:0.085823,Platymantis_paengi:0.085823):0.02786):0.032329,Platymantis_spelaeus:0.146012):0.039644,((((Platymantis_luzonensis:0.030234,Platymantis_negrosensis:0.030234):0.044574,Platymantis_rabori:0.074808):0.017032,(Platymantis_bayani:0.061353,Platymantis_guentheri:0.061353):0.030488):0.025539,Platymantis_diesmosi:0.11738):0.068276):0.074103,(((((((Platymantis_hazelae:0.017886,Platymantis_panayensis:0.017886):0.045217,Platymantis_lawtoni:0.063103):0.011775,(Platymantis_polillensis:0.027232,Platymantis_sierramadrensis:0.027232):0.047647):0.008003,Platymantis_subterrestris:0.082882):0.016354,Platymantis_isarog:0.099236):0.09965,Platymantis_corrugatus:0.198886):0.043755,(((Platymantis_banahao:0.085272,Platymantis_insulatus:0.085272):0.027618,Platymantis_pygmaeus:0.11289):0.030219,Platymantis_levigatus:0.143109):0.099532):0.017119):0.095506,(((((((((Cornufer_occidentalis:0.034194,Cornufer_pelewensis:0.034194):0.021478,Cornufer_papuensis:0.055672):0.05877,(Cornufer_boulengeri:0.051724,Cornufer_gilliardi:0.051724):0.062718):0.023316,(Cornufer_adiastolus:0.099893,Cornufer_akarithymus:0.099893):0.037865):0.017078,Cornufer_weberi:0.154836):0.039403,Cornufer_solomonis:0.194239):0.032265,Cornufer_parilis:0.226504):0.025935,(((Cornufer_bufonulus:0.108669,Cornufer_caesiops:0.108669):0.076067,Cornufer_minutus:0.184736):0.04114,((Cornufer_vertebralis:0.058268,Cornufer_

wolfi:0.058268):0.124282,Cornufer_cheesemanae:0.18255):0.043326):0.026563):0.025293,(((((Cornufer_guppyi:0.03209,Cornufer_hedigeri:0.03209):0.121532,Cornufer_vitianus:0.153622):0.036336,Cornufer_myersi:0.189957):0.043956,Cornufer_guentheri:0.233914):0.017138,(Cornufer_bufoniformis:0.097105,Cornufer_malukuna:0.097105):0.153947):0.026681):0.077534):0.075404,(((Alcalus_baluensis:0.132337,Alcalus_mariae:0.132337):0.084014,Alcalus_rajae:0.216351):0.095242,Alcalus_tasanae:0.311593):0.119077):0.024441,(((Liurana_xizangensis:0.032609,Liurana_medogensis:0.032609):0.105273,Liurana_alpina:0.137882):0.061158,Liurana_vallecula:0.19904):0.25607);

Colubridae

((Pseudoxenodon_stejnegeri:0.193649,(Pseudoxenodon_bambusicola:0.126207,(Pseudoxenodon_karlschmidti:0.105897,Pseudoxenodon_macrops:0.105897):0.02031):0.067442):0.329329,(((Hypsiglena_torquata:0.408453,(Thermophis_baileyi:0.057878,Thermophis_zhaoermii:0.057878):0.350575):0.076305,(Thamnophis_sirtalis:0.416029,(((Herpetoreas_platyceps:0.186996,(Herpetoreas_burbrinki:0.1333,Hebius_parallelum:0.1333):0.053696):0.056524,(Hebius_craspedogaster:0.230206,(Trachischium_monticola:0.190114,(Trachischium_guentheri:0.159252,Trachischium_tenuiceps:0.159252):0.030862):0.040092):0.013314):0.144621,(Amphiesma_stolatum:0.342912,(((Fowlea_piscator:0.19437,Fowlea_schnurrenbergeri:0.19437):0.040626,(Atretium_yunnanensis:0.144214,(Fowlea_flavipunctatus:0.104183,Xenochrophis_punctulatus:0.104183):0.040031):0.090782):0.084238,(Rhabdophis_himalayanus:0.234925,((Rhabdophis_subminiatus:0.17983,(Rhabdophis_adleri:0.15128,Rhabdophis_nigrocinctus:0.15128):0.02855):0.013393,(Rhabdophis_tigrinus:0.164031,(Rhabdophis_guangdongensis:0.099139,(Rhabdophis_pentasupralabialis:0.068931,(Rhabdophis_leonardi:0.056367,Rhabdophis_nuchalis:0.056367):0.012564):0.030208):0.064892):0.029192):0.041702):0.084309):0.023679):0.045229):0.027888):0.068729):0.02546,(((Calamaria_pavimentata:0.148077,Calamaria_yunnanensis:0.148077):0.282847,(Sibynophis_bistrigatus:0.342688,(Sibynophis_chinensis:0.181469,(Sibynophis_collaris:0.092891,Sibynophis_triangularis:0.092891):0.088578):0.161219):0.088235):0.05933,(((Ahaetulla_prasina:0.241556,(Ahaetulla_pulverulenta:0.219426,(Ahaetulla_fronticincta:0.16471,Ahaetulla_nasuta:0.16471):0.054716):0.02213):0.17217,((Chrysopelea_paradisi:0.294125,Chrysopelea_taprobanica:0.294125):0.083693,((Dendrelaphis_bifrenalis:0.27137,Dendrelaphis_subocularis:0.27137):0.045157,((Dendrelaphis_caudolineatus:0.077927,Dendrelaphis_fuliginosus:0.077927):0.20938,(Dendrelaphis_formosus:0.264324,((Dendrelaphis_striatus:0.166611,(Dendrelaphis_ngansonensis:0.041661,Dendrelaphis_cyanochloris:0.0416610002315044):0.124949):0.068927,(Dendrelaphis_haasi:0.139101,(Dendrelaphis_marenae:0.001273,Dendrelaphis_pictus:0.001273):0.137828):0.0964369996144772):0.028786):0.022983):0.029221):0.061291):0.035908):0.049444,(((Archelaphe_bella:0.317692,Oreocryptophis_porphyraceus:0.317692):0.038448,((Elaphe_hodgsoni:0.262531,(Elaphe_taeniura:0.244662,(Elaphe_moellendorffi:0.18103,Elaphe_cantoris:0.181030004778862):0.063632):0.01787):0.048248,((Elaphe_quatuorlineata:0.070572,Elaphe_sauromates:0.070572):0.151625,((Elaphe_bimaculata:0.13451,Elaphe_dione:0.13451):0.064013,(Elaphe_climacophora:0.184757,((Elaphe_carinata:0.068986,Elaphe_davidi:0.068986):0.100033,(Elaphe_quadrivirgata:0.1448,Elaphe_schrenckii:0.1448):0.024219):0.015738):0.013765):0.023675):0.088582):0.04536):0.047115,(((Ptyas_korros:0.297485,((Ptyas_carinata:0.211785,Ptyas_mucosa:0.211785):0.065746,((Ptyas_dhumnades:0.058366,Ptyas_nigromarginata:0.058366):0.195326,(Ptyas_fusca:0.178615,Ptyas_luzonensis:0.178615):0.075077):0.023839):0.019954):0.082962,(((Boiga_forsteni:0.149802,Boiga_cynodon:0.149802):0.167109,(Boiga_trigonata:0.

289911,(Boiga_ceylonensis:0.243756,(Boiga_barnesii:0.171844,Boiga_beddomei:0.171844):0.071912):0.046154):0.027001):0.036534,(Oligodon_sublineatus:0.283351,((((Oligodon_cruentatus:0.047791,Oligodon_theobaldi:0.047791):0.060435,(Oligodon_planiceps:0.055402,Oligodon_torquatus:0.055402):0.052823):0.076072,((Oligodon_cinereus:0.111973,(Oligodon_nagao:0.053061,Oligodon_sp:0.053061):0.058912):0.046082,(Oligodon_lipipengi:0.141933,(Oligodon_maculatus:0.083415,Oligodon_splendidus:0.083415):0.058517):0.016122):0.026243):0.053349,((Oligodon_ornatus:0.092199,Oligodon_lacroixi:0.092199):0.113684,((Oligodon_chinensis:0.041358,Oligodon_formosanus:0.041358):0.079932,(Oligodon_cyclurus:0.103513,(Oligodon_octolineatus:0.095134,(Oligodon_taeniatus:0.076277,(Oligodon_fasciolatus:0.060182,(Oligodon_barroni:0.026354,Oligodon_ocellatus:0.026354):0.033827):0.016096):0.018856):0.008379):0.017778):0.084593):0.031763):0.045705):0.070094):0.027002):0.009429,((Coelognathus_radiatus:0.309945,(Coelognathus_helena:0.27488,(Coelognathus_erythrurus:0.182002,(Coelognathus_flavolineatus:0.131576,Coelognathus_subradiatus:0.131576):0.050426):0.092878):0.035065):0.068828,(Coluber_constrictor:0.372251,(((Gonyosoma_jansenii:0.082514,Gonyosoma_oxycephalum:0.082514):0.232649,(Gonyosoma_prasinum:0.216138,(Gonyosoma_boulengeri:0.084256,Gonyosoma_frenatum:0.084256):0.131881):0.099025):0.045582,(Lycodon_paucifasciatus:0.330888,((Lycodon_carinatus:0.207808,Lycodon_laoensis:0.207808):0.108759,((Lycodon_ruhstrati:0.257378,(Lycodon_dumerilii:0.180582,(Lycodon_muelleri:0.148105,(Lycodon_bibonius:0.037841,(Lycodon_alcalai:0.005561,Lycodon_chrysoprateros:0.005561):0.032281):0.110264):0.032476):0.076796):0.045754,((Lycodon_effraensis:0.256686,(Lycodon_jara:0.190153,(Lycodon_aulicus:0.097792,Lycodon_zawi:0.097792):0.092361):0.066533):0.028707,((Lycodon_synaptor:0.187747,(Lycodon_gongshan:0.125325,((Lycodon_butleri:0.0824,Lycodon_cavernicolus:0.0824):0.030815,Lycodon_fasciatus:0.113214000617504):0.012111):0.062422):0.07142,((Lycodon_stormi:0.188749,Lycodon_subcinctus:0.188749):0.031287,((Lycodon_futsingensis:0.079124,Lycodon_septentrionalis:0.079124):0.0607,(Lycodon_rufozonatus:0.108353,Lycodon_meridionalis:0.108352999994278):0.031471):0.080211):0.039131):0.026225):0.01774):0.013435):0.01432):0.029857):0.011506):0.006523):0.011104):0.013377):0.059916):0.027083):0.019964):0.01276);

Dicroglossidae

((((((((((Fejervarya_multistriata:0.064752,Fejervarya_sakishimensis:0.064752):0.042469,Fejervarya_iskandari:0.107221):0.038855,Fejervarya_triora:0.146076):0.03003,Fejervarya_limnocharis:0.176106):0.062969,Fejervarya_cancrivora:0.239074):0.06576,(((Sphaerotheca_breviceps:0.019135,Sphaerotheca_pluvialis:0.019135):0.092758,Sphaerotheca_dobsonii:0.111893):0.082088,Sphaerotheca_rolandae:0.193981):0.110854):0.013196,(((Minervarya_pierrei:0.063672,Minervarya_syhadrensis:0.063672):0.057228,Minervarya_greenii:0.120901):0.032831,Minervarya_caperata:0.153731):0.164299):0.065577,((((((Hoplobatrachus_crassus:0.09473,Hoplobatrachus_tigerinus:0.09473):0.030359,Hoplobatrachus_rugulosus:0.125088):0.071106,Hoplobatrachus_occipitalis:0.196194):0.046022,Euphlyctis_cyanophlyctis:0.242216):0.044648,Euphlyctis_hexadactylus:0.286864):0.047213,Nannophrys_ceylonensis:0.334077):0.04953):0.09867,((((Limnonectes_fujianensis:0.118888,Limnonectes_kuhlii:0.118888):0.115431,Limnonectes_fragilis:0.234319):0.036974,(Limnonectes_deinodon:0.221556,Limnonectes_limborgi:0.221556):0.049737):0.047372,(((Limnonectes_finchi:0.155458,Limnonectes_poilani:0.155458):0.047416,Limnonectes_grunniens:0.202873):0.03648,(Limnonectes_magnus:0.111681,Limnonectes_modestus:0.111681):0.127673):0.079312):0.163611):0.021098,((((((((Nanorana_arnoldi:0.004514,Nanorana_chayuensis:0.004514):0.014484,Nan

orana_maculosa:0.018998):0.014969,Nanorana_medogensis:0.033967):0.029611,(Nanorana_conaensis:0.045451,Nanorana_blanfordii:0.045451):0.018128):0.021771,((Nanorana_polunini:0.016705,Nanorana_rostandi:0.016705):0.028359,Nanorana_liebigii:0.045064):0.040285):0.039608,((Nanorana_pleskei:0.041094,Nanorana_ventripunctata:0.041094):0.034598,Nanorana_parkeri:0.075692):0.049265):0.018532,(((Nanorana_aenea:0.070351,Nanorana_unculuanus:0.070351):0.032384,(Nanorana_bourreti:0.030177,Nanorana_yunnanensis:0.030177):0.072558):0.021804,(Nanorana_quadranus:0.103542,Nanorana_taihangnica:0.103542):0.020998):0.018949):0.078439,((((Quasipaa_boulengeri:0.068885,Quasipaa_spinosa:0.068885):0.032745,Quasipaa_shini:0.10163):0.013537,Quasipaa_yei:0.115167):0.035918,Quasipaa_verrucospinosa:0.151085):0.070843):0.281447):0.06447,((((Occidozyga_laevis:0.25994,Occidozyga_martensii:0.25994):0.056097,Occidozyga_baluensis:0.316037):0.062397,Occidozyga_lima:0.378434):0.079862,(Ingerana_borealis:0.181083,Ingerana_tenasserimensis:0.181083):0.277213):0.109549);

Elapidae

((((((((((((((((Hoplocephalus_bitorquatus:0.100408,Paroplocephalus_atriceps:0.100408):0.039832,(Notechis_scutatus:0.057908,Tropidechis_carinatus:0.057908):0.082332):0.03797,Austrelaps_superbus:0.178211):0.023795,Drysdalia_coronoides:0.202006):0.039266,Echiopsis_curta:0.241272):0.015764,Hemiaspis_dameli:0.257037):0.021134,(((Hydrelaps_darwiniensis:0.169631,Hydrophis_cyanocinctus:0.169631):0.014007,(Ephalophis_greyae:0.116568,Parahydrophis_mertoni:0.116568):0.06707):0.035262,(Aipysurus_laevis:0.121515,Emydocephalus_annulatus:0.121515):0.097385):0.05927):0.026387,((((Parasuta_monachus:0.158849,Suta_suta:0.158849):0.061776,Elapognathus_coronatus:0.220625):0.025223,(Cryptophis_nigrescens:0.186564,Rhinoplocephalus_bicolor:0.186564):0.059283):0.039556,Cacophis_squamulosus:0.285404):0.019154):0.017921,((((((Denisonia_devisi:0.213281,Pseudechis_australis:0.213281):0.054098,(Neelaps_calonotus:0.231136,Vermicella_intermedia:0.231136):0.036242):0.013263,(Oxyuranus_microlepidotus:0.18646,Pseudonaja_textilis:0.18646):0.094183):0.010902,(Aspidomorphus_muelleri:0.244635,Furina_diadema:0.244635):0.04691):0.013565,Acanthophis_praelongus:0.305109):0.007255,(Brachyurophissemifasciatus:0.251651,Simoselaps_anomalus:0.251651):0.060714):0.010114):0.019239,(Demansia_papuensis:0.221266,Toxicocalamus_preussi:0.221266):0.120452):0.016507,Micropechis_ikaheka:0.358224):0.02155,Laticauda_colubrina:0.379774):0.039979,(((((((Bungarus_candidus:0.066612,Bungarus_multicinctus:0.066612):0.061165,Bungarus_niger:0.127777):0.051899,(Bungarus_caeruleus:0.127587,Bungarus_sindanus:0.127587):0.052088):0.052149,Bungarus_fasciatus:0.231825):0.047009,Bungarus_ceylonicus:0.278834):0.05211,(Bungarus_bungaroides:0.098076,Bungarus_slowinskii:0.098076):0.232868):0.047785,Bungarus_flaviceps:0.378729):0.041024):0.008922,((((((((Naja_annulifera:0.03095,Naja_haje:0.03095):0.064507,Naja_nivea:0.095457):0.104735,((Naja_melanoleuca:0.128735,Naja_annulata:0.128735):0.025014,Naja_multifasciata:0.15375):0.046443):0.033648,((((Naja_ashei:0.04419,Naja_mossambica:0.04419):0.018505,Naja_nigricollis:0.062695):0.042683,Naja_katiensis:0.105377):0.057786,(Naja_nubiae:0.103062,Naja_pallida:0.103062):0.060101):0.070677):0.019048,(((Naja_atra:0.07887,Naja_kaouthia:0.07887):0.011773,(Naja_siamensis:0.046613,Naja_sumatrana:0.046613):0.04403):0.02406,Naja_naja:0.114703):0.138186):0.025765,Hemachatus_haemachatus:0.278653):0.056257,(Aspidelaps_scutatus:0.280203,Walterinnesia_aegyptia:0.280203):0.054708):0.065365,(((Dendroaspis_angusticeps:0.316527,Ophiophagus_hannah:0.316527):0.035416,Elapsoidea_semiannulata:0.351943):0.026364,(Hemibungarus_calligaster:0.290603,Calliophis_bibroni:0.290603):0.087704):0.02197):0.028399):0.007257,((((Sinomicrurus

_macclellandi:0.168413,Sinomicrurus_kelloggii:0.168413):0.081688,Sinomicrurus_japonicus:0.2
50101):0.117122,Micrurus_surinamensis:0.367222):0.041054,Micruroides_euryxanthus:0.408276)
:0.027656):0.021212,(((Calliophis_castoe:0.18369,Calliophis_nigrescens:0.18369):0.123815,Calli
ophis_melanurus:0.307506):0.10904,(Calliophis_bivirgata:0.284359,Calliophis_intestinalis:0.284
359):0.132187):0.040598);

Gekkonidae

((Alsophylax_pipiens:0.759255,Microgecko_persicus:0.759255):0.065634,(((Calodactylodes_illin
gworthorum:0.746996,((Chondrodactylus_fitzsimonsi:0.646981,Homopholis_walbergii:0.646981):
0.062535,(Phelsuma_inexpectata:0.18113,Phelsuma_rosagularis:0.18113):0.528386):0.03748):0.0
49408,((Dixonius_vietnamensis:0.663639,Nactus_vankampeni:0.663639):0.114176,(Gehyra_muti
lata:0.670201,(((Hemiphyllodactylus_bintik:0.160727,Hemiphyllodactylus_harterti:0.160727):0.2
75982,(Hemiphyllodactylus_titiwangsaensis:0.28322,(Hemiphyllodactylus_larutensis:0.106881,H
emiphyllodactylus_tehtarik:0.106881):0.176339):0.153489):0.123737,((Hemiphyllodactylus_gano
klonis:0.27536,(Hemiphyllodactylus_insularis:0.21351,Hemiphyllodactylus_typus:0.21351):0.061
85):0.170937,(Hemiphyllodactylus_aurantiacus:0.411384,((Hemiphyllodactylus_banaensis:0.1760
4,Hemiphyllodactylus_dushanensis:0.17604):0.207609,((Hemiphyllodactylus_chiangmaiensis:0.1
88825,(Hemiphyllodactylus_yunnanensis:0.149199,Hemiphyllodactylus_jinpingensis:0.149199):0.
039626):0.075843,(Hemiphyllodactylus_longlingensis:0.217313,(Hemiphyllodactylus_zayuensis:
0.149772,Hemiphyllodactylus_changningensis:0.149772):0.067541):0.047355):0.118981):0.0277
35):0.034913):0.114148):0.109755):0.107614):0.01859):0.017167,(((Lepidodactylus_orientalis:0.
486626,(Lepidodactylus_lugubris:0.437388,Luperosaurus_cumingii:0.437388):0.049238):0.16251
7,(Gekko_monarchus:0.530417,(Gekko_vittatus:0.480955,(Gecko_smithi:0.194568,Gekko_gecko:
0.194568):0.286386):0.049462):0.118726):0.144628,(((Altiphylax_stolickzai:0.261474,Altiphyla
x_medogensis:0.261474):0.28936,((Altiphylax_levitoni:0.495353,(Mediodactylus_russowii:0.425
595,Tropiocolotes_nubicus:0.425595):0.069758):0.041088,((Cyrtopodion_mansarulus:0.285239,
Cyrtopodion_scabrum:0.285239):0.118716,(Tenuidactylus_caspius:0.365761,(Crossobamon_orie
ntalis:0.322603,Cyrtopodion_aravallensis:0.322603):0.043158):0.038194):0.132485):0.014395):0.
181732,((Hemidactylus_anamallensis:0.658436,(((Hemidactylus_aaronbaueri:0.320593,(Hemidac
tylus_depressus:0.226104,(Hemidactylus_triedrus:0.173678,(Hemidactylus_maculatus:0.10481,H
emidactylus_prashadi:0.10481):0.068868):0.052426):0.094489):0.136119,((Hemidactylus_lesche
naulti:0.140406,Hemidactylus_flaviviridis:0.140406):0.295188,(Hemidactylus_frenatus:0.352722,
(Hemidactylus_brookii:0.245837,(Hemidactylus_gracilis:0.154203,Hemidactylus_imbricatus:0.15
4203):0.091634):0.106885):0.082871):0.021119):0.072145,(((Hemidactylus_craspedotus:0.28371
2,Hemidactylus_platyurus:0.283712):0.052897,((Hemidactylus_karenorum:0.142528,Hemidactyl
us_giganteus:0.142528):0.11895,(Hemidactylus_garnotii:0.236527,(Hemidactylus_aquilonius:0.1
02022,Hemidactylus_bowringii:0.102022):0.134505):0.02495):0.075132):0.173431,((Hemidactyl
us_fasciatus:0.443659,(Hemidactylus_persicus:0.240134,Hemidactylus_robustus:0.240134):0.203
525):0.047251,(Hemidactylus_angulatus:0.450801,(Hemidactylus_mabouia:0.338735,(Hemidacty
lus_palaichthus:0.275599,(Hemidactylus_brasilianus:0.191929,Hemidactylus_greeffii:0.191929):
0.08367):0.063136):0.112066):0.040109):0.01913):0.018817):0.129579):0.027688,(((Cyrtodactyl
us_tibetanus:0.233137,Cyrtodactylus_zhaoermii:0.233137):0.320952,((Cyrtodactylus_battalensis:
0.176653,Cyrtodactylus_chamba:0.176653):0.038985,(Cyrtodactylus_himalayanus:0.18346,Cyrto
dactylus_lawderanus:0.18346):0.032178):0.338451):0.061403,(((Cyrtodactylus_russelli:0.096623,

Cyrtodactylus_slowinskii:0.096623):0.36626,(Cyrtodactylus_fasciolatus:0.448682,(((Cyrtodactylus_annandalei:0.126573,Cyrtodactylus_feae:0.126573):0.250995,(Cyrtodactylus_sp_Khellong:0.260491,(Cyrtodactylus_gubernatoris:0.171309,Cyrtodactylus_sp_Sikkim:0.171309):0.089182):0.117077):0.040536,((Cyrtodactylus_cayuensis:0.036395,Cyrtodactylus_sp_Glow:0.036395):0.32851,(Cyrtodactylus_brevidactylus:0.336078,((Cyrtodactylus_kazirangaensis:0.228651,(((Cyrtodactylus_ayeyarwadyensis:0.09184,Cyrtodactylus_tripuraensis:0.09184):0.097593,(Cyrtodactylus_khasiensis:0.161943,(Cyrtodactylus_guwahatiensis:0.06199,Cyrtodactylus_septentrionalis:0.06199):0.099953):0.027489):0.039219):0.086295,(Cyrtodactylus_sp_CHNG:0.300127,(Cyrtodactylus_nagalandensis:0.255779,(Cyrtodactylus_gansi:0.239276,(Cyrtodactylus_jaintiaensis:0.206237,(Cyrtodactylus_sp_Mizoram:0.09114,Cyrtodactylus_montanus:0.09114):0.115096):0.033039):0.016503):0.044347):0.014819):0.021132):0.028826):0.0532):0.030577):0.014201):0.087908,((Cyrtodactylus_jarujini:0.326911,(Cyrtodactylus_angularis:0.295762,Cyrtodactylus_chanhomeae:0.295762):0.03115):0.165282,(((Cyrtodactylus_philippinicus:0.333988,(Cyrtodactylus_paradoxus:0.007877,Cyrtodactylus_chrysopylos:0.007877):0.326111):0.047213,((Cyrtodactylus_jellesmae:0.311848,(Cyrtodactylus_sadlieri:0.233526,Cyrtodactylus_sp_Timor:0.233526):0.078323):0.041523,(Cyrtodactylus_quadrivirgatus:0.328104,(Cyrtodactylus_rubidus:0.293205,(Cyrtodactylus_tiomanensis:0.242627,(Cyrtodactylus_semenanjungensis:0.060939,Cyrtodactylus_adleri:0.060939):0.181687):0.050578):0.0349):0.025267):0.02783):0.075856,((Cyrtodactylus_triedra:0.402873,(Cyrtodactylus_deccanensis:0.29326,Cyrtodactylus_collegalensis:0.29326):0.109614):0.036267,(((Cyrtodactylus_bintangtinggi:0.141595,Cyrtodactylus_pulchellus:0.141595):0.233879,(Cyrtodactylus_elok:0.21711,Cyrtodactylus_interdigitalis:0.21711):0.158365):0.042013,((Cyrtodactylus_hontreensis:0.242286,Cyrtodactylus_intermedius:0.242286):0.156054,((Cyrtodactylus_novaeguineae:0.296774,(Cyrtodactylus_loriae:0.245333,Cyrtodactylus_sermowaiensis:0.245333):0.051441):0.065898,(Cyrtodactylus_tigroides:0.283221,(Cyrtodactylus_peguensis:0.223462,Cyrtodactylus_oldhami:0.223462):0.059758):0.079451):0.035668):0.019148):0.021653):0.017917):0.035136):0.058598):0.064701):0.070631):0.046444):0.061204):0.0198):0.011318);

Megophryidae

((((((((((((((Megophrys_sangzhiensis:0.023862,Megophrys_spinata:0.023862):0.024713,Megophrys_binlingensis:0.048575):0.020433,(Megophrys_binchuanensis:0.055068,Megophrys_omeimontis:0.055068):0.013941):0.014207,Megophrys_jingdongensis:0.083215):0.011967,Megophrys_palpebralespinosa:0.095182):0.01349,Megophrys_wuliangshanensis:0.108672):0.016082,Megophrys_daweimontis:0.124755):0.03689,Megophrys_minor:0.161645):0.017186,((((Megophrys_brachykolos:0.067479,Megophrys_lini:0.067479):0.021585,Megophrys_jinggangensis:0.089064):0.021281,(Megophrys_boettgeri:0.023198,Megophrys_huangshanensis:0.023198):0.087147):0.013915,((Megophrys_baolongensis:0.047318,Megophrys_wushanensis:0.047318):0.037967,Megophrys_tuberogranulatus:0.085286):0.038974):0.054571):0.057888,(Ophryophryne_hansi:0.103829,Ophryophryne_microstoma:0.103829):0.13289):0.020592,(Megophrys_pachyproctus:0.129493,Megophrys_vegrandis:0.129493):0.127818):0.025881,(((((((((Megophrys_mangshanensis:0.034481,Megophrys_maosonensis:0.034481):0.050702,Megophrys_major:0.085183):0.01744,Megophrys_flavipunctata:0.102623):0.013694,(Megophrys_glandulosa:0.086287,Megophrys_periosa:0.086287):0.030029):0.016793,(((Megophrys_medogensis:0.048168,Megophrys_robusta:0.048168):0.050447,Megophrys_zhangi:0.098615):0.021228,Megophrys_himalayana:0.119843):0.013266):0.0285,Megophrys_oreocrypta:0.16161):0.043168,(((Megophrys_ancrae:0.105861,Megophrys_serchhipii:0.10

5861):0.02312,(Megophrys_oropedion:0.101222,Megophrys_zunhebotoensis:0.101222):0.027759):0.035818,Megophrys_megacephala:0.164799):0.039978):0.02118,Megophrys_parva:0.225957):0.032499,Megophrys_aceras:0.258456):0.024736):0.021564,((((Borneophrys_edwardinae:0.101334,Megophrys_ligayae:0.101334):0.048001,Megophrys_nasuta:0.149335):0.042604,Megophrys_stejnegeri:0.191939):0.086514,Megophrys_montana:0.278452):0.026303):0.015893,(((Megophrys_gigantica:0.050284,Megophrys_shapingensis:0.050284):0.029825,Megophrys_wawuensis:0.080108):0.186514,(Brachytarsophrys_carinense:0.083221,Brachytarsophrys_feae:0.083221):0.183401):0.054026):0.101913,(((((((((Scutiger_boulengeri:0.011197,Scutiger_mammatus:0.011197):0.040164,Scutiger_liupanensis:0.0513600009262562):0.010158,Scutiger_tuberculatus:0.061519):0.01181,Scutiger_glandulatus:0.073329):0.03839,Scutiger_occidentalis:0.111719):0.015692,((((Scutiger_nyingchiensis:0.050736,Scutiger_gongshanensis:0.050736):0.023991,Scutiger_spinosus:0.074728):0.029566,Scutiger_nepalensis:0.104294):0.010076,Scutiger_sikkimensis:0.11437):0.013042):0.015264,(Scutiger_chintingensis:0.111392,Scutiger_ningshanensis:0.111392):0.031283):0.055006,Scutiger_wuguanfui:0.197682):0.098171,((((Leptobrachium_hainanense:0.05584,Leptobrachium_ngoclinhense:0.05584):0.058829,Leptobrachium_banae:0.11467):0.103629,((Leptobrachium_boringii:0.085528,Leptobrachium_huashen:0.085528):0.095653,Leptobrachium_bompu:0.181181):0.037118):0.038042,((Leptobrachium_hasseltii:0.09162,Leptobrachium_hendricksoni:0.09162):0.105253,Leptobrachium_smithi:0.196873):0.059468):0.039511):0.059477,(Leptolalax_oshanensis:0.160979,Leptolalax_ventripunctatus:0.160979):0.194351):0.067232);

Pareidae

(((((((Pareas_formosensis:0.050645,Pareas_hamptoni:0.050645):0.231222995761871,((Pareas_atayal:0.111048,Pareas_iwasakii:0.111048):0.026257,Pareas_komaii:0.137305):0.144562):0.129184,((Pareas_boulengeri:0.182013,Pareas_chinensis:0.182013):0.155858,Pareas_stanleyi:0.337871):0.07318):0.044368,Pareas_margaritophorus:0.455418998137474):0.057758,Pareas_monticola:0.513178002717972):0.08866,((Pareas_carinatus:0.377818,Pareas_nuchalis:0.377818):0.154372,Aplopeltura_boa:0.532191):0.069647):0.051149,((Asthenodipsas_laevis:0.136827,Asthenodipsas_malaccanus:0.136827):0.420271,(Asthenodipsas_tropidonotus:0.185642,Asthenodipsas_vertebralis:0.185642):0.371455):0.095889);

Ranidae

((((((((((((((((((Hylarana_indica:0.046459,Hylarana_sreeni:0.046459):0.02851,Hylarana_caesari:0.074969):0.055357,Hylarana_magna:0.130326):0.013426,Hylarana_montana:0.143753):0.021781,Hylarana_flavescens:0.165534):0.020837,((Hylarana_aurantiaca:0.080828,Hylarana_urbis:0.080828):0.017451,(Hylarana_doni:0.065386,Hylarana_intermedia:0.065386):0.032892):0.088092):0.069762,(Hylarana_serendipi:0.192168,Hylarana_temporalis:0.192168):0.063965):0.057653,((((((Hylarana_aurata:0.078696,Hylarana_volkerjane:0.078696):0.021944,Hylarana_supragrisea:0.10064):0.023581,Hylarana_milneana:0.12422):0.047927,(Hylarana_jimiensis:0.109959,Hylarana_arfaki:0.109959):0.062188):0.008603,(Hylarana_kreffti:0.05816,Hylarana_papua:0.05816):0.12259):0.016537,((Hylarana_garritor:0.098975,Hylarana_waliesa:0.098975):0.075671,Hylarana_daemeli:0.174645):0.022642):0.116499):0.014599,((((Hylarana_mortenseni:0.007282,Hylarana_faber:0.007282):0.219971995563745,(((Hylarana_maosonensis:0.038402,Hylarana_spinulosa:0.038402):0.029705,Hylarana_latouchii:0.068107):0.092623,Hylarana_cubitalis:0.16073):0.066525):0.04402,(((Hylarana_bahuvistara:0.014455,Hylarana_malabaricus:0.014455):0.130477,Hylarana_nigrovittat

a:0.144932):0.040448,Hylarana_gracilis:0.18538):0.085894):0.038557,((Hylarana_attigua:0.1545
07,Hylarana_milleti:0.154507):0.071973,Hylarana_galamensis:0.22648):0.083352):0.018553):0.0
22978,((((Pelophylax_lateralis:0.171086,Hylarana_miopus:0.171086):0.070929,Hylarana_guenth
eri:0.242014):0.033688,Hylarana_humeralis:0.275702):0.027585,((Hylarana_erythraea:0.054623,
Hylarana_tytleri:0.054623):0.138927,(Hylarana_macrodactyla:0.116423,Hylarana_taipehensis:0.1
16423):0.077127):0.109737):0.048075):0.007304,((((((((Hylarana_grandocula:0.038167,Hylarana
_similis:0.038167):0.037682,Hylarana_melanomenta:0.075849):0.057434,(Hylarana_mangyanum:
0.057307,Hylarana_moellendorffi:0.057307):0.075976):0.074486,(Hylarana_picturata:0.091376,
Hylarana_signata:0.091376):0.116392):0.026361,(Hylarana_banjarana:0.192993,Hylarana_siberu:
0.192993):0.041137):0.033209,(((Hylarana_glandulosa:0.006796,Hylarana_baramica:0.006796):0.
059468,Hylarana_laterimaculata:0.066264):0.134461,Hylarana_rawa:0.200725):0.066613):0.024
167,(((Hylarana_amnicola:0.090667,Hylarana_darlingi:0.090667):0.053301,Hylarana_albolabris:
0.143968):0.056886,Hylarana_lepus:0.200854):0.090651):0.04151,((((((Hylarana_chalconota:0.0
18626,Hylarana_megalonesa:0.018626):0.006451,Hylarana_eschatia:0.025076):0.038002,Hylaran
a_parvaccola:0.063079):0.082078,Hylarana_mocquardi:0.145156):0.047518,Hylarana_rufipes:0.1
92674):0.092409,Hylarana_nicobariensis:0.285083):0.047932):0.025652):0.00894,Hylarana_lept
oglossa:0.367606):0.011184,((Babina_chapaensis:0.106296,Babina_okinavana:0.106296):0.06739
3,Babina_pleuraden:0.173688):0.205102):0.015981,((((Glandirana_emeljanovi:0.139745,Glandira
na_rugosa:0.139745):0.05985,Glandirana_tientaiensis:0.199595):0.098721,(Sanguirana_luzonensi
s:0.1667,Sanguirana_sanguinea:0.1667):0.131616):0.046996,Abavorana_luctuosa:0.345312):0.04
9459):0.013845,((((((((((Odorrana_amamiensis:0.03513,Odorrana_narina:0.03513):0.031073,Od
orrana_supranarina:0.066203):0.052859,(Odorrana_swinhoana:0.044523,Odorrana_utsunomiyaor
um:0.044523):0.074539):0.05008,(((Odorrana_exiliversabilis:0.043825,Odorrana_nasuta:0.04382
5):0.066413,Odorrana_tormota:0.110238):0.02447,(Odorrana_nasica:0.053895,Odorrana_versabil
is:0.053895):0.080813):0.034434):0.026583,(((((Odorrana_aureola:0.045005,Odorrana_livida:0.0
45005):0.030096,Odorrana_chloronota:0.075101):0.015305,(Odorrana_graminea:0.078031,Odorr
ana_leporipes:0.078031):0.012376):0.04022,Odorrana_hosii:0.130627):0.041131,(Odorrana_mora
fkai:0.139727,Odorrana_banaorum:0.139727):0.032031):0.023967):0.017045,Odorrana_tiannane
nsis:0.21277):0.026335,(((((Odorrana_hejiangensis:0.013795,Odorrana_nanjiangensis:0.013795):
0.055324,Odorrana_bacboensis:0.069119):0.051193,(Odorrana_fengkaiensis:0.02341,Odorrana_h
ainanensis:0.02341):0.096902):0.02612,(Odorrana_huanggangensis:0.044936,Odorrana_tianmuii:
0.044936):0.101496):0.024116,Odorrana_schmackeri:0.170548):0.068557):0.016412,(((((((Odorr
ana_andersonii:0.023563,Odorrana_jingdongensis:0.023563):0.019753,Odorrana_junlianensis:0.0
433159995943308):0.022785,Odorrana_margaretae:0.066101):0.013762,Odorrana_kuangwuensis:
0.079863):0.055335,(Odorrana_mutschmanni:0.084499,Odorrana_wuchuanensis:0.084499):0.050
699):0.037918,(((Odorrana_anlungensis:0.075369,Odorrana_yizhangensis:0.075369):0.015689,O
dorrana_lungshengensis:0.091058):0.05051,Odorrana_lipuensis:0.141567):0.031549):0.046597,(
Odorrana_chapaensis:0.145767,Odorrana_geminata:0.145767):0.073946):0.035804):0.024355,Od
orrana_ishikawae:0.279873):0.023115,Odorrana_absita:0.302988):0.079028,((((Rana_catesbeianu
s:0.161843,Rana_septentrionalis:0.161843):0.096886,(Rana_pipiens:0.177412,Rana_tlaloci:0.177
412):0.081318):0.058739,(Rana_kukunoris:0.209132,Rana_temporaria:0.209132):0.108336):0.02
4366,Pseudorana_weiningensis:0.341834):0.040182):0.0266):0.026761,((Pelophylax_ridibundus:
0.267273,Pelophylax_saharicus:0.267273):0.121221,Pelophylax_nigromaculatus:0.388494):0.046
882):0.011757,(((((((((((Amolops_iriodes:0.019238,Amolops_mengyangensis:0.019238):0.01328,

Amolops_daorum:0.032518):0.055072,(Amolops_akhaorum:0.069636,Amolops_archotaphus:0.069636):0.017954):0.037051,(((Amolops_compotrix:0.040044,Amolops_cucae:0.040044):0.039348,Amolops_vitreus:0.079392):0.032659,Amolops_chunganensis:0.112051):0.012591):0.012894,((Amolops_bellulus:0.058083,Amolops_nyingchiensis:0.058083):0.052814,Amolops_aniqiaoensis:0.110896):0.026639):0.033933,((((((Amolops_jinjiangensis:0.021779,Amolops_tuberodepressus:0.021779):0.017018,Amolops_loloensis:0.038797):0.013546,Amolops_mantzorum:0.052343):0.007308,Amolops_liangshanensis:0.059651):0.057053,(Amolops_granulosus:0.008827,Amolops_lifanensis:0.008827):0.107877):0.027541,Amolops_chayuensis:0.144245):0.027223):0.024131,((Amolops_viridimaculatus:0.076585,Amolops_beibengensis:0.076585):0.043919,Amolops_medogensis:0.120505):0.075094):0.023611,(((Amolops_sp:0.070998,Amolops_gyirongensis:0.070998):0.023378,Amolops_wangyufani:0.094376):0.012049,Amolops_deng:0.106426):0.112784):0.114817,((((Amolops_indoburmanensis:0.115254,Amolops_afghanus:0.115254):0.064079,Amolops_marmoratus:0.179333):0.034874,Amolops_yarlungzangbo:0.214207):0.038116,Amolops_panhai:0.252323):0.081704):0.018944,(Amolops_larutensis:0.259894,Amolops_cremnobatus:0.259894):0.093076):0.025588,(((((Amolops_ricketti:0.054304,Amolops_albispinus:0.054304):0.049632,Amolops_wuyiensis:0.103936):0.133322,(Amolops_daiyunensis:0.079541,Amolops_hongkongensis:0.079541):0.157717):0.065708,(Amolops_hainanensis:0.140627,Amolops_torrentis:0.140627):0.16234):0.041795,Amolops_spinapectoralis:0.344761):0.033797):0.068576):0.016422,((((((Meristogenys_dyscritus:0.068077,Meristogenys_maryatiae:0.068077):0.018227,Meristogenys_stigmachilus:0.086304):0.011159,Meristogenys_poecilus:0.097463):0.011106,(Meristogenys_amoropalamus:0.084831,Meristogenys_stenocephalus:0.084831):0.023738):0.231069,(Huia_cavitympanum:0.0935900013751984,Huia_masonii:0.093589):0.246048):0.027229,((Clinotarsus_alticola:0.147843,Clinotarsus_curtipes:0.147843):0.16937,Huia_melasma:0.317213):0.049653):0.09669):0.05106,((Staurois_guttatus:0.133828,Staurois_natator:0.133828):0.049804,Staurois_latopalmatus:0.183631):0.330985);

Rhacophoridae

((((((((((((((((((((Raorchestes_luteolus:0.039224,Raorchestes_travancoricus:0.039224):0.078659,(Raorchestes_agasthyaensis:0.057299,Raorchestes_kadalarensis:0.057299):0.060583):0.011013,Raorchestes_chlorosomma:0.128896):0.023342,(((Raorchestes_kaikatti:0.045451,Raorchestes_kakachi:0.045451):0.036441,Raorchestes_sushili:0.081892):0.035215,Raorchestes_anili:0.117107):0.03513):0.023252,((((Raorchestes_beddomii:0.051797,Raorchestes_theuerkaufi:0.051797):0.058988,Raorchestes_munnarensis:0.110785):0.009511,Raorchestes_resplendens:0.120296):0.0232,Raorchestes_dubois:0.143497):0.031993):0.016945,(((Raorchestes_chalazodes:0.030836,Raorchestes_flavioculari s:0.030836):0.019813,Raorchestes_ochlandrae:0.050649):0.105645,(Raorchestes_manohari:0.04021,Raorchestes_uthamani:0.04021):0.116084):0.036141):0.040888,((Raorchestes_blandus:0.147332,Raorchestes_archeos:0.147332):0.037926,Raorchestes_chotta:0.185257):0.048065):0.023935,((((((Raorchestes_glandulosus:0.07336,Raorchestes_jayarami:0.07336):0.026669,Raorchestes_bobingeri:0.100029):0.029463,Raorchestes_akroparallagi:0.129492):0.03997,(Raorchestes_graminirupes:0.080313,Raorchestes_johnceei:0.080313):0.089149):0.012694,Raorchestes_crustai:0.182156):0.038448,Raorchestes_nerostagona:0.220604):0.036653):0.035286,((((((((Raorchestes_menglaensis:0.056806,Raorchestes_parvulus:0.056806):0.027918,Raorchestes_gryllus:0.084724):0.01057,Raorchestes_longchuanensis:0.095295):0.039324,((Raorchestes_bombayensis:0.069776,Raorchestes_leucolatus:0.069776):0.019843,Raorchestes_tuberohumerus:0.089619):0.044999):

0.024457,Raorchestes_ghatei:0.159075):0.034731,((Raorchestes_hassanensis:0.051004,Raorchestes_ponmudi:0.051004):0.041384,Raorchestes_flaviventris:0.092389):0.101418):0.0121,Raorchestes_indigo:0.205906):0.020747,Raorchestes_echinatus:0.226654):0.011413,(((((Raorchestes_aureus:0.057913,Raorchestes_lechiya:0.057913):0.055562,Raorchestes_signatus:0.113475):0.022165,(Raorchestes_chromasynchysi:0.060257,Raorchestes_silentvalley:0.060257):0.075383):0.015682,(Raorchestes_primarrumpfi:0.055661,Raorchestes_tinniens:0.055661):0.09566):0.043736,((((Raorchestes_charius:0.065508,Raorchestes_honnametti:0.065508):0.042623,Raorchestes_griet:0.108131):0.019773,Raorchestes_coonoorensis:0.127903):0.046938,Raorchestes_marki:0.174842):0.020216):0.043009):0.054477):0.033894,((((((((Pseudophilautus_amboli:0.01198,Pseudophilautus_leucorhinus:0.01198):0.03794,Pseudophilautus_wynaadensis:0.04992):0.036579,Pseudophilautus_kani:0.0865):0.061954,Pseudophilautus_folicola:0.148454):0.035476,((Pseudophilautus_limbus:0.132027,Pseudophilautus_zorro:0.132027):0.037128,(Pseudophilautus_schneideri:0.085003,Pseudophilautus_simba:0.085003):0.084152):0.014775):0.027367,(((((Pseudophilautus_asankai:0.024861,Pseudophilautus_hoffmanni:0.024861):0.074865,Pseudophilautus_pleurotaenia:0.099726):0.038674,Pseudophilautus_ocularis:0.1384):0.02627,(Pseudophilautus_decoris:0.030508,Pseudophilautus_mittermeieri:0.030508):0.134161):0.017971,Pseudophilautus_tanu:0.18264):0.028657):0.023768,((((((((Pseudophilautus_hankeni:0.032732,Pseudophilautus_dilmah:0.032732):0.01603,Pseudophilautus_schmarda:0.048763):0.055179,Pseudophilautus_lunatus:0.103941):0.017938,Pseudophilautus_sarasinorum:0.12188):0.008333,((Pseudophilautus_alto:0.089686,Pseudophilautus_stuarti:0.089686):0.017031,Pseudophilautus_popularis:0.106717):0.023495):0.023988,Pseudophilautus_cavirostris:0.154201):0.011903,Pseudophilautus_bambaradeniyai:0.166103):0.03241,((Pseudophilautus_mooreorum:0.043738,Pseudophilautus_poppiae:0.043738):0.075407,Pseudophilautus_stellatus:0.119145):0.079368):0.036553):0.015802,((Pseudophilautus_microtympanum:0.058305,Pseudophilautus_steineri:0.058305):0.065143,Pseudophilautus_papillosus:0.123448):0.12742):0.07557):0.010244,Mercurana_myristicapalustris:0.336682):0.027413,Beddomixalus_bijui:0.364095):0.040705,((((((Kurixalus_baliogaster:0.020296,Kurixalus_bisacculus:0.020296):0.035805,Kurixalus_odontotarsus:0.056101):0.059284,(Kurixalus_naso:0.05482,Kurixalus_verrucosus:0.05482):0.060564):0.070441,((Kurixalus_banaensis:0.114474,Kurixalus_viridescens:0.114474):0.051778,Kurixalus_motokawai:0.166252):0.019573):0.051377,(((Kurixalus_berylliniris:0.069776,Kurixalus_eiffingeri:0.069776):0.022275,Kurixalus_wangi:0.09205):0.06831,Kurixalus_idiootocus:0.160361):0.076842):0.057831,Kurixalus_appendiculatus:0.295033):0.109766):0.009731,(Nasutixalus_jerdonii:0.179873,Nasutixalus_medogensis:0.179873):0.234658):0.017578,((((((((((Philautus_juliandringi:0.087469,Philautus_umbra:0.087469):0.023355,Philautus_mjobergi:0.110824):0.032214,(Philautus_amoenus:0.098163,Philautus_nephophilus:0.098163):0.044875):0.031373,Philautus_kakipanjang:0.174411):0.019853,(((Philautus_acutirostris:0.014316,Philautus_worcesteri:0.014316):0.099187,Philautus_surdus:0.113502):0.072107,(Philautus_petersi:0.139612,Philautus_refugii:0.139612):0.045998):0.008654):0.021992,(Philautus_davidlabangi:0.063086,Philautus_disgregus:0.063086):0.153169):0.018102,(((Philautus_abditus:0.091823,Philautus_vermiculatus:0.091823):0.038435,(Philautus_acutus:0.055953,Philautus_aurantium:0.055953):0.074305):0.013763,(Philautus_bunitus:0.021947,Philautus_kerangae:0.021947):0.122074):0.090337):0.013103,Philautus_aurifasciatus:0.247461):0.011503,(Philautus_macroscelis:0.142054,Philautus_everetti:0.142054):0.11691):0.009721,((Philautus_hosii:0.103654,Philautus_ingeri:0.103654):0.1086,Philautus_tectus:0.212255):0.05643):0.046021,Philautus_cornutus:0.314705):0.117404):0.021569,(((((((((((((((((Rhacophorus_dugritei:0.012047,Rhacophorus_hui:0.012047):0.026007,Rhacophorus_minimus:0.038055):0.

005323,Rhacophorus_hongchibaensis:0.043378):0.012292,((Rhacophorus_hungfuensis:0.019356,
Rhacophorus_wui:0.019356):0.026606,Rhacophorus_puerensis:0.045962):0.009708):0.021342,R
hacophorus_schlegelii:0.077012):0.015215,((Rhacophorus_duboisi:0.0209,Rhacophorus_omeimo
ntis:0.0209):0.045188,Rhacophorus_burmanus:0.066088):0.026139):0.011588,(Rhacophorus_sma
ragdinus:0.083406,Rhacophorus_arboreus:0.083406):0.020409):0.016942,((Rhacophorus_lishuien
sis:0.021939,Rhacophorus_zhoukaiyae:0.021939):0.039123,Rhacophorus_dorsoviridis:0.061062):
0.059695):0.010891,Rhacophorus_moltrechti:0.131648):0.026436,Rhacophorus_feae:0.158085):0.
013122,((Rhacophorus_nigropunctatus:0.071193,Rhacophorus_pinglongensis:0.071193):0.03698,
Rhacophorus_chenfui:0.108173):0.063033):0.01857,Rhacophorus_dennysi:0.189777):0.05424,Rh
acophorus_vampyrus:0.244017):0.044545,(Rhacophorus_achantharrhena:0.085892,Rhacophorus_
dulitensis:0.085892):0.20267):0.033881,(((((Rhacophorus_belalongensis:0.051207,Rhacophorus_
gauni:0.051207):0.011577,Rhacophorus_gadingensis:0.062784):0.024186,Rhacophorus_bimacula
tus:0.08697):0.041157,(Rhacophorus_angulirostris:0.084695,Rhacophorus_penanorum:0.084695):
0.043432):0.14018,((((Rhacophorus_fasciatus:0.017671,Rhacophorus_harrissoni:0.017671):0.025
379,Rhacophorus_rufipes:0.04305):0.165251,Rhacophorus_monticola:0.208301):0.034643,Rhaco
phorus_cyanopunctatus:0.242944):0.025363):0.054136):0.030746,((((((((Rhacophorus_norhayatii:
0.060592,Rhacophorus_reinwardti:0.060592):0.030747,Rhacophorus_borneensis:0.091339):0.038
516,Rhacophorus_bipunctatus:0.129854):0.020592,(Rhacophorus_kio:0.078887,Rhacophorus_hel
enae:0.078887):0.071559):0.014421,Rhacophorus_rhodopus:0.164866999091148):0.084251,(((R
hacophorus_malabaricus:0.103441,Rhacophorus_pseudomalabaricus:0.103441):0.071118,Rhacop
horus_pardalis:0.174559):0.043236,(Rhacophorus_annamensis:0.10372,Rhacophorus_exechopyg
us:0.10372):0.114075):0.031324):0.022998,(((Rhacophorus_bengkuluensis:0.120572,Rhacophoru
s_catamitus:0.120572):0.084479,(Rhacophorus_indonesiensis:0.165388,Rhacophorus_robertinger
i:0.165388):0.039663):0.034265,Rhacophorus_poecilonotus:0.239316):0.0328):0.015419,(((((Rha
cophorus_orlovi:0.092067,Rhacophorus_tuberculatus:0.092067):0.102882,Rhacophorus_calcaneu
s:0.194949):0.019419,(Rhacophorus_baluensis:0.163841,Rhacophorus_nigropalmatus:0.163841):
0.050528):0.019066,Rhacophorus_translineatus:0.233435):0.016366,Rhacophorus_lateralis:0.249
801):0.037735):0.065652):0.057508,(((((((((((Polypedates_leucomystax:0.028919,Polypedates_te
raiensis:0.028919):0.021283,Polypedates_megacephalus:0.050201):0.011762,Polypedates_impres
us:0.061964):0.060358,((Polypedates_braueri:0.063324,Polypedates_mutus:0.063324):0.036733,
Polypedates_macrotis:0.100056):0.022266):0.024021,(Polypedates_cruciger:0.091527,Polypedate
s_maculatus:0.091527):0.054816):0.014417,Polypedates_discantus:0.16076):0.017091,Polypedat
es_pseudocruciger:0.177851):0.023157,Polypedates_colletti:0.201008):0.069579,Polypedates_otil
ophus:0.270587):0.02932,((Taruga_fastigo:0.05932,Taruga_longinasus:0.05932):0.022993,Taruga
_eques:0.082313):0.217594):0.069435,((Ghatixalus_asterops:0.139811,Ghatixalus_variabilis:0.13
9811):0.041462,Ghatixalus_magnus:0.181274):0.188069):0.023127,(((Feihyla_hansenae:0.14397
5,Feihyla_vittata:0.143975):0.157705,Feihyla_palpebralis:0.30168):0.034161,Feihyla_kajau:0.33
5841):0.056629):0.018227):0.011945,(((Chiromantis_petersii:0.08395,Chiromantis_xerampelina:
0.08395):0.094454,Chiromantis_rufescens:0.178403):0.091318,(Chiromantis_doriae:0.121793,Ch
iromantis_nongkhorensis:0.121793):0.147928):0.152921):0.011585,Gracixalus_medogensis:0.434
226):0.019451):0.01693,((((((Gracixalus_quangi:0.053458,Gracixalus_supercornutus:0.053458):0.
043391,Gracixalus_gracilipes:0.096849):0.032292,Gracixalus_seesom:0.129141):0.048054,Graci
xalus_quyeti:0.177194):0.078506,Gracixalus_lumarius:0.2557):0.046469,(((Gracixalus_nonggang
ensis:0.052751,Gracixalus_waza:0.052751):0.063093,Gracixalus_sapaensis:0.115844):0.014359,(

Gracixalus_jinxiuensis:0.109225,Gracixalus_jinggangensis:0.109225):0.020978):0.171966):0.168439):0.009446,(((((((((Theloderma_baibungense:0.0916459981703758,Theloderma_pyaukkya:0.091646):0.028175,Theloderma_asperum:0.119821):0.029204,Theloderma_licin:0.149025):0.009238,Theloderma_petilum:0.158264):0.030163,Theloderma_albopunctatum:0.188427):0.051885,((Theloderma_phrynoderma:0.129856,Theloderma_ryabovi:0.129856):0.028084,Theloderma_moloch:0.15794):0.082371):0.042025,Theloderma_leporosum:0.282337):0.026304,(((((((Theloderma_laeve:0.121659,Theloderma_truongsonense:0.121659):0.029374,Theloderma_nebulosum:0.151033):0.036269,Theloderma_annae:0.187302):0.057331,((Theloderma_bicolor:0.151719,Theloderma_corticale:0.151719):0.053409,Theloderma_gordoni:0.205128):0.039505):0.016924,Theloderma_rhododiscus:0.261557):0.013489,Theloderma_palliatum:0.275046):0.014293,(Theloderma_lateriticum:0.145131,Theloderma_lacustrinum:0.145131):0.144208):0.019302):0.022418,((Theloderma_horridum:0.08133,Theloderma_stellatum:0.08133):0.055693,Theloderma_vietnamense:0.137023):0.194036):0.031181,((Nyctixalus_pictus:0.204058,Nyctixalus_spinosus:0.204058):0.046637,Nyctixalus_margaritifer:0.250696):0.111544):0.117813):0.019894,(((((Liuixalus_calcarius:0.022565,Liuixalus_ocellatus:0.022565):0.011682,Liuixalus_romeri:0.034247):0.009876,Liuixalus_shiwandashan:0.044123):0.014417,Liuixalus_feii:0.05854):0.012924,Liuixalus_hainanus:0.071464):0.428483):0.044736,(((Buergeria_oxycephala:0.178761,Buergeria_robusta:0.178761):0.054573,Buergeria_buergeri:0.233333):0.097542,Buergeria_japonica:0.330875):0.213808);

Scincidae

((Typhlosaurus_lomiae:0.203951,Acontias_meleagris:0.203951):0.508416,(((((Eumeces_schneideri:0.233269,Scincus_mitranus:0.233269):0.238766,(Ophiomorus_punctatissimus:0.418765,Eurylepis_taeniolata:0.418765):0.053269):0.036706,((Chalcides_ocellatus:0.118784,(Chalcides_sexlineatus:0.071672,Chalcides_viridanus:0.071672):0.047112):0.286159,((Feylinia_polylepis:0.307215,Scelotes_mirus:0.307215):0.07393,(Madascincus_melanopleura:0.315113,Amphiglossus_reticulatus:0.315113):0.066031):0.023799):0.103797):0.038083,((Brachymeles_miriamae:0.285945,(Brachymeles_bonitae:0.16073,(Brachymeles_talinis:0.109124,(Brachymeles_gracilis:0.077319,Brachymeles_bicolor:0.077319):0.031805):0.051607):0.125214):0.233641,(Plestiodon_chinensis:0.266953,(((Plestiodon_fasciatus:0.051654,Plestiodon_kishinouyei:0.051654):0.139112,(Plestiodon_laticeps:0.142318,(Plestiodon_egregius:0.113775,Plestiodon_skiltonianus:0.113775):0.028543):0.048449):0.045382,(Plestiodon_quadrilineatus:0.202906,((Plestiodon_stimpsonii:0.097382,(Plestiodon_elegans:0.08119,(Plestiodon_marginatus:0.051842,Plestiodon_tamdaoensis:0.051842):0.029348):0.016192):0.043739,(Plestiodon_barbouri:0.125793,(Plestiodon_latiscutatus:0.107976,(Plestiodon_japonicus:0.025624,(Plestiodon_capito:0.007416,Plestiodon_tunganus:0.007416):0.018208):0.082352):0.017817):0.015329):0.061785):0.033242):0.030805):0.252632):0.027238):0.066988,((Ristella_rurkii:0.54598,(Tribolonotus_novaeguineae:0.524623,(((Mesoscincus_schwartzei:0.419002,(Lamprolepis_smaragdina:0.341903,((Lygosoma_koratense:0.1939,Lygosoma_quadrupes:0.1939):0.109336,(Lygosoma_albopunctatum:0.253215,(Lygosoma_punctata:0.227771,(Lygosoma_bowringii:0.190314,Lygosoma_lineolatum:0.190314):0.037457):0.025443):0.050021):0.038667):0.077099):0.056996,((Emoia_caeruleocauda:0.249456,(Emoia_cyanura:0.187752,Emoia_atrocostata:0.187752):0.061704):0.110098,((Leptosiaphos_vigintiserierum:0.290563,(Carlia_fusca:0.164809,Lygisaurus_novaeguineae:0.164809):0.125754):0.032192,(Eugongylus_rufescens:0.294058,(Nannoscincus_mariei:0.230909,(Oligosoma_lichenigera:0.160789,Oligosoma_aeneum:0.160789):0.07012):0.06315):0.028696):0.0368):0.116443):0.020624,((Tiliqua_scincoides:0.30919,Egernia_stokesi

i:0.30919):0.157675,((((Trachylepis_perrotetii:0.285623,Mabuya_mabouya:0.285623):0.04425,(H
eremites_vittatus:0.197695,Heremites_auratus:0.197695):0.132178):0.030352,(Eutropis_dissimili
s:0.304851,(Dasia_olivacea:0.232587,(Dasia_grisea:0.177753,Dasia_vittata:0.177753):0.054834):
0.072265):0.055373):0.040968,(Eutropis_longicaudata:0.362188,((Eutropis_multifasciata:0.2661
71,(Eutropis_macrophthalma:0.149736,Eutropis_rudis:0.149736):0.116436):0.068925,(Eutropis_t
ytleri:0.325061,((Eutropis_macularia:0.036768,Eutropis_rugifera:0.036768):0.268144,((Eutropis_
multicarinata:0.129777,(Eutropis_cumingi:0.088597,Eutropis_indeprensa:0.088597):0.04118):0.1
40281,((Eutropis_carinata:0.198088,(Eutropis_beddomei:0.080887,(Eutropis_nagarjunensis:0.042
418,Eutropis_trivittata:0.042418):0.038469):0.117201):0.056924,((Eutropis_bibronii:0.178977,Eu
tropis_quadricarinata:0.178977):0.058597,(Eutropis_madaraszi:0.185763,(Eutropis_allapallensis:
0.104907,Eutropis_clivicola:0.104907):0.080856):0.051811):0.017438):0.015046):0.034854):0.02
0149):0.010034):0.027092):0.039005):0.065672):0.029756):0.028001):0.021358):0.032038,(Sphe
nomorphus_praesignis:0.527611,(((Asymblepharus_nyingchiensis:0.245778,Asymblepharus_med
ogensis:0.245778):0.157691,(Ablepharus_pannonicus:0.305691,(Asymblepharus_alaicus:0.24624
6,Asymblepharus_sp:0.246246):0.059445):0.097778):0.110728,((((Tropidophorus_baconi:0.2068
08,Tropidophorus_grayi:0.206808):0.122512,(Tropidophorus_cocincinensis:0.242411,Tropidopho
rus_microlepis:0.242411):0.086909):0.049632,(Tropidophorus_brookei:0.20076,(Tropidophorus_
beccarii:0.153797,(Tropidophorus_misaminius:0.074171,Tropidophorus_partelloi:0.074171):0.07
9626):0.046962):0.178192):0.107143,((Isopachys_anguinoides:0.405989,(Tytthoscincus_aesculeti
cola:0.378345,(Sphenomorphus_stellatus:0.268706,((Tytthoscincus_atrigularis:0.182867,Tytthosc
incus_hallieri:0.182867):0.044777,(Tytthoscincus_parvus:0.14421,Sphenomorphus_buenloicus:0.
14421):0.083434):0.041062):0.109638):0.027645):0.053339,((Larutia_seribuatensis:0.432162,(((
Sphenomorphus_indicus:0.322728,Sphenomorphus_maculatus:0.322728004582405):0.049744,(S
phenomorphus_simus:0.3368,((Sphenomorphus_cyanolaemus:0.077137,Sphenomorphus_sabanus:
0.077137):0.185154,(Sphenomorphus_multisquamatus:0.185532,Sphenomorphus_variegatus:0.18
5532):0.076759):0.074509):0.035672):0.051513,((Scincella_reevesii:0.376797,((Insulasaurus_arb
orens:0.233323,(Insulasaurus_wrighti:0.179217,Insulasaurus_victoria:0.179217):0.054106):0.106
205,(Lipinia_pulchella:0.29429,(Lipinia_noctua:0.227152,Papuascincus_stanleyanus:0.227152):0.
067138):0.045238):0.037269):0.022002,(Scincella_doriae:0.385144,(Lipinia_vittigera:0.364168,(
Scincella_rupicola:0.351437,((Scincella_vandenburghi:0.258603,(Scincella_huanrenensis:0.0920
33,Scincella_tsinlingensis:0.092033):0.166569):0.065368,(Scincella_lateralis:0.279777,(Scincella
_assata:0.198374,(Scincella_cherriei:0.092925,Scincella_potanini:0.0929250024995804):0.10544
9):0.081403):0.044194):0.027466):0.012731):0.020976):0.013655):0.025186):0.008177):0.01234
5,((Sphenomorphus_concinnatus:0.34042,(Sphenomorphus_melanopogon:0.298693,((Sphenomor
phus_jobiensis:0.187414,Sphenomorphus_muelleri:0.187414):0.087727,((Sphenomorphus_solom
onis:0.06082,Sphenomorphus_scutatus:0.06082):0.174739,(Sphenomorphus_fasciatus:0.097528,(
Sphenomorphus_leptofasciatus:0.068629,(Sphenomorphus_cranei:0.029803,Sphenomorphus_mai
ndroni:0.029803):0.038826):0.028899):0.138031):0.039582):0.023552):0.041727):0.060049,(((Le
rista_neander:0.258977,Ctenotus_labillardieri:0.258977):0.067384,((Anomalopus_swansoni:0.255
416,Calyptotis_scutirostrum:0.255416):0.058513,(Hemiergis_peronii:0.279576,(Eulamprus_kosci
uskoi:0.139008,Eulamprus_quoyii:0.139008):0.140568):0.034353):0.012431):0.056993,(Otosaur
us_cumingi:0.358792,((Sphenomorphus_diwata:0.255039,(Sphenomorphus_acutus:0.241044,(Par
voscincus_steerei:0.159998,Parvoscincus_tagapayo:0.159998):0.081046):0.013996):0.042291,((T
ropidophorus_berdmorei:0.188915,(Tropidophorus_latiscutatus:0.091164,Tropidophorus_matsuii:

0.091164):0.097752):0.051102,((Tropidophorus_murphyi:0.107893,(Tropidophorus_baviensis:0.064144,Tropidophorus_hainanus:0.064144):0.043749):0.101941,(Tropidophorus_sinicus:0.185532,(Tropidophorus_robinsoni:0.123048,Tropidophorus_thai:0.123048):0.062483):0.024302):0.030184):0.057313):0.061462):0.024561):0.017115):0.044039):0.014821):0.026766):0.028104):0.013413):0.050408):0.035794):0.098555);

Viperidae
(((((((((((((Protobothrops_elegans:0.067677,Protobothrops_mucrosquamatus:0.067677):0.014036,(Protobothrops_maolanensis:0.050089,Protobothrops_trungkhanhensis:0.050089):0.031623):0.054702,(Protobothrops_flavoviridis:0.036487,Protobothrops_tokarensis:0.036487):0.099928):0.007474,(((Protobothrops_jerdonii:0.037299,Protobothrops_xiangchengensis:0.037299):0.05249,Protobothrops_dabieshanensis:0.089789):0.028843,Protobothrops_cornutus:0.118632):0.025257):0.042039,Protobothrops_mangshanensis:0.185928):0.026276,((Protobothrops_himalayanus:0.079082,Protobothrops_kaulbacki:0.079082):0.074663,Protobothrops_sieversorum:0.153745):0.058459):0.036404,(((Ovophis_convictus:0.129411,Ovophis_monticola:0.129411):0.043336,Ovophis_zayuensis:0.172747):0.010407,(Ovophis_makazayazaya:0.137348,Ovophis_tonkinensis:0.137348):0.045806):0.065454):0.030122,((((((Gloydius_monticola:0.067493,Gloydius_huangi:0.067493):0.01537,Gloydius_rubromaculatus:0.082863):0.016442,(Gloydius_angusticep:0.078024,Gloydius_strauchi:0.078024):0.021281):0.012634,(Gloydius_liupanensis:0.092725,Gloydius_qinlinensis:0.092725):0.019214):0.024424,((Gloydius_blomhoffi:0.045877,Gloydius_tsushimaensis:0.045877):0.053494,(Gloydius_brevicaudus:0.018939,Gloydius_ussuriensis:0.018939):0.080432):0.036992):0.008804,((((Gloydius_intermedius:0.016254,Gloydius_shedaoensis:0.016254):0.018075,Gloydius_halys:0.034328):0.004653,Gloydius_stejnegeri:0.038982):0.007399,Gloydius_cognatus:0.046381):0.098785996981144):0.133563):0.021975,((((((((((Bothrops_asper:0.088421,Bothrops_brazili:0.088421):0.035317,(Bothrops_erythromelas:0.091421,Bothrops_jararaca:0.091421):0.032318):0.011829,Bothrops_chloromelas:0.135568):0.022821,Bothrops_ammodytoides:0.158388):0.018907,Bothrops_pictus:0.177296):0.060457,((Atropoides_picadoi:0.143439,Cerrophidion_tzotzilorum:0.143439):0.04255,(Porthidium_ophryomegas:0.124876,Porthidium_yucatanicum:0.124876):0.061113):0.051763):0.013191,(((Mixcoatlus_browni:0.111687,Mixcoatlus_melanurus:0.111687):0.015369,Mixcoatlus_barbouri:0.127056):0.051709,Ophryacus_undulatus:0.178765):0.072178):0.014011,((Bothriechis_lateralis:0.136536,Bothriechis_nigroviridis:0.136536):0.081809,Bothriechis_schlegelii:0.218346):0.046609):0.011645,((((((Crotalus_basiliscus:0.110042,Crotalus_pricei:0.110042):0.023255,(Crotalus_enyo:0.101112,Crotalus_willardi:0.101112):0.032185):0.01599,(Crotalus_adamanteus:0.114667,Crotalus_ruber:0.114667):0.03462):0.022214,Crotalus_cerastes:0.1715):0.026574,(Sistrurus_catenatus:0.146901,Sistrurus_miliarius:0.146901):0.051174):0.060429,(Agkistrodon_contortrix:0.144917,Agkistrodon_piscivorus:0.144917):0.113587):0.018096):0.007024,Lachesis_acrochorda:0.283624):0.01708):0.006967,(Ovophis_okinavensis:0.140521,Trimeresurus_gracilis:0.140521):0.16715):0.014563,((((((((((((Trimeresurus_erythrurus:0.017731,Trimeresurus_purpureomaculatus:0.017731):0.010122,Trimeresurus_cantori:0.027852):0.009196,Trimeresurus_andersonii:0.037049):0.029261,Trimeresurus_albolabris:0.06631):0.015621,Trimeresurus_septentrionalis:0.08193):0.015616,(Trimeresurus_fasciatus:0.048057,Trimeresurus_insularis:0.048057):0.049489):0.068704,((Trimeresurus_macrops:0.050043,Trimeresurus_venustus:0.050043):0.025566,Trimeresurus_kanburiensis:0.075609):0.090641):0.018822,(((((Trimeresurus_gumprechti:0.053008,Trimeresurus_stejnegeri:0.053008):0.029021,Trimeresurus_truongsonensis:0.082029):0.011609,Trimere

surus_vogeli:0.093639):0.023666,Trimeresurus_yunnanensis:0.117304):0.009162,Trimeresurus_
medoensis:0.126466):0.058606):0.00961,Trimeresurus_sichuanensis:0.194682):0.015301,((((Trim
eresurus_flavomaculatus:0.02389,Trimeresurus_mcgregori:0.02389):0.083327,(Trimeresurus_sch
ultzei:0.08427,Trimeresurus_sumatranus:0.08427):0.022947):0.01919,Trimeresurus_malcolmi:0.1
26406):0.039775,Trimeresurus_hageni:0.166181):0.043802):0.016792,((Trimeresurus_popeiorum:
0.074359,Trimeresurus_sabahi:0.074359):0.110693,Trimeresurus_tibetanus:0.185051):0.041724):
0.055712,(((Trimeresurus_borneensis:0.126783,Trimeresurus_wiroti:0.126783):0.058094,Trimere
surus_puniceus:0.184877):0.051404,((Trimeresurus_gramineus:0.09954,Trimeresurus_trigonocep
halus:0.09954):0.057734,Trimeresurus_malabaricus:0.157274):0.079007):0.046206):0.039747):0.
047264,((((Hypnale_hypnale:0.124461,Hypnale_zara:0.124461):0.051554,Hypnale_nepa:0.17601
6):0.086055,Calloselasma_rhodostoma:0.26207):0.090027,(((Tropidolaemus_subannulatus:0.181
675,Tropidolaemus_wagleri:0.181675):0.116911,Deinagkistrodon_acutus:0.298586):0.026586,Ga
rthius_chaseni:0.325172):0.026925):0.017401):0.044439,Azemiops_feae:0.413937):0.076838,((((
((((((((((Vipera_dinniki:0.004376,Vipera_renardi:0.004376):0.010058,Vipera_lotievi:0.014433):0.
014156,Vipera_eriwanensis:0.02859):0.019292,Vipera_ursinii:0.047882):0.009325,Vipera_orlovi:
0.057206):0.021506,(Vipera_anatolica:0.061283,Vipera_kaznakovi:0.061283):0.01743):0.023703,
(((Vipera_berus:0.017635,Vipera_barani:0.017635):0.0151,Vipera_nikolskii:0.032736):0.032401,
Vipera_seoanei:0.065137):0.037279):0.05538,(Vipera_aspis:0.085084,Vipera_latastei:0.085084):
0.072711):0.034899,(Vipera_ammodytes:0.077665,Vipera_transcaucasiana:0.077665):0.115029):
0.081541,((Daboia_mauritanica:0.153101001127243,Daboia_palaestinae:0.153101):0.055699,Da
boia_siamensis:0.2088):0.065435):0.028376,(((((Montivipera_albizona:0.031112,Montivipera_wa
gneri:0.031112):0.009687,Montivipera_bornmuelleri:0.040798):0.023297,Montivipera_xanthina:0.
064095):0.03855,(Montivipera_latifii:0.02616,Montivipera_raddei:0.02616):0.076485):0.067347,
(Macrovipera_lebetina:0.035178,Macrovipera_schweizeri:0.035178):0.134815):0.132619):0.0363
68,(((Pseudocerastes_persicus:0.10702,Pseudocerastes_urarachnoides:0.10702):0.058245,Pseudoc
erastes_fieldi:0.165265):0.075247,Eristicophis_macmahoni:0.240512):0.098467):0.065213,(((((((
Echis_leucogaster:0.033682,Echis_pyramidum:0.033682):0.062176,Echis_borkini:0.095858):0.02
4117,Echis_khosatzkii:0.119975):0.099633,(Echis_coloratus:0.103214,Echis_omanensis:0.10321
4):0.116393):0.027481,(Echis_jogeri:0.096743,Echis_ocellatus:0.096743):0.150345):0.016766,Ec
his_carinatus:0.263854):0.120908,((((Causus_resimus:0.142287,Causus_rhombeatus:0.142287):0.
019133,Causus_defilippii:0.16142):0.05938,Causus_lichtensteini:0.220801):0.124122,((Cerastes_
cerastes:0.13493,Cerastes_gasperettii:0.13493):0.078572,Cerastes_vipera:0.213501):0.131421):0.
039839):0.019431):0.012482,((((((((Bitis_cornuta:0.069743,Bitis_rubida:0.069743):0.049242,Biti
s_atropos:0.118985):0.050894,Bitis_xeropaga:0.16988):0.061481,(Bitis_caudalis:0.08039,Bitis_p
eringueyi:0.08039):0.150971):0.043845,((Bitis_gabonica:0.032677,Bitis_rhinoceros:0.032677):0.
090733,Bitis_nasicornis:0.12341):0.151796):0.021336,Bitis_worthingtoni:0.296541):0.03016,Biti
s_arietans:0.326701):0.060597,((((Atheris_nitschei:0.122891,Atheris_desaixi:0.122891):0.03023,(
Atheris_squamigera:0.060738,Atheris_hispida:0.060738):0.092383):0.022425,((Atheris_ceratoph
ora:0.03995,Atheris_matildae:0.03995):0.091442,Atheris_barbouri:0.131392):0.044154):0.03002
4,Atheris_chlorechis:0.20557):0.181728):0.029377):0.012456,Proatheris_superciliaris:0.42913):0.
061644);

Data S2.

Densities for calibrated nodes from **Table S5**. "Prior" mean**s** the marginal prior, and "Posterior" means the marginal posterior. For the majority of calibrated nodes, the incomplete overlap of prior and posterior distributions suggested the data contain sufficient information to overrule the priors for these parameters.

Agamidae

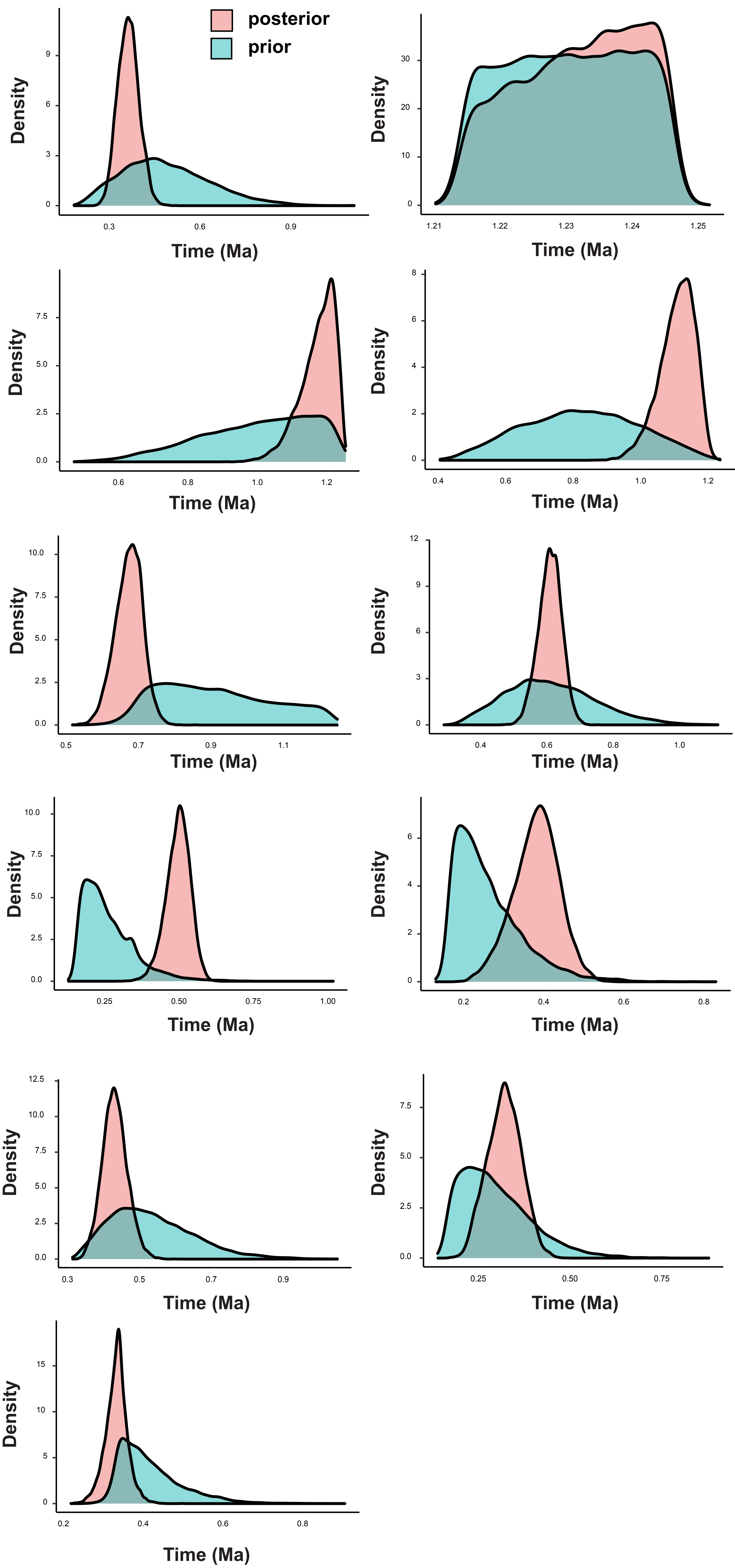

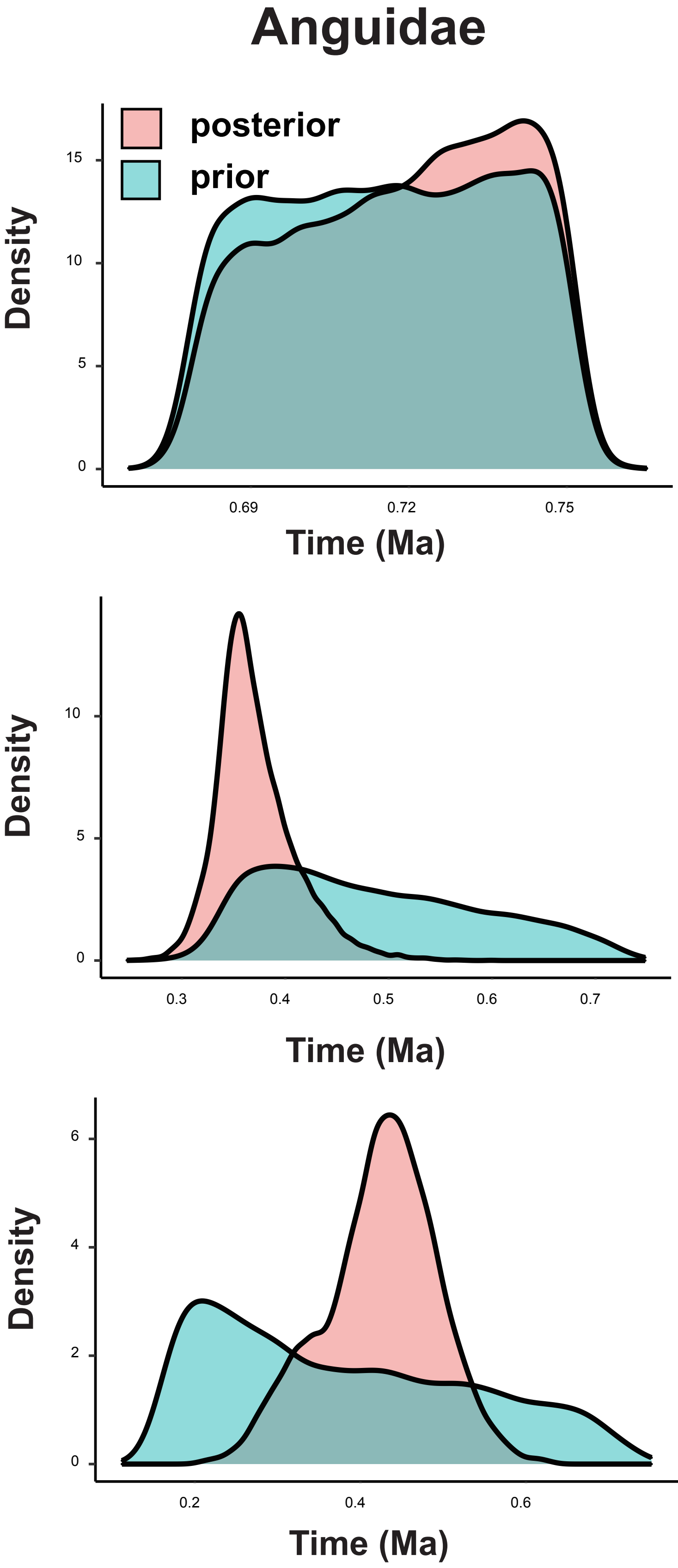
Anguidae
posterior
prior
Density
15
10
5
0
0.69
0.72
0.75
Time (Ma)
Density
10
5
0
0.3
0.4
0.5
0.6
0.7
Time (Ma)
Density
6
4
2
0
0.2
0.4
0.6
Time (Ma)

Bufonidae

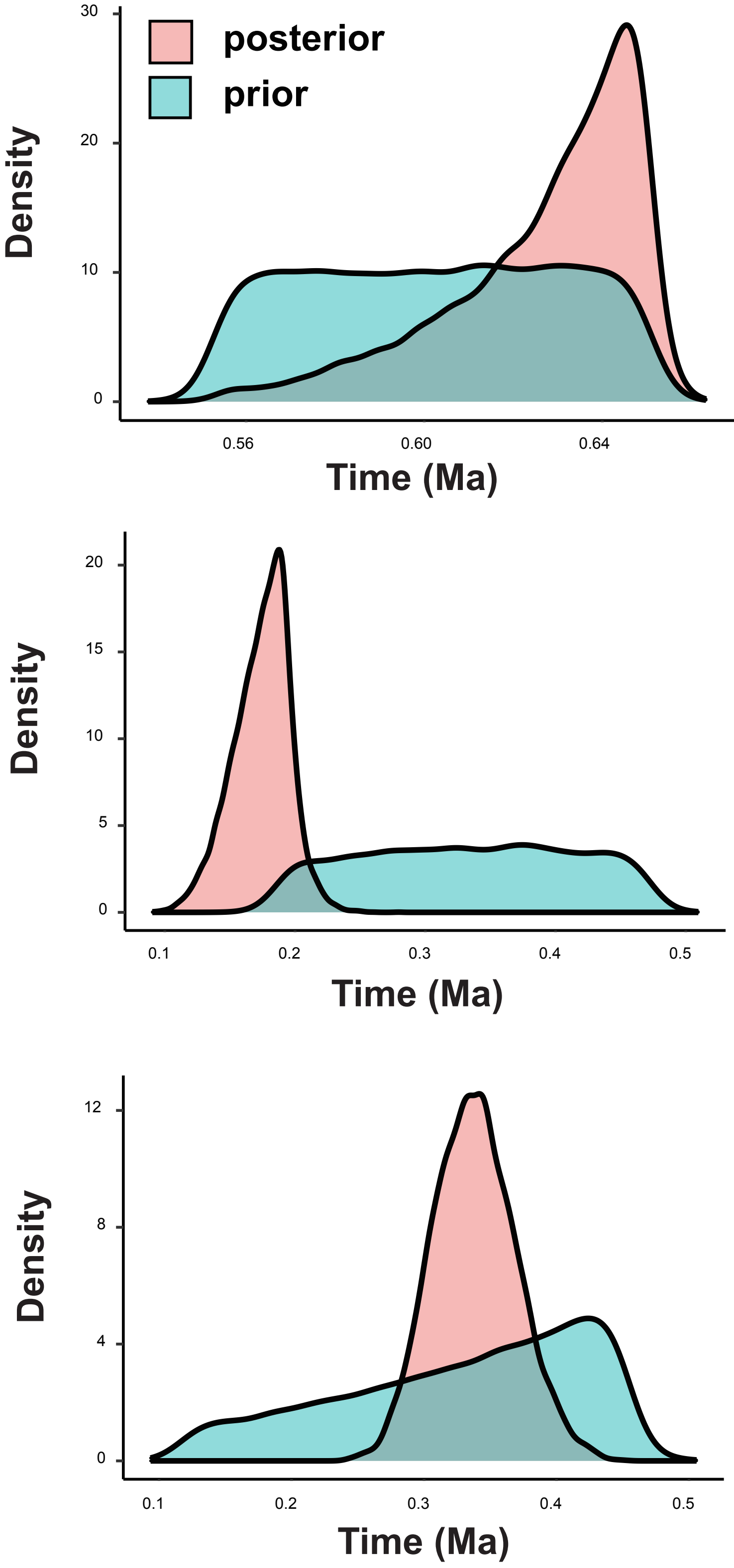

posterior
prior
30
20
10
0
Density
0.56
0.60
0.64
Time (Ma)
20
15
10
5
0
Density
0.1
0.2
0.3
0.4
0.5
Time (Ma)
12
8
4
0
Density
0.1
0.2
0.3
0.4
0.5
Time (Ma)

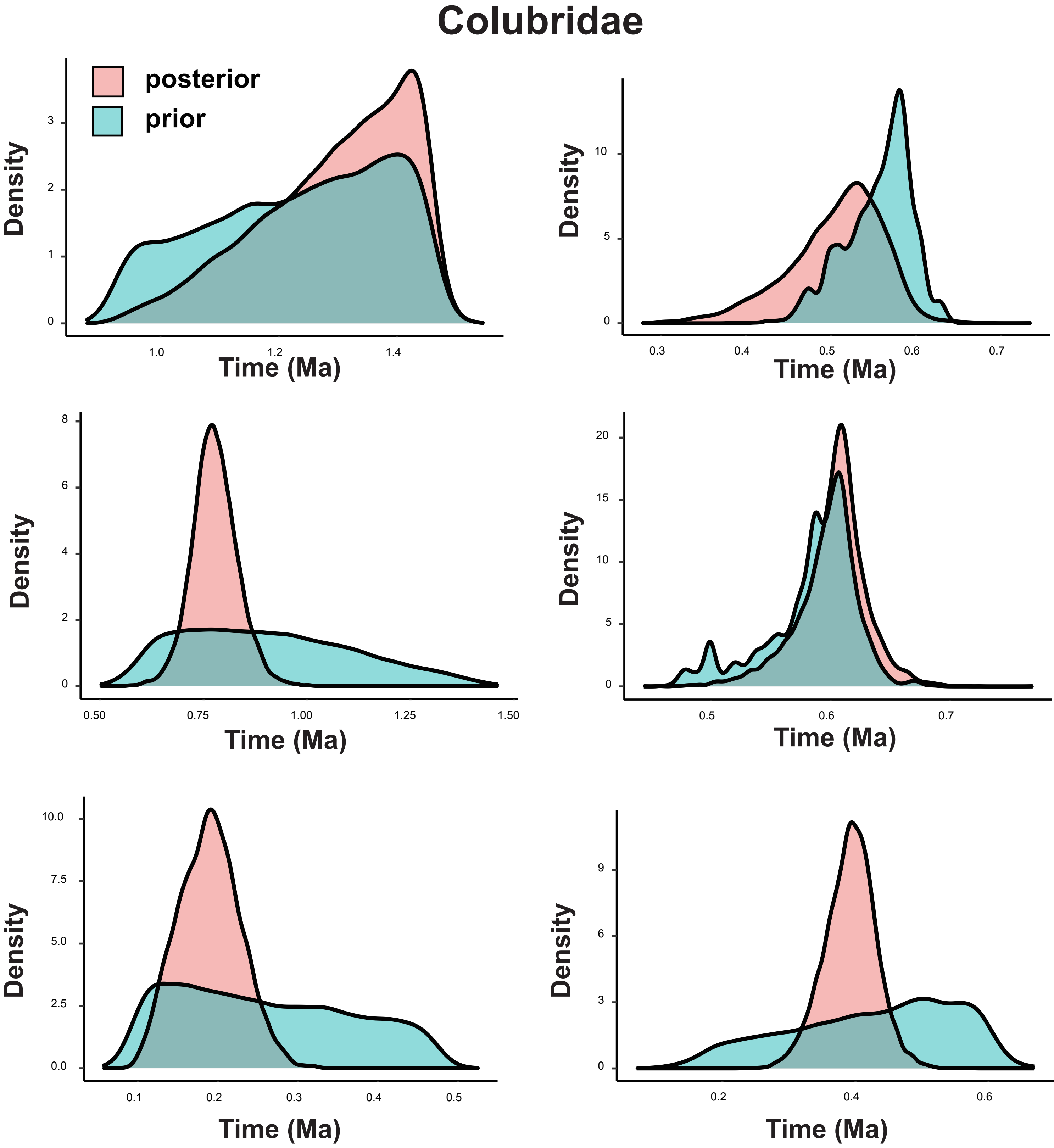

Colubridae
posterior
prior
Density
Time (Ma)
0
1
2
3
1.0
1.2
1.4
0
5
10
0.3
0.4
0.5
0.6
0.7
0
2
4
6
8
0.50
0.75
1.00
1.25
1.50
0
5
10
15
20
0.5
0.6
0.7
0.0
2.5
5.0
7.5
10.0
0.1
0.2
0.3
0.4
0.5
0
3
6
9
0.2
0.4
0.6

Dicroglossidae

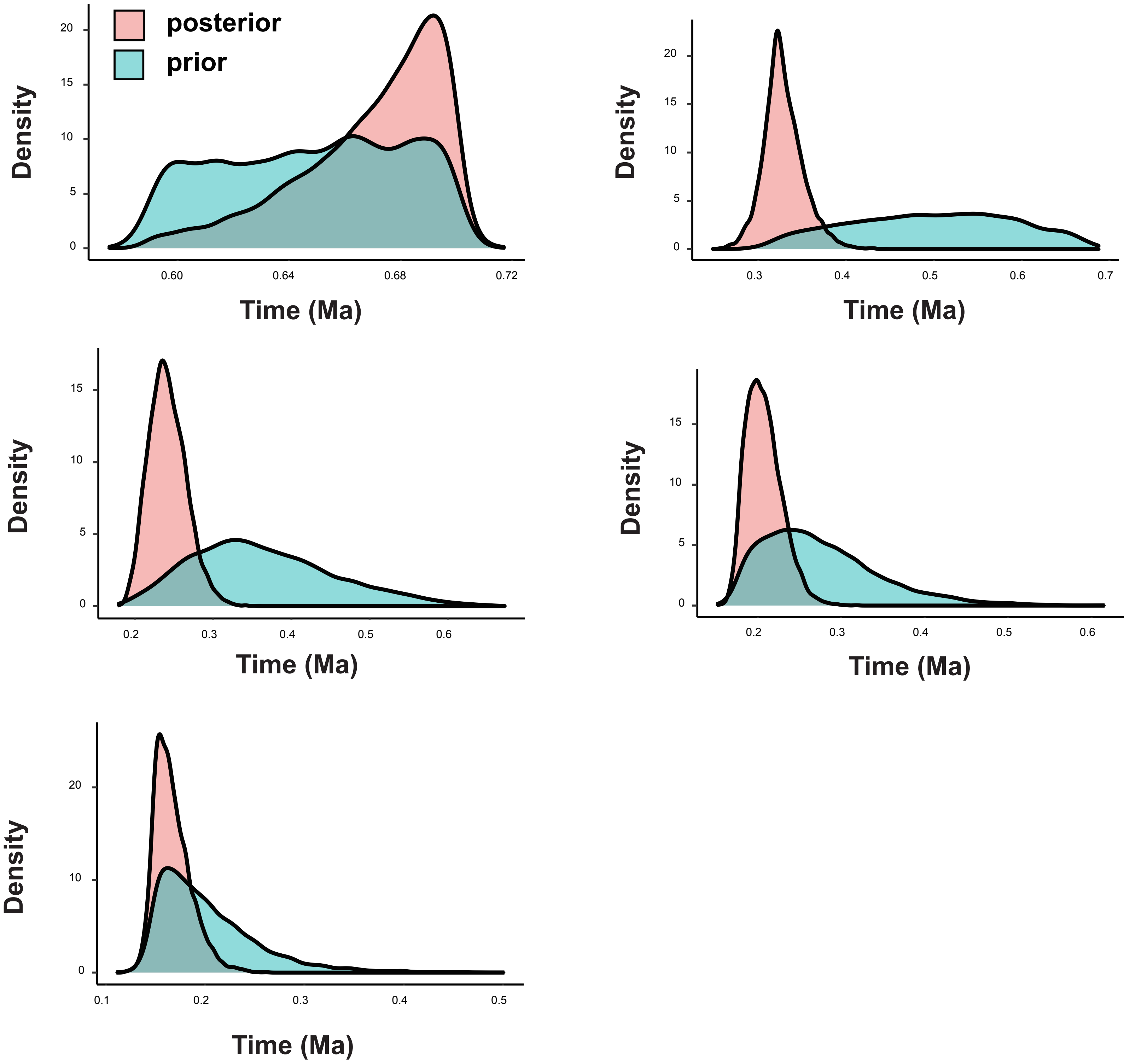

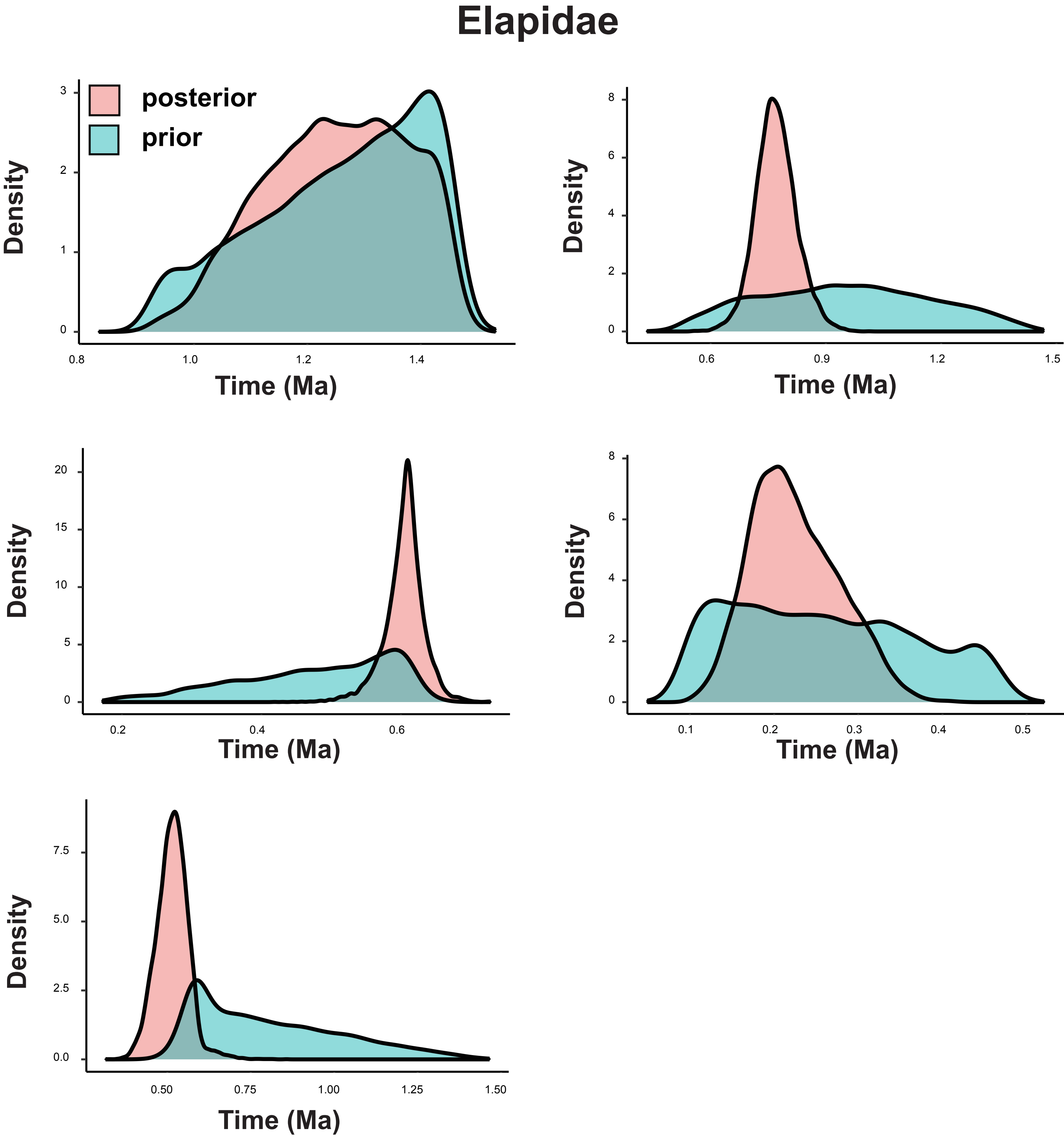

Elapidae
posterior
prior
Density
Time (Ma)

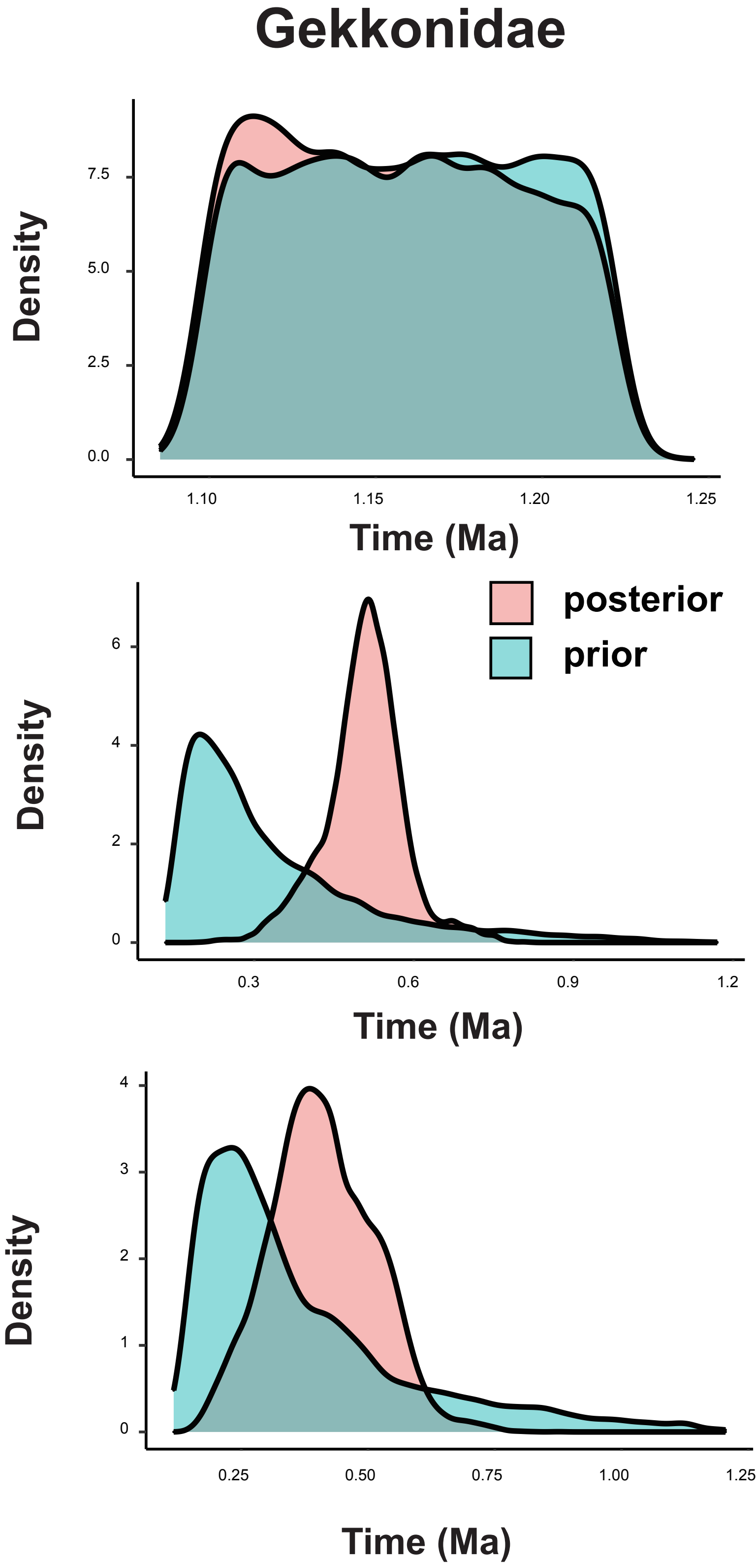

Gekkonidae
Density
7.5
5.0
2.5
0.0
1.10
1.15
1.20
1.25
Time (Ma)
posterior
prior
Density
6
4
2
0
0.3
0.6
0.9
1.2
Time (Ma)
Density
4
3
2
1
0
0.25
0.50
0.75
1.00
1.25
Time (Ma)

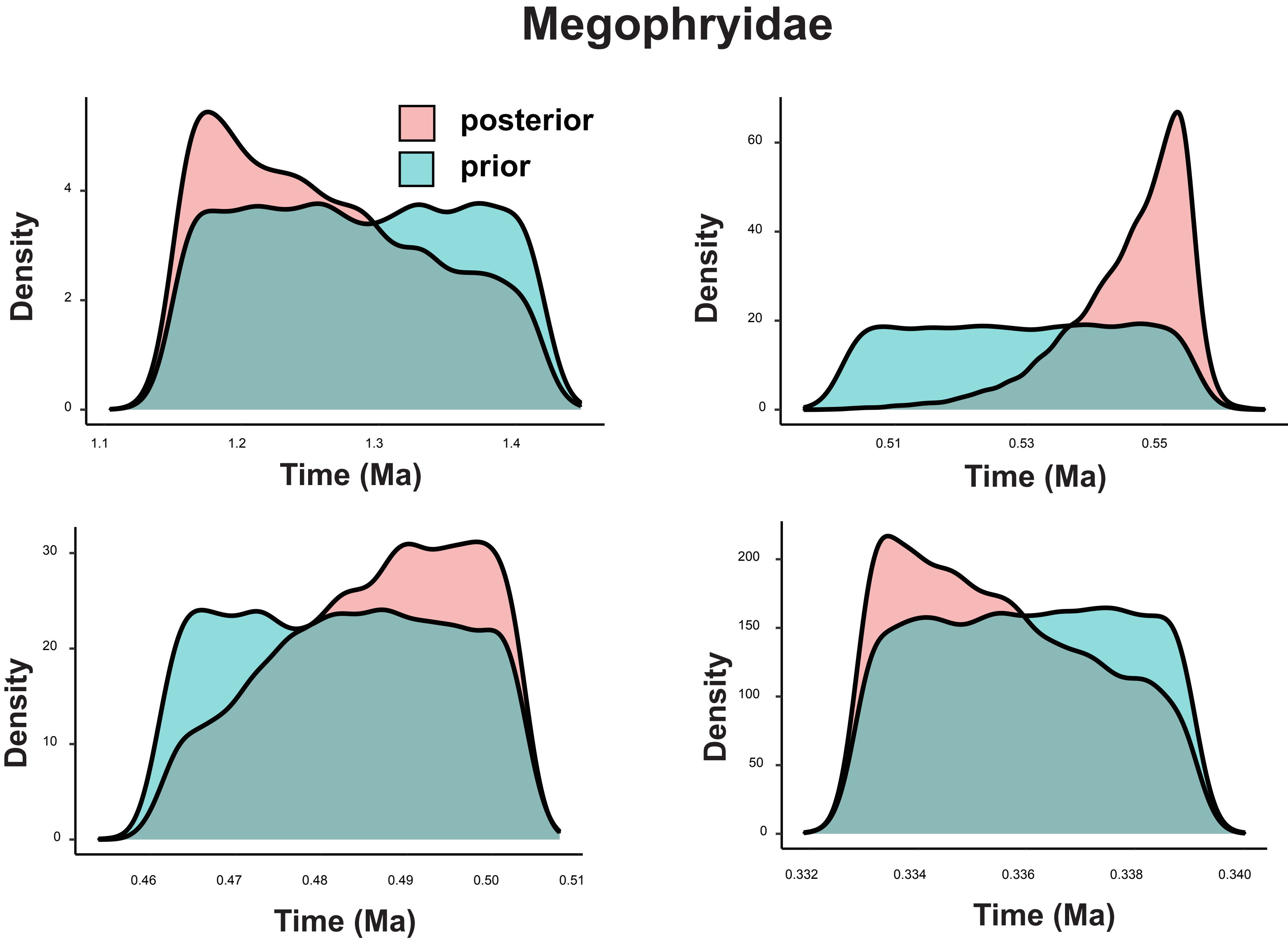

Megophryidae
posterior
prior
Density
Time (Ma)
0
2
4
1.1
1.2
1.3
1.4
0
20
40
60
0.51
0.53
0.55
0
10
20
30
0.46
0.47
0.48
0.49
0.50
0.51
0
50
100
150
200
0.332
0.334
0.336
0.338
0.340

Pareidae

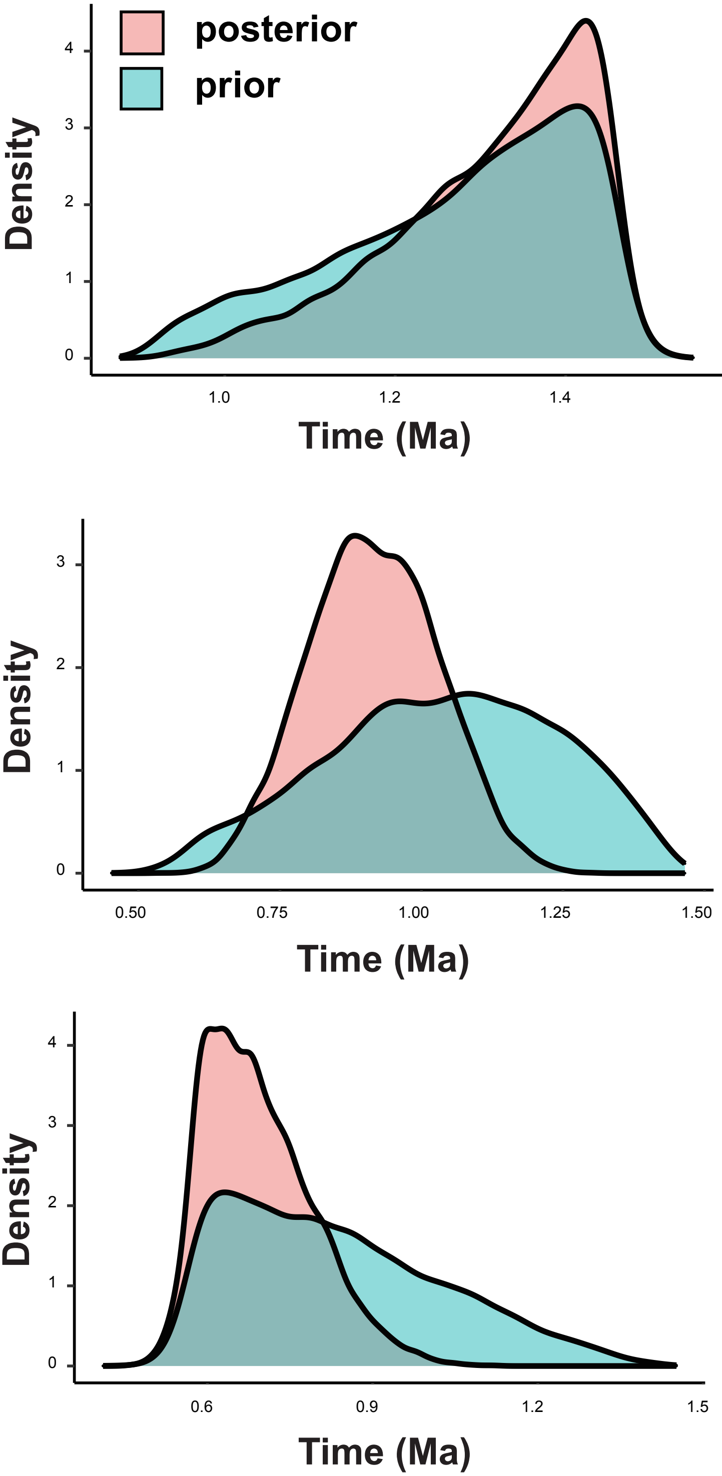
posterior
prior
Density
0
1
2
3
4
1.0
1.2
1.4
Time (Ma)
Density
0
1
2
3
0.50
0.75
1.00
1.25
1.50
Time (Ma)
Density
0
1
2
3
4
0.6
0.9
1.2
1.5
Time (Ma)

Ranidae

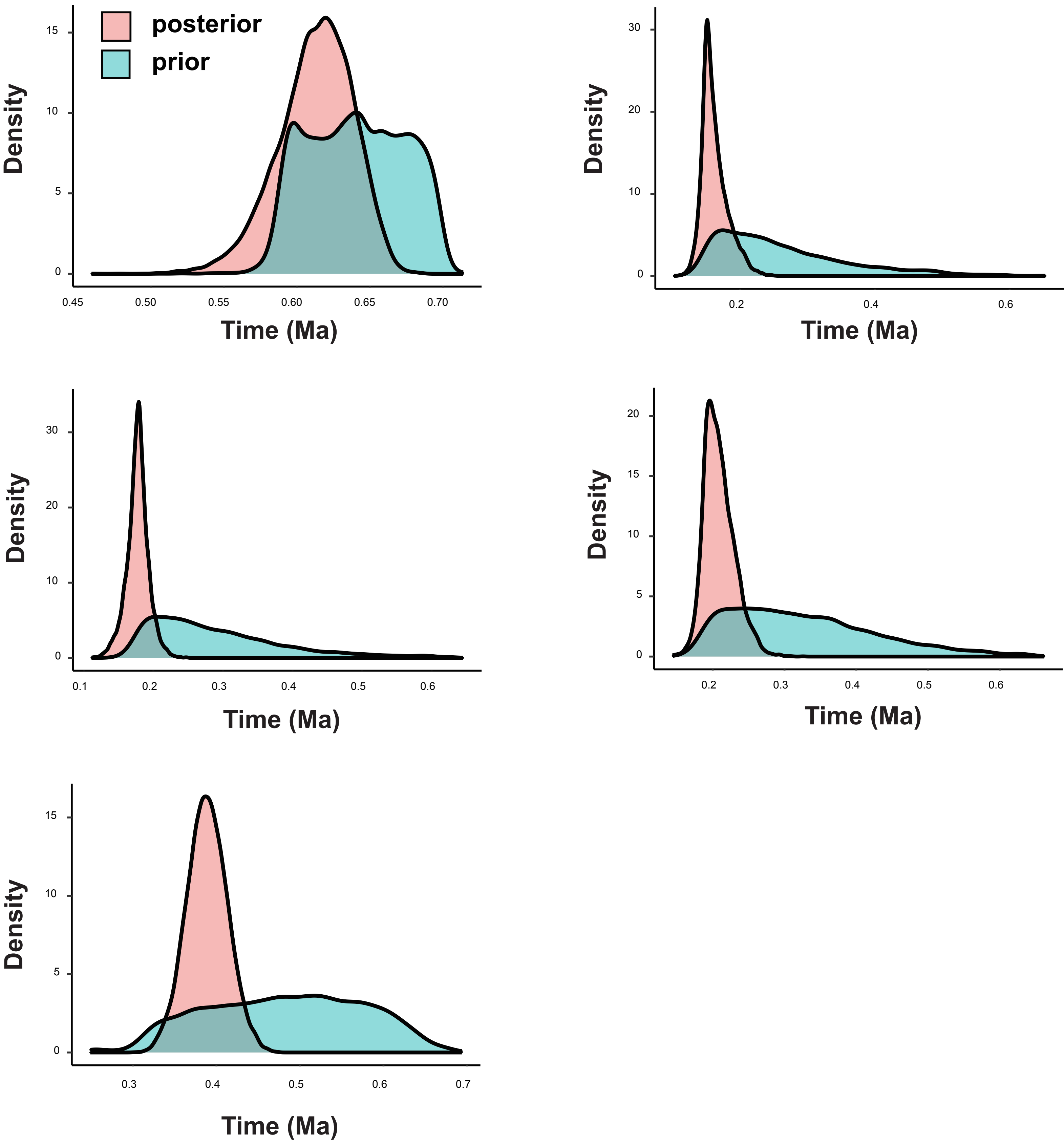
posterior
prior
Density
Time (Ma)
0
5
10
15
20
30
0.45
0.50
0.55
0.60
0.65
0.70
0.1
0.2
0.3
0.4
0.5
0.6
0.7

Scincidae

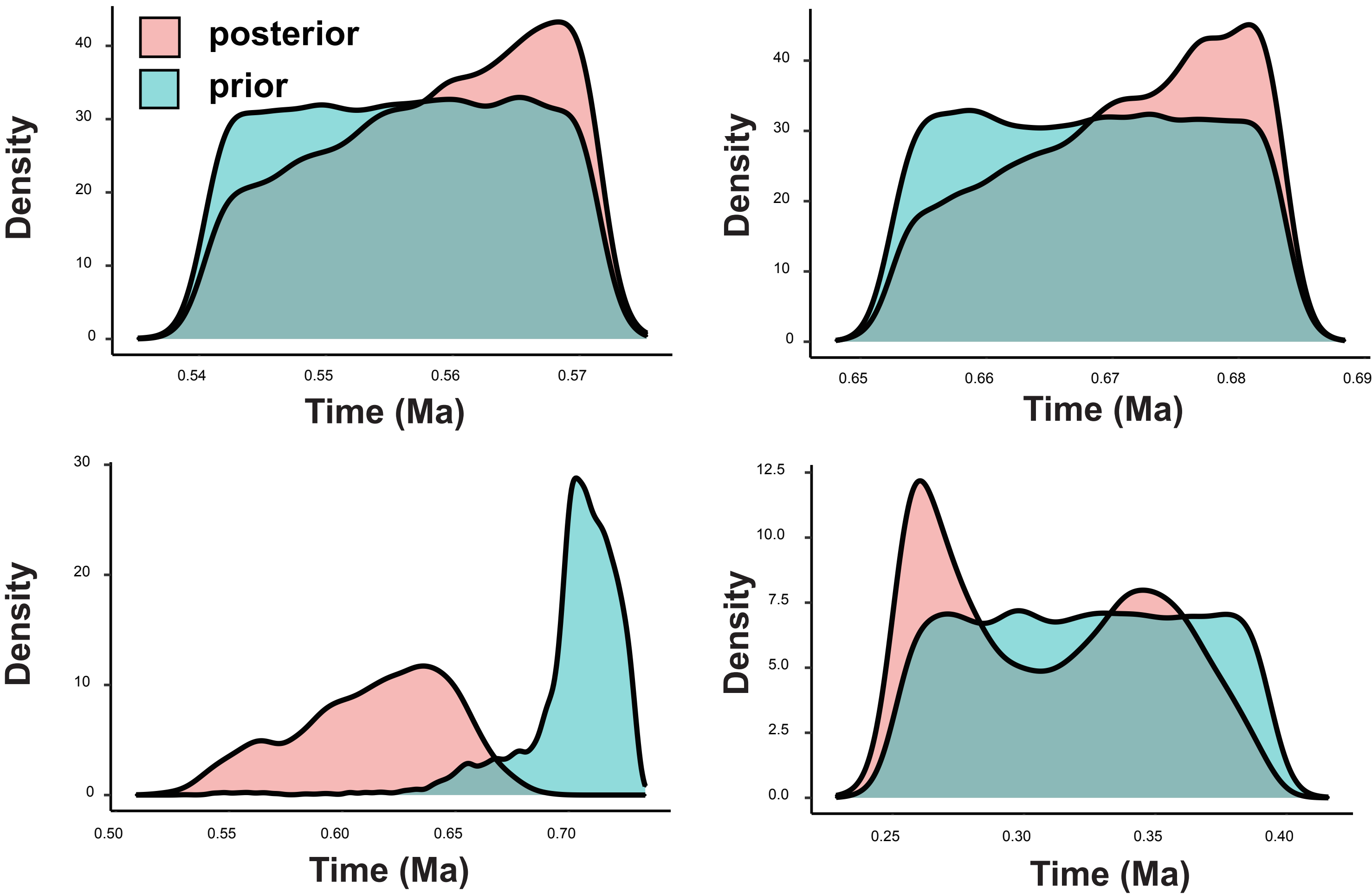

posterior
prior
Density
Time (Ma)
0
10
20
30
40
0.54
0.55
0.56
0.57
0.65
0.66
0.67
0.68
0.69
0.50
0.55
0.60
0.65
0.70
0.0
2.5
5.0
7.5
10.0
12.5
0.25
0.30
0.35
0.40

Rhacophoridae

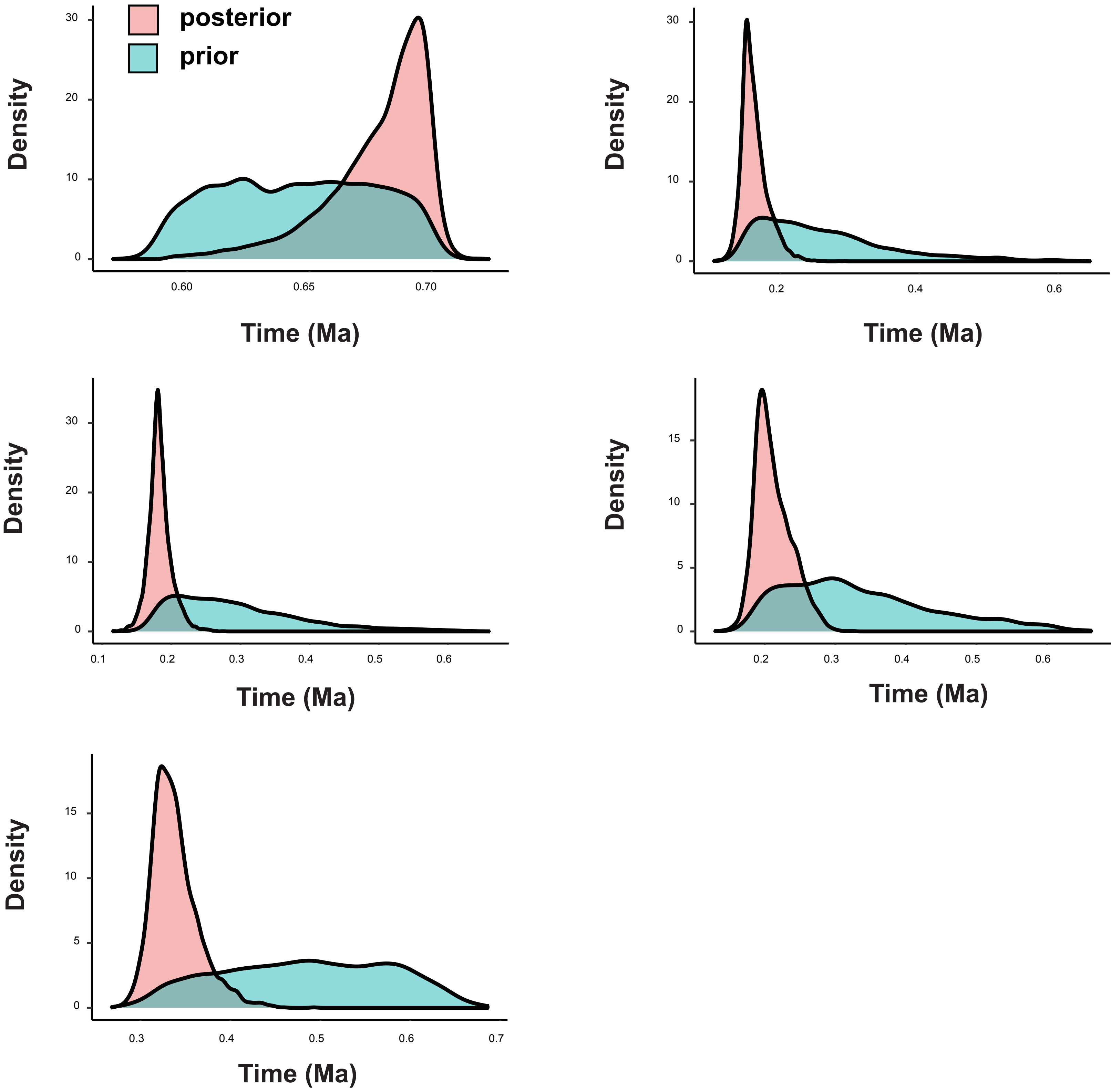

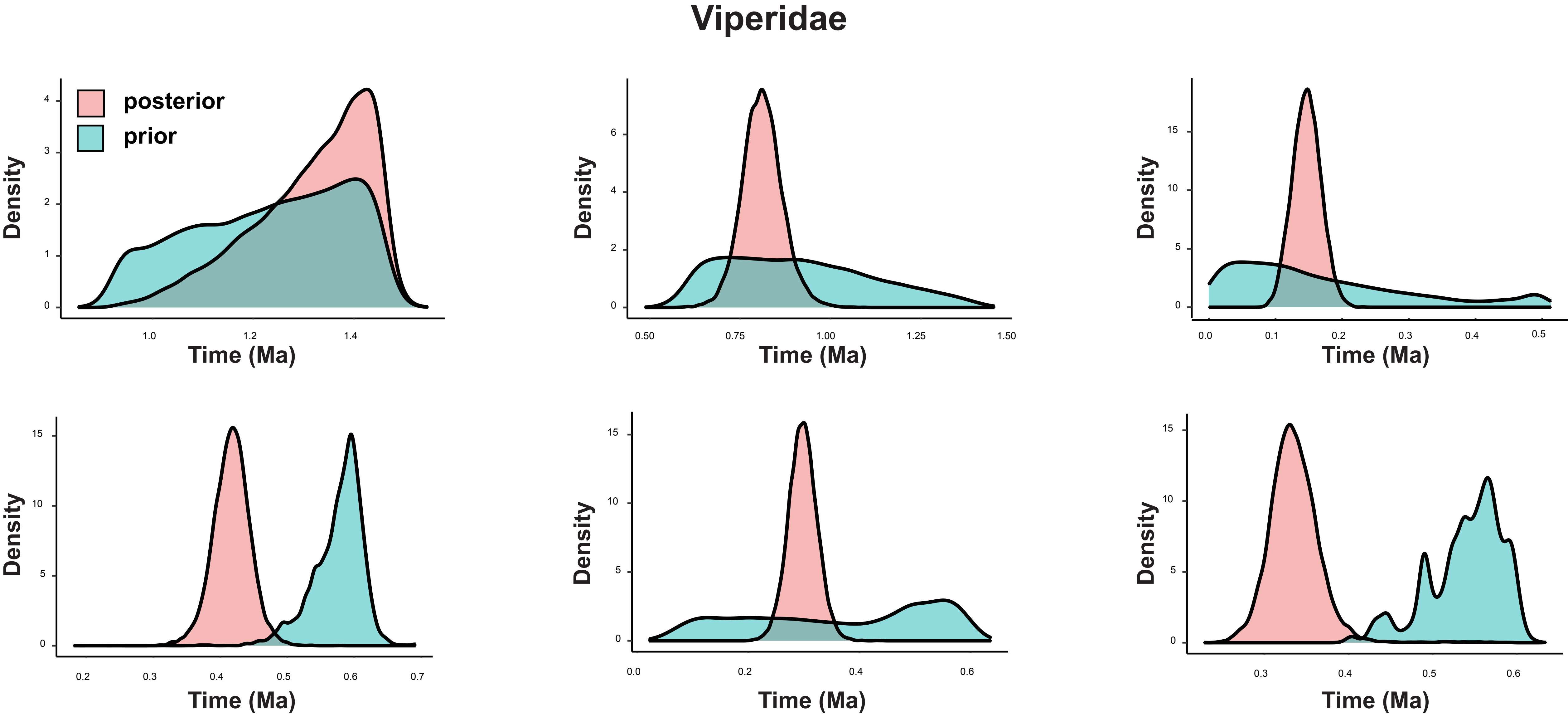

Viperidae
posterior
prior
Density
Time (Ma)

Ceratobatrachidae

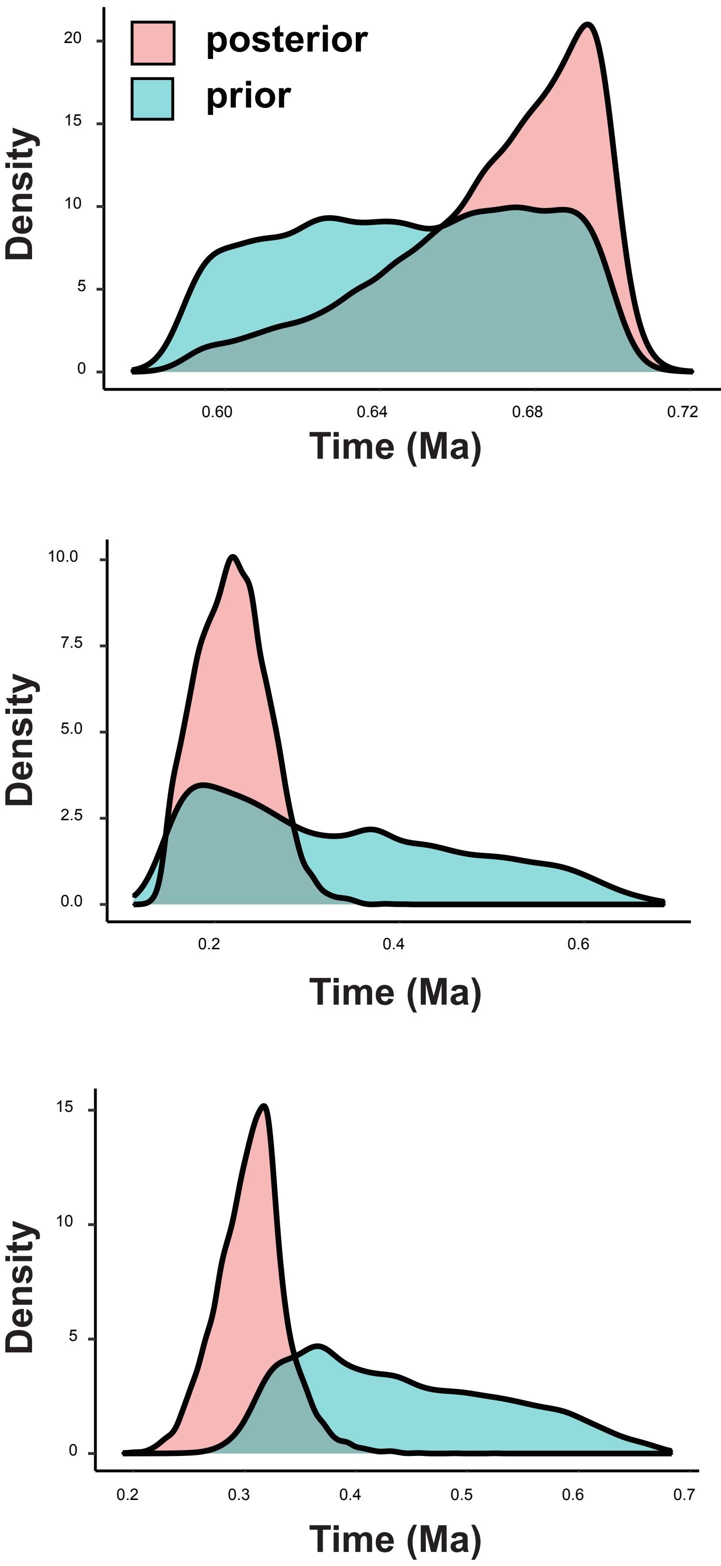

Data S3.

BioGeoBEARS analysis results based on two regions. Ancestral areas for reconstructed nodes are indicated by the following colors and letters: Red (A): the Himalaya; blue (B): non-Himalayan regions; black (AB): both regions; Red branches represent *in situ* diversification events, blue branches represent dispersal events, and gray branches were ambiguous. Black branches are not related to Himalayan diversification.

Agamidae

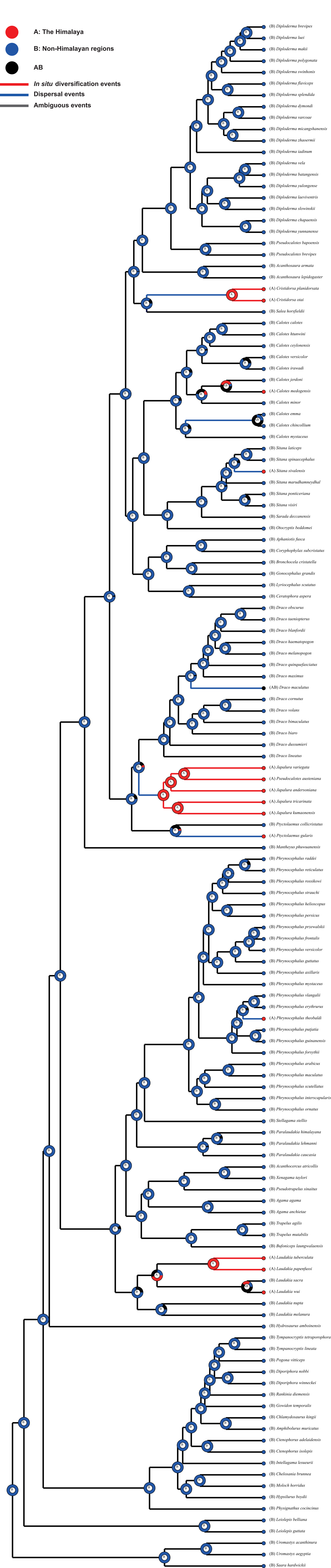
A: The Himalaya
B: Non-Himalayan regions
AB
In situ diversification events
Dispersal events
Ambiguous events
(B) Diploderma brevipes
(B) Diploderma luei
(B) Diploderma makii
(B) Diploderma polygonata
(B) Diploderma swinhonis
(B) Diploderma flaviceps
(B) Diploderma splendida
(B) Diploderma dymondi
(B) Diploderma varcoae
(B) Diploderma micangshanensis
(B) Diploderma zhaoermii
(B) Diploderma iadinum
(B) Diploderma vela
(B) Diploderma batangensis
(B) Diploderma yulongense
(B) Diploderma laeviventris
(B) Diploderma slowinskii
(B) Diploderma chapaensis
(B) Diploderma yunnanense
(B) Pseudocalotes bapoensis
(B) Pseudocalotes brevipes
(B) Acanthosaura armata
(B) Acanthosaura lepidogaster
(A) Cristidorsa planidorsata
(A) Cristidorsa otai
(B) Salea horsfieldii
(B) Calotes calotes
(B) Calotes htunwini
(B) Calotes ceylonensis
(B) Calotes versicolor
(B) Calotes irawadi
(B) Calotes jerdoni
(A) Calotes medogensis
(B) Calotes minor
(B) Calotes emma
(B) Calotes chincollium
(B) Calotes mystaceus
(B) Sitana laticeps
(B) Sitana spinaecephalus
(A) Sitana sivalensis
(B) Sitana marudhamneydhal
(B) Sitana ponticeriana
(B) Sitana visiri
(B) Sarada deccanensis
(B) Otocryptis beddomei
(B) Aphaniotis fusca
(B) Coryphophylax subcristatus
(B) Bronchocela cristatella
(B) Gonocephalus grandis
(B) Lyriocephalus scutatus
(B) Ceratophora aspera
(B) Draco obscurus
(B) Draco taeniopterus
(B) Draco blanfordii
(B) Draco haematopogon
(B) Draco melanopogon
(B) Draco quinquefasciatus
(B) Draco maximus
(AB) Draco maculatus
(B) Draco cornutus
(B) Draco volans
(B) Draco bimaculatus
(B) Draco biaro
(B) Draco dussumieri
(B) Draco lineatus
(A) Japalura variegata
(A) Pseudocalotes austeniana
(A) Japalura andersoniana
(A) Japalura tricarinata
(A) Japalura kumaonensis
(B) Ptyctolaemus collicristatus
(A) Ptyctolaemus gularis
(B) Mantheyus phuwuanensis
(B) Phrynocephalus raddei
(B) Phrynocephalus reticulatus
(B) Phrynocephalus rossikowi
(B) Phrynocephalus strauchi
(B) Phrynocephalus helioscopus
(B) Phrynocephalus persicus
(B) Phrynocephalus przewalskii
(B) Phrynocephalus frontalis
(B) Phrynocephalus versicolor
(B) Phrynocephalus guttatus
(B) Phrynocephalus axillaris
(B) Phrynocephalus mystaceus
(B) Phrynocephalus vlangalii
(B) Phrynocephalus erythrurus
(A) Phrynocephalus theobaldi
(B) Phrynocephalus putjatia
(B) Phrynocephalus guinanensis
(B) Phrynocephalus forsythii
(B) Phrynocephalus arabicus
(B) Phrynocephalus maculatus
(B) Phrynocephalus scutellatus
(B) Phrynocephalus interscapularis
(B) Phrynocephalus ornatus
(B) Stellagama stellio
(B) Paralaudakia himalayana
(B) Paralaudakia lehmanni
(B) Paralaudakia caucasia
(B) Acanthocercus atricollis
(B) Xenagama taylori
(B) Pseudotrapelus sinaitus
(B) Agama agama
(B) Agama anchietae
(B) Trapelus agilis
(B) Trapelus mutabilis
(B) Bufoniceps laungwalaensis
(A) Laudakia tuberculata
(A) Laudakia papenfussi
(B) Laudakia sacra
(A) Laudakia wui
(B) Laudakia nupta
(B) Laudakia melanura
(B) Hydrosaurus amboinensis
(B) Tympanocryptis tetraporophora
(B) Tympanocryptis lineata
(B) Pogona vitticeps
(B) Diporiphora nobbi
(B) Diporiphora winneckei
(B) Rankinia diemensis
(B) Gowidon temporalis
(B) Chlamydosaurus kingii
(B) Amphibolurus muricatus
(B) Ctenophorus adelaidensis
(B) Ctenophorus isolepis
(B) Intellagama lesueurii
(B) Chelosania brunnea
(B) Moloch horridus
(B) Hypsilurus boydii
(B) Physignathus cocincinus
(B) Leiolepis belliana
(B) Leiolepis guttata
(B) Uromastyx acanthinura
(B) Uromastyx aegyptia
(B) Saara hardwickii

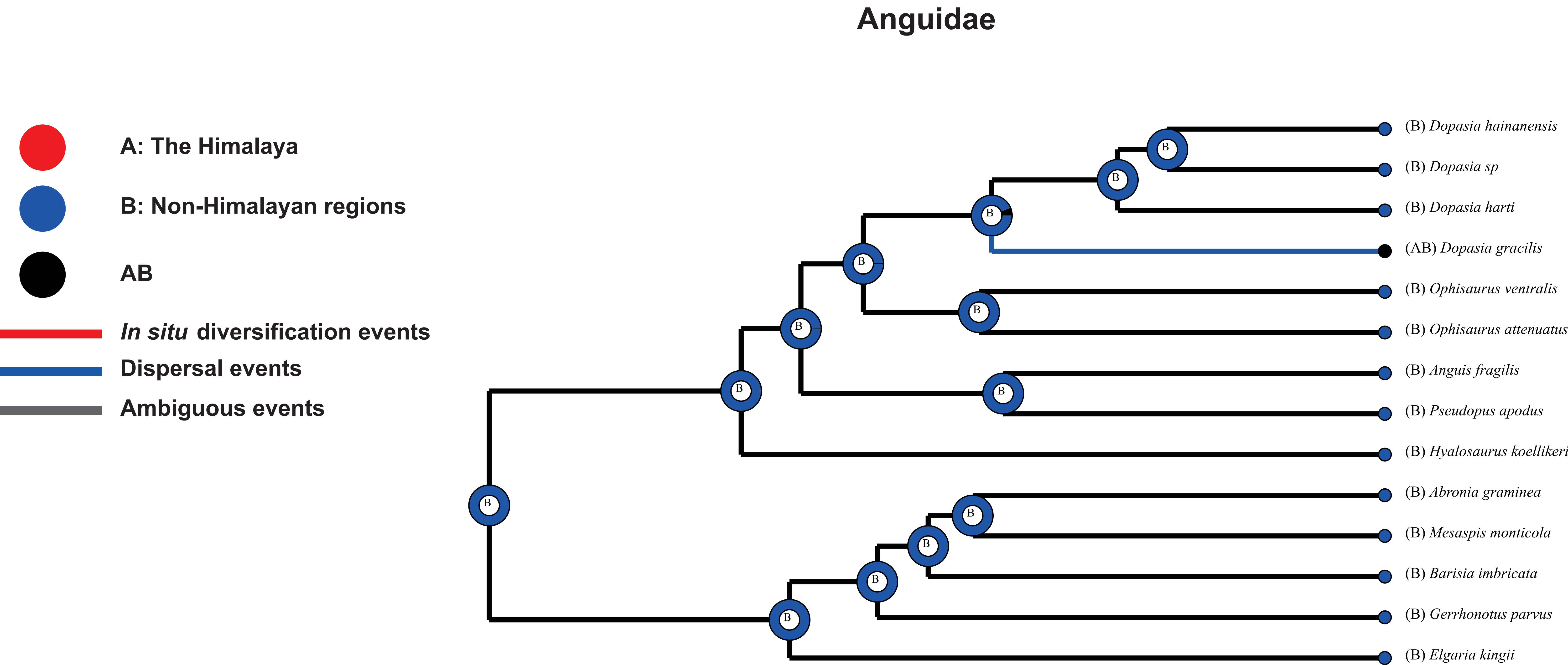

Anguidae
A: The Himalaya
B: Non-Himalayan regions
AB
In situ diversification events
Dispersal events
Ambiguous events
(B) *Dopasia hainanensis*
(B) *Dopasia sp*
(B) *Dopasia harti*
(AB) *Dopasia gracilis*
(B) *Ophisaurus ventralis*
(B) *Ophisaurus attenuatus*
(B) *Anguis fragilis*
(B) *Pseudopus apodus*
(B) *Hyalosaurus koellikeri*
(B) *Abronia graminea*
(B) *Mesaspis monticola*
(B) *Barisia imbricata*
(B) *Gerrhonotus parvus*
(B) *Elgaria kingii*

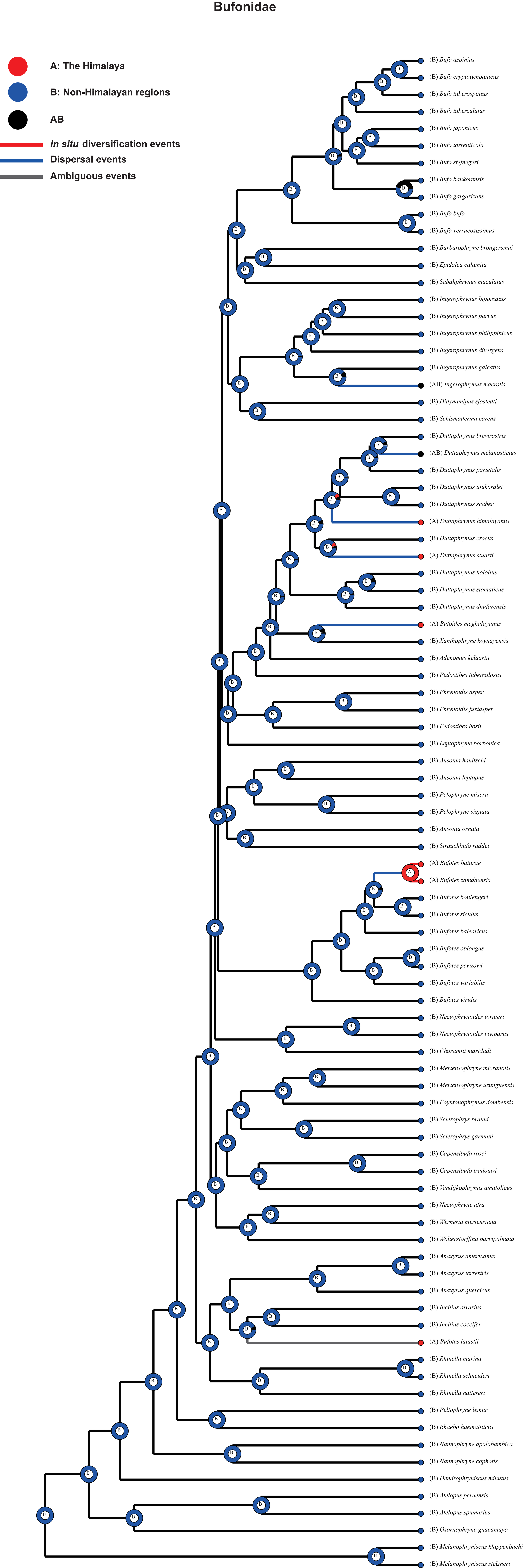
Bufonidae
A: The Himalaya
B: Non-Himalayan regions
AB
In situ diversification events
Dispersal events
Ambiguous events
(B) Bufo aspinius
(B) Bufo cryptotympanicus
(B) Bufo tuberospinius
(B) Bufo tuberculatus
(B) Bufo japonicus
(B) Bufo torrenticola
(B) Bufo stejnegeri
(B) Bufo bankorensis
(B) Bufo gargarizans
(B) Bufo bufo
(B) Bufo verrucosissimus
(B) Barbarophryne brongersmai
(B) Epidalea calamita
(B) Sabahphrynus maculatus
(B) Ingerophrynus biporcatus
(B) Ingerophrynus parvus
(B) Ingerophrynus philippinicus
(B) Ingerophrynus divergens
(B) Ingerophrynus galeatus
(AB) Ingerophrynus macrotis
(B) Didynamipus sjostedti
(B) Schismaderma carens
(B) Duttaphrynus brevirostris
(AB) Duttaphrynus melanostictus
(B) Duttaphrynus parietalis
(B) Duttaphrynus atukoralei
(B) Duttaphrynus scaber
(A) Duttaphrynus himalayanus
(B) Duttaphrynus crocus
(A) Duttaphrynus stuarti
(B) Duttaphrynus hololius
(B) Duttaphrynus stomaticus
(B) Duttaphrynus dhufarensis
(A) Bufoides meghalayanus
(B) Xanthophryne koynayensis
(B) Adenomus kelaartii
(B) Pedostibes tuberculosus
(B) Phrynoidis asper
(B) Phrynoidis juxtasper
(B) Pedostibes hosii
(B) Leptophryne borbonica
(B) Ansonia hanitschi
(B) Ansonia leptopus
(B) Pelophryne misera
(B) Pelophryne signata
(B) Ansonia ornata
(B) Strauchbufo raddei
(A) Bufotes baturae
(A) Bufotes zamdaensis
(B) Bufotes boulengeri
(B) Bufotes siculus
(B) Bufotes balearicus
(B) Bufotes oblongus
(B) Bufotes pewzowi
(B) Bufotes variabilis
(B) Bufotes viridis
(B) Nectophrynoides tornieri
(B) Nectophrynoides viviparus
(B) Churamiti maridadi
(B) Mertensophryne micranotis
(B) Mertensophryne uzunguensis
(B) Poyntonophrynus dombensis
(B) Sclerophrys brauni
(B) Sclerophrys garmani
(B) Capensibufo rosei
(B) Capensibufo tradouwi
(B) Vandijkophrynus amatolicus
(B) Nectophryne afra
(B) Werneria mertensiana
(B) Wolterstorffina parvipalmata
(B) Anaxyrus americanus
(B) Anaxyrus terrestris
(B) Anaxyrus quercicus
(B) Incilius alvarius
(B) Incilius coccifer
(A) Bufotes latastii
(B) Rhinella marina
(B) Rhinella schneideri
(B) Rhinella nattereri
(B) Peltophryne lemur
(B) Rhaebo haematiticus
(B) Nannophryne apolobambica
(B) Nannophryne cophotis
(B) Dendrophryniscus minutus
(B) Atelopus peruensis
(B) Atelopus spumarius
(B) Osornophryne guacamayo
(B) Melanophryniscus klappenbachi
(B) Melanophryniscus stelzneri

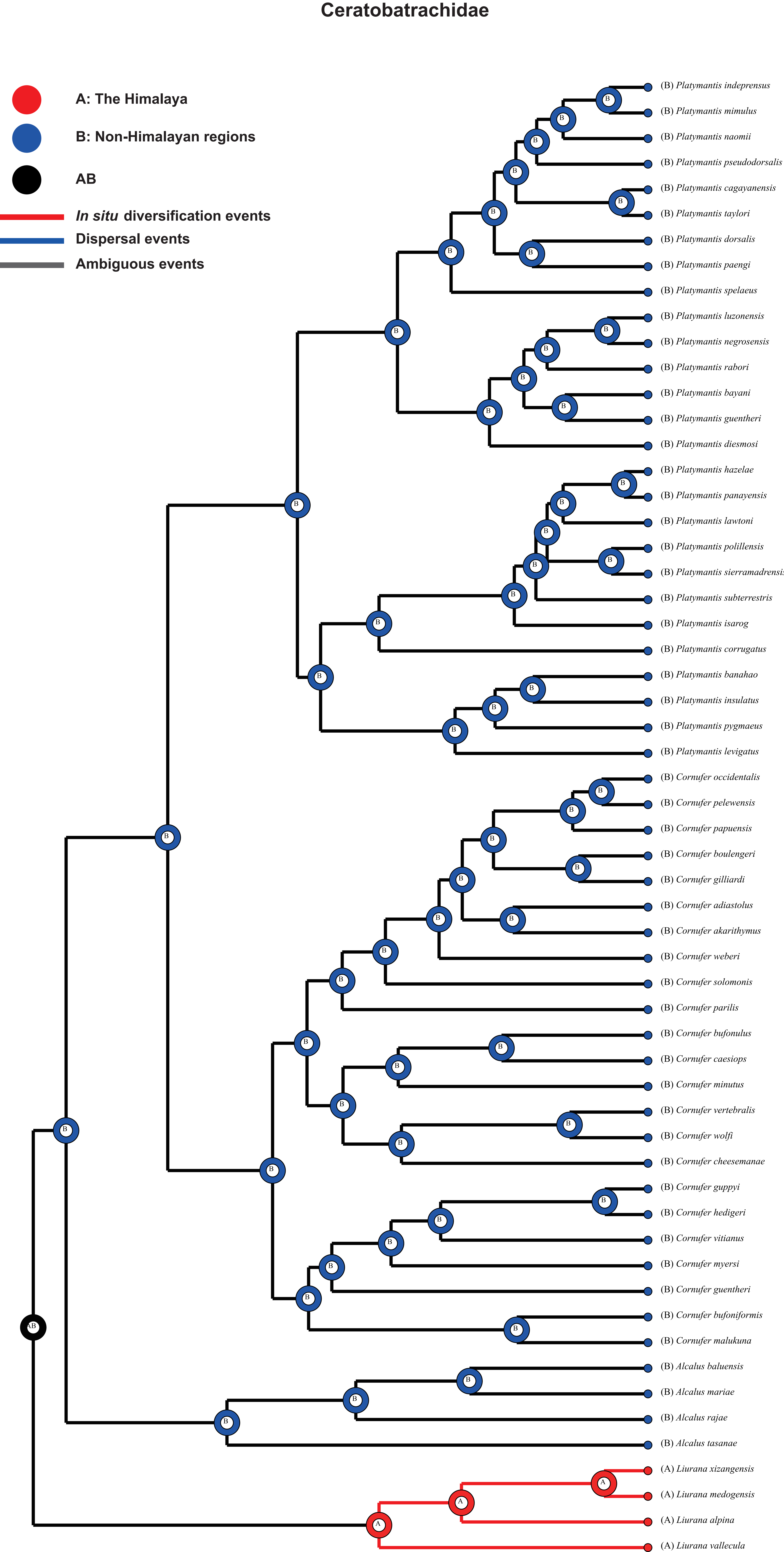
Ceratobatrachidae
A: The Himalaya
B: Non-Himalayan regions
AB
In situ diversification events
Dispersal events
Ambiguous events
(B) Platymantis indeprensus
(B) Platymantis mimulus
(B) Platymantis naomii
(B) Platymantis pseudodorsalis
(B) Platymantis cagayanensis
(B) Platymantis taylori
(B) Platymantis dorsalis
(B) Platymantis paengi
(B) Platymantis spelaeus
(B) Platymantis luzonensis
(B) Platymantis negrosensis
(B) Platymantis rabori
(B) Platymantis bayani
(B) Platymantis guentheri
(B) Platymantis diesmosi
(B) Platymantis hazelae
(B) Platymantis panayensis
(B) Platymantis lawtoni
(B) Platymantis polillensis
(B) Platymantis sierramadrensis
(B) Platymantis subterrestris
(B) Platymantis isarog
(B) Platymantis corrugatus
(B) Platymantis banahao
(B) Platymantis insulatus
(B) Platymantis pygmaeus
(B) Platymantis levigatus
(B) Cornufer occidentalis
(B) Cornufer pelewensis
(B) Cornufer papuensis
(B) Cornufer boulengeri
(B) Cornufer gilliardi
(B) Cornufer adiastolus
(B) Cornufer akarithymus
(B) Cornufer weberi
(B) Cornufer solomonis
(B) Cornufer parilis
(B) Cornufer bufonulus
(B) Cornufer caesiops
(B) Cornufer minutus
(B) Cornufer vertebralis
(B) Cornufer wolfi
(B) Cornufer cheesemanae
(B) Cornufer guppyi
(B) Cornufer hedigeri
(B) Cornufer vitianus
(B) Cornufer myersi
(B) Cornufer guentheri
(B) Cornufer bufoniformis
(B) Cornufer malukuna
(B) Alcalus baluensis
(B) Alcalus mariae
(B) Alcalus rajae
(B) Alcalus tasanae
(A) Liurana xizangensis
(A) Liurana medogensis
(A) Liurana alpina
(A) Liurana vallecula

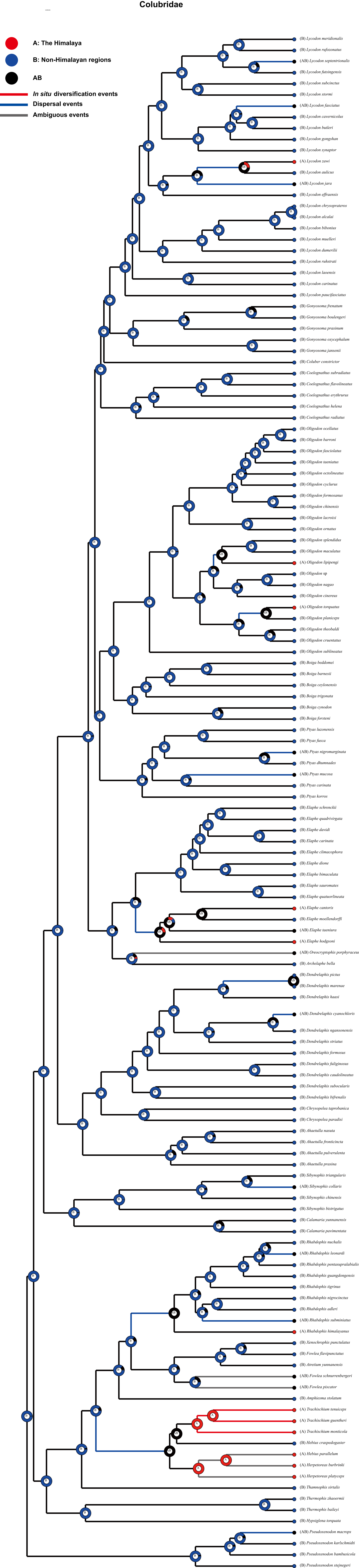

Colubridae
A: The Himalaya
B: Non-Himalayan regions
AB
In situ diversification events
Dispersal events
Ambiguous events
(B) *Lycodon meridionalis*
(B) *Lycodon rufozonatus*
(AB) *Lycodon septentrionalis*
(B) *Lycodon futsingensis*
(B) *Lycodon subcinctus*
(B) *Lycodon stormi*
(AB) *Lycodon fasciatus*
(B) *Lycodon cavernicolus*
(B) *Lycodon butleri*
(B) *Lycodon gongshan*
(B) *Lycodon synaptor*
(A) *Lycodon zawi*
(B) *Lycodon aulicus*
(AB) *Lycodon jara*
(B) *Lycodon effraensis*
(B) *Lycodon chrysoprateros*
(B) *Lycodon alcalai*
(B) *Lycodon bibonius*
(B) *Lycodon muelleri*
(B) *Lycodon dumerilii*
(B) *Lycodon ruhstrati*
(B) *Lycodon laoensis*
(B) *Lycodon carinatus*
(B) *Lycodon paucifasciatus*
(B) *Gonyosoma frenatum*
(B) *Gonyosoma boulengeri*
(B) *Gonyosoma prasinum*
(B) *Gonyosoma oxycephalum*
(B) *Gonyosoma jansenii*
(B) *Coluber constrictor*
(B) *Coelognathus subradiatus*
(B) *Coelognathus flavolineatus*
(B) *Coelognathus erythrurus*
(B) *Coelognathus helena*
(B) *Coelognathus radiatus*
(B) *Oligodon ocellatus*
(B) *Oligodon barroni*
(B) *Oligodon fasciolatus*
(B) *Oligodon taeniatus*
(B) *Oligodon octolineatus*
(B) *Oligodon cyclurus*
(B) *Oligodon formosanus*
(B) *Oligodon chinensis*
(B) *Oligodon lacroixi*
(B) *Oligodon ornatus*
(B) *Oligodon splendidus*
(B) *Oligodon maculatus*
(A) *Oligodon lipipengi*
(B) *Oligodon* sp
(B) *Oligodon nagao*
(B) *Oligodon cinereus*
(A) *Oligodon torquatus*
(B) *Oligodon planiceps*
(B) *Oligodon theobaldi*
(B) *Oligodon cruentatus*
(B) *Oligodon sublineatus*
(B) *Boiga beddomei*
(B) *Boiga barnesii*
(B) *Boiga ceylonensis*
(B) *Boiga trigonata*
(B) *Boiga cynodon*
(B) *Boiga forsteni*
(B) *Ptyas luzonensis*
(B) *Ptyas fusca*
(AB) *Ptyas nigromarginata*
(B) *Ptyas dhumnades*
(AB) *Ptyas mucosa*
(B) *Ptyas carinata*
(B) *Ptyas korros*
(B) *Elaphe schrenckii*
(B) *Elaphe quadrivirgata*
(B) *Elaphe davidi*
(B) *Elaphe carinata*
(B) *Elaphe climacophora*
(B) *Elaphe dione*
(B) *Elaphe bimaculata*
(B) *Elaphe sauromates*
(B) *Elaphe quatuorlineata*
(A) *Elaphe cantoris*
(B) *Elaphe moellendorffi*
(AB) *Elaphe taeniura*
(A) *Elaphe hodgsoni*
(AB) *Oreocryptophis porphyraceus*
(B) *Archelaphe bella*
(B) *Dendrelaphis pictus*
(B) *Dendrelaphis marenae*
(B) *Dendrelaphis haasi*
(AB) *Dendrelaphis cyanochloris*
(B) *Dendrelaphis ngansonensis*
(B) *Dendrelaphis striatus*
(B) *Dendrelaphis formosus*
(B) *Dendrelaphis fuliginosus*
(B) *Dendrelaphis caudolineatus*
(B) *Dendrelaphis subocularis*
(B) *Dendrelaphis bifrenalis*
(B) *Chrysopelea taprobanica*
(B) *Chrysopelea paradisi*
(B) *Ahaetulla nasuta*
(B) *Ahaetulla fronticincta*
(B) *Ahaetulla pulverulenta*
(B) *Ahaetulla prasina*
(B) *Sibynophis triangularis*
(AB) *Sibynophis collaris*
(B) *Sibynophis chinensis*
(B) *Sibynophis bistrigatus*
(B) *Calamaria yunnanensis*
(B) *Calamaria pavimentata*
(B) *Rhabdophis nuchalis*
(AB) *Rhabdophis leonardi*
(B) *Rhabdophis pentasupralabialis*
(B) *Rhabdophis guangdongensis*
(B) *Rhabdophis tigrinus*
(B) *Rhabdophis nigrocinctus*
(B) *Rhabdophis adleri*
(AB) *Rhabdophis subminiatus*
(A) *Rhabdophis himalayanus*
(B) *Xenochrophis punctulatus*
(B) *Fowlea flavipunctatus*
(B) *Atretium yunnanensis*
(AB) *Fowlea schnurrenbergeri*
(AB) *Fowlea piscator*
(B) *Amphiesma stolatum*
(A) *Trachischium tenuiceps*
(A) *Trachischium guentheri*
(A) *Trachischium monticola*
(B) *Hebius craspedogaster*
(A) *Hebius parallelum*
(A) *Herpetoreas burbrinki*
(A) *Herpetoreas platyceps*
(B) *Thamnophis sirtalis*
(B) *Thermophis zhaoermii*
(B) *Thermophis baileyi*
(B) *Hypsiglena torquata*
(AB) *Pseudoxenodon macrops*
(B) *Pseudoxenodon karlschmidti*
(B) *Pseudoxenodon bambusicola*
(B) *Pseudoxenodon stejnegeri*

Dicroglossidae

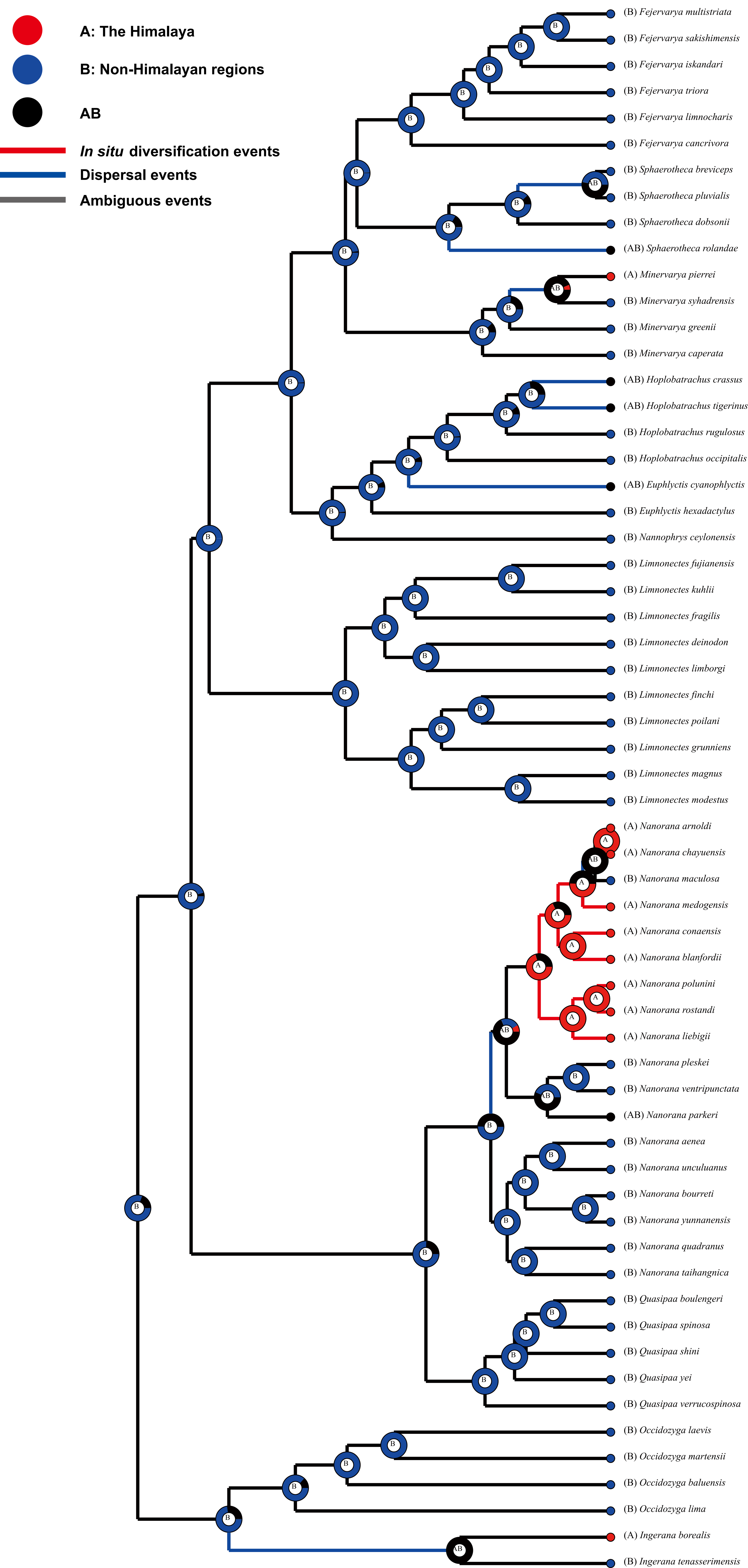

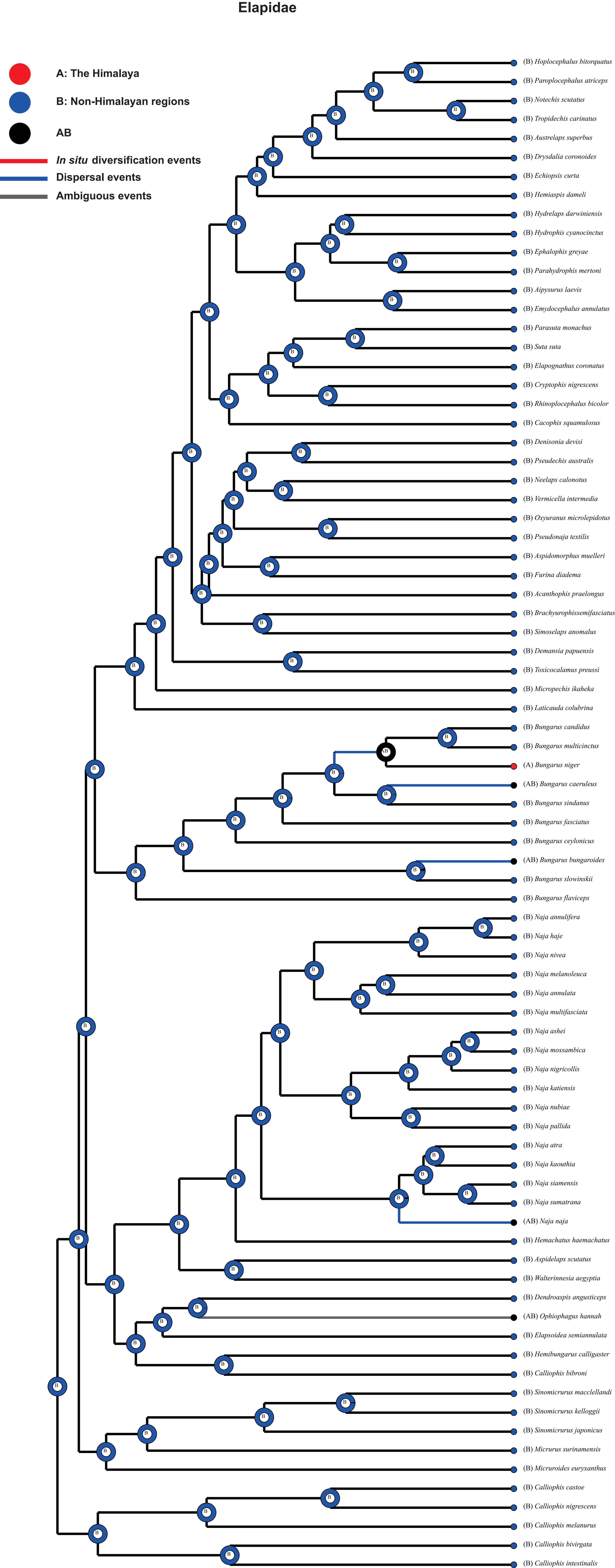

Elapidae
A: The Himalaya
B: Non-Himalayan regions
AB
In situ diversification events
Dispersal events
Ambiguous events
(B) Hoplocephalus bitorquatus
(B) Paroplocephalus atriceps
(B) Notechis scutatus
(B) Tropidechis carinatus
(B) Austrelaps superbus
(B) Drysdalia coronoides
(B) Echiopsis curta
(B) Hemiaspis dameli
(B) Hydrelaps darwiniensis
(B) Hydrophis cyanocinctus
(B) Ephalophis greyae
(B) Parahydrophis mertoni
(B) Aipysurus laevis
(B) Emydocephalus annulatus
(B) Parasuta monachus
(B) Suta suta
(B) Elapognathus coronatus
(B) Cryptophis nigrescens
(B) Rhinoplocephalus bicolor
(B) Cacophis squamulosus
(B) Denisonia devisi
(B) Pseudechis australis
(B) Neelaps calonotus
(B) Vermicella intermedia
(B) Oxyuranus microlepidotus
(B) Pseudonaja textilis
(B) Aspidomorphus muelleri
(B) Furina diadema
(B) Acanthophis praelongus
(B) Brachyurophissemifasciatus
(B) Simoselaps anomalus
(B) Demansia papuensis
(B) Toxicocalamus preussi
(B) Micropechis ikaheka
(B) Laticauda colubrina
(B) Bungarus candidus
(B) Bungarus multicinctus
(A) Bungarus niger
(AB) Bungarus caeruleus
(B) Bungarus sindanus
(B) Bungarus fasciatus
(B) Bungarus ceylonicus
(AB) Bungarus bungaroides
(B) Bungarus slowinskii
(B) Bungarus flaviceps
(B) Naja annulifera
(B) Naja haje
(B) Naja nivea
(B) Naja melanoleuca
(B) Naja annulata
(B) Naja multifasciata
(B) Naja ashei
(B) Naja mossambica
(B) Naja nigricollis
(B) Naja katiensis
(B) Naja nubiae
(B) Naja pallida
(B) Naja atra
(B) Naja kaouthia
(B) Naja siamensis
(B) Naja sumatrana
(AB) Naja naja
(B) Hemachatus haemachatus
(B) Aspidelaps scutatus
(B) Walterinnesia aegyptia
(B) Dendroaspis angusticeps
(AB) Ophiophagus hannah
(B) Elapsoidea semiannulata
(B) Hemibungarus calligaster
(B) Calliophis bibroni
(B) Sinomicrurus macclellandi
(B) Sinomicrurus kelloggii
(B) Sinomicrurus japonicus
(B) Micrurus surinamensis
(B) Micruroides euryxanthus
(B) Calliophis castoe
(B) Calliophis nigrescens
(B) Calliophis melanurus
(B) Calliophis bivirgata
(B) Calliophis intestinalis

Gekkonidae

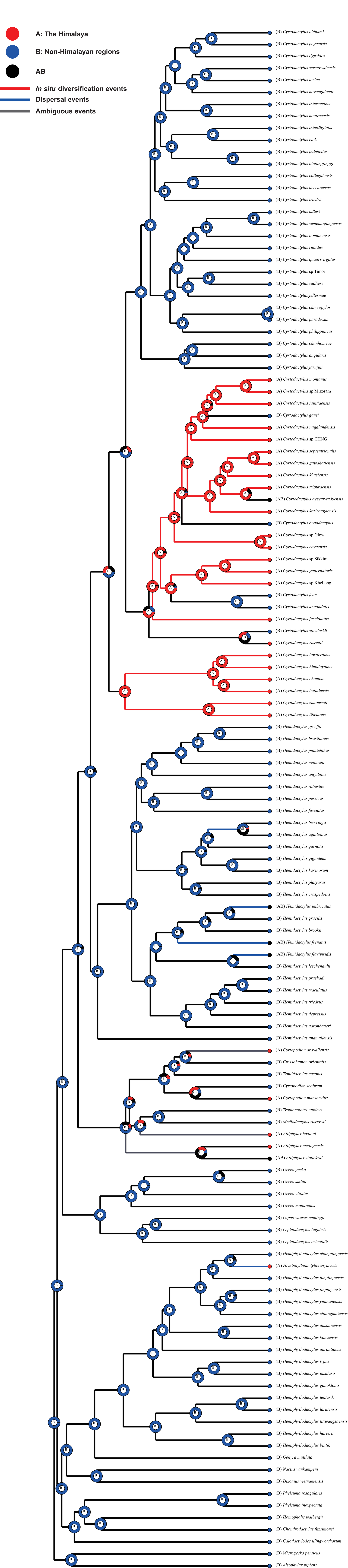
A: The Himalaya
B: Non-Himalayan regions
AB
In situ diversification events
Dispersal events
Ambiguous events
(B) Cyrtodactylus oldhami
(B) Cyrtodactylus peguensis
(B) Cyrtodactylus tigroides
(B) Cyrtodactylus sermowaiensis
(B) Cyrtodactylus loriae
(B) Cyrtodactylus novaeguineae
(B) Cyrtodactylus intermedius
(B) Cyrtodactylus hontreensis
(B) Cyrtodactylus interdigitalis
(B) Cyrtodactylus elok
(B) Cyrtodactylus pulchellus
(B) Cyrtodactylus bintangtinggi
(B) Cyrtodactylus collegalensis
(B) Cyrtodactylus deccanensis
(B) Cyrtodactylus triedra
(B) Cyrtodactylus adleri
(B) Cyrtodactylus semenanjungensis
(B) Cyrtodactylus tiomanensis
(B) Cyrtodactylus rubidus
(B) Cyrtodactylus quadrivirgatus
(B) Cyrtodactylus sp Timor
(B) Cyrtodactylus sadlieri
(B) Cyrtodactylus jellesmae
(B) Cyrtodactylus chrysopylos
(B) Cyrtodactylus paradoxus
(B) Cyrtodactylus philippinicus
(B) Cyrtodactylus chanhomeae
(B) Cyrtodactylus angularis
(B) Cyrtodactylus jarujini
(A) Cyrtodactylus montanus
(A) Cyrtodactylus sp Mizoram
(A) Cyrtodactylus jaintiaensis
(B) Cyrtodactylus gansi
(A) Cyrtodactylus nagalandensis
(A) Cyrtodactylus sp CHNG
(A) Cyrtodactylus septentrionalis
(A) Cyrtodactylus guwahatiensis
(A) Cyrtodactylus khasiensis
(A) Cyrtodactylus tripuraensis
(AB) Cyrtodactylus ayeyarwadyensis
(A) Cyrtodactylus kazirangaensis
(B) Cyrtodactylus brevidactylus
(A) Cyrtodactylus sp Glow
(A) Cyrtodactylus cayuensis
(A) Cyrtodactylus sp Sikkim
(A) Cyrtodactylus gubernatoris
(A) Cyrtodactylus sp Khellong
(B) Cyrtodactylus feae
(B) Cyrtodactylus annandalei
(A) Cyrtodactylus fasciolatus
(B) Cyrtodactylus slowinskii
(A) Cyrtodactylus russelli
(A) Cyrtodactylus lawderanus
(A) Cyrtodactylus himalayanus
(A) Cyrtodactylus chamba
(A) Cyrtodactylus battalensis
(A) Cyrtodactylus zhaoermii
(A) Cyrtodactylus tibetanus
(B) Hemidactylus greeffii
(B) Hemidactylus brasilianus
(B) Hemidactylus palaichthus
(B) Hemidactylus mabouia
(B) Hemidactylus angulatus
(B) Hemidactylus robustus
(B) Hemidactylus persicus
(B) Hemidactylus fasciatus
(B) Hemidactylus bowringii
(B) Hemidactylus aquilonius
(B) Hemidactylus garnotii
(B) Hemidactylus giganteus
(B) Hemidactylus karenorum
(B) Hemidactylus platyurus
(B) Hemidactylus craspedotus
(AB) Hemidactylus imbricatus
(B) Hemidactylus gracilis
(B) Hemidactylus brookii
(AB) Hemidactylus frenatus
(AB) Hemidactylus flaviviridis
(B) Hemidactylus leschenaulti
(B) Hemidactylus prashadi
(B) Hemidactylus maculatus
(B) Hemidactylus triedrus
(B) Hemidactylus depressus
(B) Hemidactylus aaronbaueri
(B) Hemidactylus anamallensis
(A) Cyrtopodion aravallensis
(B) Crossobamon orientalis
(B) Tenuidactylus caspius
(B) Cyrtopodion scabrum
(A) Cyrtopodion mansarulus
(B) Tropiocolotes nubicus
(B) Mediodactylus russowii
(A) Altiphylax levitoni
(A) Altiphylax medogensis
(AB) Altiphylax stoliczkai
(B) Gekko gecko
(B) Gecko smithi
(B) Gekko vittatus
(B) Gekko monarchus
(B) Luperosaurus cumingii
(B) Lepidodactylus lugubris
(B) Lepidodactylus orientalis
(B) Hemiphyllodactylus changningensis
(A) Hemiphyllodactylus zayuensis
(B) Hemiphyllodactylus longlingensis
(B) Hemiphyllodactylus jinpingensis
(B) Hemiphyllodactylus yunnanensis
(B) Hemiphyllodactylus chiangmaiensis
(B) Hemiphyllodactylus dushanensis
(B) Hemiphyllodactylus banaensis
(B) Hemiphyllodactylus aurantiacus
(B) Hemiphyllodactylus typus
(B) Hemiphyllodactylus insularis
(B) Hemiphyllodactylus ganoklonis
(B) Hemiphyllodactylus tehtarik
(B) Hemiphyllodactylus larutensis
(B) Hemiphyllodactylus titiwangsaensis
(B) Hemiphyllodactylus harterti
(B) Hemiphyllodactylus bintik
(B) Gehyra mutilata
(B) Nactus vankampeni
(B) Dixonius vietnamensis
(B) Phelsuma rosagularis
(B) Phelsuma inexpectata
(B) Homopholis walbergii
(B) Chondrodactylus fitzsimonsi
(B) Calodactylodes illingworthorum
(B) Microgecko persicus
(B) Alsophylax pipiens

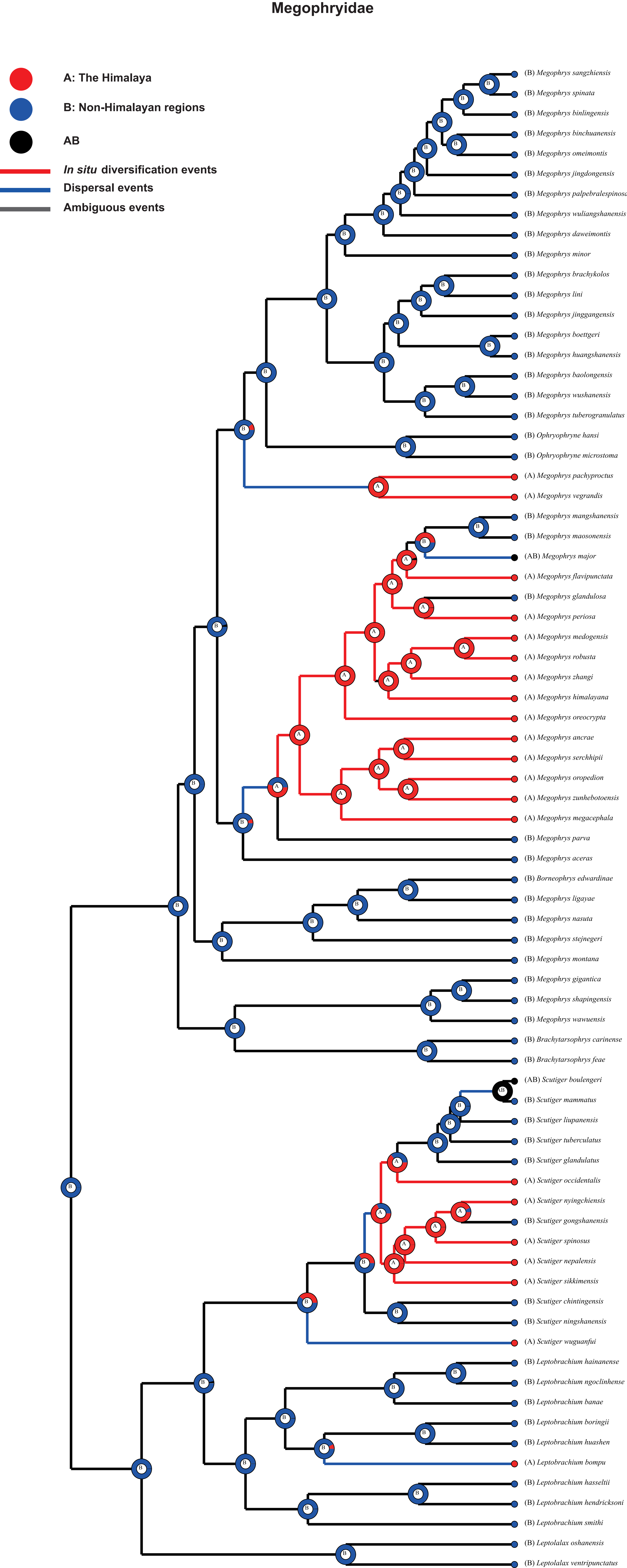

Megophryidae
A: The Himalaya
B: Non-Himalayan regions
AB
In situ diversification events
Dispersal events
Ambiguous events
(B) *Megophrys sangzhiensis*
(B) *Megophrys spinata*
(B) *Megophrys binlingensis*
(B) *Megophrys binchuanensis*
(B) *Megophrys omeimontis*
(B) *Megophrys jingdongensis*
(B) *Megophrys palpebralespinosa*
(B) *Megophrys wuliangshanensis*
(B) *Megophrys daweimontis*
(B) *Megophrys minor*
(B) *Megophrys brachykolos*
(B) *Megophrys lini*
(B) *Megophrys jinggangensis*
(B) *Megophrys boettgeri*
(B) *Megophrys huangshanensis*
(B) *Megophrys baolongensis*
(B) *Megophrys wushanensis*
(B) *Megophrys tuberogranulatus*
(B) *Ophryophryne hansi*
(B) *Ophryophryne microstoma*
(A) *Megophrys pachyproctus*
(A) *Megophrys vegrandis*
(B) *Megophrys mangshanensis*
(B) *Megophrys maosonensis*
(AB) *Megophrys major*
(A) *Megophrys flavipunctata*
(B) *Megophrys glandulosa*
(A) *Megophrys periosa*
(A) *Megophrys medogensis*
(A) *Megophrys robusta*
(A) *Megophrys zhangi*
(A) *Megophrys himalayana*
(A) *Megophrys oreocrypta*
(A) *Megophrys ancrae*
(A) *Megophrys serchhipii*
(A) *Megophrys oropedion*
(A) *Megophrys zunhebotoensis*
(A) *Megophrys megacephala*
(B) *Megophrys parva*
(B) *Megophrys aceras*
(B) *Borneophrys edwardinae*
(B) *Megophrys ligayae*
(B) *Megophrys nasuta*
(B) *Megophrys stejnegeri*
(B) *Megophrys montana*
(B) *Megophrys gigantica*
(B) *Megophrys shapingensis*
(B) *Megophrys wawuensis*
(B) *Brachytarsophrys carinense*
(B) *Brachytarsophrys feae*
(AB) *Scutiger boulengeri*
(B) *Scutiger mammatus*
(B) *Scutiger liupanensis*
(B) *Scutiger tuberculatus*
(B) *Scutiger glandulatus*
(A) *Scutiger occidentalis*
(A) *Scutiger nyingchiensis*
(B) *Scutiger gongshanensis*
(A) *Scutiger spinosus*
(A) *Scutiger nepalensis*
(A) *Scutiger sikkimensis*
(B) *Scutiger chintingensis*
(B) *Scutiger ningshanensis*
(A) *Scutiger wuguanfui*
(B) *Leptobrachium hainanense*
(B) *Leptobrachium ngoclinhense*
(B) *Leptobrachium banae*
(B) *Leptobrachium boringii*
(B) *Leptobrachium huashen*
(A) *Leptobrachium bompu*
(B) *Leptobrachium hasseltii*
(B) *Leptobrachium hendricksoni*
(B) *Leptobrachium smithi*
(B) *Leptolalax oshanensis*
(B) *Leptolalax ventripunctatus*

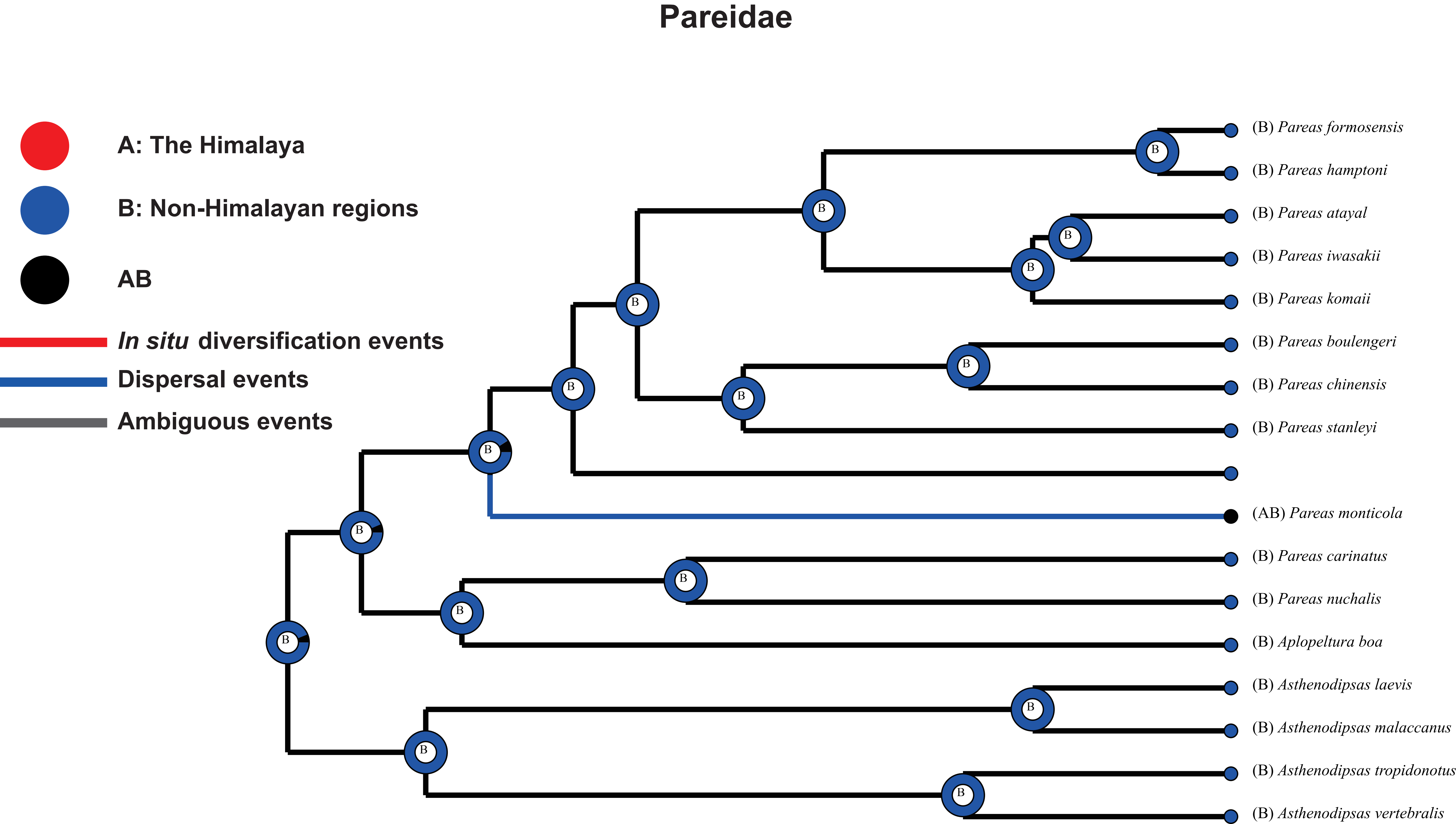
Pareidae
A: The Himalaya
B: Non-Himalayan regions
AB
In situ diversification events
Dispersal events
Ambiguous events
(B) Pareas formosensis
(B) Pareas hamptoni
(B) Pareas atayal
(B) Pareas iwasakii
(B) Pareas komaii
(B) Pareas boulengeri
(B) Pareas chinensis
(B) Pareas stanleyi
(AB) Pareas monticola
(B) Pareas carinatus
(B) Pareas nuchalis
(B) Aplopeltura boa
(B) Asthenodipsas laevis
(B) Asthenodipsas malaccanus
(B) Asthenodipsas tropidonotus
(B) Asthenodipsas vertebralis

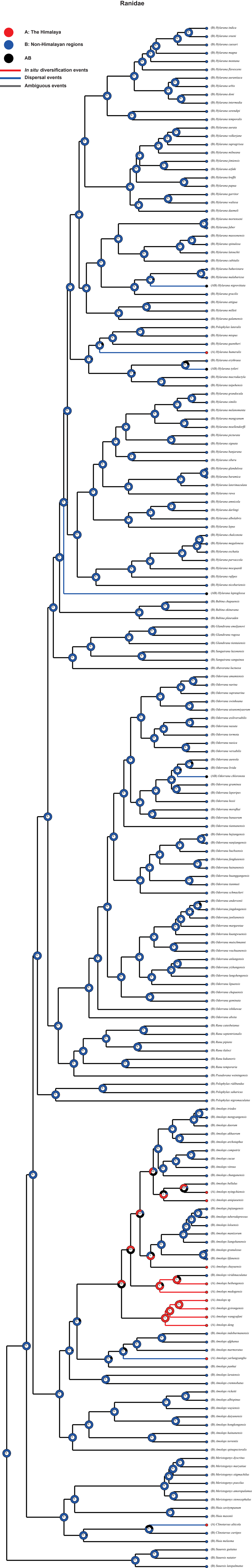
Ranidae
A: The Himalaya
B: Non-Himalayan regions
AB
In situ diversification events
Dispersal events
Ambiguous events

Rhacophoridae

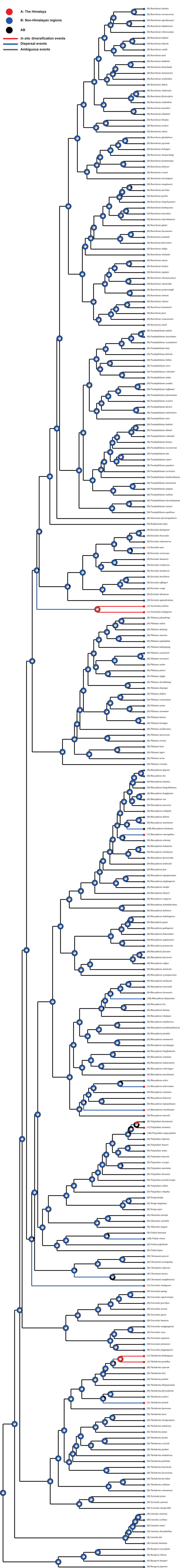

Scinidae

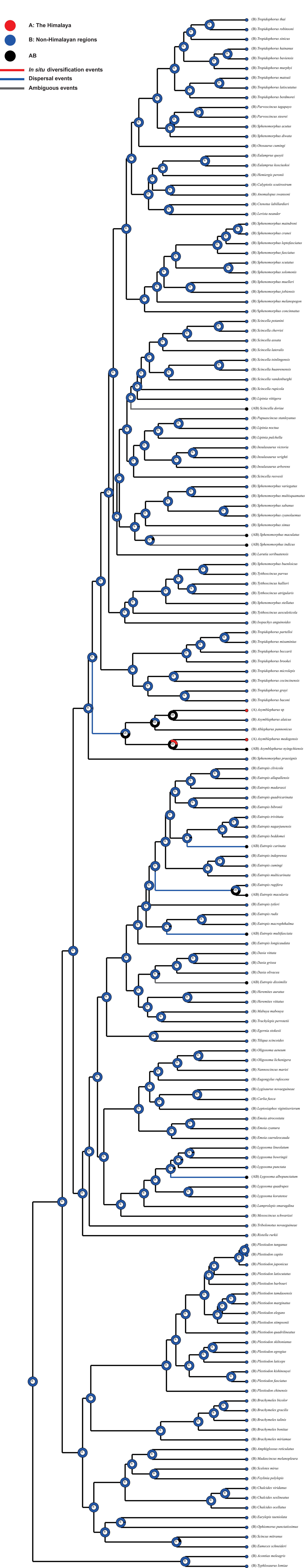

A: The Himalaya
B: Non-Himalayan regions
AB
In situ diversification events
Dispersal events
Ambiguous events
(B) Tropidophorus thai
(B) Tropidophorus robinsoni
(B) Tropidophorus sinicus
(B) Tropidophorus hainanus
(B) Tropidophorus baviensis
(B) Tropidophorus murphyi
(B) Tropidophorus matsuii
(B) Tropidophorus latiscutatus
(B) Tropidophorus berdmorei
(B) Parvoscincus tagapayo
(B) Parvoscincus steerei
(B) Sphenomorphus acutus
(B) Sphenomorphus diwata
(B) Otosaurus cumingii
(B) Eulamprus quoyii
(B) Eulamprus kosciuskoi
(B) Hemiergis peronii
(B) Calyptotis scutirostrum
(B) Anomalopus swansoni
(B) Ctenotus labillardieri
(B) Lerista neander
(B) Sphenomorphus maindroni
(B) Sphenomorphus cranei
(B) Sphenomorphus leptofasciatus
(B) Sphenomorphus fasciatus
(B) Sphenomorphus scutatus
(B) Sphenomorphus solomonis
(B) Sphenomorphus muelleri
(B) Sphenomorphus jobiensis
(B) Sphenomorphus melanopogon
(B) Sphenomorphus concinnatus
(B) Scincella potanini
(B) Scincella cherriei
(B) Scincella assata
(B) Scincella lateralis
(B) Scincella tsinlingensis
(B) Scincella huanrenensis
(B) Scincella vandenburghi
(B) Scincella rupicola
(B) Lipinia vittigera
(AB) Scincella doriae
(B) Papuascincus stanleyanus
(B) Lipinia noctua
(B) Lipinia pulchella
(B) Insulasaurus victoria
(B) Insulasaurus wrighti
(B) Insulasaurus arboreus
(B) Scincella reevesii
(B) Sphenomorphus variegatus
(B) Sphenomorphus multisquamatus
(B) Sphenomorphus sabanus
(B) Sphenomorphus cyanolaemus
(B) Sphenomorphus simus
(AB) Sphenomorphus maculatus
(AB) Sphenomorphus indicus
(B) Larutia seribuatensis
(B) Sphenomorphus buenloicus
(B) Tytthoscincus parvus
(B) Tytthoscincus hallieri
(B) Tytthoscincus atrigularis
(B) Sphenomorphus stellatus
(B) Tytthoscincus aesculeticola
(B) Isopachys anguinoides
(B) Tropidophorus partelloi
(B) Tropidophorus misaminius
(B) Tropidophorus beccarii
(B) Tropidophorus brookei
(B) Tropidophorus microlepis
(B) Tropidophorus cocincinensis
(B) Tropidophorus grayi
(B) Tropidophorus baconi
(A) Asymblepharus sp
(B) Asymblepharus alaicus
(B) Ablepharus pannonicus
(A) Asymblepharus medogensis
(AB) Asymblepharus nyingchiensis
(B) Sphenomorphus praesignis
(B) Eutropis clivicola
(B) Eutropis allapallensis
(B) Eutropis madaraszi
(B) Eutropis quadricarinata
(B) Eutropis bibronii
(B) Eutropis trivittata
(B) Eutropis nagarjunensis
(B) Eutropis beddomei
(AB) Eutropis carinata
(B) Eutropis indeprensa
(B) Eutropis cumingi
(B) Eutropis multicarinata
(B) Eutropis rugifera
(AB) Eutropis macularia
(B) Eutropis tytleri
(B) Eutropis rudis
(B) Eutropis macrophthalma
(AB) Eutropis multifasciata
(B) Eutropis longicaudata
(B) Dasia vittata
(B) Dasia grisea
(B) Dasia olivacea
(AB) Eutropis dissimilis
(B) Heremites auratus
(B) Heremites vittatus
(B) Mabuya mabouya
(B) Trachylepis perrotetii
(B) Egernia stokesii
(B) Tiliqua scincoides
(B) Oligosoma aeneum
(B) Oligosoma lichenigera
(B) Nannoscincus mariei
(B) Eugongylus rufescens
(B) Lygisaurus novaeguineae
(B) Carlia fusca
(B) Leptosiaphos vigintiserierum
(B) Emoia atrocostata
(B) Emoia cyanura
(B) Emoia caeruleocauda
(B) Lygosoma lineolatum
(B) Lygosoma bowringii
(B) Lygosoma punctata
(AB) Lygosoma albopunctatum
(B) Lygosoma quadrupes
(B) Lygosoma koratense
(B) Lamprolepis smaragdina
(B) Mesoscincus schwartzei
(B) Tribolonotus novaeguineae
(B) Ristella rurkii
(B) Plestiodon tunganus
(B) Plestiodon capito
(B) Plestiodon japonicus
(B) Plestiodon latiscutatus
(B) Plestiodon barbouri
(B) Plestiodon tamdaoensis
(B) Plestiodon marginatus
(B) Plestiodon elegans
(B) Plestiodon stimpsonii
(B) Plestiodon quadrilineatus
(B) Plestiodon skiltonianus
(B) Plestiodon egregius
(B) Plestiodon laticeps
(B) Plestiodon kishinouyei
(B) Plestiodon fasciatus
(B) Plestiodon chinensis
(B) Brachymeles bicolor
(B) Brachymeles gracilis
(B) Brachymeles talinis
(B) Brachymeles bonitae
(B) Brachymeles miriamae
(B) Amphiglossus reticulatus
(B) Madascincus melanopleura
(B) Scelotes mirus
(B) Feylinia polylepis
(B) Chalcides viridanus
(B) Chalcides sexlineatus
(B) Chalcides ocellatus
(B) Eurylepis taeniolata
(B) Ophiomorus punctatissimus
(B) Scincus mitranus
(B) Eumeces schneideri
(B) Acontias meleagris
(B) Typhlosaurus lomiae

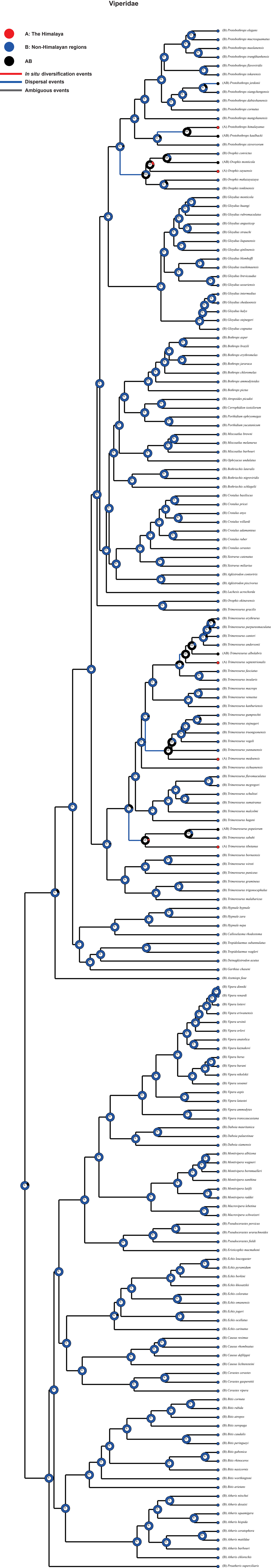
Viperidae
A: The Himalaya
B: Non-Himalayan regions
AB
In situ diversification events
Dispersal events
Ambiguous events

Data S4.

BioGeoBEARS analysis results based on nine regions. Trees with ancestral nodes reconstructed for specific biogeographic regions, indicated by the following letters and colors: Red (A): the Himalaya; red (B): Southeast Asia, including tropical regions of China, Malesia, and Papuasia; cyan (C): East Asia, the eastern boreal part of Russia and temperate regions of East Asia, including Japan and Taiwan; dark green (D): South Asia, south of the Himalaya, including Sri Lanka; purple (E): Central–West Asia, including the Pakistan, Uzbekistan, Tajikistan, The Arabia Peninsula countries); orange (F): Australia (and New Zealand); gray (G): Europe. light green (H): Africa; blue (I): America (north and south America); black: combined regions (the Himalaya and other regions like AB); white: other regions (etc., BCD). Red branches represent "dispersal into the Himalaya" events, black branches were not related to Himalayan diversification.

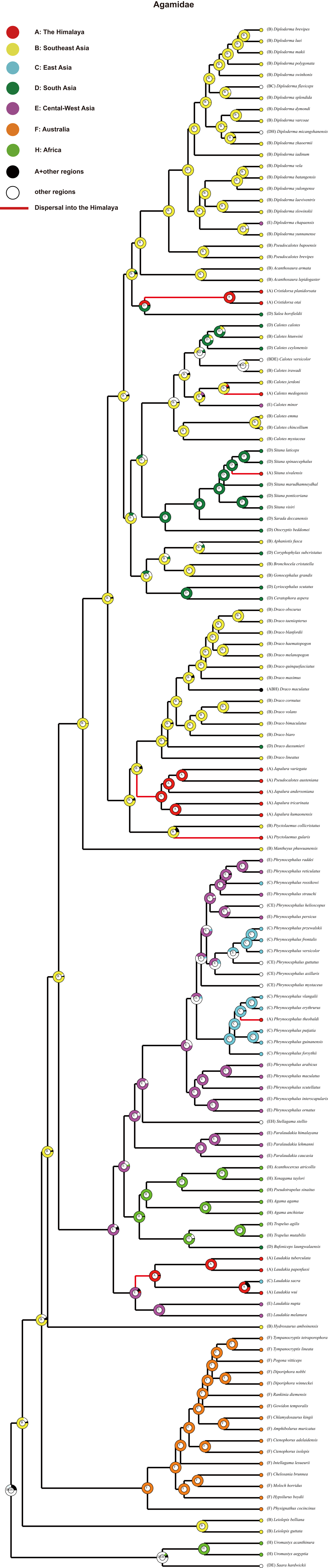
Agamidae
A: The Himalaya
B: Southeast Asia
C: East Asia
D: South Asia
E: Cental-West Asia
F: Australia
H: Africa
A+other regions
other regions
Dispersal into the Himalaya
(B) Diploderma brevipes
(B) Diploderma luei
(B) Diploderma makii
(B) Diploderma polygonata
(B) Diploderma swinhonis
(BC) Diploderma flaviceps
(B) Diploderma splendida
(B) Diploderma dymondi
(B) Diploderma varcoae
(DH) Diploderma micangshanensis
(B) Diploderma zhaoermii
(B) Diploderma iadinum
(B) Diploderma vela
(B) Diploderma batangensis
(B) Diploderma yulongense
(B) Diploderma laeviventris
(B) Diploderma slowinskii
(E) Diploderma chapaensis
(B) Diploderma yunnanense
(B) Pseudocalotes bapoensis
(B) Pseudocalotes brevipes
(B) Acanthosaura armata
(B) Acanthosaura lepidogaster
(A) Cristidorsa planidorsata
(A) Cristidorsa otai
(D) Salea horsfieldii
(D) Calotes calotes
(B) Calotes htunwini
(D) Calotes ceylonensis
(BDE) Calotes versicolor
(B) Calotes irawadi
(B) Calotes jerdoni
(A) Calotes medogensis
(E) Calotes minor
(B) Calotes emma
(B) Calotes chincollium
(B) Calotes mystaceus
(D) Sitana laticeps
(D) Sitana spinaecephalus
(A) Sitana sivalensis
(D) Sitana marudhamneydhal
(D) Sitana ponticeriana
(D) Sitana visiri
(D) Sarada deccanensis
(D) Otocryptis beddomei
(B) Aphaniotis fusca
(D) Coryphophylax subcristatus
(B) Bronchocela cristatella
(B) Gonocephalus grandis
(D) Lyriocephalus scutatus
(D) Ceratophora aspera
(B) Draco obscurus
(B) Draco taeniopterus
(B) Draco blanfordii
(B) Draco haematopogon
(B) Draco melanopogon
(B) Draco quinquefasciatus
(B) Draco maximus
(ABH) Draco maculatus
(B) Draco cornutus
(B) Draco volans
(B) Draco bimaculatus
(B) Draco biaro
(D) Draco dussumieri
(B) Draco lineatus
(A) Japalura variegata
(A) Pseudocalotes austeniana
(A) Japalura andersoniana
(A) Japalura tricarinata
(A) Japalura kumaonensis
(B) Ptyctolaemus collicristatus
(A) Ptyctolaemus gularis
(B) Mantheyus phuwuanensis
(E) Phrynocephalus raddei
(E) Phrynocephalus reticulatus
(C) Phrynocephalus rossikowi
(E) Phrynocephalus strauchi
(CE) Phrynocephalus helioscopus
(E) Phrynocephalus persicus
(C) Phrynocephalus przewalskii
(C) Phrynocephalus frontalis
(C) Phrynocephalus versicolor
(CE) Phrynocephalus guttatus
(CE) Phrynocephalus axillaris
(CE) Phrynocephalus mystaceus
(C) Phrynocephalus vlangalii
(C) Phrynocephalus erythrurus
(A) Phrynocephalus theobaldi
(C) Phrynocephalus putjatia
(C) Phrynocephalus guinanensis
(C) Phrynocephalus forsythii
(E) Phrynocephalus arabicus
(E) Phrynocephalus maculatus
(E) Phrynocephalus scutellatus
(E) Phrynocephalus interscapularis
(E) Phrynocephalus ornatus
(EH) Stellagama stellio
(E) Paralaudakia himalayana
(E) Paralaudakia lehmanni
(E) Paralaudakia caucasia
(H) Acanthocercus atricollis
(H) Xenagama taylori
(H) Pseudotrapelus sinaitus
(H) Agama agama
(H) Agama anchietae
(H) Trapelus agilis
(H) Trapelus mutabilis
(D) Bufoniceps laungwalaensis
(A) Laudakia tuberculata
(A) Laudakia papenfussi
(C) Laudakia sacra
(A) Laudakia wui
(E) Laudakia nupta
(E) Laudakia melanura
(B) Hydrosaurus amboinensis
(F) Tympanocryptis tetraporophora
(F) Tympanocryptis lineata
(F) Pogona vitticeps
(F) Diporiphora nobbi
(F) Diporiphora winneckei
(F) Rankinia diemensis
(F) Gowidon temporalis
(F) Chlamydosaurus kingii
(F) Amphibolurus muricatus
(F) Ctenophorus adelaidensis
(F) Ctenophorus isolepis
(F) Intellagama lesueurii
(F) Chelosania brunnea
(F) Moloch horridus
(F) Hypsilurus boydii
(F) Physignathus cocincinus
(B) Leiolepis belliana
(B) Leiolepis guttata
(H) Uromastyx acanthinura
(H) Uromastyx aegyptia
(DE) Saara hardwickii

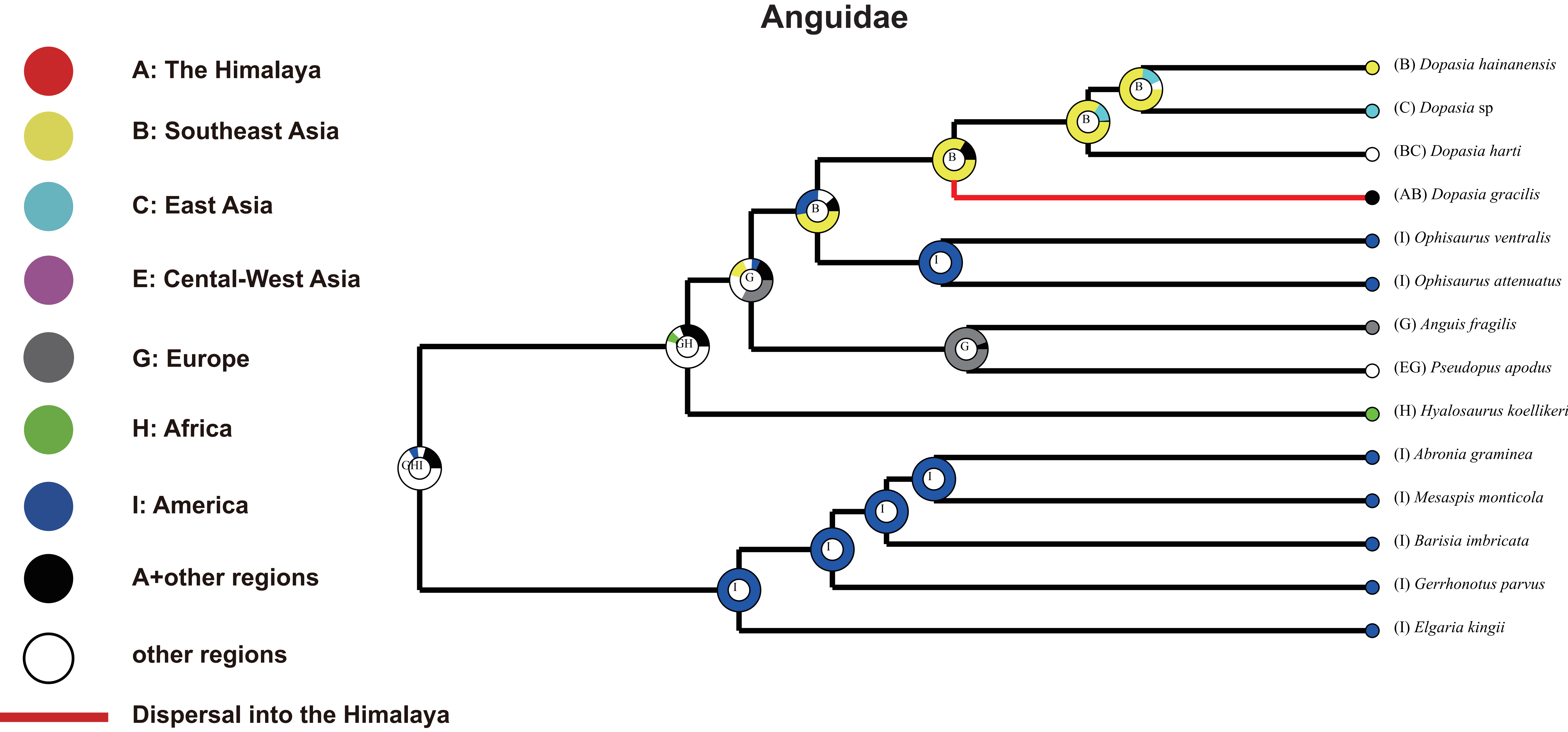
Anguidae
A: The Himalaya
B: Southeast Asia
C: East Asia
E: Cental-West Asia
G: Europe
H: Africa
I: America
A+other regions
other regions
Dispersal into the Himalaya
(B) Dopasia hainanensis
(C) Dopasia sp
(BC) Dopasia harti
(AB) Dopasia gracilis
(I) Ophisaurus ventralis
(I) Ophisaurus attenuatus
(G) Anguis fragilis
(EG) Pseudopus apodus
(H) Hyalosaurus koellikeri
(I) Abronia graminea
(I) Mesaspis monticola
(I) Barisia imbricata
(I) Gerrhonotus parvus
(I) Elgaria kingii
B
B
B
B
I
G
G
GH
GHI
I
I
I
I
I

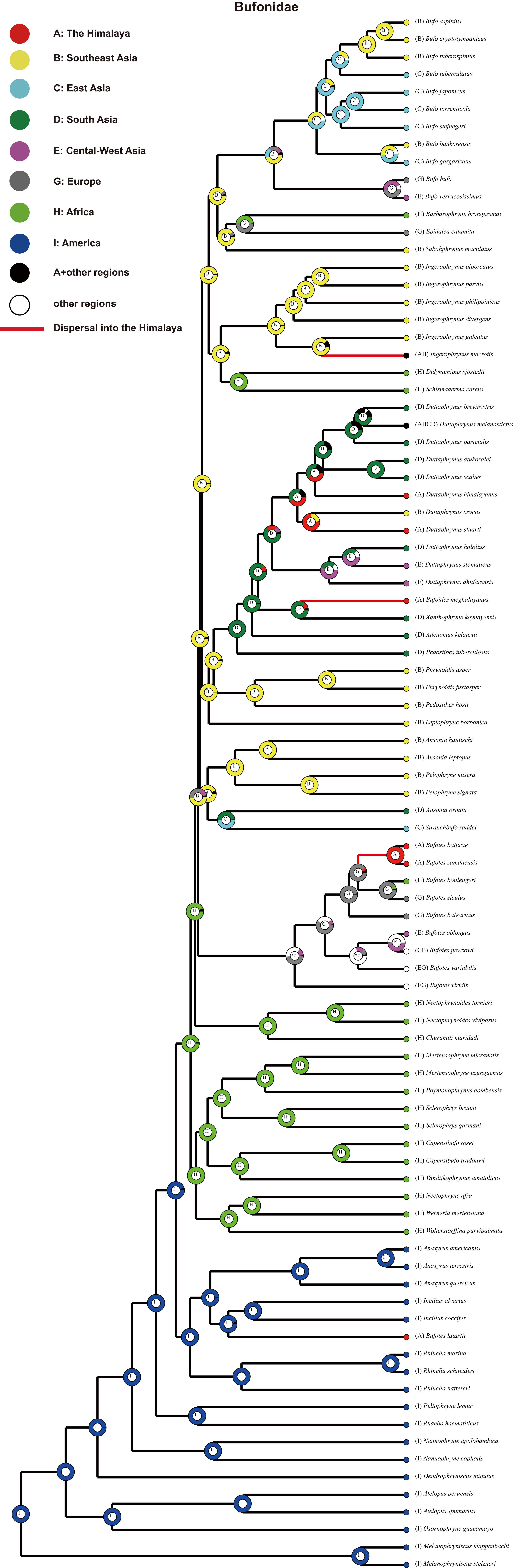
Bufonidae
A: The Himalaya
B: Southeast Asia
C: East Asia
D: South Asia
E: Cental-West Asia
G: Europe
H: Africa
I: America
A+other regions
other regions
Dispersal into the Himalaya
(B) Bufo aspinius
(B) Bufo cryptotympanicus
(B) Bufo tuberospinius
(C) Bufo tuberculatus
(C) Bufo japonicus
(C) Bufo torrenticola
(C) Bufo stejnegeri
(B) Bufo bankorensis
(C) Bufo gargarizans
(G) Bufo bufo
(E) Bufo verrucosissimus
(H) Barbarophryne brongersmai
(G) Epidalea calamita
(B) Sabahphrynus maculatus
(B) Ingerophrynus biporcatus
(B) Ingerophrynus parvus
(B) Ingerophrynus philippinicus
(B) Ingerophrynus divergens
(B) Ingerophrynus galeatus
(AB) Ingerophrynus macrotis
(H) Didynamipus sjostedti
(H) Schismaderma carens
(D) Duttaphrynus brevirostris
(ABCD) Duttaphrynus melanostictus
(D) Duttaphrynus parietalis
(D) Duttaphrynus atukoralei
(D) Duttaphrynus scaber
(A) Duttaphrynus himalayanus
(B) Duttaphrynus crocus
(A) Duttaphrynus stuarti
(D) Duttaphrynus hololius
(E) Duttaphrynus stomaticus
(E) Duttaphrynus dhufarensis
(A) Bufoides meghalayanus
(D) Xanthophryne koynayensis
(D) Adenomus kelaartii
(D) Pedostibes tuberculosus
(B) Phrynoidis asper
(B) Phrynoidis juxtasper
(B) Pedostibes hosii
(B) Leptophryne borbonica
(B) Ansonia hanitschi
(B) Ansonia leptopus
(B) Pelophryne misera
(B) Pelophryne signata
(D) Ansonia ornata
(C) Strauchbufo raddei
(A) Bufotes baturae
(A) Bufotes zamdaensis
(H) Bufotes boulengeri
(G) Bufotes siculus
(G) Bufotes balearicus
(E) Bufotes oblongus
(CE) Bufotes pewzowi
(EG) Bufotes variabilis
(EG) Bufotes viridis
(H) Nectophrynoides tornieri
(H) Nectophrynoides viviparus
(H) Churamiti maridadi
(H) Mertensophryne micranotis
(H) Mertensophryne uzunguensis
(H) Poyntonophrynus dombensis
(H) Sclerophrys brauni
(H) Sclerophrys garmani
(H) Capensibufo rosei
(H) Capensibufo tradouwi
(H) Vandijkophrynus amatolicus
(H) Nectophryne afra
(H) Werneria mertensiana
(H) Wolterstorffina parvipalmata
(I) Anaxyrus americanus
(I) Anaxyrus terrestris
(I) Anaxyrus quercicus
(I) Incilius alvarius
(I) Incilius coccifer
(A) Bufotes latastii
(I) Rhinella marina
(I) Rhinella schneideri
(I) Rhinella nattereri
(I) Peltophryne lemur
(I) Rhaebo haematiticus
(I) Nannophryne apolobambica
(I) Nannophryne cophotis
(I) Dendrophryniscus minutus
(I) Atelopus peruensis
(I) Atelopus spumarius
(I) Osornophryne guacamayo
(I) Melanophryniscus klappenbachi
(I) Melanophryniscus stelzneri

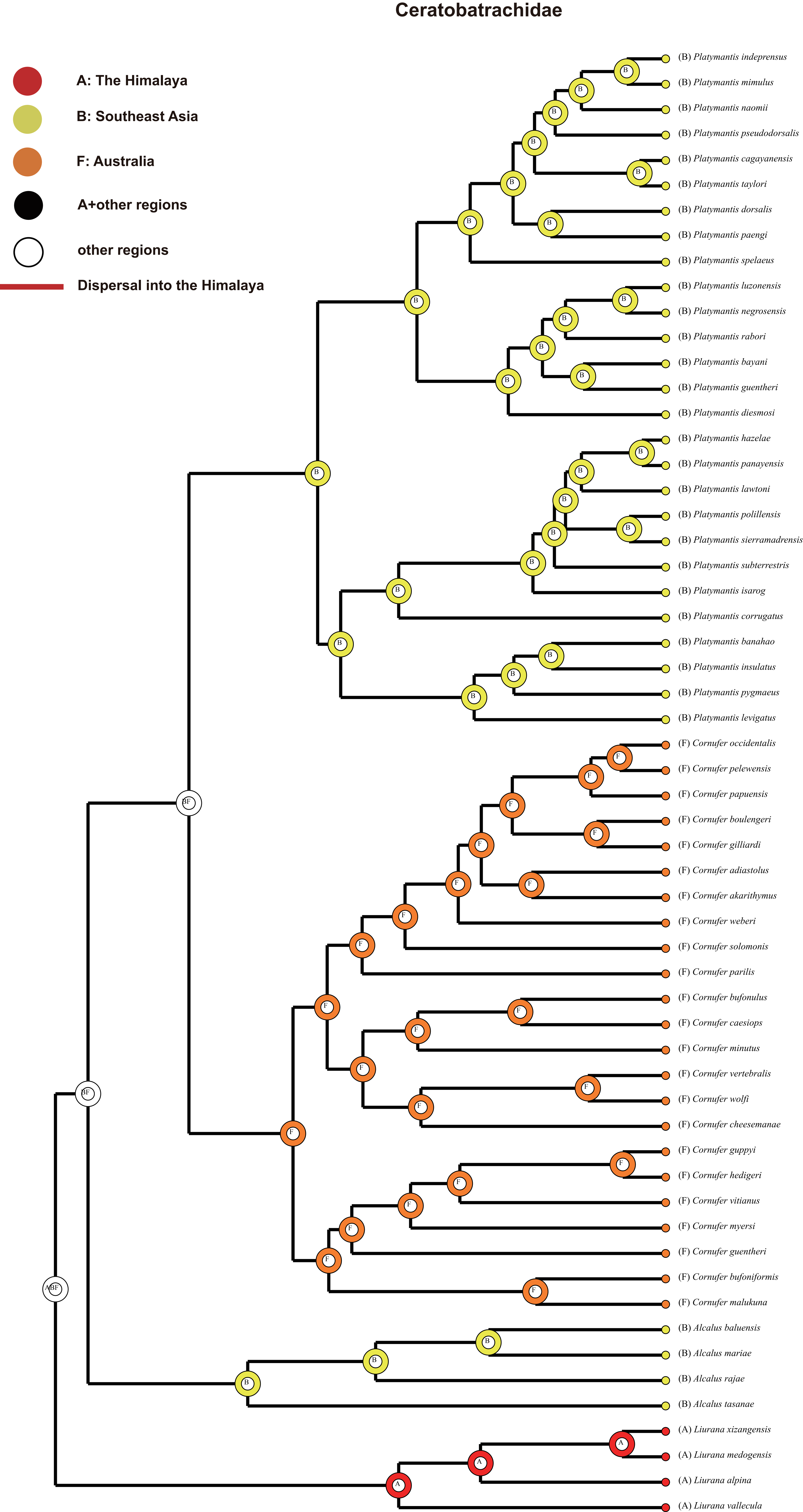
Ceratobatrachidae
A: The Himalaya
B: Southeast Asia
F: Australia
A+other regions
other regions
Dispersal into the Himalaya
(B) Platymantis indeprensus
(B) Platymantis mimulus
(B) Platymantis naomii
(B) Platymantis pseudodorsalis
(B) Platymantis cagayanensis
(B) Platymantis taylori
(B) Platymantis dorsalis
(B) Platymantis paengi
(B) Platymantis spelaeus
(B) Platymantis luzonensis
(B) Platymantis negrosensis
(B) Platymantis rabori
(B) Platymantis bayani
(B) Platymantis guentheri
(B) Platymantis diesmosi
(B) Platymantis hazelae
(B) Platymantis panayensis
(B) Platymantis lawtoni
(B) Platymantis polillensis
(B) Platymantis sierramadrensis
(B) Platymantis subterrestris
(B) Platymantis isarog
(B) Platymantis corrugatus
(B) Platymantis banahao
(B) Platymantis insulatus
(B) Platymantis pygmaeus
(B) Platymantis levigatus
(F) Cornufer occidentalis
(F) Cornufer pelewensis
(F) Cornufer papuensis
(F) Cornufer boulengeri
(F) Cornufer gilliardi
(F) Cornufer adiastolus
(F) Cornufer akarithymus
(F) Cornufer weberi
(F) Cornufer solomonis
(F) Cornufer parilis
(F) Cornufer bufonulus
(F) Cornufer caesiops
(F) Cornufer minutus
(F) Cornufer vertebralis
(F) Cornufer wolfi
(F) Cornufer cheesemanae
(F) Cornufer guppyi
(F) Cornufer hedigeri
(F) Cornufer vitianus
(F) Cornufer myersi
(F) Cornufer guentheri
(F) Cornufer bufoniformis
(F) Cornufer malukuna
(B) Alcalus baluensis
(B) Alcalus mariae
(B) Alcalus rajae
(B) Alcalus tasanae
(A) Liurana xizangensis
(A) Liurana medogensis
(A) Liurana alpina
(A) Liurana vallecula

Colubridae

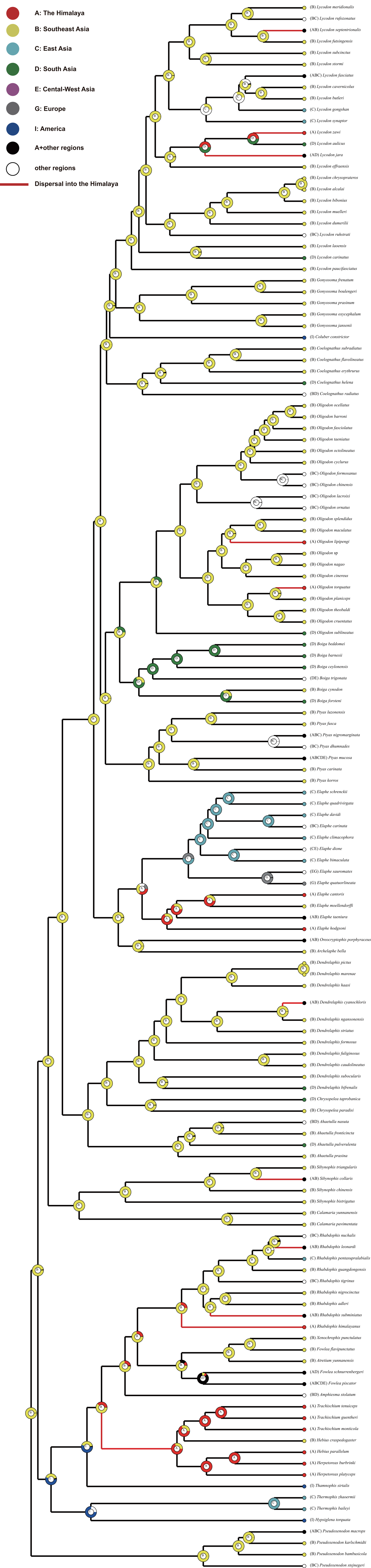

A: The Himalaya
B: Southeast Asia
C: East Asia
D: South Asia
E: Cental-West Asia
G: Europe
I: America
A+other regions
other regions
Dispersal into the Himalaya
(B) Lycodon meridionalis
(BC) Lycodon rufozonatus
(AB) Lycodon septentrionalis
(B) Lycodon futsingensis
(B) Lycodon subcinctus
(B) Lycodon stormi
(ABC) Lycodon fasciatus
(B) Lycodon cavernicolus
(B) Lycodon butleri
(C) Lycodon gongshan
(C) Lycodon synaptor
(A) Lycodon zawi
(D) Lycodon aulicus
(AD) Lycodon jara
(B) Lycodon effraensis
(B) Lycodon chrysoprateros
(B) Lycodon alcalai
(B) Lycodon bibonius
(B) Lycodon muelleri
(B) Lycodon dumerilii
(BC) Lycodon ruhstrati
(B) Lycodon laoensis
(D) Lycodon carinatus
(B) Lycodon paucifasciatus
(B) Gonyosoma frenatum
(B) Gonyosoma boulengeri
(B) Gonyosoma prasinum
(B) Gonyosoma oxycephalum
(B) Gonyosoma jansenii
(I) Coluber constrictor
(B) Coelognathus subradiatus
(B) Coelognathus flavolineatus
(B) Coelognathus erythrurus
(D) Coelognathus helena
(BD) Coelognathus radiatus
(B) Oligodon ocellatus
(B) Oligodon barroni
(B) Oligodon fasciolatus
(B) Oligodon taeniatus
(B) Oligodon octolineatus
(B) Oligodon cyclurus
(BC) Oligodon formosanus
(BC) Oligodon chinensis
(BC) Oligodon lacroixi
(BC) Oligodon ornatus
(B) Oligodon splendidus
(B) Oligodon maculatus
(A) Oligodon lipipengi
(B) Oligodon sp
(B) Oligodon nagao
(B) Oligodon cinereus
(A) Oligodon torquatus
(B) Oligodon planiceps
(B) Oligodon theobaldi
(B) Oligodon cruentatus
(D) Oligodon sublineatus
(D) Boiga beddomei
(D) Boiga barnesii
(D) Boiga ceylonensis
(DE) Boiga trigonata
(B) Boiga cynodon
(D) Boiga forsteni
(B) Ptyas luzonensis
(B) Ptyas fusca
(ABC) Ptyas nigromarginata
(BC) Ptyas dhumnades
(ABCDE) Ptyas mucosa
(B) Ptyas carinata
(B) Ptyas korros
(C) Elaphe schrenckii
(C) Elaphe quadrivirgata
(C) Elaphe davidi
(BC) Elaphe carinata
(C) Elaphe climacophora
(CE) Elaphe dione
(C) Elaphe bimaculata
(EG) Elaphe sauromates
(G) Elaphe quatuorlineata
(A) Elaphe cantoris
(B) Elaphe moellendorffi
(AB) Elaphe taeniura
(A) Elaphe hodgsoni
(AB) Oreocryptophis porphyraceus
(B) Archelaphe bella
(B) Dendrelaphis pictus
(B) Dendrelaphis marenae
(B) Dendrelaphis haasi
(AB) Dendrelaphis cyanochloris
(B) Dendrelaphis ngansonensis
(B) Dendrelaphis striatus
(B) Dendrelaphis formosus
(B) Dendrelaphis fuliginosus
(B) Dendrelaphis caudolineatus
(B) Dendrelaphis subocularis
(D) Dendrelaphis bifrenalis
(D) Chrysopelea taprobanica
(B) Chrysopelea paradisi
(BD) Ahaetulla nasuta
(B) Ahaetulla fronticincta
(D) Ahaetulla pulverulenta
(B) Ahaetulla prasina
(B) Sibynophis triangularis
(AB) Sibynophis collaris
(B) Sibynophis chinensis
(B) Sibynophis bistrigatus
(B) Calamaria yunnanensis
(B) Calamaria pavimentata
(BC) Rhabdophis nuchalis
(AB) Rhabdophis leonardi
(C) Rhabdophis pentasupralabialis
(B) Rhabdophis guangdongensis
(BC) Rhabdophis tigrinus
(B) Rhabdophis nigrocinctus
(B) Rhabdophis adleri
(AB) Rhabdophis subminiatus
(A) Rhabdophis himalayanus
(B) Xenochrophis punctulatus
(B) Fowlea flavipunctatus
(B) Atretium yunnanensis
(AD) Fowlea schnurrenbergeri
(ABCDE) Fowlea piscator
(BD) Amphiesma stolatum
(A) Trachischium tenuiceps
(A) Trachischium guentheri
(A) Trachischium monticola
(B) Hebius craspedogaster
(A) Hebius parallelum
(A) Herpetoreas burbrinki
(A) Herpetoreas platyceps
(I) Thamnophis sirtalis
(C) Thermophis zhaoermii
(C) Thermophis baileyi
(I) Hypsiglena torquata
(ABC) Pseudoxenodon macrops
(B) Pseudoxenodon karlschmidti
(B) Pseudoxenodon bambusicola
(BC) Pseudoxenodon stejnegeri

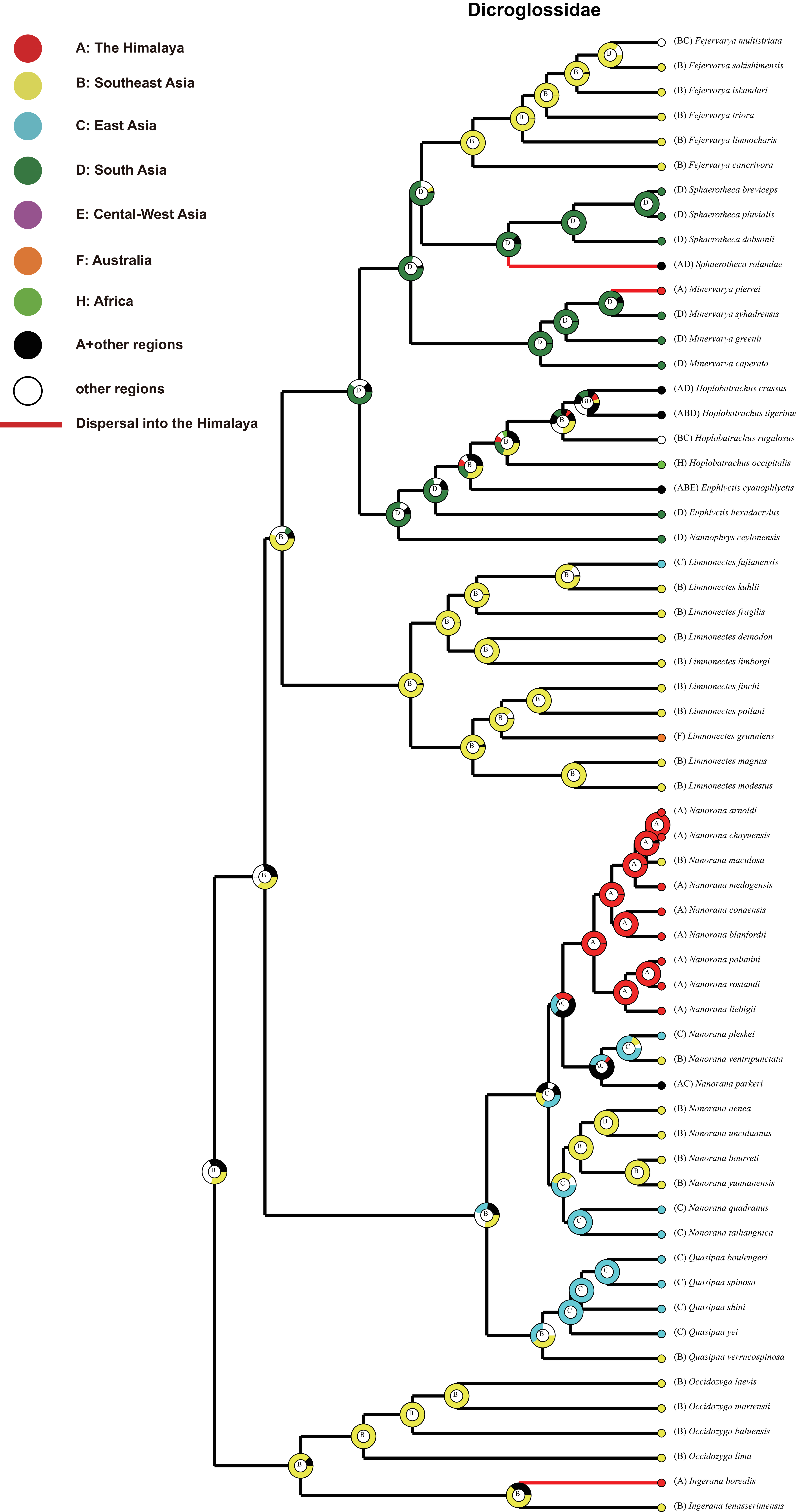
Dicroglossidae
A: The Himalaya
B: Southeast Asia
C: East Asia
D: South Asia
E: Cental-West Asia
F: Australia
H: Africa
A+other regions
other regions
Dispersal into the Himalaya
(BC) Fejervarya multistriata
(B) Fejervarya sakishimensis
(B) Fejervarya iskandari
(B) Fejervarya triora
(B) Fejervarya limnocharis
(B) Fejervarya cancrivora
(D) Sphaerotheca breviceps
(D) Sphaerotheca pluvialis
(D) Sphaerotheca dobsonii
(AD) Sphaerotheca rolandae
(A) Minervarya pierrei
(D) Minervarya syhadrensis
(D) Minervarya greenii
(D) Minervarya caperata
(AD) Hoplobatrachus crassus
(ABD) Hoplobatrachus tigerinu
(BC) Hoplobatrachus rugulosus
(H) Hoplobatrachus occipitalis
(ABE) Euphlyctis cyanophlyctis
(D) Euphlyctis hexadactylus
(D) Nannophrys ceylonensis
(C) Limnonectes fujianensis
(B) Limnonectes kuhlii
(B) Limnonectes fragilis
(B) Limnonectes deinodon
(B) Limnonectes limborgi
(B) Limnonectes finchi
(B) Limnonectes poilani
(F) Limnonectes grunniens
(B) Limnonectes magnus
(B) Limnonectes modestus
(A) Nanorana arnoldi
(A) Nanorana chayuensis
(B) Nanorana maculosa
(A) Nanorana medogensis
(A) Nanorana conaensis
(A) Nanorana blanfordii
(A) Nanorana polunini
(A) Nanorana rostandi
(A) Nanorana liebigii
(C) Nanorana pleskei
(B) Nanorana ventripunctata
(AC) Nanorana parkeri
(B) Nanorana aenea
(B) Nanorana unculuanus
(B) Nanorana bourreti
(B) Nanorana yunnanensis
(C) Nanorana quadranus
(C) Nanorana taihangnica
(C) Quasipaa boulengeri
(C) Quasipaa spinosa
(C) Quasipaa shini
(C) Quasipaa yei
(B) Quasipaa verrucospinosa
(B) Occidozyga laevis
(B) Occidozyga martensii
(B) Occidozyga baluensis
(B) Occidozyga lima
(A) Ingerana borealis
(B) Ingerana tenasserimensis

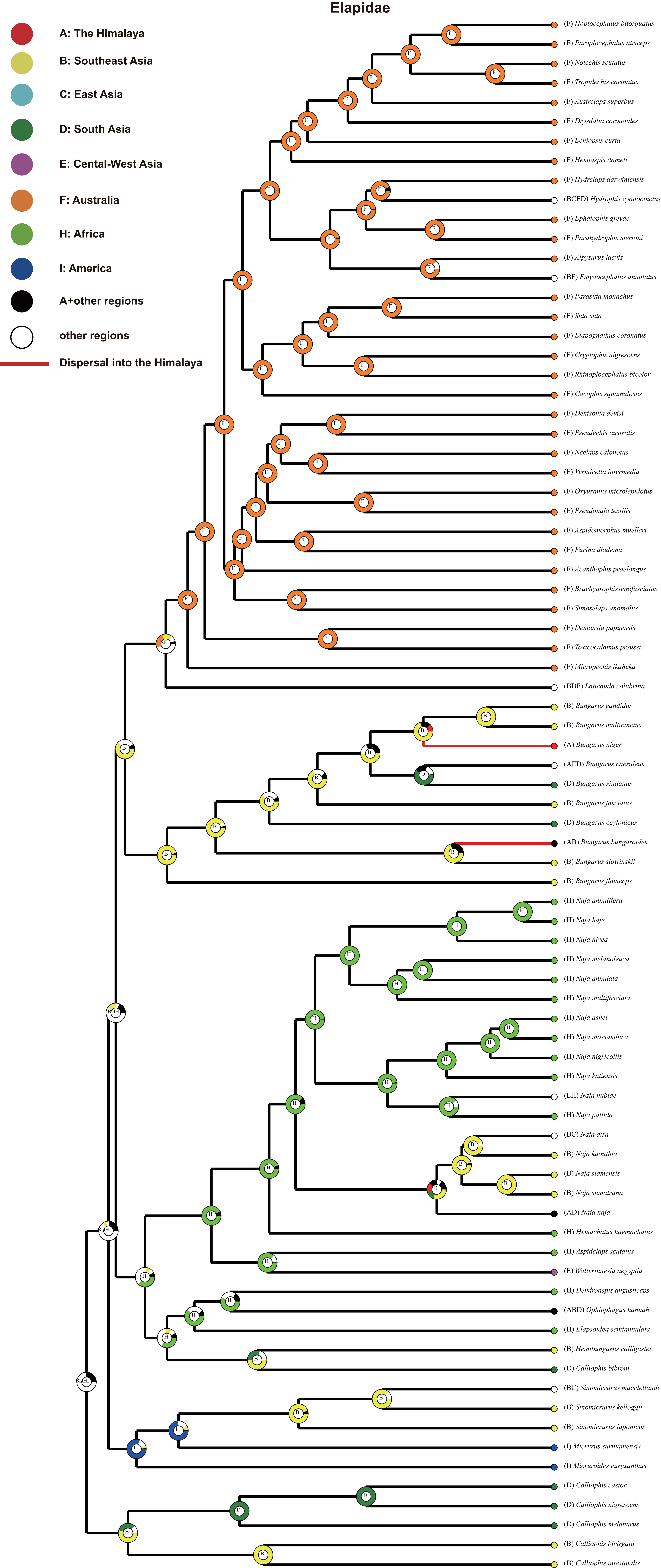
Elapidae
A: The Himalaya
B: Southeast Asia
C: East Asia
D: South Asia
E: Cental-West Asia
F: Australia
H: Africa
I: America
A+other regions
other regions
Dispersal into the Himalaya
(F) Hoplocephalus bitorquatus
(F) Paroplocephalus atriceps
(F) Notechis scutatus
(F) Tropidechis carinatus
(F) Austrelaps superbus
(F) Drysdalia coronoides
(F) Echiopsis curta
(F) Hemiaspis dameli
(F) Hydrelaps darwiniensis
(BCED) Hydrophis cyanocinctus
(F) Ephalophis greyae
(F) Parahydrophis mertoni
(F) Aipysurus laevis
(BF) Emydocephalus annulatus
(F) Parasuta monachus
(F) Suta suta
(F) Elapognathus coronatus
(F) Cryptophis nigrescens
(F) Rhinoplocephalus bicolor
(F) Cacophis squamulosus
(F) Denisonia devisi
(F) Pseudechis australis
(F) Neelaps calonotus
(F) Vermicella intermedia
(F) Oxyuranus microlepidotus
(F) Pseudonaja textilis
(F) Aspidomorphus muelleri
(F) Furina diadema
(F) Acanthophis praelongus
(F) Brachyurophissemifasciatus
(F) Simoselaps anomalus
(F) Demansia papuensis
(F) Toxicocalamus preussi
(F) Micropechis ikaheka
(BDF) Laticauda colubrina
(B) Bungarus candidus
(B) Bungarus multicinctus
(A) Bungarus niger
(AED) Bungarus caeruleus
(D) Bungarus sindanus
(B) Bungarus fasciatus
(D) Bungarus ceylonicus
(AB) Bungarus bungaroides
(B) Bungarus slowinskii
(B) Bungarus flaviceps
(H) Naja annulifera
(H) Naja haje
(H) Naja nivea
(H) Naja melanoleuca
(H) Naja annulata
(H) Naja multifasciata
(H) Naja ashei
(H) Naja mossambica
(H) Naja nigricollis
(H) Naja katiensis
(EH) Naja nubiae
(H) Naja pallida
(BC) Naja atra
(B) Naja kaouthia
(B) Naja siamensis
(B) Naja sumatrana
(AD) Naja naja
(H) Hemachatus haemachatus
(H) Aspidelaps scutatus
(E) Walterinnesia aegyptia
(H) Dendroaspis angusticeps
(ABD) Ophiophagus hannah
(H) Elapsoidea semiannulata
(B) Hemibungarus calligaster
(D) Calliophis bibroni
(BC) Sinomicrurus macclellandi
(B) Sinomicrurus kelloggii
(B) Sinomicrurus japonicus
(I) Micrurus surinamensis
(I) Micruroides euryxanthus
(D) Calliophis castoe
(D) Calliophis nigrescens
(D) Calliophis melanurus
(B) Calliophis bivirgata
(B) Calliophis intestinalis

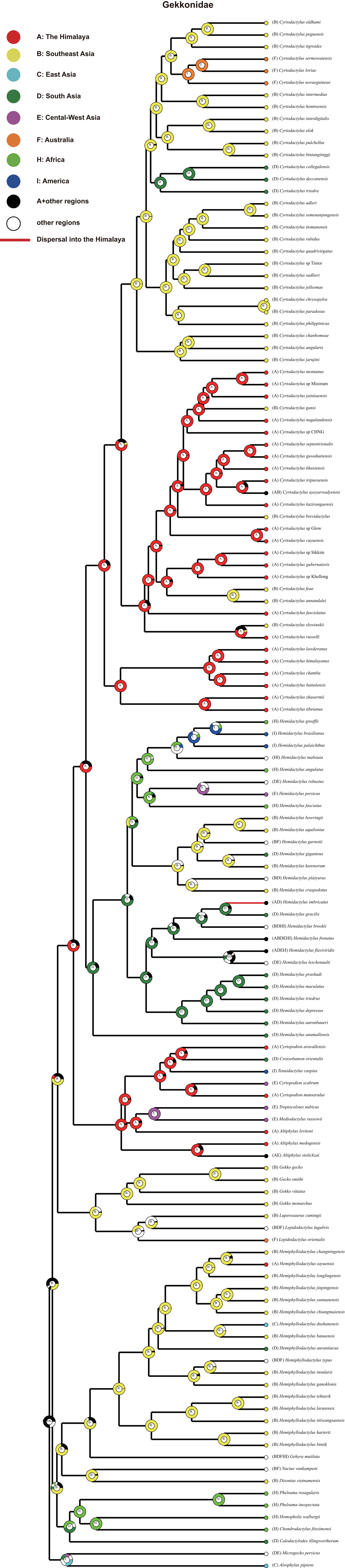
Gekkonidae
A: The Himalaya
B: Southeast Asia
C: East Asia
D: South Asia
E: Cental-West Asia
F: Australia
H: Africa
I: America
A+other regions
other regions
Dispersal into the Himalaya
(B) Cyrtodactylus oldhami
(B) Cyrtodactylus peguensis
(B) Cyrtodactylus tigroides
(F) Cyrtodactylus sermowaiensis
(F) Cyrtodactylus loriae
(F) Cyrtodactylus novaeguineae
(B) Cyrtodactylus intermedius
(B) Cyrtodactylus hontreensis
(B) Cyrtodactylus interdigitalis
(B) Cyrtodactylus elok
(B) Cyrtodactylus pulchellus
(B) Cyrtodactylus bintangtinggi
(D) Cyrtodactylus collegalensis
(D) Cyrtodactylus deccanensis
(D) Cyrtodactylus triedra
(B) Cyrtodactylus adleri
(B) Cyrtodactylus semenanjungensis
(B) Cyrtodactylus tiomanensis
(B) Cyrtodactylus rubidus
(B) Cyrtodactylus quadrivirgatus
(B) Cyrtodactylus sp Timor
(B) Cyrtodactylus sadlieri
(B) Cyrtodactylus jellesmae
(B) Cyrtodactylus chrysopylos
(B) Cyrtodactylus paradoxus
(B) Cyrtodactylus philippinicus
(B) Cyrtodactylus chanhomeae
(B) Cyrtodactylus angularis
(B) Cyrtodactylus jarujini
(A) Cyrtodactylus montanus
(A) Cyrtodactylus sp Mizoram
(A) Cyrtodactylus jaintiaensis
(B) Cyrtodactylus gansi
(A) Cyrtodactylus nagalandensis
(A) Cyrtodactylus sp CHNG
(A) Cyrtodactylus septentrionalis
(A) Cyrtodactylus guwahatiensis
(A) Cyrtodactylus khasiensis
(A) Cyrtodactylus tripuraensis
(AB) Cyrtodactylus ayeyarwadyensis
(A) Cyrtodactylus kazirangaensis
(B) Cyrtodactylus brevidactylus
(A) Cyrtodactylus sp Glow
(A) Cyrtodactylus cayuensis
(A) Cyrtodactylus sp Sikkim
(A) Cyrtodactylus gubernatoris
(A) Cyrtodactylus sp Khellong
(B) Cyrtodactylus feae
(B) Cyrtodactylus annandalei
(A) Cyrtodactylus fasciolatus
(B) Cyrtodactylus slowinskii
(A) Cyrtodactylus russelli
(A) Cyrtodactylus lawderanus
(A) Cyrtodactylus himalayanus
(A) Cyrtodactylus chamba
(A) Cyrtodactylus battalensis
(A) Cyrtodactylus zhaoermii
(A) Cyrtodactylus tibetanus
(H) Hemidactylus greeffii
(I) Hemidactylus brasilianus
(I) Hemidactylus palaichthus
(HI) Hemidactylus mabouia
(H) Hemidactylus angulatus
(DE) Hemidactylus robustus
(E) Hemidactylus persicus
(H) Hemidactylus fasciatus
(B) Hemidactylus bowringii
(B) Hemidactylus aquilonius
(BF) Hemidactylus garnotii
(D) Hemidactylus giganteus
(B) Hemidactylus karenorum
(BD) Hemidactylus platyurus
(B) Hemidactylus craspedotus
(AD) Hemidactylus imbricatus
(D) Hemidactylus gracilis
(BDHI) Hemidactylus brookii
(ABDEHI) Hemidactylus frenatus
(ADEH) Hemidactylus flaviviridis
(DE) Hemidactylus leschenaulti
(D) Hemidactylus prashadi
(D) Hemidactylus maculatus
(D) Hemidactylus triedrus
(D) Hemidactylus depressus
(D) Hemidactylus aaronbaueri
(D) Hemidactylus anamallensis
(A) Cyrtopodion aravallensis
(D) Crossobamon orientalis
(I) Tenuidactylus caspius
(E) Cyrtopodion scabrum
(A) Cyrtopodion mansarulus
(E) Tropiocolotes nubicus
(E) Mediodactylus russowii
(A) Altiphylax levitoni
(A) Altiphylax medogensis
(AE) Altiphylax stolickzai
(B) Gekko gecko
(B) Gecko smithi
(B) Gekko vittatus
(B) Gekko monarchus
(B) Luperosaurus cumingii
(BDF) Lepidodactylus lugubris
(F) Lepidodactylus orientalis
(B) Hemiphyllodactylus changningensis
(A) Hemiphyllodactylus zayuensis
(B) Hemiphyllodactylus longlingensis
(B) Hemiphyllodactylus jinpingensis
(B) Hemiphyllodactylus yunnanensis
(B) Hemiphyllodactylus chiangmaiensis
(C) Hemiphyllodactylus dushanensis
(B) Hemiphyllodactylus banaensis
(D) Hemiphyllodactylus aurantiacus
(BDF) Hemiphyllodactylus typus
(B) Hemiphyllodactylus insularis
(B) Hemiphyllodactylus ganoklonis
(B) Hemiphyllodactylus tehtarik
(B) Hemiphyllodactylus larutensis
(B) Hemiphyllodactylus titiwangsaensis
(B) Hemiphyllodactylus harterti
(B) Hemiphyllodactylus bintik
(BDFHI) Gehyra mutilata
(BF) Nactus vankampeni
(B) Dixonius vietnamensis
(H) Phelsuma rosagularis
(H) Phelsuma inexpectata
(H) Homopholis walbergii
(H) Chondrodactylus fitzsimonsi
(D) Calodactylodes illingworthorum
(DE) Microgecko persicus
(C) Alsophylax pipiens

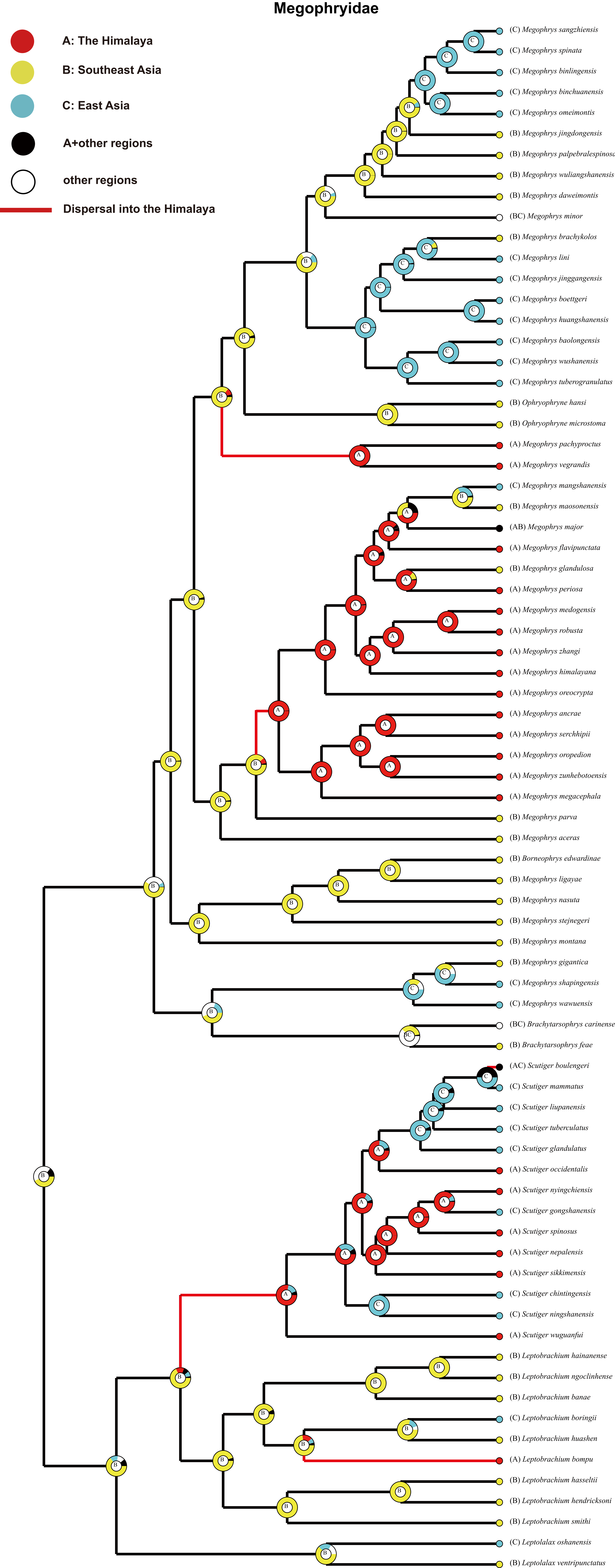
Megophryidae
A: The Himalaya
B: Southeast Asia
C: East Asia
A+other regions
other regions
Dispersal into the Himalaya
(C) Megophrys sangzhiensis
(C) Megophrys spinata
(C) Megophrys binlingensis
(C) Megophrys binchuanensis
(C) Megophrys omeimontis
(B) Megophrys jingdongensis
(B) Megophrys palpebralespinosa
(B) Megophrys wuliangshanensis
(B) Megophrys daweimontis
(BC) Megophrys minor
(B) Megophrys brachykolos
(C) Megophrys lini
(C) Megophrys jinggangensis
(C) Megophrys boettgeri
(C) Megophrys huangshanensis
(C) Megophrys baolongensis
(C) Megophrys wushanensis
(C) Megophrys tuberogranulatus
(B) Ophryophryne hansi
(B) Ophryophryne microstoma
(A) Megophrys pachyproctus
(A) Megophrys vegrandis
(C) Megophrys mangshanensis
(B) Megophrys maosonensis
(AB) Megophrys major
(A) Megophrys flavipunctata
(B) Megophrys glandulosa
(A) Megophrys periosa
(A) Megophrys medogensis
(A) Megophrys robusta
(A) Megophrys zhangi
(A) Megophrys himalayana
(A) Megophrys oreocrypta
(A) Megophrys ancrae
(A) Megophrys serchhipii
(A) Megophrys oropedion
(A) Megophrys zunhebotoensis
(A) Megophrys megacephala
(B) Megophrys parva
(B) Megophrys aceras
(B) Borneophrys edwardinae
(B) Megophrys ligayae
(B) Megophrys nasuta
(B) Megophrys stejnegeri
(B) Megophrys montana
(B) Megophrys gigantica
(C) Megophrys shapingensis
(C) Megophrys wawuensis
(BC) Brachytarsophrys carinense
(B) Brachytarsophrys feae
(AC) Scutiger boulengeri
(C) Scutiger mammatus
(C) Scutiger liupanensis
(C) Scutiger tuberculatus
(C) Scutiger glandulatus
(A) Scutiger occidentalis
(A) Scutiger nyingchiensis
(C) Scutiger gongshanensis
(A) Scutiger spinosus
(A) Scutiger nepalensis
(A) Scutiger sikkimensis
(C) Scutiger chintingensis
(C) Scutiger ningshanensis
(A) Scutiger wuguanfui
(B) Leptobrachium hainanense
(B) Leptobrachium ngoclinhense
(B) Leptobrachium banae
(C) Leptobrachium boringii
(B) Leptobrachium huashen
(A) Leptobrachium bompu
(B) Leptobrachium hasseltii
(B) Leptobrachium hendricksoni
(B) Leptobrachium smithi
(C) Leptolalax oshanensis
(B) Leptolalax ventripunctatus

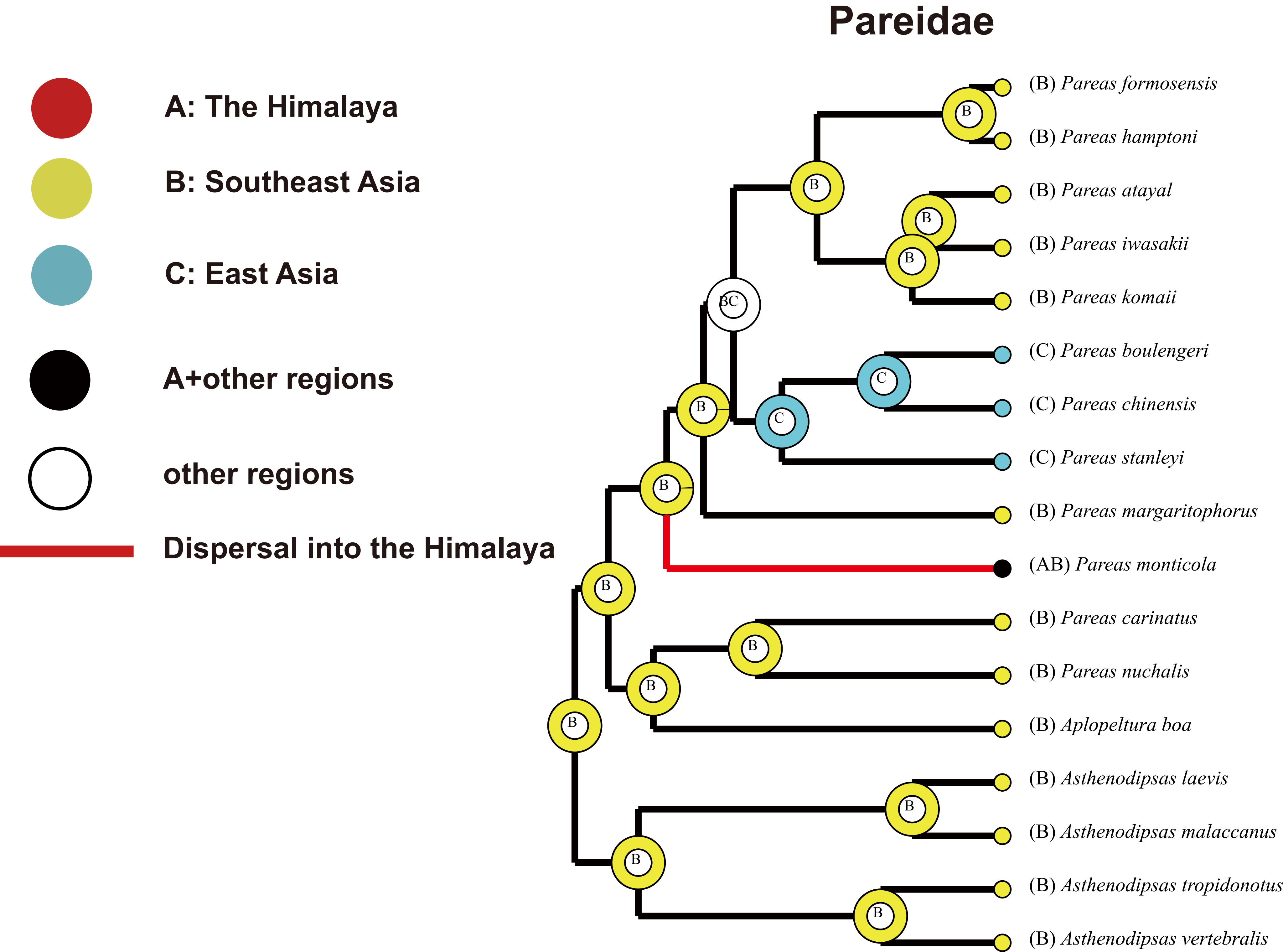

Pareidae
A: The Himalaya
B: Southeast Asia
C: East Asia
A+other regions
other regions
Dispersal into the Himalaya
(B) Pareas formosensis
(B) Pareas hamptoni
(B) Pareas atayal
(B) Pareas iwasakii
(B) Pareas komaii
(C) Pareas boulengeri
(C) Pareas chinensis
(C) Pareas stanleyi
(B) Pareas margaritophorus
(AB) Pareas monticola
(B) Pareas carinatus
(B) Pareas nuchalis
(B) Aplopeltura boa
(B) Asthenodipsas laevis
(B) Asthenodipsas malaccanus
(B) Asthenodipsas tropidonotus
(B) Asthenodipsas vertebralis

Ranidae

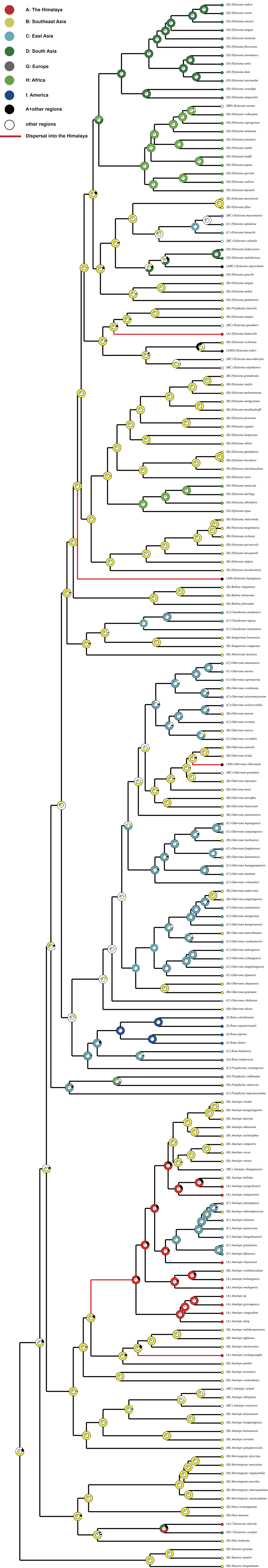
A: The Himalaya
B: Southeast Asia
C: East Asia
D: South Asia
G: Europe
H: Africa
I: America
A+other regions
other regions
Dispersal into the Himalaya
(D) Hylarana indica
(D) Hylarana sreeni
(D) Hylarana caesari
(D) Hylarana magna
(D) Hylarana montana
(D) Hylarana flavescens
(D) Hylarana aurantiaca
(D) Hylarana urbis
(D) Hylarana doni
(D) Hylarana intermedia
(D) Hylarana serendipi
(D) Hylarana temporalis
(BH) Hylarana aurata
(H) Hylarana volkerjane
(H) Hylarana supragrisea
(H) Hylarana milneana
(H) Hylarana jimiensis
(H) Hylarana arfaki
(H) Hylarana kreffti
(H) Hylarana papua
(H) Hylarana garritor
(H) Hylarana waliesa
(H) Hylarana daemeli
(B) Hylarana mortenseni
(B) Hylarana faber
(BC) Hylarana maosonensis
(C) Hylarana spinulosa
(C) Hylarana latouchii
(BC) Hylarana cubitalis
(D) Hylarana bahuvistara
(D) Hylarana malabaricus
(ABC) Hylarana nigrovittata
(D) Hylarana gracilis
(B) Hylarana attigua
(B) Hylarana milleti
(H) Hylarana galamensis
(B) Pelophylax lateralis
(B) Hylarana miopus
(BC) Hylarana guentheri
(A) Hylarana humeralis
(B) Hylarana erythraea
(ABD) Hylarana tytleri
(BC) Hylarana macrodactyla
(BC) Hylarana taipehensis
(B) Hylarana grandocula
(B) Hylarana similis
(B) Hylarana melanomenta
(B) Hylarana mangyanum
(B) Hylarana moellendorffi
(B) Hylarana picturata
(B) Hylarana signata
(B) Hylarana banjarana
(B) Hylarana siberu
(B) Hylarana glandulosa
(B) Hylarana baramica
(B) Hylarana laterimaculata
(B) Hylarana rawa
(H) Hylarana amnicola
(H) Hylarana darlingi
(H) Hylarana albolabris
(H) Hylarana lepus
(B) Hylarana chalconota
(B) Hylarana megalonesa
(B) Hylarana eschatia
(B) Hylarana parvaccola
(B) Hylarana mocquardi
(B) Hylarana rufipes
(B) Hylarana nicobariensis
(AB) Hylarana leptoglossa
(B) Babina chapaensis
(B) Babina okinavana
(B) Babina pleuraden
(C) Glandirana emeljanovi
(C) Glandirana rugosa
(C) Glandirana tientaiensis
(B) Sanguirana luzonensis
(B) Sanguirana sanguinea
(B) Abavorana luctuosa
(C) Odorrana amamiensis
(C) Odorrana narina
(C) Odorrana supranarina
(B) Odorrana swinhoana
(C) Odorrana utsunomiyaorum
(C) Odorrana exiliversabilis
(B) Odorrana nasuta
(C) Odorrana tormota
(B) Odorrana nasica
(C) Odorrana versabilis
(B) Odorrana aureola
(B) Odorrana livida
(AB) Odorrana chloronota
(BC) Odorrana graminea
(B) Odorrana leporipes
(B) Odorrana hosii
(B) Odorrana morafkai
(B) Odorrana banaorum
(B) Odorrana tiannanensis
(C) Odorrana hejiangensis
(C) Odorrana nanjiangensis
(B) Odorrana bacboensis
(C) Odorrana fengkaiensis
(B) Odorrana hainanensis
(C) Odorrana huanggangensis
(C) Odorrana tianmuii
(C) Odorrana schmackeri
(B) Odorrana andersonii
(B) Odorrana jingdongensis
(C) Odorrana junlianensis
(C) Odorrana margaretae
(C) Odorrana kuangwuensis
(B) Odorrana mutschmanni
(C) Odorrana wuchuanensis
(C) Odorrana anlungensis
(C) Odorrana yizhangensis
(C) Odorrana lungshengensis
(C) Odorrana lipuensis
(B) Odorrana chapaensis
(B) Odorrana geminata
(C) Odorrana ishikawae
(B) Odorrana absita
(I) Rana catesbeianus
(I) Rana septentrionalis
(I) Rana pipiens
(I) Rana tlaloci
(C) Rana kukunoris
(G) Rana temporaria
(C) Pseudorana weiningensis
(G) Pelophylax ridibundus
(H) Pelophylax saharicus
(C) Pelophylax nigromaculatus
(B) Amolops iriodes
(B) Amolops mengyangensis
(B) Amolops daorum
(B) Amolops akhaorum
(B) Amolops archotaphus
(B) Amolops compotrix
(B) Amolops cucae
(B) Amolops vitreus
(BC) Amolops chunganensis
(B) Amolops bellulus
(A) Amolops nyingchiensis
(A) Amolops aniqiaoensis
(C) Amolops jinjiangensis
(B) Amolops tuberodepressus
(C) Amolops loloensis
(C) Amolops mantzorum
(C) Amolops liangshanensis
(C) Amolops granulosus
(C) Amolops lifanensis
(A) Amolops chayuensis
(B) Amolops viridimaculatus
(A) Amolops beibengensis
(A) Amolops medogensis
(A) Amolops sp
(A) Amolops gyirongensis
(A) Amolops wangyufani
(A) Amolops deng
(B) Amolops indoburmanensis
(B) Amolops afghanus
(B) Amolops marmoratus
(A) Amolops yarlungzangbo
(B) Amolops panhai
(B) Amolops larutensis
(B) Amolops cremnobatus
(BC) Amolops ricketti
(B) Amolops albispinus
(BC) Amolops wuyiensis
(B) Amolops daiyunensis
(B) Amolops hongkongensis
(B) Amolops hainanensis
(B) Amolops torrentis
(B) Amolops spinapectoralis
(B) Meristogenys dyscritus
(B) Meristogenys maryatiae
(B) Meristogenys stigmachilus
(B) Meristogenys poecilus
(B) Meristogenys amoropalamus
(B) Meristogenys stenocephalus
(B) Huia cavitympanum
(B) Huia masonii
(A) Clinotarsus alticola
(D) Clinotarsus curtipes
(B) Huia melasma
(B) Staurois guttatus
(B) Staurois natator
(B) Staurois latopalmatus

Rhacophoridae

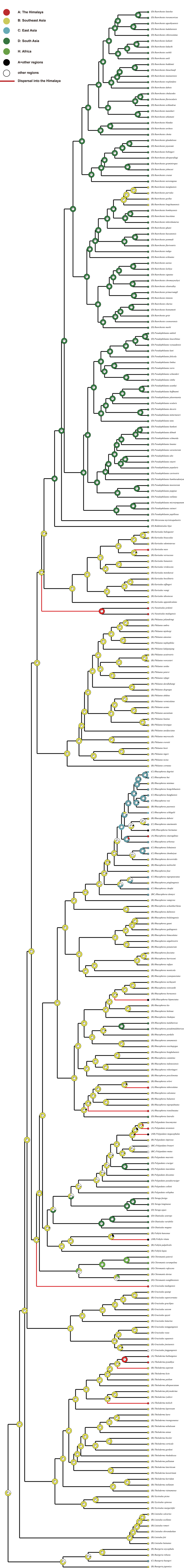

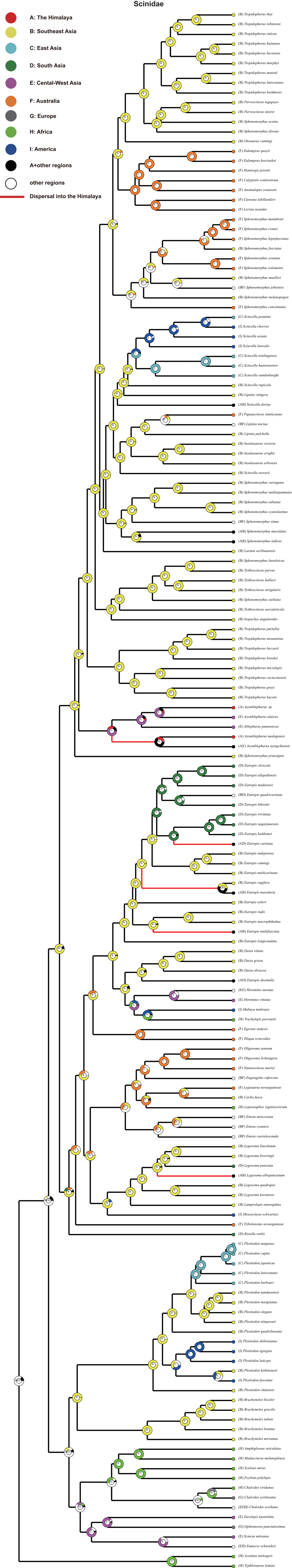
Scinidae
A: The Himalaya
B: Southeast Asia
C: East Asia
D: South Asia
E: Cental-West Asia
F: Australia
G: Europe
H: Africa
I: America
A+other regions
other regions
Dispersal into the Himalaya
(B) Tropidophorus thai
(B) Tropidophorus robinsoni
(B) Tropidophorus sinicus
(B) Tropidophorus hainanus
(B) Tropidophorus baviensis
(B) Tropidophorus murphyi
(B) Tropidophorus matsuii
(B) Tropidophorus latiscutatus
(B) Tropidophorus berdmorei
(B) Parvoscincus tagapayo
(B) Parvoscincus steerei
(B) Sphenomorphus acutus
(B) Sphenomorphus diwata
(B) Otosaurus cumingi
(F) Eulamprus quoyii
(F) Eulamprus kosciuskoi
(F) Hemiergis peronii
(F) Calyptotis scutirostrum
(F) Anomalopus swansoni
(F) Ctenotus labillardieri
(F) Lerista neander
(F) Sphenomorphus maindroni
(F) Sphenomorphus cranei
(F) Sphenomorphus leptofasciatus
(B) Sphenomorphus fasciatus
(F) Sphenomorphus scutatus
(F) Sphenomorphus solomonis
(B) Sphenomorphus muelleri
(BF) Sphenomorphus jobiensis
(B) Sphenomorphus melanopogon
(F) Sphenomorphus concinnatus
(C) Scincella potanini
(I) Scincella cherriei
(I) Scincella assata
(I) Scincella lateralis
(C) Scincella tsinlingensis
(C) Scincella huanrenensis
(C) Scincella vandenburghi
(B) Scincella rupicola
(B) Lipinia vittigera
(AB) Scincella doriae
(F) Papuascincus stanleyanus
(BF) Lipinia noctua
(B) Lipinia pulchella
(B) Insulasaurus victoria
(B) Insulasaurus wrighti
(B) Insulasaurus arborens
(B) Scincella reevesii
(B) Sphenomorphus variegatus
(B) Sphenomorphus multisquamatus
(B) Sphenomorphus sabanus
(B) Sphenomorphus cyanolaemus
(BF) Sphenomorphus simus
(AB) Sphenomorphus maculatus
(AB) Sphenomorphus indicus
(B) Larutia seribuatensis
(B) Sphenomorphus buenloicus
(B) Tytthoscincus parvus
(B) Tytthoscincus hallieri
(B) Tytthoscincus atrigularis
(B) Sphenomorphus stellatus
(B) Tytthoscincus aesculeticola
(B) Isopachys anguinoides
(B) Tropidophorus partelloi
(B) Tropidophorus misaminius
(B) Tropidophorus beccarii
(B) Tropidophorus brookei
(B) Tropidophorus microlepis
(B) Tropidophorus cocincinensis
(B) Tropidophorus grayi
(B) Tropidophorus baconi
(A) Asymblepharus sp
(E) Asymblepharus alaicus
(E) Ablepharus pannonicus
(A) Asymblepharus madogensis
(AE) Asymblepharus nyingchiensis
(B) Sphenomorphus praesignis
(D) Eutropis clivicola
(D) Eutropis allapallensis
(D) Eutropis madaraszi
(BD) Eutropis quadricarinata
(D) Eutropis bibronii
(D) Eutropis trivittata
(D) Eutropis nagarjunensis
(D) Eutropis beddomei
(AD) Eutropis carinata
(B) Eutropis indeprensa
(B) Eutropis cumingi
(B) Eutropis multicarinata
(B) Eutropis rugifera
(AB) Eutropis macularia
(B) Eutropis tytleri
(B) Eutropis rudis
(B) Eutropis macrophthalma
(AB) Eutropis multifasciata
(B) Eutropis longicaudata
(B) Dasia vittata
(B) Dasia grisea
(B) Dasia olivacea
(AD) Eutropis dissimilis
(EG) Heremites auratus
(E) Heremites vittatus
(I) Mabuya mabouya
(H) Trachylepis perrotetii
(F) Egernia stokesii
(F) Tiliqua scincoides
(F) Oligosoma aeneum
(F) Oligosoma lichenigera
(F) Nannoscincus mariei
(BF) Eugongylus rufescens
(F) Lygisaurus novaeguineae
(B) Carlia fusca
(H) Leptosiaphos vigintiserierum
(BF) Emoia atrocostata
(BF) Emoia cyanura
(BF) Emoia caeruleocauda
(B) Lygosoma lineolatum
(B) Lygosoma bowringii
(B) Lygosoma punctata
(AB) Lygosoma albopunctatum
(B) Lygosoma quadrupes
(B) Lygosoma koratense
(B) Lamprolepis smaragdina
(H) Mesoscincus schwartzei
(F) Tribolonotus novaeguineae
(D) Ristella rurkii
(C) Plestiodon tunganus
(C) Plestiodon capito
(C) Plestiodon japonicus
(C) Plestiodon latiscutatus
(C) Plestiodon barbouri
(B) Plestiodon tamdaoensis
(B) Plestiodon marginatus
(B) Plestiodon elegans
(B) Plestiodon stimpsonii
(B) Plestiodon quadrilineatus
(I) Plestiodon skiltonianus
(I) Plestiodon egregius
(I) Plestiodon laticeps
(I) Plestiodon kishinouyei
(I) Plestiodon fasciatus
(B) Plestiodon chinensis
(B) Brachymeles bicolor
(B) Brachymeles gracilis
(B) Brachymeles talinis
(B) Brachymeles bonitae
(B) Brachymeles miriamae
(H) Amphiglossus reticulatus
(H) Madascincus melanopleura
(H) Scelotes mirus
(H) Feylinia polylepis
(H) Chalcides viridanus
(G) Chalcides sexlineatus
(EGH) Chalcides ocellatus
(E) Eurylepis taeniolata
(G) Ophiomorus punctatissimus
(E) Scincus mitranus
(EH) Eumeces schneideri
(H) Acontias meleagris
(H) Typhlosaurus lomiae

Viperidae

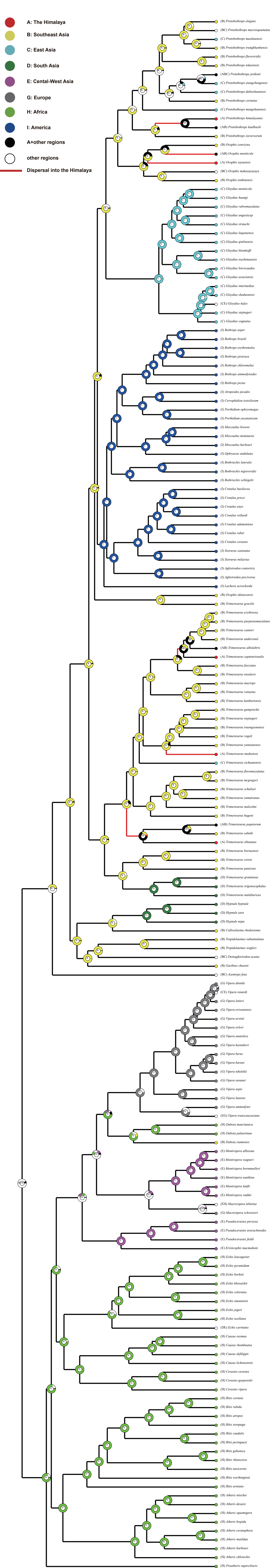
A: The Himalaya
B: Southeast Asia
C: East Asia
D: South Asia
E: Cental-West Asia
G: Europe
H: Africa
I: America
A+other regions
other regions
Dispersal into the Himalaya

Data S5.

Sequences information in this study divided by family. Sequences obtained newly in this study are marked in bold.

Ranidae

Species	16s	COI	ND3	ND2	12s
Abavorana_luctuosa	KY982539	-	-	-	KF477635
Amnirana_albolabris	KR264062	-	-	-	-
Amnirana_amnicola	KX289630	-	-	-	-
Amnirana_darlingi	KR264121	-	-	-	-
Amnirana_galamensis	KF991278	-	-	-	-
Amnirana_lepus	KR264067	-	-	-	-
Amnirana_nicobariensis	AB530582	-	-	-	KP298048
Amolops_afghanus	MN953654	-	-	MN958713	-
Amolops_akhaorum	FJ417158	-	-	FJ417207	-
Amolops_albispinus	KX507312	KX507332	-	-	-
Amolops_aniqiaoensis	MN953655	KU243073	-	MN958714	-
Amolops_aniqiaoensis	MN953656	-	-	MN958715	-
Amolops_archotaphus	MN953659	-	-	MN958718	-
Amolops_beibengensis	MN953662	-	-	MN958721	-
Amolops_bellulus	DQ204473	KU243079	FJ417225	FJ417176	DQ204429
Amolops_chayuensis	MN953666	KU243075	-	MN958725	-
Amolops_chunganensis	KX507310	KX507330	KX645666	KX645666	KX645666
Amolops_compotrix	FJ417141	-	FJ417239	FJ417190	-
Amolops_cremnobatus	MN953672	-	FJ417241	MN958730	-
Amolops_cucae	FJ417145	-	FJ417243	FJ417194	-
Amolops_daiyunensis	**MW023599**	KX507328	-	**MW111442**	MG991855
Amolops_daorum	MN953678	-	FJ417249	MN958736	AF206101
Amolops_granulosus	MN953680	-	-	MN958738	DQ204436
Amolops_gyirongensis	MN953682	-	-	MN958739	-
Amolops_gyirongensis	MN953683	-	-	MN958740	-
Amolops_hainanensis	MN953688	KX507342	-	MN958745	MG991858
Amolops_hongkongensis	MN953689	MG991921	-	MN958746	MG991861
Amolops_indoburmanensis	MN953692	-	-	MN958749	MG909644
Amolops_deng	MN953695	-	-	MN958752	-
Amolops_deng	**MW023600**	**-**	**-**	**MW111443**	**-**
Amolops_iriodes	FJ417152	-	FJ417250	FJ417201	-
Amolops_jinjiangensis	MN953700	-	-	MN958757	EF453726
Amolops_larutensis	AB211484	-	-	-	AB211461
Amolops_liangshanensis	EF453743	-	-	MN958812	EF453728
Amolops_lifanensis	MN953702	JN700797	-	MN958760	-
Amolops_loloensis	MN953757	NC_029250	NC_029250	MN958814	DQ204439
Amolops_mantzorum	**MW023601**	NC_024180	FJ417253	**MW111444**	NC_024180
Amolops_marmoratus	MN953708	MG935420	EU861515	MN958766	MG909645
Amolops_medogensis	MN953710	KU243076	-	MN958768	-
Amolops_medogensis	MN953711	-	-	MN958769	-
Amolops_mengyangensis	KR827703	KR087618	-	-	-

Amolops_nyingchiensis	MN953715	KU243071	-	MN958773	-
Amolops_nyingchiensis	MN953716	-	-	MN958774	-
Amolops_panhai	MN953720	KR087621	-	MN958778	MG909646
Amolops_ricketti	**MW023602**	NC_023949	NC_023949	**MW111445**	NC_023949
Amolops_sp	**MW023603**	-	-	**MW111446**	-
Amolops_spinapectoralis	MN953769	-	FJ417259	MN958826	DQ204444
Amolops_torrentis	EF453744	MG991932	-	MN958784	MG991872
Amolops_tuberodepressus	MN953729	KR559270	FJ417260	MN958786	KR559270
Amolops_viridimaculatus	MN953731	-	-	MN958788	DQ204446
Amolops_vitreus	FJ417163	-	FJ417261	FJ417212	-
Amolops_wangyufani	MN953741	-	-	MN958797	-
Amolops_wangyufani	MN953740	-	-	MN958796	-
Amolops_wuyiensis	**MW023604**	KX507325	KM282625	**MW111447**	KM282625
Amolops_yarlungzangbo	MN953744	-	-	MN958800	-
Amolops_yarlungzangbo	MN953745	-	-	MN958801	-
Babina_chapaensis	KU840598	KR087625	-	-	KU840523
Babina_okinavana	NC_022872	NC_022872	NC_022872	NC_022872	NC_022872
Babina_pleuraden	KR264059	HQ395351	KF771303	-	-
Chalcorana_chalconota	AB530588	-	-	-	-
Chalcorana_eschatia	KR264083	-	-	-	-
Chalcorana_megalonesa	KR264085	-	-	-	-
Chalcorana_mocquardi	KR264100	-	-	-	-
Chalcorana_parvaccola	KR264082	-	-	-	-
Chalcorana_rufipes	KR264081	-	-	-	-
Clinotarsus_alticola	MG909604	-	-	-	MG909673
Clinotarsus_curtipes	KM069013	KM069121	-	-	-
Glandirana_emeljanovi	KF771343	KF771343	KF771343	KF771343	KF771343
Glandirana_rugosa	KF771341	KF771341	KF771341	KF771341	KF771341
Glandirana_tientaiensis	KF771342	KF771342	KF771342	KF771342	KF771342
Huia_cavitympanum	KU840576	-	FJ417216	FJ417167	MG909681
Huia_cavitympanum	AB211489	-	-	-	AB211466
Huia_masonii	KP298037	-	-	-	KP298037
Huia_melasma	MG909601	-	-	-	MG909675
Humerana_humeralis	KU589224	-	-	-	-
Humerana_miopus	KR827778	KR087686	-	-	-
Hydrophylax_bahuvistara	KT334413	-	-	-	-
Hydrophylax_gracilis	KM068934	KM069041	-	-	-
Hydrophylax_leptoglossa	AB530527	-	-	-	-
Hydrophylax_malabaricus	KM068968	KM069075	-	-	-
Hylarana_attigua	EU754872	-	-	-	-
Hylarana_erythraea	KR827794	-	-	-	-
Hylarana_latouchii	KF771284	KF771284	-	-	-
Hylarana_macrodactyla	KR264120	-	-	-	-
Hylarana_taipehensis	KR264041	-	-	-	-

Hylarana_tytleri	KR264119	-	-	-	-
Indosylvirana_caesari	KM068915	KM069022	-	-	-
Indosylvirana_aurantiaca	KM068907	KM069014	-	-	-
Indosylvirana_doni	KM068922	KM069029	-	-	-
Indosylvirana_flavescens	KM068930	KM069037	-	-	-
Indosylvirana_indica	KM068943	KM069050	-	-	-
Indosylvirana_intermedia	KM068956	KM069063	-	-	-
Indosylvirana_magna	KM068964	KM069071	-	-	-
Indosylvirana_milleti	KR827825	-	-	-	-
Indosylvirana_montana	KM068972	KM069079	-	-	-
Indosylvirana_serendipi	KM068979	KM069086	-	-	-
Indosylvirana_sreeni	KM068987	KM069095	-	-	-
Indosylvirana_temporalis	KM069000	KM069108	-	-	-
Indosylvirana_urbis	KM069006	KM069114	-	-	-
Meristogenys_amoropalamus	AB526619	-	-	AB526619	AB360011
Meristogenys_dyscritus	AB526616	-	-	AB526616	AB526616
Meristogenys_maryatiae	AB526611	-	-	AB526611	AB360046
Meristogenys_poecilus	AB526610	-	-	-	AB526610
Meristogenys_stenocephalus	AB526612	-	-	-	AB526612
Meristogenys_stigmachilus	AB526614	-	-	-	AB526614
Odorrana_absita	EU861542	-	EU861516	EU861568	-
Odorrana_amamiensis	AB200947	-	-	-	AB200923
Odorrana_andersonii	KF185057	JN700826	KF771312	-	DQ359965
Odorrana_anlungensis	KF185049	-	-	-	KF185013
Odorrana_aureola	DQ650568	-	DQ650437	DQ650504	-
Odorrana_bacboensis	DQ650569	-	-	-	-
Odorrana_banaorum	DQ650586	-	DQ650455	DQ650522	-
Odorrana_chapaensis	KX893901	KR087835	EU861527	EU861579	DQ204431
Odorrana_chloronota	DQ283394	-	-	-	DQ283394
Odorrana_exiliversabilis	KF185056	-	-	-	KF185020
Odorrana_fengkaiensis	KT315375	-	-	-	KT315354
Odorrana_geminata	EU861546	-	EU861520	EU861572	-
Odorrana_graminea	KF185038	-	-	-	KF185002
Odorrana_hainanensis	NC_034984	NC_034984	NC_034984	NC_034984	NC_034984
Odorrana_hejiangensis	KF185052	-	-	-	KF185016
Odorrana_hosii	MG935960	MG935666	DQ650471	DQ650539	KU840535
Odorrana_huanggangensis	KF185059	-	-	-	KF185023
Odorrana_ishikawae	AB511282	AB511282	AB511282	AB511282	AB511282
Odorrana_jingdongensis	KF185050	KR087840	-	-	KF185014
Odorrana_jingdongensis	FJ417120	-	FJ417218	FJ417169	-
Odorrana_junlianensis	KF185058	-	-	-	KF185022
Odorrana_kuangwuensis	KF185034	-	-	-	KF184998
Odorrana_leporipes	KF185036	-	-	-	KF185000
Odorrana_lipuensis	KM388699	-	-	-	-

Odorrana_livida	DQ650615	-	-	-	-
Odorrana_lungshengensis	KF185054	-	-	-	KF185018
Odorrana_margaretae	EU861566	KR087843	EU861540	EU861592	KT315371
Odorrana_morafkai	DQ650632	-	DQ650498	DQ650562	KU840526
Odorrana_mutschmanni	KU356765	-	-	-	KU356761
Odorrana_nanjiangensis	KF185042	-	-	-	KF185006
Odorrana_narina	AB511287	-	-	-	AB511287
Odorrana_nasica	DQ283345	-	-	-	DQ283345
Odorrana_nasuta	KF185053	-	-	-	KF185017
Odorrana_schmackeri	KF185047	KP732086	KP732086	KP732086	KF185011
Odorrana_supranarina	AB200950	-	-	-	AB200926
Odorrana_swinhoana	AB200953	-	-	-	AB200929
Odorrana_tianmuii	KF185040	-	-	-	KF185004
Odorrana_tiannanensis	EF453751	KR087851	-	-	EF453736
Odorrana_tormota	DQ835616	DQ835616	DQ835616	DQ835616	DQ835616
Odorrana_utsunomiyaorum	AB200952	-	-	-	AB200928
Odorrana_versabilis	KF185055	-	-	-	KF185019
Odorrana_wuchuanensis	KF185043	KU680791	KU680791	KU680791	KF185007
Odorrana_yizhangensis	KF185048	-	-	-	KF185012
Papurana__daemeli	KR264056	-	-	-	-
Papurana_arfaki	KR264048	-	-	-	-
Papurana_aurata	KR264101	-	-	-	-
Papurana_garritor	KR264042	-	-	-	-
Papurana_jimiensis	KR264053	-	-	-	EU004610
Papurana_kreffti	KR264050	-	-	-	-
Papurana_milneana	KR264044	-	-	-	-
Papurana_papua	KR264054	-	-	-	-
Papurana_supragrisea	KR264058	-	-	-	-
Papurana_volkerjane	KR264105	-	-	-	-
Papurana_waliesa	KR264046	-	-	-	-
Pelophylax_lateralis	KF991279	MG935537	-	-	AF206098
Pelophylax_nigromaculatus	KT878718	KT878718	KT878718	KT878718	KT878718
Pelophylax_ridibundus	MH410481	KT879314	MH410361	KJ160682	AB640897
Pelophylax_saharicus	KP177671	KP177812	GU812242	GU812146	-
Pseudorana_weiningensis	DQ359996	-	-	KX269432	KX269217
Pulchrana_banjarana	KF477645	-	-	-	KF477645
Pulchrana_baramica	KF052066	-	-	-	-
Pulchrana_glandulosa	AB719226	-	-	-	AB719209
Pulchrana_grandocula	KF477662	-	-	-	KF477662
Pulchrana_laterimaculata	AB719228	-	-	-	AB719211
Pulchrana_mangyanum	KF477685	-	-	-	KF477685
Pulchrana_melanomenta	KF477692	-	-	-	KF477692
Pulchrana_moellendorffi	KF477696	-	-	-	KF477696
Pulchrana_picturata	KR264070	-	-	-	-

Pulchrana_rawa	AB719222	-	-	-	AB719205
Pulchrana_siberu	KF477741	-	-	-	KF477741
Pulchrana_signata	KF052074	-	-	-	-
Pulchrana_similis	KR264078	-	-	-	-
Rana_catesbeianus	NC_022696	NC_022696	NC_022696	NC_022696	NC_022696
Rana_kukunoris	KX269185	NC_035804	NC_035804	KX269401	KX269185
Rana_pipiens	X86284	EF525892	-	-	Y10945
Rana_septentrionalis	KX269179	EF525899	-	AY206487	KX269179
Rana_temporaria	AB058882	KP697915	-	AF314018	AB058864
Rana_tlaloci	AY779234	-	-	KX269393	AY779234
Sanguirana_luzonensis	KT881700	-	-	-	KP298050
Sanguirana_sanguinea	KP298051	-	-	-	KP298051
Staurois_guttatus	MG909607	-	-	-	MG909682
Staurois_latopalmatus	FJ417117	-	FJ417215	FJ417166	-
Staurois_natator	DQ347312	-	-	-	-
Sylvirana_cubitalis	KR264077	-	-	-	-
Sylvirana_faber	KR827803	KR087706	-	-	-
Sylvirana_guentheri	NC_024748	NC_024748	NC_024748	NC_024748	NC_024748
Sylvirana_maosonensis	KR827809	-	-	-	-
Sylvirana_mortenseni	MG606638	-	MG607315	MG606971	KU840542
Sylvirana_nigrovittata	KF739002	-	-	-	-
Sylvirana_spinulosa	KR264093	-	-	-	-
**Outgroup**					
Buergeria_oxycephalus	KU244359	KU244459	-	-	-
Cacosternum_magnaglandiferus	KJ461733	-	-	DQ251462	DQ022321
Conraua_crassipes	DQ022355	KY079583	-	-	DQ022324
Cornufer_guentheri	KP298021	AY883979	-	-	DQ283198
Mantidactylus_guttulatus	FJ559237	KF611529	-	-	-
Phrynomantis_annectens	AF215377.2	EF396060	-	-	-
Odontobatrachus_natator	KP284863	-	-	-	KP005222
Phrynodon_sandersoni	DQ283083	-	-	-	DQ283083
Ptychadena_cooperi	-	KF380535	-	-	-
Quasipaa_delacouri	EU979810	-	-	-	EU979664-EU979750
Kurixalus_verrucosus	KX554502	KX554568	-	-	KC465825
Theloderma_corticale	KU244365	KU244453	-	-	-
**Species**	**Cytb**	**RAG1**	**Tyr**	**RAG2**	**Rhod**
Abavorana_luctuosa	-	KR264407	KR264482	-	-
Amnirana_albolabris	KR264152	-	-	-	-
Amnirana_amnicola	-	-	-	-	-
Amnirana_darlingi	KR264208	KR264430	KR264506	-	-
Amnirana_galamensis	-	-	-	-	-
Amnirana_lepus	KR264157	KR264390	KR264458	-	-
Amnirana_nicobariensis	KR264189	KU840721	KU840777	-	KU840659

Amolops_afghanus	-	**MW111381**	**MW111304**	-	-
Amolops_akhaorum	-	FJ417291	FJ417324	-	-
Amolops_albispinus	-	-	-	-	-
Amolops_aniqiaoensis	-	**MW111382**	**MW111305**	-	-
Amolops_aniqiaoensis	-	**MW111383**	**MW111306**	-	-
Amolops_archotaphus	-	**MW111384**	**MW111307**	-	-
Amolops_beibengensis	-	**MW111385**	**MW111308**	-	-
Amolops_bellulus	-	FJ417267	FJ417300	-	-
Amolops_chayuensis	-	**MW111386**	**MW111309**	-	-
Amolops_chunganensis	KX645666	**MW111387**	**MW111310**	-	-
Amolops_compotrix	-	EF088235	EU076757	-	-
Amolops_cremnobatus	-	**MW111388**	**MW111311**	-	-
Amolops_cucae	-	FJ417284	FJ417317	-	-
Amolops_daiyunensis	-	**MW111389**	**MW111312**	-	-
Amolops_daorum	-	**MW111390**	**MW111313**	-	-
Amolops_granulosus	-	**MW111391**	**MW111314**	-	-
Amolops_gyirongensis	-	**MW111392**	**MW111315**	-	-
Amolops_gyirongensis	-	**MW111393**	**MW111316**	-	-
Amolops_hainanensis	-	**MW111394**	**MW111317**	DQ019518	KU840677
Amolops_hongkongensis	-	**MW111395**	**MW111318**	-	-
Amolops_indoburmanensis	-	**MW111396**	**MW111319**	-	-
Amolops_deng	-	**MW111397**	**MW111320**	-	-
Amolops_deng	-	**MW111398**	**MW111321**	-	-
Amolops_iriodes	-	FJ417289	FJ417322	-	-
Amolops_jinjiangensis	-	**MW111399**	**MW111322**	-	-
Amolops_larutensis	-	DQ347279	DQ347186	-	-
Amolops_liangshanensis	-	**MW111400**	**MW111323**	-	-
Amolops_lifanensis	-	**MW111401**	**MW111324**	-	-
Amolops_loloensis	NC_029250	**MW111402**	**MW111325**	-	-
Amolops_mantzorum	NC_024180	**MW111403**	**MW111326**	-	-
Amolops_marmoratus	AB259738	**MW111404**	**MW111327**	-	-
Amolops_medogensis	-	**MW111405**	**MW111328**	-	-
Amolops_medogensis	-	**MW111406**	**MW111329**	-	-
Amolops_mengyangensis	-	-	-	-	-
Amolops_nyingchiensis	-	**MW111407**	**MW111330**	-	-
Amolops_nyingchiensis	-	**MW111408**	**MW111331**	-	-
Amolops_panhai	-	**MW111409**	**MW111332**	-	-
Amolops_ricketti	NC_023949	**MW111410**	**MW111333**	-	DQ360009
Amolops_sp	-	**MW111411**	**MW111334**	-	-
Amolops_spinapectoralis	-	**MW111412**	**MW111335**	-	-
Amolops_torrentis	-	**MW111413**	**MW111336**	-	-
Amolops_tuberodepressus	KR559270	**MW111414**	**MW111337**	-	-
Amolops_viridimaculatus	-	**MW111415**	**MW111338**	-	-
Amolops_vitreus	-	FJ417295	FJ417328	-	-

Amolops_wangyufani	-	**MW111416**	**MW111339**	-	-
Amolops_wangyufani	-	**MW111417**	**MW111340**	-	-
Amolops_wuyiensis	KM282625	**MW111418**	**MW111341**	-	-
Amolops_yarlungzangbo	-	**MW111419**	**MW111342**	-	-
Amolops_yarlungzangbo	-	**MW111420**	**MW111343**	-	-
Babina_chapaensis	KR264164	KR264393	DQ282981	-	DQ283987
Babina_okinavana	NC_022872	-	-	-	-
Babina_pleuraden	KR264150	KR264384	KR264453	-	-
Chalcorana_chalconota	-	-	-	-	-
Chalcorana_eschatia	KR264174	KR264402	KR264476	-	-
Chalcorana_megalonesa	-	-	-	-	-
Chalcorana_mocquardi	KR264191	KR264418	KR264494	-	-
Chalcorana_parvaccola	KR264173	KR264401	KR264475	-	-
Chalcorana_rufipes	KR264172	KR264400	KR264474	-	-
Clinotarsus_alticola	-	MG909641	MG909717	-	-
Clinotarsus_curtipes	KM069226	KM069261	AF249180	-	AF249117
Glandirana_emeljanovi	KF771343	-	-	-	-
Glandirana_rugosa	KF771341	-	-	-	-
Glandirana_tientaiensis	KF771342	-	-	-	-
Huia_cavitympanum	-	EF088246	MG909710	-	KU840634
Huia_cavitympanum	-	-	-	-	-
Huia_masonii	-	EF088247	EU076770	-	-
Huia_melasma	-	MG909637	MG909715	-	-
Humerana_humeralis	-	-	-	-	-
Humerana_miopus	KR264171	-	-	-	-
Hydrophylax_bahuvistara	-	-	-	-	-
Hydrophylax_gracilis	KM069149	KM069232	-	-	-
Hydrophylax_leptoglossa	-	-	-	-	-
Hydrophylax_malabaricus	KM069182	KM069242	-	-	-
Hylarana_attigua	-	-	-	-	-
Hylarana_erythraea	-	-	-	-	-
Hylarana_latouchii	-	-	-	-	-
Hylarana_macrodactyla	KR264207	KR264429	KR264505	-	-
Hylarana_taipehensis	KR264132	KR264366	-	-	-
Hylarana_tytleri	KR264206	KR264428	-	-	-
Indosylvirana_caesari	KM069130	KM069228	-	-	-
Indosylvirana_aurantiaca	KM069122	-	-	-	-
Indosylvirana_doni	KM069137	KM069229	-	-	-
Indosylvirana_flavescens	KM069145	KM069230	-	-	-
Indosylvirana_indica	KM069158	KM069235	-	-	-
Indosylvirana_intermedia	KM069170	KM069238	-	-	-
Indosylvirana_magna	KM069178	-	-	-	-
Indosylvirana_milleti	-	-	-	-	-
Indosylvirana_montana	KM069186	KM069245	-	-	-

Indosylvirana_serendipi	KM069193	KM069248	-	-	-
Indosylvirana_sreeni	KM069201	KM069249	-	-	-
Indosylvirana_temporalis	KM069214	KM069254	-	-	-
Indosylvirana_urbis	KM069219	KM069256	-	-	-
Meristogenys_amoropalamus	AB360107	AB526673	-	-	AB360222
Meristogenys_dyscritus	AB526628	AB526670	-	-	AB360216
Meristogenys_maryatiae	AB526623	AB526665	-	-	AB360231
Meristogenys_poecilus	AB526622	AB526664	-	-	-
Meristogenys_stenocephalus	AB526624	AB526666	-	-	-
Meristogenys_stigmachilus	AB526626	AB526668	-	-	AB360235
Odorrana_absita	-	EF088245	EU076768	-	-
Odorrana_amamiensis	-	-	-	-	-
Odorrana_andersonii	KR264154	KR264387	KR264455	-	DQ360018
Odorrana_anlungensis	-	-	-	-	-
Odorrana_aureola	-	-	-	-	-
Odorrana_bacboensis	-	-	-	-	-
Odorrana_banaorum	-	-	-	-	-
Odorrana_chapaensis	-	EF088255	EU076778	-	DQ283992
Odorrana_chloronota	-	-	-	-	-
Odorrana_exiliversabilis	-	-	-	-	-
Odorrana_fengkaiensis	-	-	-	-	-
Odorrana_geminata	-	-	-	-	-
Odorrana_graminea	-	-	-	-	-
Odorrana_hainanensis	NC_034984	KU840725	-	-	KU840664
Odorrana_hejiangensis	-	-	-	-	-
Odorrana_hosii	KR264179	KU840732	KU840785	-	KU840633
Odorrana_huanggangensis	-	-	-	-	-
Odorrana_ishikawae	AB511282	-	-	-	-
Odorrana_jingdongensis	-	-	-	-	-
Odorrana_jingdongensis	-	EF088258	EU076781	-	-
Odorrana_junlianensis	-	-	-	-	-
Odorrana_kuangwuensis	-	-	-	-	-
Odorrana_leporipes	-	-	-	-	-
Odorrana_lipuensis	-	-	-	-	-
Odorrana_livida	-	-	-	-	-
Odorrana_lungshengensis	-	-	-	-	-
Odorrana_margaretae	KU217312	EF088261	EU076785	-	DQ360017
Odorrana_morafkai	-	EF088263	EU076787	-	KU840630
Odorrana_mutschmanni	-	-	-	-	-
Odorrana_nanjiangensis	-	-	-	-	-
Odorrana_narina	-	-	-	-	-
Odorrana_nasica	-	-	-	-	-
Odorrana_nasuta	-	-	-	-	-
Odorrana_schmackeri	KP732086	-	-	-	-

Odorrana_supranarina	-	-	-	-	-
Odorrana_swinhoana	-	-	-	-	-
Odorrana_tianmuii	-	-	-	-	-
Odorrana_tiannanensis	-	-	-	-	-
Odorrana_tormota	DQ835616	EU076750	EU076766	-	-
Odorrana_utsunomiyaorum	-	-	-	-	-
Odorrana_versabilis	-	-	-	-	-
Odorrana_wuchuanensis	KU680791	-	-	-	-
Odorrana_yizhangensis	-	-	-	-	-
Papurana__daemeli	KR264147	KR264381	KR264452	-	-
Papurana_arfaki	KR264139	KR264373	KR264447	-	-
Papurana_aurata	KR264192	KR264419	KR264495	-	-
Papurana_garritor	KR264133	KR264367	KR264441	-	-
Papurana_jimiensis	KR264144	KR264377	-	-	-
Papurana_kreffti	KR264141	KR264375	KR264448	-	-
Papurana_milneana	KR264135	KR264369	KR264443	-	-
Papurana_papua	KR264145	KR264379	KR264450	-	-
Papurana_supragrisea	KR264149	KR264383	-	-	-
Papurana_volkerjane	KR264196	KR264421	KR264498	-	-
Papurana_waliesa	KR264137	KR264371	KR264445	-	-
Pelophylax_lateralis	KF991320	EF088273	EU076800	-	-
Pelophylax_nigromaculatus	KT878718	HQ902533	DQ282932	-	KU840635
Pelophylax_ridibundus	MG575226	-	KT879356	-	-
Pelophylax_saharicus	GU799108	KP177854	KT879338	-	AY148014
Pseudorana_weiningensis	KX269362	KX269582	DQ360050	KX269657	DQ360019
Pulchrana_banjarana	-	-	KF477517	-	-
Pulchrana_baramica	-	-	-	-	-
Pulchrana_glandulosa	-	-	-	-	-
Pulchrana_grandocula	-	-	KF477534	-	-
Pulchrana_laterimaculata	-	-	-	-	-
Pulchrana_mangyanum	-	-	KF477555	-	-
Pulchrana_melanomenta	-	-	KF477562	-	-
Pulchrana_moellendorffi	-	-	KF477566	-	-
Pulchrana_picturata	KR264159	KR264433	KR264461	-	-
Pulchrana_rawa	-	-	-	-	-
Pulchrana_siberu	-	-	KF477591	-	-
Pulchrana_signata	-	-	-	-	-
Pulchrana_similis	KR264168	-	KR264468	-	-
Rana_catesbeianus	NC_022696	AB612037	AB612039	AB612038	-
Rana_kukunoris	KX269332	KX269550	KX269778	KX269625	-
Rana_pipiens	KM396244	-	EU769542	-	DQ283831
Rana_septentrionalis	AY083273	KX269529	KX269757	KX269605	-
Rana_temporaria	MF624355	AY323776	KC800261	AY323803	-
Rana_tlaloci	KX269323	KX269540	KX269768	KX269616	-

Sanguirana_luzonensis	KR264202	KT881670	KR264502	-	DQ347351
Sanguirana_sanguinea	-	KT881675	KT881866	-	DQ347392
Staurois_guttatus	-	MG909642	MG909718	-	KU840657
Staurois_latopalmatus	-	EF088277	EU076805	-	-
Staurois_natator	AB259736	DQ347250	DQ347155	-	DQ347371
Sylvirana_cubitalis	KR264167	KR264396	KR264467	-	-
Sylvirana_faber	-	-	-	-	-
Sylvirana_guentheri	NC_024748	KX208782	KR264440	KX208921	KU840653
Sylvirana_maosonensis	-	-	-	-	-
Sylvirana_mortenseni	KR264166	KU840712	-	-	KU840650
Sylvirana_nigrovittata	-	-	-	-	-
Sylvirana_spinulosa	KR264185	KR264411	KR264487	-	-
**Outgroup**					
Buergeria_oxycephalus	-	-	KU244357	-	KU244313
Cacosternum_magnaglandiferus	-	-	KF144577	-	-
Conraua_crassipes	-	DQ019498	-	KX208877	-
Cornufer_guentheri	-	DQ019496	DQ283024	DQ019522	DQ283881
Mantidactylus_guttulatus	JN133020	-	-	-	-
Phrynomantis_annectens	-	EF396099	EF395985	EF396139	-
Odontobatrachus_natator	KP005427	KP005354	-	KX208908	-
Phrynodon_sandersoni	-	GU457749	DQ282973	-	DQ283975
Ptychadena_cooperi	-	MF078097	MF078140	-	DQ283792
Quasipaa_delacouri	-	-	EU979991	-	EU979900
Kurixalus_verrucosus	-	KX554766	KX554671	-	-
Theloderma_corticale	-	-	KU244353	-	KU244315
**Species**	**BDNF**	**POMC**	**SLC8A3**	**CXCR4**	**C-myc**
Abavorana_luctuosa	-	-	-	-	KR264252
Amnirana_albolabris	-	-	-	-	-
Amnirana_amnicola	-	-	-	-	-
Amnirana_darlingi	-	-	-	KR264356	-
Amnirana_galamensis	-	-	-	-	-
Amnirana_lepus	-	-	-	KR264310	-
Amnirana_nicobariensis	-	-	-	-	KR264245
Amolops_afghanus	-	-	-	-	-
Amolops_akhaorum	-	-	-	-	-
Amolops_albispinus	-	-	-	-	-
Amolops_aniqiaoensis	-	-	-	-	-
Amolops_aniqiaoensis	-	-	-	-	-
Amolops_archotaphus	-	-	-	-	-
Amolops_beibengensis	-	-	-	-	-
Amolops_bellulus	-	-	-	-	-
Amolops_chayuensis	-	-	-	-	-
Amolops_chunganensis	-	-	-	-	-
Amolops_compotrix	-	-	-	-	-

Amolops_cremnobatus	-	-	-	-	-
Amolops_cucae	-	-	-	-	-
Amolops_daiyunensis	-	-	-	-	-
Amolops_daorum	-	-	-	-	-
Amolops_granulosus	-	-	-	-	-
Amolops_gyirongensis	-	-	-	-	-
Amolops_gyirongensis	-	-	-	-	-
Amolops_hainanensis	-	-	-	-	-
Amolops_hongkongensis	-	-	-	-	-
Amolops_indoburmanensis	-	-	-	-	-
Amolops_deng	-	-	-	-	-
Amolops_deng	-	-	-	-	-
Amolops_iriodes	-	-	-	-	-
Amolops_jinjiangensis	-	-	-	-	-
Amolops_larutensis	-	-	-	-	-
Amolops_liangshanensis	-	-	-	-	-
Amolops_lifanensis	-	-	-	-	-
Amolops_loloensis	-	-	-	-	-
Amolops_mantzorum	-	-	-	-	-
Amolops_marmoratus	-	-	-	-	-
Amolops_medogensis	-	-	-	-	-
Amolops_medogensis	-	-	-	-	-
Amolops_mengyangensis	-	-	-	-	-
Amolops_nyingchiensis	-	-	-	-	-
Amolops_nyingchiensis	-	-	-	-	-
Amolops_panhai	-	-	-	-	-
Amolops_ricketti	-	-	-	-	-
Amolops_sp	-	-	-	-	-
Amolops_spinapectoralis	-	-	-	-	-
Amolops_torrentis	-	-	-	-	-
Amolops_tuberodepressus	-	-	-	-	-
Amolops_viridimaculatus	-	-	-	-	-
Amolops_vitreus	-	-	-	-	-
Amolops_wangyufani	-	-	-	-	-
Amolops_wangyufani	-	-	-	-	-
Amolops_wuyiensis	-	-	-	-	-
Amolops_yarlungzangbo	-	-	-	-	-
Amolops_yarlungzangbo	-	-	-	-	-
Babina_chapaensis	-	-	-	KR264317	KR264238
Babina_okinavana	-	-	-	-	-
Babina_pleuraden	-	-	-	KR264303	KR264228
Chalcorana_chalconota	-	-	-	-	-
Chalcorana_eschatia	-	-	-	KR264326	KR264248
Chalcorana_megalonesa	-	-	-	-	-

Chalcorana_mocquardi	-	-	-	KR264341	KR264261
Chalcorana_parvaccola	-	-	-	KR264325	KR264247
Chalcorana_rufipes	-	-	-	KR264324	KR264246
Clinotarsus_alticola	-	-	-	-	-
Clinotarsus_curtipes	-	-	-	-	-
Glandirana_emeljanovi	-	-	-	-	-
Glandirana_rugosa	-	-	-	-	-
Glandirana_tientaiensis	-	-	-	-	-
Huia_cavitympanum	-	-	-	-	-
Huia_cavitympanum	-	-	-	-	-
Huia_masonii	-	-	-	-	-
Huia_melasma	-	-	-	-	-
Humerana_humeralis	-	-	-	-	-
Humerana_miopus	-	-	-	-	-
Hydrophylax_bahuvistara	-	-	-	-	-
Hydrophylax_gracilis	-	-	-	-	-
Hydrophylax_leptoglossa	-	-	-	-	-
Hydrophylax_malabaricus	-	-	-	-	-
Hylarana_attigua	-	-	-	-	-
Hylarana_erythraea	-	-	-	-	-
Hylarana_latouchii	-	-	-	-	-
Hylarana_macrodactyla	-	-	-	KR264355	KR264276
Hylarana_taipehensis	-	-	-	KR264285	KR264215
Hylarana_tytleri	-	-	-	-	KR264275
Indosylvirana_caesari	-	-	-	-	-
Indosylvirana_aurantiaca	-	-	-	-	-
Indosylvirana_doni	-	-	-	-	-
Indosylvirana_flavescens	-	-	-	-	-
Indosylvirana_indica	-	-	-	-	-
Indosylvirana_intermedia	-	-	-	-	-
Indosylvirana_magna	-	-	-	-	-
Indosylvirana_milleti	-	-	-	-	-
Indosylvirana_montana	-	-	-	-	-
Indosylvirana_serendipi	-	-	-	-	-
Indosylvirana_sreeni	-	-	-	-	-
Indosylvirana_temporalis	-	-	-	-	-
Indosylvirana_urbis	-	-	-	-	-
Meristogenys_amoropalamus	-	AB526659	AB526687	-	-
Meristogenys_dyscritus	-	AB360155	AB526684	-	-
Meristogenys_maryatiae	-	AB526651	AB526679	-	-
Meristogenys_poecilus	-	B526650	AB526678	-	-
Meristogenys_stenocephalus	-	AB526652	AB526680	-	-
Meristogenys_stigmachilus	-	AB526654	AB526682	-	-
Odorrana_absita	-	-	-	-	-

Odorrana_amamiensis	-	-	-	-	-
Odorrana_andersonii	-	-	-	-	-
Odorrana_anlungensis	-	-	-	-	-
Odorrana_aureola	-	-	-	-	-
Odorrana_bacboensis	-	-	-	-	-
Odorrana_banaorum	-	-	-	-	-
Odorrana_chapaensis	-	-	-	-	-
Odorrana_chloronota	-	-	-	-	-
Odorrana_exiliversabilis	-	-	-	-	-
Odorrana_fengkaiensis	-	-	-	-	-
Odorrana_geminata	-	-	-	-	-
Odorrana_graminea	-	-	-	-	-
Odorrana_hainanensis	-	-	-	-	-
Odorrana_hejiangensis	-	-	-	-	-
Odorrana_hosii	-	-	-	KR264331	KR264251
Odorrana_huanggangensis	-	-	-	-	-
Odorrana_ishikawae	-	-	-	-	-
Odorrana_jingdongensis	-	-	-	-	-
Odorrana_jingdongensis	-	-	-	-	-
Odorrana_junlianensis	-	-	-	-	-
Odorrana_kuangwuensis	-	-	-	-	-
Odorrana_leporipes	-	-	-	-	-
Odorrana_lipuensis	-	-	-	-	-
Odorrana_livida	-	-	-	-	-
Odorrana_lungshengensis	-	-	-	-	-
Odorrana_margaretae	-	-	-	-	-
Odorrana_morafkai	-	-	-	-	-
Odorrana_mutschmanni	-	-	-	-	-
Odorrana_nanjiangensis	-	-	-	-	-
Odorrana_narina	-	-	-	-	-
Odorrana_nasica	-	-	-	-	-
Odorrana_nasuta	-	-	-	-	-
Odorrana_schmackeri	-	-	-	-	-
Odorrana_supranarina	-	-	-	-	-
Odorrana_swinhoana	-	-	-	-	-
Odorrana_tianmuii	-	-	-	-	-
Odorrana_tiannanensis	-	-	-	-	-
Odorrana_tormota	-	-	-	-	-
Odorrana_utsunomiyaorum	-	-	-	-	-
Odorrana_versabilis	-	-	-	-	-
Odorrana_wuchuanensis	-	-	-	-	-
Odorrana_yizhangensis	-	-	-	-	-
Papurana__daemeli	-	-	-	KR264300	-
Papurana_arfaki	-	-	-	KR264292	KR264220

Papurana_aurata	-	-	-	KR264342	-
Papurana_garritor	-	-	-	KR264286	-
Papurana_jimiensis	-	-	-	KR264297	-
Papurana_kreffti	-	-	-	KR264294	KR264222
Papurana_milneana	-	-	-	KR264288	KR264216
Papurana_papua	-	-	-	KR264298	KR264224
Papurana_supragrisea	-	-	-	KR264302	KR264227
Papurana_volkerjane	-	-	-	-	KR264264
Papurana_waliesa	-	-	-	KR264290	KR264218
Pelophylax_lateralis	KF991301	-	-	-	-
Pelophylax_nigromaculatus	KX269291	-	-	-	-
Pelophylax_ridibundus	-	-	-	-	-
Pelophylax_saharicus	-	-	-	-	-
Pseudorana_weiningensis	KX269292	-	-	-	-
Pulchrana_banjarana	-	KF477787	-	-	-
Pulchrana_baramica	-	-	-	-	-
Pulchrana_glandulosa	-	-	-	-	-
Pulchrana_grandocula	-	-	-	-	-
Pulchrana_laterimaculata	-	-	-	-	-
Pulchrana_mangyanum	-	KF477823	-	-	-
Pulchrana_melanomenta	-	KF477830	-	-	-
Pulchrana_moellendorffi	-	-	-	-	-
Pulchrana_picturata	-	-	-	KR264313	-
Pulchrana_rawa	-	-	-	-	-
Pulchrana_siberu	-	-	-	-	-
Pulchrana_signata	-	-	-	-	-
Pulchrana_similis	-	-	-	KR264321	KR264241
Rana_catesbeianus	AB612040	AY819103	AB777225	AB612041	AY819188
Rana_kukunoris	KX269260	KX269474	KX269702	-	-
Rana_pipiens	-	-	-	-	-
Rana_septentrionalis	KX269240	KX269454	KX269681	-	-
Rana_temporaria	KC800199	KC800339	EF107369	EF017988	-
Rana_tlaloci	KX269251	KX269465	KX269692	-	-
Sanguirana_luzonensis	-	-	-	KR264350	-
Sanguirana_sanguinea	-	-	-	KT881648	-
Staurois_guttatus	-	-	-	-	-
Staurois_latopalmatus	-	-	-	-	-
Staurois_natator	-	-	-	-	-
Sylvirana_cubitalis	-	-	-	KR264320	-
Sylvirana_faber	-	-	-	-	-
Sylvirana_guentheri	-	-	-	KR264284	KR264214
Sylvirana_maosonensis	-	-	-	-	-
Sylvirana_mortenseni	-	MG606304	-	KR264319	KR264240
Sylvirana_nigrovittata	-	-	-	-	-

Sylvirana_spinulosa	-	-	-	KR264336	KR264257
**Outgroup**					
Buergeria_oxycephalus	KU244396	-	-	-	-
Cacosternum_magnaglandiferus	-	-	-	-	-
Conraua_crassipes	-	-	-	KX200444	-
Cornufer_guentheri	-	KP298156	-	-	-
Mantidactylus_guttulatus	-	-	-	-	-
Phrynomantis_annectens	EF396025	-	-	-	-
Odontobatrachus_natator	KF693490	-	-	-	-
Phrynodon_sandersoni	-	-	-	-	-
Ptychadena_cooperi	-	-	-	MF078275	-
Quasipaa_delacouri	-	-	-	-	-
Kurixalus_verrucosus	KX554863	-	-	-	-
Theloderma_corticale	KU244394	-	-	-	-

**Species**	**RH1**	**NCX1**
Abavorana_luctuosa	-	-
Amnirana_albolabris	-	-
Amnirana_amnicola	-	-
Amnirana_darlingi	-	-
Amnirana_galamensis	-	-
Amnirana_lepus	-	-
Amnirana_nicobariensis	-	-
Amolops_afghanus	-	-
Amolops_akhaorum	-	-
Amolops_albispinus	-	-
Amolops_aniqiaoensis	-	-
Amolops_aniqiaoensis	-	-
Amolops_archotaphus	-	-
Amolops_beibengensis	-	-
Amolops_bellulus	-	-
Amolops_chayuensis	-	-
Amolops_chunganensis	-	-
Amolops_compotrix	-	-
Amolops_cremnobatus	-	-
Amolops_cucae	-	-
Amolops_daiyunensis	-	-
Amolops_daorum	-	-
Amolops_granulosus	-	-
Amolops_gyirongensis	-	-
Amolops_gyirongensis	-	-
Amolops_hainanensis	-	-
Amolops_hongkongensis	-	-
Amolops_indoburmanensis	-	-
Amolops_deng	-	-

Amolops_deng	-	-
Amolops_iriodes	-	-
Amolops_jinjiangensis	-	-
Amolops_larutensis	-	-
Amolops_liangshanensis	-	-
Amolops_lifanensis	-	-
Amolops_loloensis	-	-
Amolops_mantzorum	-	-
Amolops_marmoratus	-	-
Amolops_medogensis	-	-
Amolops_medogensis	-	-
Amolops_mengyangensis	-	-
Amolops_nyingchiensis	-	-
Amolops_nyingchiensis	-	-
Amolops_panhai	-	-
Amolops_ricketti	-	-
Amolops_sp	-	-
Amolops_spinapectoralis	-	-
Amolops_torrentis	-	-
Amolops_tuberodepressus	-	-
Amolops_viridimaculatus	-	-
Amolops_vitreus	-	-
Amolops_wangyufani	-	-
Amolops_wangyufani	-	-
Amolops_wuyiensis	-	-
Amolops_yarlungzangbo	-	-
Amolops_yarlungzangbo	-	-
Babina_chapaensis	-	-
Babina_okinavana	-	-
Babina_pleuraden	-	-
Chalcorana_chalconota	-	-
Chalcorana_eschatia	-	-
Chalcorana_megalonesa	-	-
Chalcorana_mocquardi	-	-
Chalcorana_parvaccola	-	-
Chalcorana_rufipes	-	-
Clinotarsus_alticola	-	-
Clinotarsus_curtipes	-	-
Glandirana_emeljanovi	-	-
Glandirana_rugosa	-	-
Glandirana_tientaiensis	-	-
Huia_cavitympanum	-	-
Huia_cavitympanum	-	-
Huia_masonii	-	-

Huia_melasma	-	-
Humerana_humeralis	-	-
Humerana_miopus	-	-
Hydrophylax_bahuvistara	-	-
Hydrophylax_gracilis	-	-
Hydrophylax_leptoglossa	-	-
Hydrophylax_malabaricus	-	-
Hylarana_attigua	-	-
Hylarana_erythraea	-	-
Hylarana_latouchii	-	-
Hylarana_macrodactyla	-	-
Hylarana_taipehensis	-	-
Hylarana_tytleri	-	-
Indosylvirana_caesari	-	-
Indosylvirana_aurantiaca	-	-
Indosylvirana_doni	-	-
Indosylvirana_flavescens	-	-
Indosylvirana_indica	-	-
Indosylvirana_intermedia	-	-
Indosylvirana_magna	-	-
Indosylvirana_milleti	-	-
Indosylvirana_montana	-	-
Indosylvirana_serendipi	-	-
Indosylvirana_sreeni	-	-
Indosylvirana_temporalis	-	-
Indosylvirana_urbis	-	-
Meristogenys_amoropalamus	AB526701	AB526645
Meristogenys_dyscritus	AB526698	AB526642
Meristogenys_maryatiae	AB526693	AB526637
Meristogenys_poecilus	AB526692	AB526636
Meristogenys_stenocephalus	AB526694	AB526638
Meristogenys_stigmachilus	AB526696	AB526640
Odorrana_absita	-	-
Odorrana_amamiensis	-	-
Odorrana_andersonii	-	-
Odorrana_anlungensis	-	-
Odorrana_aureola	-	-
Odorrana_bacboensis	-	-
Odorrana_banaorum	-	-
Odorrana_chapaensis	-	-
Odorrana_chloronota	-	-
Odorrana_exiliversabilis	-	-
Odorrana_fengkaiensis	-	-
Odorrana_geminata	-	-

Odorrana_graminea	-	-
Odorrana_hainanensis	-	-
Odorrana_hejiangensis	-	-
Odorrana_hosii	-	-
Odorrana_huanggangensis	-	-
Odorrana_ishikawae	-	-
Odorrana_jingdongensis	-	-
Odorrana_jingdongensis	-	-
Odorrana_junlianensis	-	-
Odorrana_kuangwuensis	-	-
Odorrana_leporipes	-	-
Odorrana_lipuensis	-	-
Odorrana_livida	-	-
Odorrana_lungshengensis	-	-
Odorrana_margaretae	-	-
Odorrana_morafkai	-	-
Odorrana_mutschmanni	-	-
Odorrana_nanjiangensis	-	-
Odorrana_narina	-	-
Odorrana_nasica	-	-
Odorrana_nasuta	-	-
Odorrana_schmackeri	-	-
Odorrana_supranarina	-	-
Odorrana_swinhoana	-	-
Odorrana_tianmuii	-	-
Odorrana_tiannanensis	-	-
Odorrana_tormota	-	-
Odorrana_utsunomiyaorum	-	-
Odorrana_versabilis	-	-
Odorrana_wuchuanensis	-	-
Odorrana_yizhangensis	-	-
Papurana__daemeli	-	-
Papurana_arfaki	-	-
Papurana_aurata	-	-
Papurana_garritor	-	-
Papurana_jimiensis	-	-
Papurana_kreffti	-	-
Papurana_milneana	-	-
Papurana_papua	-	-
Papurana_supragrisea	-	-
Papurana_volkerjane	-	-
Papurana_waliesa	-	-
Pelophylax_lateralis	-	-
Pelophylax_nigromaculatus	-	-

Pelophylax_ridibundus	-	-
Pelophylax_saharicus	-	-
Pseudorana_weiningensis	-	-
Pulchrana_banjarana	-	-
Pulchrana_baramica	-	-
Pulchrana_glandulosa	-	-
Pulchrana_grandocula	-	-
Pulchrana_laterimaculata	-	-
Pulchrana_mangyanum	-	-
Pulchrana_melanomenta	-	-
Pulchrana_moellendorffi	-	-
Pulchrana_picturata	-	-
Pulchrana_rawa	-	-
Pulchrana_siberu	-	-
Pulchrana_signata	-	-
Pulchrana_similis	-	-
Rana_catesbeianus	-	AB612042
Rana_kukunoris	-	-
Rana_pipiens	-	-
Rana_septentrionalis	-	-
Rana_temporaria	-	EF018012
Rana_tlaloci	-	-
Sanguirana_luzonensis	-	-
Sanguirana_sanguinea	-	-
Staurois_guttatus	-	-
Staurois_latopalmatus	-	-
Staurois_natator	-	-
Sylvirana_cubitalis	-	-
Sylvirana_faber	-	-
Sylvirana_guentheri	-	-
Sylvirana_maosonensis	-	-
Sylvirana_mortenseni	-	MG605971
Sylvirana_nigrovittata	-	-
Sylvirana_spinulosa	-	-
**Outgroup**		
Buergeria_oxycephalus	-	-
Cacosternum_magnaglandiferus	-	-
Conraua_crassipes	-	EF018016
Cornufer_guentheri	-	AY948830
Mantidactylus_guttulatus	-	-
Phrynomantis_annectens	-	-
Odontobatrachus_natator	-	-
Phrynodon_sandersoni	-	-
Ptychadena_cooperi	-	MF078178

Quasipaa_delacouri	-	-
Kurixalus_verrucosus	-	-
Theloderma_corticale	-	-

Rhacophoridae

Species	ND1	COI	12s	16s	Cytb
Ghatixalus_asterops	-	-	EU178098	EU178091	-
Ghatixalus_variabilis	-	-	KU170006	KU169981	-
Ghatixalus_magnus	-	-	KT359618	KT359624	-
Mercurana_myristicapalustris	-	-	KC594293	KC594294	-
Nasutixalus_jerdonii	-	-	KU230457	KU170001	-
Nasutixalus_medogensis	-	-	GQ285679	GQ285679	-
Beddomixalus_bijui	-	-	KC594289	KC594290	-
Taruga_eques	-	-	AY141800	GQ204689	GQ204505
Taruga_fastigo	-	-	AY141802	GQ204690	GQ204506
Taruga_longinasus	-	-	GQ204745	GQ204691	GQ204507
Nyctixalus_margaritifer	-	-	EU178094	EU178087	-
Nyctixalus_pictus	-	KU244454	GQ204783	GQ204732	GQ204549
Nyctixalus_spinosus	-	-	DQ283114	DQ283114	-
Feihyla_kajau	-	-	KC961241	KC961088	-
Feihyla_palpebralis	-	KP996795	EU215546	EU215546	-
Feihyla_vittatus	-	-	GQ285684	**MW019898**	EU924596
Feihyla_hansenae	-	KR087645	AY880579	AY880493	-
Buergeria_buergeri	-	-	AB127977	AB127977	AB127977
Buergeria_japonica	-	-	AF458123	AF458123	MF536335
Buergeria_oxycephala	-	-	EU215524	EU215524	GQ204547
Buergeria_robusta	-	-	AF458125	AF458125	-
Liuixalus_calcarius	-	-	-	AB871420	-
Liuixalus_feii	-	-	-	KT198731	-
Liuixalus_hainanus	-	-	GQ285671	GQ285671	-
Liuixalus_ocellatus	-	KP996840	-	AB871416	-
Liuixalus_romeri	-	KP996831	-	KT192636	-
Liuixalus_shiwandashan	-	-	EU215528	EU215528	-
Gracixalus_gracilipes	-	KR087671	GQ285668	GQ285668	EU924593
Gracixalus_jinxiuensis	-	-	EU215525	EU215525	EU924599
Gracixalus_jinggangensis	-	-	-	KY624587	-
Gracixalus_nonggangensis	-	-	-	JX841320	-
Gracixalus_waza	-	-	-	JX896682	-
Gracixalus_seesom	-	-	-	LC011934	-
Gracixalus_sapaensis	-	-	GQ285670	GQ285670	-
Gracixalus_quangi	-	-	-	JN862538	-
Gracixalus_supercornutus	-	-	-	JN862543	-
Gracixalus_lumarius	-	-	-	KF918412	-
Gracixalus_quyeti	-	-	-	EU871429	-
Gracixalus_medogensis	-	-	-	**MW023606**	-
Kurixalus_appendiculatus	-	-	KC961247	KC961090	KF933134
Kurixalus_baliogaster	-	-	AB933301	AB933301	-

Kurixalus_banaensis	-	-	GQ285667	GQ285667	-
Kurixalus_berylliniris	-	DQ468677	-	DQ468669	-
Kurixalus_bisacculus	-	-	EU215548	EU215548	-
Kurixalus_eiffingeri	-	DQ468680	-	DQ468672	-
Kurixalus_idiootocus	-	KT259131	EU215547	EU215547	GQ204503
Kurixalus_motokawai	-	-	-	LC002890	-
Kurixalus_naso	-	-	**MW111527**	**MW111527**	-
Kurixalus_naso	-	-	**MW111528**	**MW111528**	-
Kurixalus_odontotarsus	-	KR087747	EU215549	EU215549	-
Kurixalus_verrucosus	-	-	GU227274	GU227329	-
Kurixalus_viridescens	-	-	-	AB933285	-
Kurixalus_wangi	-	DQ468679	-	DQ468671	-
Chiromantis_doriae	-	KR087627	EF564444	EF564516	EU924595
Chiromantis_nongkhorensis	-	KR087635	GQ204774	GQ204723	GQ204540
Chiromantis_petersii	-	-	GQ204784	GQ204733	GQ204550
Chiromantis_rufescens	-	-	GQ204775	GQ204724	GQ204541
Chiromantis_xerampelina	-	-	GQ204785	AY880495	GQ204551
Polypedates_braueri	-	KR087858	MN511823	MN511823	AB451722
Polypedates_colletti	-	-	AB728189	AB728189	GQ204514
Polypedates_cruciger	-	-	AY141799	GQ204687	GQ204504
Polypedates_discantus	-	-	AB728180	AB728180	-
Polypedates_impresus	-	-	AB728174	AB728174	-
Polypedates_leucomystax	-	KR087872	AB728137	AB728137	GQ204509
Polypedates_macrotis	-	-	AB728187	AB728187	-
Polypedates_maculatus	-	-	AB728188	AB728188	-
Polypedates_megacephalus	-	KR087886	MN511821	MN511821	-
Polypedates_mutus	-	-	MN511837	MN511837	-
Polypedates_otilophus	-	-	AB728190	AB728190	GQ204513
Polypedates_pseudocruciger	-	-	KU170009	EU450009	-
Polypedates_teraiensis	-	-	-	AB530516	-
Theloderma_albopunctatum	-	KU244441	GQ285677	GQ285677	-
Theloderma_annae	-	-	-	LC168170	-
Theloderma_asperum	-	-	GQ204776	GQ204725	GQ204542
Theloderma_baibungensis	-	-	KU981089	KU981089	-
Theloderma_baibungensis	-	-	**MW111523**	**MW111523**	-
Theloderma_bicolor	-	-	KF991263	JX046475	KF991324
Theloderma_corticale	-	KU244452	-	KU244364	-
Theloderma_gordoni	-	KU244451	-	KU244363	-
Theloderma_horridum	-	-	LC012861	LC012861	-
Theloderma_lacustrinum	-	-	-	KX095245	-
Theloderma_laeve	-	-	KT461897	KT461898	-
Theloderma_lateriticum	-	-	LC012850	LC012850	-
Theloderma_leporosum	-	-	AB847128	AB847128	-
Theloderma_licin	-	KU244447	-	KU244368	-

Theloderma_moloch	-	-	**MW111524**	**MW111524**	-
Theloderma_nebulosum	-	-	-	JN688168	-
Theloderma_palliatum	-	-	KT461893	KT461901	-
Theloderma_phrynoderma	-	KU244448	KJ128280	KJ128282	-
Theloderma_pyaukkya	-	KU244443	-	KU244361	-
Theloderma_rhododiscus	-	-	DQ283392	DQ283393	-
Theloderma_ryabovi	-	-	LC012860	LC012860	-
Theloderma_stellatum	-	-	-	KT461917	-
Theloderma_truongsonense	-	-	-	KT461925	-
Theloderma_vietnamense	-	-	-	KU561887	-
Theloderma_petilum	-	-	KJ802925	KJ802925	-
Raorchestes_agasthyaensis	JX092859	-	-	JX092723	JX092785
Raorchestes_akroparallagi	-	-	JX092726	JX092650	JX092786
Raorchestes_anili	JX092863	KM052268	JX092729	JX092700	JX092790
Raorchestes_archeos	JX092862	-	-	JX092675	JX092789
Raorchestes_aureus	JX092882	-	JX092745	JX092672	JX092811
Raorchestes_beddomii	EU450034	-	JX092731	JX092653	JX092793
Raorchestes_blandus	JX092854	-	JX092725	JX092660	JX092781
Raorchestes_bobingeri	EU450049	-	JX092733	JX092680	JX092795
Raorchestes_bombayensis	EU450054	-	-	JX092657	JX092796
Raorchestes_chalazodes	JX092869	-	JX092734	JX092676	-
Raorchestes_charius	JX092870	-	JX092736	JX092691	JX092799
Raorchestes_chlorosomma	EU450052	-	-	EU450017	-
Raorchestes_chotta	EU450056	-	JX092737	JX092656	JX092800
Raorchestes_chromasynchysi	JX092872	-	JX092738	JX092667	JX092802
Raorchestes_coonoorensis	EU450035	-	JX092740	JX092716	JX092805
Raorchestes_crustai	JX092877	-	JX092742	JX092677	JX092806
Raorchestes_dubois	EU450032	-	-	JX092668	JX092808
Raorchestes_echinatus	-	-	-	JX092696	JX092839
Raorchestes_flaviocularis	JX092880	-	-	JX092682	JX092809
Raorchestes_flaviventris	JX092884	-	JX092746	JX092694	JX092813
Raorchestes_ghatei	-	-	-	KF366386	-
Raorchestes_glandulosus	EU450042	-	JX092744	JX092665	JX092810
Raorchestes_graminirupes	EU450050	-	JX092772	JX092649	JX092812
Raorchestes_griet	EU450033	-	-	JX092654	JX092814
Raorchestes_gryllus	-	-	GQ285674	GQ285674	-
Raorchestes_hassanensis	-	-	JX092748	JX092673	JX092815
Raorchestes_honnametti	KT151653	-	-	KT151651	-
Raorchestes_indigo	JX092886	-	JX092749	JX092678	-
Raorchestes_jayarami	EU450057	-	JX092750	JX092686	JX092816
Raorchestes_johnceei	JX092888	-	JX092751	JX092679	JX092817
Raorchestes_kadalarensis	JX092889	-	-	JX092701	JX092818
Raorchestes_kaikatti	EU450055	-	JX092752	JX092718	JX092822
Raorchestes_kakachi	JX092912	-	JX092765	JX092695	JX092843

Raorchestes_lechiya	-	-	-	KT359623	-
Raorchestes_leucolatus	JX092908	-	-	JX092669	JX092838
Raorchestes_longchuanensis	**MW111462**	-	GQ285675	**MW019899**	-
Raorchestes_luteolus	EU450041	-	JX092756	JX092659	JX092823
Raorchestes_manohari	JX092895	-	-	JX092674	JX092824
Raorchestes_marki	EU450060	-	JX092757	JX092719	JX092825
Raorchestes_menglaensis	**MW111463**	-	GQ285676	**MW019900**	-
Raorchestes_munnarensis	JX092899	-	-	JX092655	JX092828
Raorchestes_nerostagona	JX092900	-	JX092760	JX092661	JX092830
Raorchestes_ochlandrae	JX092902	-	JX092743	JX092666	JX092831
Raorchestes_parvulus	**MW149296**	KR087901	-	**MW019901**	-
Raorchestes_ponmudi	EU450047	-	JX092762	JX092651	JX092832
Raorchestes_primarrumpfi	JX092904	-	-	JX092717	JX092833
Raorchestes_resplendens	JX092905	-	-	JX092683	JX092835
Raorchestes_signatus	EU450036	-	KU170011	KU169986	JX092836
Raorchestes_silentvalley	-	-	-	KT359629	-
Raorchestes_sushili	EU450059	-	JX092766	JX092684	JX092844
Raorchestes_theuerkaufi	JX092914	-	JX092767	JX092693	JX092845
Raorchestes_tinniens	EU450037	-	-	JX092715	JX092846
Raorchestes_travancoricus	EU450061	-	JX092776	JX092721	JX092847
Raorchestes_tuberohumerus	EU450040	-	-	EU450004	JX092848
Raorchestes_uthamani	JX092918	-	-	JX092722	JX092849
Pseudophilautus_kani	JX092892	-	JX092754	JX092724	JX092820
Pseudophilautus_alto	-	-	GQ204738	GQ204677	GQ204494
Pseudophilautus_amboli	-	KM052264	JX092727	JX092658	JX092787
Pseudophilautus_asankai	-	-	FJ788141	FJ788160	-
Pseudophilautus_bambaradeniyai	-	-	-	KP272047	-
Pseudophilautus_cavirostris	-	-	GQ204737	GQ204676	GQ204493
Pseudophilautus_decoris	-	-	FJ788144	FJ788163	-
Pseudophilautus_dilmah	-	-	-	KP272046	-
Pseudophilautus_folicola	-	-	-	GQ204680	GQ204497
Pseudophilautus_hankeni	-	-	GU593346	GU593348	-
Pseudophilautus_hoffmanni	-	-	FJ788142	FJ788161	-
Pseudophilautus_leucorhinus	-	-	AY763796	AY753559	-
Pseudophilautus_limbus	-	-	AY141779	GQ204668	GQ204485
Pseudophilautus_lunatus	-	-	FJ788150	GQ204675	GQ204492
Pseudophilautus_microtympanum	-	-	GQ204739	GQ204678	GQ204495
Pseudophilautus_mittermeieri	-	-	GQ204741	GQ204681	GQ204498
Pseudophilautus_mooreorum	-	-	FJ788134	FJ788153	-
Pseudophilautus_ocularis	-	-	FJ788145	FJ788164	-
Pseudophilautus_papillosus	-	-	FJ788151	FJ788170	-
Pseudophilautus_pleurotaenia	-	-	FJ788146	FJ788165	-
Pseudophilautus_poppiae	-	-	FJ788136	GQ204670	GQ204487
Pseudophilautus_popularis	-	-	FJ788149	FJ788168	-

Pseudophilautus_sarasinorum	-	-	AY141761	GQ204667	GQ204484
Pseudophilautus_schmarda	-	-	AY141780	GQ204669	GQ204486
Pseudophilautus_schneideri	-	-	GU593345	GU593347	-
Pseudophilautus_simba	-	-	GQ204740	GQ204679	GQ204496
Pseudophilautus_steineri	-	-	FJ788138	FJ788157	-
Pseudophilautus_stellatus	-	-	JN862535	JN862536	-
Pseudophilautus_stuarti	-	-	GQ204735	GQ204672	GQ204489
Pseudophilautus_tanu	-	-	FJ788152	FJ788171	-
Pseudophilautus_wynaadensis	JX092920	KM052269	KU170008	KU169983	JX092850
Pseudophilautus_zorro	-	-	FJ788147	GQ204671	GQ204488
Philautus_abditus	-	-	GQ285673	GQ285673	GQ204526
Philautus_acutirostris	-	-	AF458137	AF458137	-
Philautus_acutus	-	-	JN705337	JN705366	-
Philautus_amoenus	-	-	KC961236	KC961076	-
Philautus_aurantium	-	-	GQ204756	GQ204705	GQ204522
Philautus_aurifasciatus	-	-	AY141804	AY141850	-
Philautus_bunitus	-	-	JN705339	JN705368	-
Philautus_cornutus	-	-	-	KY435421	-
Philautus_davidlabangi	-	-	JN705356	JN705386	-
Philautus_disgregus	-	-	KC961250	KC961077	GQ204521
Philautus_everetti	-	-	-	JN705377	-
Philautus_hosii	-	-	JN705353	JN705384	-
Philautus_ingeri	-	-	JN705354	JN705385	GQ204523
Philautus_juliandringi	-	-	JN705347	JN705378	-
Philautus_kakipanjang	-	-	-	KF240718	-
Philautus_kerangae	-	-	KC961238	KC961079	-
Philautus_macroscelis	-	-	JN705346	JN705375	-
Philautus_mjobergi	-	-	JN705349	JN705380	-
Philautus_nephophilus	-	-	-	KT445970	-
Philautus_petersi	-	-	JN705350	JN705381	-
Philautus_refugii	-	-	JN705351	JN705382	-
Philautus_surdus	-	-	AF458138	AF458138	-
Philautus_tectus	-	-	JN705340	JN705369	-
Philautus_umbra	-	-	JN705348	JN705379	-
Philautus_vermiculatus	-	-	-	KY435439	-
Philautus_worcesteri	-	-	GQ204758	GQ204707	GQ204524
Rhacophorus_achantharrhena	MF066239	MF066239	MF066239	MF066239	MF066239
Rhacophorus_angulirostris	-	-	JN705322	JN377348	-
Rhacophorus_annamensis	-	-	GQ204768	GQ204717	GQ204534
Rhacophorus_arboreus	-	-	-	AY880523	-
Rhacophorus_baluensis	-	-	KC961239	KC961089	-
Rhacophorus_belalongensis	-	-	JN705324	JN377352	-
Rhacophorus_bengkuluensis	-	-	-	KM212948	-
Rhacophorus_bimaculatus	-	-	KF933261	-	KF933141

Rhacophorus_bipunctatus	-	-	**MW111517**	**MW111517**	-
Rhacophorus_bipunctatus	-	-	**MW111518**	**MW111518**	-
Rhacophorus_borneensis	-	-	AB781693	AB781693	-
Rhacophorus_burmanus	-	-	**MW111519**	**MW111519**	-
Rhacophorus_burmanus	-	-	**MW111520**	**MW111520**	-
Rhacophorus_calcaneus	-	-	-	LC010570	-
Rhacophorus_catamitus	-	-	-	JF748387	-
Rhacophorus_chenfui	-	-	GQ204763	GQ204712	GQ204529
Rhacophorus_cyanopunctatus	-	-	KC961249	KC961084	-
Rhacophorus_dennysi	KT191129	KT191129	EF564467	EF564539	EU924604
Rhacophorus_dorsoviridis	-	-	EF564490	EF564562	EU924610
Rhacophorus_duboisi	-	-	EF564495	EF564567	EU924613
Rhacophorus_dugritei	-	-	EU215541	EU215541	-
Rhacophorus_dulitensis	-	-	JN705326	JX219434	-
Rhacophorus_exechopygus	-	-	-	LC010586	-
Rhacophorus_fasciatus	-	-	JN705331	JN377357	-
Rhacophorus_feae	-	-	EF564474	EF564546	EU924606
Rhacophorus_gadingensis	-	-	KC961242	KC961087	-
Rhacophorus_gauni	-	-	JN705325	JN377351	-
Rhacophorus_harrissoni	-	-	JN705332	JN377359	-
Rhacophorus_helenae	-	-	-	KX139177	-
Rhacophorus_hongchibaensis	-	-	JN688883	JN688883	-
Rhacophorus_hui	-	-	EU924627	EU924622	EU924607
Rhacophorus_hungfuensis	-	-	EU215538	EU215538	-
Rhacophorus_indonesiensis	-	-	-	AB983368	-
Rhacophorus_kio	-	KR087904	EU215532	EU215532	-
Rhacophorus_lateralis	-	-	AY880612	AY880525	-
Rhacophorus_malabaricus	-	-	KU170014	AY948724	-
Rhacophorus_smaragdinus	-	-	JX219411	JX219411	-
Rhacophorus_minimus	-	-	EF564489	EF564561	EU924609
Rhacophorus_moltrechti	-	DQ468684	EU215543	EU215543	-
Rhacophorus_monticola	-	-	AY326060	AY326060	-
Rhacophorus_nigropalmatus	-	-	GQ204761	GQ204710	GQ204527
Rhacophorus_nigropunctatus	-	-	EU215533	EU215533	EU924611
Rhacophorus_norhayatii	-	-	AB728191	AB728191	-
Rhacophorus_omeimontis	-	-	EF564492	EF564564	EU924612
Rhacophorus_orlovi	-	-	DQ283049	DQ283049	-
Rhacophorus_pardalis	-	-	GQ204762	GQ204711	GQ204528
Rhacophorus_penanorum	-	-	JN705323	JN377349	-
Rhacophorus_pinglongensis	-	-	KU170683	KU170683	-
Rhacophorus_poecilonotus	MF066240	MF066240	MF066240	MF066240	MF066240
Rhacophorus_pseudomalabaricus	-	-	-	KC593855	-
Rhacophorus_puerensis	-	-	EF564469	EF564541	EU924605
Rhacophorus_reinwardti	-	-	GQ204771	GQ204720	GQ204537

Rhacophorus_rhodopus	-	KR087912	EF564500	EF564572	EU924616
Rhacophorus_bipunctatus	-	-	EF564507	EF564579	-
Rhacophorus_rhodopus	-	-	AY843750	AY843750	-
Rhacophorus_robertingeri	-	-	-	LC010615	-
Rhacophorus_rufipes	-	-	JN705334	JN377360	-
Rhacophorus_schlegelii	NC_007178	NC_007178	NC_007178	NC_007178	NC_007178
Rhacophorus_translineatus	-	-	**MW111521**	**MW111521**	-
Rhacophorus_vampyrus	-	-	-	HQ656815	-
Rhacophorus_tuberculatus	-	-	**MW111522**	**MW111522**	-
Rhacophorus_wui	-	-	JN688881	JN688881	-
Rhacophorus_zhoukaiyae	-	-	KU601494	KU601494	-
Rhacophorus_lishuiensis	-	-	KY653717	KY653717	-
**Outgroup**					
Microhyla_annectens	-	AB611944	AB634601	AB611947	-
Pelophylax_lateralis	-	-	KF991259	KF991279	KF991320
Pelophylax_nigromaculatus	-	KT878718	KT878718	KT878718	KT878718
Pelophylax_ridibundus	-	KT879314	AB640897	MH410481	MG575226
Pelophylax_saharicus	-	KP177812	-	KP177671	GU799108
Rana_kukunoris	-	-	KC465786	KC465786	-
Rana_catesbeianus	-	NC_022696	NC_022696	NC_022696	NC_022696
Rana_temporaria	-	KP697915	AB058864	AB058882	MF624355
Rana_pipiens	-	EF525892	Y10945	X86284	-
Rana_tlaloci	-	-	AY779234	AY779234	KX269323
Rana_septentrionalis	-	EF525899	KX269179	KX269179	AY083273
Alcalus_tasanae	-	-	KU243088	-	-
Liurana_xizangensis	-	-	KU243084	-	-
Limnonectes_limborgi	-	-	DQ347061	GU934365	-
Occidozyga_martensi	-	-	KU243090	DQ458255	-
Odontobatrachus_natator	-	-	KP005222	KP005103	KP005427
Conraua_crassipes	-	-	DQ347015	DQ347305	-
Blommersia_wittei	-	-	AY341586	AY880490	-
Laliostoma_labrosum	-	-	DQ283057	DQ283057	-
**Species**	**BDNF**	**POMC**	**RAG1**	**Rhod**	**Tyr**
Ghatixalus_asterops	-	-	-	-	-
Ghatixalus_variabilis	-	-	KU169956	KU169932	-
Ghatixalus_magnus	-	-	KT359634	KT359630	-
Mercurana_myristicapalustris	-	-	KC594295	KC594296	-
Nasutixalus_jerdonii	-	-	KU169976	KU169952	-
Nasutixalus_medogensis	GQ285690	GQ285721	GQ285753	GQ285782	GQ285800
Beddomixalus_bijui	-	-	KC594291	KC594292	-
Taruga_eques	GQ204447	-	GQ204571	GQ204633	-
Taruga_fastigo	GQ204448	-	GQ204572	GQ204634	-
Taruga_longinasus	GQ204449	-	GQ204573	GQ204635	-
Nyctixalus_margaritifer	-	-	-	-	-

Nyctixalus_pictus	GQ204483	-	GQ204613	GQ204666	KU244355
Nyctixalus_spinosus	-	-	-	DQ283827	-
Feihyla_kajau	KC961110	KC961180	-	-	KC961234
Feihyla_palpebralis	-	GQ285741	GQ285773	EU215576	-
Feihyla_vittatus	GQ285711	GQ285742	GQ285774	GQ285793	GQ285811
Feihyla_hansenae	GQ285710	-	-	AY880626	EU215606
Buergeria_buergeri	AB728215	AB728249	AB728271	AB728288	AB728322
Buergeria_japonica	GQ285691	GQ285722	GQ285754	GQ285783	GQ285801
Buergeria_oxycephala	GQ285695	GQ285726	GQ285758	EU215556	EU215585
Buergeria_robusta	-	-	-	-	-
Liuixalus_calcarius	-	-	-	-	-
Liuixalus_feii	-	-	-	-	-
Liuixalus_hainanus	GQ285694	GQ285725	GQ285757	GQ285785	GQ285803
Liuixalus_ocellatus	-	-	-	-	-
Liuixalus_romeri	-	-	-	-	-
Liuixalus_shiwandashan	GQ285693	GQ285724	-	EU215559	EU215589
Gracixalus_gracilipes	GQ285701	GQ285732	GQ285764	GQ285789	GQ285807
Gracixalus_jinxiuensis	GQ285700	GQ285731	GQ285763	EU215557	EU215587
Gracixalus_jinggangensis	-	-	-	-	-
Gracixalus_nonggangensis	-	-	-	-	-
Gracixalus_waza	-	-	-	-	-
Gracixalus_seesom	-	-	-	-	-
Gracixalus_sapaensis	GQ285699	GQ285730	GQ285762	GQ285788	GQ285806
Gracixalus_quangi	-	-	-	-	-
Gracixalus_supercornutus	-	-	-	-	-
Gracixalus_lumarius	-	-	-	-	-
Gracixalus_quyeti	-	-	-	-	-
Gracixalus_medogensis	-	-	-	-	-
Kurixalus_appendiculatus	KC961138	KC961188	-	-	KC961231
Kurixalus_baliogaster	-	-	-	-	-
Kurixalus_banaensis	GQ285689	GQ285720	GQ285752	GQ285781	GQ285799
Kurixalus_berylliniris	-	-	-	-	-
Kurixalus_bisacculus	GQ285686	GQ285717	GQ285749	EU215578	EU215608
Kurixalus_eiffingeri	-	-	-	-	-
Kurixalus_idiootocus	GQ285688	GQ285719	GQ285751	EU215577	EU215607
Kurixalus_motokawai	-	-	-	-	-
Kurixalus_naso	-	-	-	-	-
Kurixalus_naso	-	-	-	-	-
Kurixalus_odontotarsus	GQ285687	GQ285718	GQ285750	EU215579	EU215609
Kurixalus_verrucosus	-	-	JQ060901	-	JQ060917
Kurixalus_viridescens	-	-	-	-	-
Kurixalus_wangi	-	-	-	-	-
Chiromantis_doriae	GQ285716	-	EU924511	EU924539	EU924567
Chiromantis_nongkhorensis	-	-	GQ204604	GQ204659	-

Chiromantis_petersii	-	-	-	-	-
Chiromantis_rufescens	GQ204476	-	GQ204605	GQ204660	DQ347139
Chiromantis_xerampelina	-	-	-	AY880628	-
Polypedates_braueri	AB728193	AB728234	AB728251	AB728273	AB728307
Polypedates_colletti	AB728212	AB728246	AB728268	AB728285	AB728319
Polypedates_cruciger	GQ204446	HM998970	GQ204570	GQ204632	-
Polypedates_discantus	-	-	-	-	-
Polypedates_impresus	AB728208	AB728242	AB728264	AB728281	AB728315
Polypedates_leucomystax	AB728205	AB728240	AB728262	AB728279	AB728313
Polypedates_macrotis	AB728210	AB728244	AB728266	AB728283	AB728317
Polypedates_maculatus	AB728211	AB728245	AB728267	AB728284	AB728318
Polypedates_megacephalus	AB728200	AB728239	AB728258	AB728278	AB728312
Polypedates_mutus	AB728196	AB728237	AB728254	AB728276	AB728310
Polypedates_otilophus	AB728213	AB728247	AB728269	AB728286	AB728320
Polypedates_pseudocruciger	-	-	KU169959	KU169935	-
Polypedates_teraiensis	-	-	-	-	-
Theloderma_albopunctatum	KU244401	GQ285728	GQ285760	GQ285786	GQ285804
Theloderma_annae	-	-	-	-	-
Theloderma_asperum	-	-	GQ204606	GQ204661	-
Theloderma_baibungensis	-	-	-	-	-
Theloderma_baibungensis	-	-	-	-	-
Theloderma_bicolor	KF991305	-	KF991342	AY880659	-
Theloderma_corticale	KU244393	-	-	KU244316	KU244354
Theloderma_gordoni	KU244395	-	-	KU244334	KU244358
Theloderma_horridum	-	-	-	-	-
Theloderma_lacustrinum	-	-	-	-	-
Theloderma_laeve	-	-	-	-	-
Theloderma_lateriticum	-	-	-	-	-
Theloderma_leporosum	-	-	-	-	-
Theloderma_licin	KU244384	-	-	KU244333	KU244346
Theloderma_moloch	-	-	KU169968	KU169944	-
Theloderma_nebulosum	-	-	-	-	-
Theloderma_palliatum	-	-	-	-	-
Theloderma_phrynoderma	KU244402	-	-	KJ128278	KJ128276
Theloderma_pyaukkya	KU244399	-	-	KU244331	KU244339
Theloderma_rhododiscus	-	-	-	DQ284007	DQ282998
Theloderma_ryabovi	-	-	-	-	-
Theloderma_stellatum	-	-	-	-	-
Theloderma_truongsonense	-	-	-	-	-
Theloderma_vietnamense	KU561888	-	-	KU561891	KU561897
Theloderma_petilum	-	-	-	-	-
Raorchestes_agasthyaensis	-	-	-	JX092980	JX092926
Raorchestes_akroparallagi	-	-	-	JX092981	JX092927
Raorchestes_anili	-	-	-	JX092984	-

Raorchestes_archeos	-	-	-	JX092983	-
Raorchestes_aureus	-	-	-	JX092996	JX092945
Raorchestes_beddomii	-	-	-	-	-
Raorchestes_blandus	-	-	-	-	-
Raorchestes_bobingeri	-	-	-	-	JX092930
Raorchestes_bombayensis	-	-	-	JX092986	JX092931
Raorchestes_chalazodes	-	-	-	JX092988	JX092932
Raorchestes_charius	-	-	KU169960	JX092989	JX092933
Raorchestes_chlorosomma	-	-	-	-	-
Raorchestes_chotta	-	-	-	-	JX092934
Raorchestes_chromasynchysi	-	-	-	JX092990	JX092935
Raorchestes_coonoorensis	-	-	-	-	JX092939
Raorchestes_crustai	-	-	-	-	JX092940
Raorchestes_dubois	-	-	-	JX092993	JX092942
Raorchestes_echinatus	-	-	-	-	-
Raorchestes_flaviocularis	-	-	-	JX092994	JX092943
Raorchestes_flaviventris	-	-	-	-	-
Raorchestes_ghatei	-	-	-	-	-
Raorchestes_glandulosus	-	-	KU169962	JX092995	JX092944
Raorchestes_graminirupes	-	-	-	-	JX092946
Raorchestes_griet	-	-	KU169965	JX092997	-
Raorchestes_gryllus	GQ285714	GQ285745	GQ285777	GQ285796	GQ285814
Raorchestes_hassanensis	-	-	-	-	-
Raorchestes_honnametti	-	-	-	-	-
Raorchestes_indigo	-	-	-	JX092999	JX092947
Raorchestes_jayarami	-	-	-	JX093000	JX092948
Raorchestes_johnceei	-	-	-	JX093001	JX092949
Raorchestes_kadalarensis	-	-	-	JX093002	JX092950
Raorchestes_kaikatti	-	-	-	JX093003	JX092951
Raorchestes_kakachi	-	-	-	-	-
Raorchestes_lechiya	-	-	-	-	-
Raorchestes_leucolatus	-	-	-	-	JX092968
Raorchestes_longchuanensis	GQ285713	GQ285744	GQ285776	GQ285795	GQ285813
Raorchestes_luteolus	-	-	-	JX093004	JX092954
Raorchestes_manohari	-	-	-	JX093005	JX092955
Raorchestes_marki	-	-	-	JX093006	JX092956
Raorchestes_menglaensis	GQ285715	GQ285746	GQ285778	GQ285797	GQ285815
Raorchestes_munnarensis	-	-	-	JX093008	JX092959
Raorchestes_nerostagona	-	-	-	JX093009	JX092960
Raorchestes_ochlandrae	-	-	-	JX093010	JX092962
Raorchestes_parvulus	-	-	-	-	-
Raorchestes_ponmudi	-	-	-	JX093011	JX092963
Raorchestes_primarrumpfi	-	-	-	JX093012	JX092964
Raorchestes_resplendens	-	-	KU169963	JX093013	JX092965

Raorchestes_signatus	-	-	KU169961	KU169937	JX092966
Raorchestes_silentvalley	-	-	-	-	-
Raorchestes_sushili	-	-	-	JX093018	JX092972
Raorchestes_theuerkaufi	-	-	-	-	-
Raorchestes_tinniens	-	-	KU169966	KU169942	JX092973
Raorchestes_travancoricus	-	-	-	JX093019	JX092974
Raorchestes_tuberohumerus	-	-	-	-	-
Raorchestes_uthamani	-	-	-	JX093020	-
Pseudophilautus_kani	-	-	-	-	JX092952
Pseudophilautus_alto	GQ204440	-	GQ204562	-	-
Pseudophilautus_amboli	-	-	KU169957	JX092982	JX092928
Pseudophilautus_asankai	-	-	-	-	-
Pseudophilautus_bambaradeniyai	-	-	-	-	-
Pseudophilautus_cavirostris	GQ204439	-	GQ204561	GQ204622	-
Pseudophilautus_decoris	-	-	-	-	-
Pseudophilautus_dilmah	-	-	-	-	-
Pseudophilautus_folicola	-	-	GQ204564	GQ204625	-
Pseudophilautus_hankeni	-	-	-	-	-
Pseudophilautus_hoffmanni	-	-	-	-	-
Pseudophilautus_leucorhinus	-	-	-	-	-
Pseudophilautus_limbus	-	-	GQ204553	-	-
Pseudophilautus_lunatus	GQ204438	-	GQ204560	GQ204621	-
Pseudophilautus_microtympanum	GQ204441	-	GQ204563	GQ204623	-
Pseudophilautus_mittermeieri	GQ204443	-	GQ204565	GQ204626	-
Pseudophilautus_mooreorum	-	-	-	-	-
Pseudophilautus_ocularis	-	-	-	-	-
Pseudophilautus_papillosus	-	-	-	-	-
Pseudophilautus_pleurotaenia	-	-	-	-	-
Pseudophilautus_poppiae	-	-	GQ204555	GQ204616	-
Pseudophilautus_popularis	-	-	-	-	-
Pseudophilautus_sarasinorum	-	-	GQ204552	GQ204614	-
Pseudophilautus_schmarda	GQ204435	-	GQ204554	GQ204615	-
Pseudophilautus_schneideri	-	-	-	-	-
Pseudophilautus_simba	GQ204442	-	-	GQ204624	-
Pseudophilautus_steineri	-	-	-	-	-
Pseudophilautus_stellatus	-	-	-	-	-
Pseudophilautus_stuarti	-	-	GQ204557	GQ204618	-
Pseudophilautus_tanu	-	-	-	-	-
Pseudophilautus_wynaadensis	-	-	KU169958	KU169934	-
Pseudophilautus_zorro	-	-	GQ204556	GQ204617	-
Philautus_abditus	GQ285712	GQ285743	GQ285775	GQ285794	GQ285812
Philautus_acutirostris	-	-	-	-	-
Philautus_acutus	KC961117	KC961174	-	-	KC961191
Philautus_amoenus	KC961140	KC961159	-	-	KC961196

Philautus_aurantium	GQ204460	KC961178	GQ204587	GQ204642	KC961193
Philautus_aurifasciatus	-	-	-	-	-
Philautus_bunitus	KC961120	KC961176	-	-	KC961194
Philautus_cornutus	-	-	-	-	-
Philautus_davidlabangi	KC961130	KC961166	-	-	KC961207
Philautus_disgregus	KC961137	KC961167	GQ204586	GQ204641	-
Philautus_everetti	-	-	-	-	-
Philautus_hosii	KC961113	KC961172	-	-	KC961205
Philautus_ingeri	KC961114	KC961173	GQ204588	AY880629	KC961206
Philautus_juliandringi	KC961131	KC961160	-	-	KC961199
Philautus_kakipanjang	-	-	-	-	-
Philautus_kerangae	KC961121	KC961177	-	-	KC961195
Philautus_macroscelis	KC961112	KC961168	-	-	KC961202
Philautus_mjobergi	KC961132	KC961162	-	-	KC961197
Philautus_nephophilus	-	-	-	-	-
Philautus_petersi	KC961134	KC961163	-	-	KC961204
Philautus_refugii	KC961135	KC961164	-	-	KC961200
Philautus_surdus	-	-	-	-	-
Philautus_tectus	KC961116	KC961171	-	-	KC961209
Philautus_umbra	KC961133	KC961161	-	-	KC961198
Philautus_vermiculatus	-	-	-	-	-
Philautus_worcesteri	GQ204462	-	GQ204589	GQ204643	-
Rhacophorus_achantharrhena	-	-	-	-	-
Rhacophorus_angulirostris	KC961099	KC961142	-	-	KC961221
Rhacophorus_annamensis	GQ204470	-	GQ204598	GQ204653	-
Rhacophorus_arboreus	-	-	-	AY880653	-
Rhacophorus_baluensis	KC961093	KC961153	-	-	-
Rhacophorus_belalongensis	KC961101	KC961144	-	-	-
Rhacophorus_bengkuluensis	-	-	-	-	-
Rhacophorus_bimaculatus	-	-	-	-	-
Rhacophorus_bipunctatus	-	-	-	-	-
Rhacophorus_bipunctatus	-	-	-	-	-
Rhacophorus_borneensis	-	-	-	-	-
Rhacophorus_burmanus	-	-	-	EU215567	EU215597
Rhacophorus_burmanus	-	-	-	-	-
Rhacophorus_calcaneus	-	-	-	-	-
Rhacophorus_catamitus	-	-	-	-	-
Rhacophorus_chenfui	GQ204467	-	GQ204594	GQ204648	EU924575
Rhacophorus_cyanopunctatus	KC961098	KC961152	-	-	KC961230
Rhacophorus_dennysi	-	-	EU924520	EU924548	EU924576
Rhacophorus_dorsoviridis	-	-	EU924526	EU924554	EU924582
Rhacophorus_duboisi	-	-	EU924529	EU924557	EU924585
Rhacophorus_dugritei	GQ285705	GQ285736	GQ285768	EU215571	EU215601
Rhacophorus_dulitensis	KC961122	KC961158	-	-	KC961235

Rhacophorus_exechopygus	-	-	-	-	-
Rhacophorus_fasciatus	KC961104	KC961148	-	-	KC961226
Rhacophorus_feae	-	-	EU924522	EU924550	EU924578
Rhacophorus_gadingensis	KC961102	KC961145	-	-	KC961223
Rhacophorus_gauni	KC961103	KC961146	-	-	KC961224
Rhacophorus_harrissoni	KC961107	KC961149	-	-	KC961227
Rhacophorus_helenae	-	-	-	-	-
Rhacophorus_hongchibaensis	-	-	-	JN688897	JN688906
Rhacophorus_hui	-	-	EU924523	EU924551	EU924579
Rhacophorus_hungfuensis	-	-	-	EU215568	EU215598
Rhacophorus_indonesiensis	-	-	-	-	-
Rhacophorus_kio	GQ285703	GQ285734	GQ285766	EU215562	EU215592
Rhacophorus_lateralis	-	-	-	AY880655	-
Rhacophorus_malabaricus	-	-	AY948912	KU169943	-
Rhacophorus_smaragdinus	-	-	-	-	-
Rhacophorus_minimus	-	-	EU924525	EU924553	EU924581
Rhacophorus_moltrechti	-	-	-	EU215573	EU215603
Rhacophorus_monticola	-	-	-	-	-
Rhacophorus_nigropalmatus	GQ204465	KC961154	GQ204592	GQ204646	KC961210
Rhacophorus_nigropunctatus	GQ285704	GQ285735	GQ285767	EU215563	EU215593
Rhacophorus_norhayatii	AB728214	AB728248	AB728270	AB728287	AB728321
Rhacophorus_omeimontis	-	-	EU924528	EU924556	EU924584
Rhacophorus_orlovi	-	-	-	DQ283778	-
Rhacophorus_pardalis	GQ204466	KC961156	GQ204593	GQ204647	KC961212
Rhacophorus_penanorum	KC961100	KC961143	-	-	KC961222
Rhacophorus_pinglongensis	-	-	-	-	-
Rhacophorus_poecilonotus	-	-	-	-	-
Rhacophorus_pseudomalabaricus	-	-	-	-	-
Rhacophorus_puerensis	-	-	EU924521	EU924549	EU924577
Rhacophorus_reinwardti	GQ204472	-	GQ204601	GQ204656	-
Rhacophorus_rhodopus	-	-	EU924532	EU924560	EU924588
Rhacophorus_bipunctatus	-	-	-	EU215560	EU215590
Rhacophorus_rhodopus	-	-	-	AY844737	-
Rhacophorus_robertingeri	-	-	-	-	-
Rhacophorus_rufipes	KC961106	KC961150	-	-	KC961228
Rhacophorus_schlegelii	-	-	-	-	-
Rhacophorus_translineatus	-	-	-	-	-
Rhacophorus_vampyrus	-	-	-	-	-
Rhacophorus_tuberculatus	-	-	-	-	-
Rhacophorus_wui	-	-	-	JN688896	JN688910
Rhacophorus_zhoukaiyae	KU601449	KU601458	KU601467	KU601476	KU601485
Rhacophorus_lishuiensis	-	-	-	-	-
**Outgroup**					
Microhyla_annectens	AB611943	-	AB611940	-	AB611942

Pelophylax_lateralis	KF991301	-	EF088273	-	-
Pelophylax_nigromaculatus	KX269291	-	HQ902533	KU840635	DQ282932
Pelophylax_ridibundus	-	-	-	-	KT879356
Pelophylax_saharicus	-	-	KP177854	AY148014	KT879338
Rana_kukunoris	-	GQ285748	GQ285780	GQ285798	GQ285816
Rana_catesbeianus	AB612040	AY819103	AB612037	-	AB612039
Rana_temporaria	KC800199	KC800339	AY323776	-	KC800261
Rana_pipiens	-	-	-	DQ283831	EU769542
Rana_tlaloci	KX269251	KX269465	KX269540	-	KX269768
Rana_septentrionalis	KX269240	KX269454	KX269529	-	KX269757
Alcalus_tasanae	-	-	KU243098	KU243108	KU243118
Liurana_xizangensis	-	-	KU243093	KU243102	KU243112
Limnonectes_limborgi	-	-	DQ347286	DQ347407	DQ347194
Occidozyga_martensi	-	-	KU243099	KU243109	KU243119
Odontobatrachus_natator	KP005321	-	KP005354	-	-
Conraua_crassipes	-	-	DQ347244	DQ347364	DQ347148
Blommersia_wittei	EF396018	-	AY323774	AY323743	AY341751
Laliostoma_labrosum	-	-	AY571652	AF249106	AF249169

Dicroglossidae

**Species**	**RAG2**	**RAG1**	**Rhod**	**Tyr**	**12s**
Nanorana_arnoldi	-	-	EU979858	EU979949	EU979777
Nanorana_aenea	HM163645	HM163609	EU979895	EU979986	AF206093
Nanorana_blanfordii	**MW111356**	**MW111370**	**MW111350**	**MW111344**	-
Nanorana_bourreti	HM163630	HM163594	EU979882	EU979973	EU979767
Nanorana_chayuensis	**MW111357**	**MW111371**	**MW111351**	**MW111345**	DQ118466
Nanorana_conaensis	HM163625	HM163589	EU979874	EU979965	EU979774
Nanorana_liebigii	**MW111358**	**MW111372**	**MW111352**	**MW111346**	DQ118455
Nanorana_liebigii	**MW111359**	**MW111373**	**MW111353**	**MW111347**	-
Nanorana_maculosa	HM163624	HM163588	EU979859	EU979950	DQ118468
Nanorana_medogensis	HM163626	HM163590	EU979862	EU979953	DQ118462
Nanorana_parkeri	HM163620	HM163584	EU979873	EU979964	AF206110
Nanorana_pleskei	HM163622	HM163586	EU979870	EU979961	DQ346988
Nanorana_rostandi	**MW111360**	**MW111374**	**MW111354**	**MW111348**	-
Nanorana_quadranus	HM163627	HM163591	EU979887	EU979978	GQ225906
Nanorana_polunini	**MW111361**	**MW111375**	**MW111355**	**MW111349**	-
Nanorana_taihangnica	HM163644	HM163608	EU979893	EU979984	KF199146
Nanorana_unculuanus	HM163631	HM163595	DQ458262	DQ458277	-
Nanorana_yunnanensis	HM163629	HM163593	EU979875	EU979966	DQ118448
Nanorana_ventripunctata	HM163621	HM163585	EU979868	EU979959	-
Quasipaa_boulengeri	HM163641	HM163605	EU979911	EU980002	EU979791
Quasipaa_shini	HM163638	HM163602	EU979907	EU979998	-
Quasipaa_spinosa	HM163642	HM163606	EU979913	EU980004	AF206088
Quasipaa_verrucospinosa	HM163636	HM163599	EU979896	EU979987	EU979790
Quasipaa_yei	HM163632	HM163596	EU979905	EU979996	DQ118445
Euphlyctis_cyanophlyctis	AB488997	DQ347205	AF249111	AF249174	DQ346962
Euphlyctis_hexadactylus	-	MG719916	-	-	NC_014584
Fejervarya_cancrivora	HM163617	HM163581	EU979938	EU980029	-
Fejervarya_limnocharis	HM163616	HM163580	EU979936	EU980027	-
Fejervarya_iskandari	AB488981	AB488954	AB489021	AB277355	-
Fejervarya_triora	AB488982	AB488939	AB489022	AB489003	-
Fejervarya_sakishimensis	AB488985	AB488940	AB489025	AB489004	-
Fejervarya_multistriata	AB500252	AB500220	AB500257	AB500265	-
Hoplobatrachus_crassus	-	DQ347211	AF249109	AF249172	DQ346972
Hoplobatrachus_occipitalis	HM163648	DQ347217	DQ283787	EU980026	DQ346979
Hoplobatrachus_rugulosus	-	DQ347222	DQ458257	EU980024	DQ346985
Hoplobatrachus_tigerinus	AB488998	AB488958	AB489039	AB277358	AB290422
Limnonectes_finchi	-	-	AY322230	AY322355	AY322306
Limnonectes_fragilis	HM163647	HM163611	DQ458270	EU980020	AY703867
Limnonectes_fujianensis	HM163618	HM163582	DQ458260	DQ458275	AY703865
Limnonectes_grunniens	-	-	DQ283885	EU980023	-
Limnonectes_kuhlii	-	DQ347232	AF249116	EU980019	AY703869

Limnonectes_deinodon	AB489000	AB488960	AB489041	AB277359	AF183125
Limnonectes_limborgi	-	DQ347286	DQ347407	-	DQ347061
Limnonectes_magnus	-	-	DQ347373	DQ347157	DQ347022
Limnonectes_modestus	-	-	EU979930	EU980021	AY313766
Limnonectes_poilani	-	-	DQ283997	DQ282989	DQ283378
Minervarya_pierrei	AB490160	AB488942	AB489027	AB489006	AB488865
Minervarya_caperata	AB488990	AB488946	AB489031	AB489010	-
Minervarya_greenii	AB488988	AB488944	AB489029	AB489008	AB488868
Minervarya_syhadrensis	AB488989	AB488945	AB489030	AB489009	AF249011
Sphaerotheca_breviceps	-	-	AF249110	DQ282927	AF161039
Sphaerotheca_dobsonii	AB488999	AB488959	AB489040	AB277357	AB277290
Sphaerotheca_pluvialis	-	DQ347214	-	AF249173	-
Sphaerotheca_rolandae	-	-	-	-	-
Nannophrys_ceylonensis	-	AY948917	AF249112	AF249175	DQ346975
Ingerana_tenasserimensis	-	DQ347258	AY322236	AY322344	AY322308
Ingerana_borealis	-	KU243100	KU243110	KU243120	KU243091
Occidozyga_baluensis	-	-	DQ283844	DQ282936	DQ283143
Occidozyga_laevis	-	DQ347254	AY322227	AY322342	DQ347024
Occidozyga_lima	AB489001	DQ019503	DQ283901	DQ282951	DQ283224
Occidozyga_martensii	-	KU243099	DQ458266	DQ458283	DQ283357
**Outgroup**					
Staurois_nanator	-	DQ347250	DQ347371	DQ347155	-
Rana_sylvatica	DQ019546	DQ347278	DQ347397	DQ347185	-
Boophis_doulioti	DQ019519	AY571643	AY341792	-	-
Boophis_tephraeomystax	-	DQ347234	AF249105	-	DQ347000
Blommersia_wittei	AY323795	AY323774	AY880667	AY341751	AY880536
Mantella_aurantiaca	AY723547	AY723530	AY263281	DQ282901	DQ283035
Rhacophorus_dugritei	-	GQ285768	EU215571	EU215601	EF564471
Petropedetes_martiensseni	-	DQ347289	DQ347410	DQ347197	DQ347064
Aubria_subsigillata	-	-	DQ283865	DQ282975	-
Strongylopus_bonaespei	-	DQ347288	DQ347409	DQ347196	DQ347063
Amietia_angolensis	DQ019515	DQ347257	DQ347377	DQ347163	DQ347029
Pelophylax_nigromaculatus	KX269656	KX269581	DQ360014	DQ360045	KX269216
Rana_pipiens	-	DQ347265	DQ347384	EU769542	DQ347039
Rana_catesbeianus	AB612038	AB612037	-	AB612039	NC_022696
Rana_temporaria	AY323803	AY323776	-	KC800261	AB058864
Liurana_alpina	-	KU243094	KU243104	KU243114	KU243085
Liurana_xizangensis	-	KU243093	KU243102	KU243112	KU243084
Cornufer_guentheri	-	-	DQ284031	DQ283024	DQ283198
Platymantis_dorsalis	-	DQ347246	DQ347367	DQ347151	DQ347199
Platymantis_hazelae	-	DQ347248	DQ347369	DQ347153	DQ347019
Platymantis_naomii	-	DQ347249	DQ347370	DQ347154	DQ347201
Platymantis_papuensis	-	DQ347268	DQ347387	DQ347175	DQ347042
**Species**	**16s**				

Nanorana_arnoldi	EU979837
Nanorana_aenea	AF206474
Nanorana_blanfordii	-
Nanorana_bourreti	EU979827
Nanorana_chayuensis	DQ118510
Nanorana_conaensis	EU979834
Nanorana_liebigii	DQ118499
Nanorana_liebigii	-
Nanorana_maculosa	DQ118512
Nanorana_medogensis	DQ118506
Nanorana_parkeri	AF206491
Nanorana_pleskei	AF206492
Nanorana_rostandi	-
Nanorana_quadranus	GQ225932
Nanorana_polunini	-
Nanorana_taihangnica	KF199146
Nanorana_unculuanus	DQ118491
Nanorana_yunnanensis	DQ118492
Nanorana_ventripunctata	EU979839
Quasipaa_boulengeri	EU979851
Quasipaa_shini	DQ118487
Quasipaa_spinosa	AF206469
Quasipaa_verrucospinosa	EU979813
Quasipaa_yei	DQ118488
Euphlyctis_cyanophlyctis	AY014366
Euphlyctis_hexadactylus	NC_014584
Fejervarya_cancrivora	EU979849
Fejervarya_limnocharis	AF206466
Fejervarya_iskandari	AB277303
Fejervarya_triora	AB488883
Fejervarya_sakishimensis	AB488886
Fejervarya_multistriata	AB488884
Hoplobatrachus_crassus	AF249044
Hoplobatrachus_occipitalis	DQ283059
Hoplobatrachus_rugulosus	AF206465
Hoplobatrachus_tigerinus	FJ008057
Limnonectes_finchi	AY322295
Limnonectes_fragilis	AY703854
Limnonectes_fujianensis	DQ118518
Limnonectes_grunniens	U66125
Limnonectes_kuhlii	DQ283370
Limnonectes_deinodon	AB277306
Limnonectes_limborgi	AF261269
Limnonectes_magnus	AY313706

Limnonectes_modestus	AY313766
Limnonectes_poilani	DQ283378
Minervarya_pierrei	AB488888
Minervarya_caperata	AB355845
Minervarya_greenii	AB488891
Minervarya_syhadrensis	AY841748
Sphaerotheca_breviceps	AF249042
Sphaerotheca_dobsonii	AB277305
Sphaerotheca_pluvialis	AF249042
Sphaerotheca_rolandae	GU191122
Nannophrys_ceylonensis	AF215394
Ingerana_tenasserimensis	AY322302
Ingerana_borealis	-
Occidozyga_baluensis	DQ283143
Occidozyga_laevis	AY322300
Occidozyga_lima	AF206497
Occidozyga_martensii	DQ283357
**Outgroup**	
Staurois_nanator	-
Rana_sylvatica	-
Boophis_doulioti	-
Boophis_tephraeomystax	DQ283032
Blommersia_wittei	-
Mantella_aurantiaca	DQ283035
Rhacophorus_dugritei	EF564541
Petropedetes_martiensseni	DQ347346
Aubria_subsigillata	DQ283173
Strongylopus_bonaespei	DQ347345
Amietia_angolensis	DQ347318
Pelophylax_nigromaculatus	DQ359991
Rana_pipiens	Y10945
Rana_catesbeianus	NC_022696
Rana_temporaria	AB058882
Liurana_alpina	-
Liurana_xizangensis	-
Cornufer_guentheri	-
Platymantis_dorsalis	DQ347308
Platymantis_hazelae	DQ347310
Platymantis_naomii	DQ347311
Platymantis_papuensis	DQ347326

Ceratobatrachidae

**Species**	**12s**	**16s**	**RAG1**	**Tyr**	**POMC**
Liurana_alpina	KU243086	KU243086	KU243094	KU243114	-
Liurana_medogensis	-	-	-	KU243113	-
Liurana_vallecula	-	-	-	MK462155	-
Liurana_vallecula	-	-	-	MK462156	-
Liurana_xizangensis	KU243083	KU243083	KU243093	KU243112	-
Alcalus_baluensis	DQ283142	DQ283142	DQ347270	DQ347177	-
Alcalus_tasanae	KU243089	KU243089	KU243096	KU243116	-
Alcalus_mariae	KP298038	KP298038	KP298263	-	KP298166
Alcalus_rajae	-	KP298028	-	KP298326	KP298163
Cornufer_guentheri	KP298021	-	KP298261	DQ347179	KP298156
Cornufer_guppyi	KP298024	KP298024	DQ347269	DQ347176	KP298159
Cornufer_minutus	KP298016	KP298016	KP298256	KP298318	KP298151
Cornufer_vertebralis	KP298018	-	KP298258	KP298320	KP298153
Cornufer_bufoniformis	KP298022	KP298022	-	-	KP298157
Cornufer_malukuna	KP298025	KP298025	-	KP298323	KP298160
Cornufer_wolfi	KP298033	KP298033	-	KP298328	KP298164
Cornufer_pelewensis	KP298045	KP298045	-	KP298330	KP298168
Cornufer_adiastolus	KP298052	KP298052	-	KP298331	KP298169
Cornufer_boulengeri	KP298056	KP298056	KP298266	KP298333	KP298173
Cornufer_gilliardi	KP298060	KP298060	-	KP298335	KP298177
Cornufer_occidentalis	KP298071	KP298071	-	KP298339	KP298187
Cornufer_papuensis	KP298072	KP298072	-	KP298340	KP298188
Cornufer_vitianus	KP298079	KP298079	KP298271	KP298343	KP298193
Cornufer_caesiops	KP298081	KP298081	-	KP298345	KP298195
Cornufer_bufonulus	KP298082	KP298082	-	KP298346	KP298196
Cornufer_hedigeri	KP298083	KP298083	KP298273	KP298347	KP298197
Cornufer_akarithymus	KP298084	KP298084	-	KP298348	KP298198
Cornufer_solomonis	KP298088	KP298088	KP298275	KP298350	KP298202
Cornufer_cheesemanae	KP298096	KP298096	-	KP298355	KP298208
Cornufer_myersi	KP298121	KP298121	KP298298	KP298373	KP298231
Cornufer_parilis	KP298122	KP298122	KP298299	KP298374	KP298232
Cornufer_weberi	KP298149	KP298149	-	KP298394	KP298254
Platymantis_hazelae	KP298061	KP298061	DQ347248	DQ347153	KP298178
Platymantis_corrugatus	KP298058	KP298058	KP298267	KP298334	KP298175
Platymantis_insulatus	KP298062	KP298062	-	KP298337	KP298179
Platymantis_isarog	KP298063	KP298063	-	KP298338	KP298180
Platymantis_banahao	KP298089	KP298089	-	KP298351	KP298203
Platymantis_bayani	KP298091	KP298091	KP298276	KP298352	KP298204
Platymantis_cagayanensis	KP298094	KP298094	KP298278	KP298353	KP298206
Platymantis_diesmosi	KP298097	KP298097	KP298280	KP298356	KP298209
Platymantis_dorsalis	KP298098	KP298098	KP298281	KP298357	KP298210

Platymantis_guentheri	KP298103	KP298103	KP298286	KP298361	KP298215
Platymantis_indeprensus	KP298108	KP298108	KP298290	KP298366	KP298220
Platymantis_lawtoni	KP298114	KP298114	KP298293	KP298369	-
Platymantis_levigatus	KP298115	KP298115	KP298294	KP298370	KP298225
Platymantis_luzonensis	KP298117	KP298117	-	KP298371	KP298227
Platymantis_mimulus	KP298120	KP298120	KP298297	-	KP298230
Platymantis_negrosensis	KP298123	KP298123	KP298300	KP298375	KP298233
Platymantis_naomii	KP298124	KP298124	KP298301	KP298376	KP298234
Platymantis_paengi	KP298125	KP298125	KP298302	KP298377	KP298235
Platymantis_panayensis	KP298126	KP298126	KP298303	KP298378	KP298236
Platymantis_polillensis	KP298128	KP298128	KP298305	KP298380	-
Platymantis_pseudodorsalis	KP298129	KP298129	-	KP298381	KP298238
Platymantis_pygmaeus	KP298130	KP298130	KP298306	KP298382	KP298239
Platymantis_rabori	KP298131	KP298131	KP298307	KP298383	KP298240
Platymantis_sierramadrensis	KP298139	KP298139	-	KP298389	KP298248
Platymantis_spelaeus	KP298143	KP298143	KP298313	KP298390	KP298251
Platymantis_subterrestris	KP298146	KP298146	KP298315	KP298392	KP298253
Platymantis_taylori	KP298148	KP298148	KP298316	KP298393	-
**Outgroup**					
Occidozyga_lima	DQ283224	AF206497	DQ019503	DQ282951	-
Nanorana_parkeri	AF206110	AF206491	DQ019501	EU979964	-
Fejervarya_limnocharis	AB277282	AF206466	-	EU980027	AB526646
Rana_sylvatica	DQ347052	DQ283387	DQ019511	DQ347185	-
Rana_temporaria	AB058864	AB058882	AY323776	KC800261	KC800339
Rana_pipiens	Y10945	X86284	-	EU769542	-
Rana_catesbeianus	NC_022696	NC_022696	AB612037	-	AY819103
Rana_tlaloci	AY779234	AY779234	KX269540	KX269768	KX269465
Rana_septentrionalis	KX269179	KX269179	KX269529	KX269757	KX269454
Rhacophorus_dugritei	EF564471	EF564541	GQ285768	EU215601	GQ285736
Mantidactylus_femoralis	AY843698	AY843698	-	DQ282900	-
Boophis_doulioti	AY341608	AY341663	AY571643	-	-
Pelophylax_nigromaculatus	KT878718	KT878718	HQ902533	DQ282932	-
Phrynodon_sandersoni	DQ283083	DQ283083	GU457749	DQ282973	-

Bufonidae

Species	12s	16s	CXCR4	NCX1	POMC
Adenomus_kelaartii	FJ882780	FJ882780	EF107447	EF107221	-
Anaxyrus_americanus	FJ882827	DQ158426	DQ306520	FJ882676	DQ158268
Anaxyrus_quercicus	DQ158484	DQ158484	DQ306549	-	DQ158325
Anaxyrus_terrestris	FJ882829	FJ882829	DQ306537	FJ882677	DQ158330
Ansonia_hanitschi	AB331710	FJ882794	FJ882695	FJ882642	-
Ansonia_leptopus	FJ882795	FJ882795	FJ882697	FJ882644	-
Ghatophryne_ornata	FJ882797	FJ882797	FJ882694	FJ882641	-
Atelopus_peruensis	DQ158419	DQ158419	DQ306495	-	AY819078
Atelopus_spumarius	DQ283260	DQ283260	GU183852	GU183854	-
Barbarophryne_brongersmai	FJ882817	FJ882817	FJ882718	FJ882663	-
Bufo_aspinius	AF160770	AF160787	-	-	-
Bufo_bankorensis	AB159476	AB159589	-	-	-
Bufo_bufo	AY325988	FJ882806	DQ306504	FJ882650	DQ158280
Bufo_cryptotympanicus	AF160771	AF160789	-	-	-
Bufo_gargarizans	NC_008410	NC_008410	FJ882707	FJ882653	-
Bufo_japonicus	AB159452	AB159565	AB612061	AB612062	-
Bufo_stejnegeri	AF218710	NC_027686	-	-	-
Bufo_tibetanus	AF160766	AF160784	-	-	-
Bufo_torrenticola	AB159465	AB159578	JN653312	-	JN653305
Bufo_tuberculatus	AF160765	AF160783	-	-	-
Bufo_tuberospinius	AF160769	AF160788	-	-	-
Bufo_verrucosissimus	FJ882807	FJ882807	FJ882705	FJ882651	-
Bufotes_balearicus	-	EU497437	-	-	-
Bufotes_boulengeri	-	EU497492	-	-	-
Bufotes_oblongus	AF160777	AF160796	-	-	-
Bufotes_pewzowi	GQ489032	-	-	-	-
Bufotes_siculus	-	EU497446	-	-	-
Bufotes_variabilis	GQ489041	-	-	-	-
Bufotes_viridis	AY680267	FJ882813	FJ882714	FJ882659	-
Bufotes_zamdaensis	-	-	-	-	-
Bufotes_zamdaensis	-	-	-	-	-
Bufotes_latastii	-	-	-	-	-
Bufotes_baturae	-	-	-	-	-
Capensibufo_rosei	AF220864	AF220911	-	-	-
Capensibufo_tradouwi	AF220865	AF220912	-	-	-
Churamiti_maridadi	FJ882769	FJ882769	FJ882715	FJ882660	-
Dendrophryniscus_minutus	AY843582	AY843582	DQ306496	-	AY819081
Didynamipus_sjostedti	AY325991	AY325991	-	-	-
Duttaphrynus_atukoralei	FJ882835	FJ882835	FJ882682	FJ882629	-
Duttaphrynus_brevirostris	FJ882786	FJ882786	FJ882686	FJ882633	-
Duttaphrynus_crocus	FJ882789	FJ882789	FJ882690	FJ882637	-

Duttaphrynus_dhufarensis	FJ882837	FJ882837	FJ882679	FJ882626	-
Duttaphrynus_himalayanus	**MW111525**	**MW111525**	FJ882688	FJ882635	-
Duttaphrynus_hololius	FJ882781	FJ882781	FJ882680	FJ882627	-
Duttaphrynus_melanostictus	AB331714	FJ882791	DQ306508	AY948805	DQ158317
Duttaphrynus_parietalis	FJ882784	FJ882784	FJ882684	FJ882631	-
Duttaphrynus_scaber	FJ882785	FJ882785	FJ882683	FJ882630	-
Duttaphrynus_stomaticus	FJ882787	FJ882787	FJ882681	FJ882628	-
Duttaphrynus_stuarti	**MW111526**	**MW111526**	FJ882689	FJ882636	-
Epidalea_calamita	FJ882809	FJ882809	FJ882709	FJ882655	-
Incilius_alvarius	AY325984	DQ283269	DQ306516	-	AY819079
Incilius_coccifer	DQ158443	DQ158443	DQ306526	-	DQ158284
Ingerophrynus_divergens	FJ882802	FJ882802	FJ882701	FJ882648	-
Ingerophrynus_galeatus	FJ882767	FJ882768	DQ306506	FJ882649	DQ158293
Ingerophrynus_macrotis	DQ158468	FJ882803	DQ306525	-	DQ158309
Ingerophrynus_biporcatus	AY325987	KY555646	KY555668	KX192102	-
Ingerophrynus_parvus	KF664931	MG935843	KF665955	-	KU183067
Ingerophrynus_philippinicus	AF375488	-	-	-	-
Bufoides_meghalayanus	KT991331	KT991342	-	-	-
Leptophryne_borbonica	FJ882799	AB331716	EF107450	EF107224	-
Melanophryniscus_klappenbachi	AY843699	AY843699	-	-	-
Melanophryniscus_stelzneri	FJ882853	FJ882853	DQ306494	AY948822	DQ158263
Mertensophryne_micranotis	FJ882821	FJ882821	EF107491	EF107271	-
Mertensophryne_uzunguensis	FJ882819	FJ882819	FJ882720	FJ882665	-
Nannophryne_apolobambica	DQ158494	DQ158494	DQ306515	-	DQ158335
Nannophryne_cophotis	DQ158446	DQ158446	DQ306540	-	DQ158287
Nectophryne_afra	EU394535	EU394535	-	-	-
Nectophrynoides_tornieri	FJ882815	FJ882815	EF107490	EF107270	-
Nectophrynoides_viviparus	FJ882816	FJ882816	FJ882716	FJ882661	-
Osornophryne_guacamayo	AY819334	AY326036	-	-	AY819083
Pedostibes_hosii	FJ882804	AY325993	EF107449	EF107223	-
Pedostibes_tuberculosus	FJ882793	FJ882793	FJ882693	FJ882640	-
Pelophryne_misera	FJ882800	FJ882800	FJ882700	FJ882647	-
Pelophryne_signata	FJ882801	FJ882801	FJ882699	FJ882646	-
Peltophryne_lemur	DQ158465	DQ158465	DQ306513	-	DQ158306
Phrynoidis_asper	DQ158432	AF124109	DQ306503	-	DQ158273
Phrynoidis_juxtasper	FJ882805	FJ882805	DQ306542	FJ882656	DQ158304
Poyntonophrynus_dombensis	AF220857	AF220907	-	-	-
Rhaebo_haematiticus	DQ158461	DQ158461	DQ306501	-	DQ158277
Rhinella_nattereri	FJ882774	AY028496	DQ306557	FJ882673	DQ158298
Rhinella_schneideri	FJ882831	DQ158480	DQ306528	FJ882674	DQ158322
Rhinella_marina	-	-	-	-	-
Sabahphrynus_maculatus	AB331718	AB331718	-	-	-
Schismaderma_carens	AY325997	AY325997	FJ882717	FJ882662	DQ158266
Sclerophrys_brauni	FJ882822	FJ882822	DQ306514	EF107272	DQ158279

Sclerophrys_garmani	FJ882823	FJ882823	DQ306547	FJ882668	DQ158294
Strauchbufo_raddei	AF160776	GU183855	-	-	-
Vandijkophrynus_amatolicus	AF220851	AF220898	-	-	-
Werneria_mertensiana	DQ283348	DQ283348	-	-	-
Wolterstorffina_parvipalmata	FJ882818	FJ882818	FJ882719	FJ882664	-
Xanthophryne_koynayensis	FJ882782	FJ882782	FJ882691	FJ882638	-
**Outgroup**					
Odontophrynus_occidentalis	FJ882743	FJ882744	-	AY948835	-
Leptodactylus_melanonotus	DQ347060	AY943237	AY364194	AY948838	-
Leptodactylus_ocellatus	AY843688	-	DQ306492	-	DQ158259

**Species**	**RAG1**	**ND2**
Adenomus_kelaartii	EF107284	FJ882780
Anaxyrus_americanus	DQ158352	FJ882827
Anaxyrus_quercicus	DQ158403	-
Anaxyrus_terrestris	-	FJ882829
Ansonia_hanitschi	-	FJ882794
Ansonia_leptopus	-	FJ882795
Ghatophryne_ornata	-	FJ882797
Atelopus_peruensis	-	-
Atelopus_spumarius	-	DQ068447
Barbarophryne_brongersmai	-	FJ882817
Bufo_aspinius	-	-
Bufo_bankorensis	-	-
Bufo_bufo	EU497611	FJ882806
Bufo_cryptotympanicus	-	-
Bufo_gargarizans	-	FJ882843
Bufo_japonicus	AB612057	-
Bufo_stejnegeri	-	NC_027686
Bufo_tibetanus	-	NC_020048
Bufo_torrenticola	-	-
Bufo_tuberculatus	-	-
Bufo_tuberospinius	-	-
Bufo_verrucosissimus	-	FJ882807
Bufotes_balearicus	EU497608	DQ629598
Bufotes_boulengeri	EU497613	DQ629608
Bufotes_oblongus	-	DQ629601
Bufotes_pewzowi	GU064322	DQ629603
Bufotes_siculus	EU497609	DQ629608
Bufotes_variabilis	EU497606	DQ629600
Bufotes_viridis	EU497603	FJ882813
Bufotes_zamdaensis	-	**MW111460**
Bufotes_zamdaensis	-	**MW111461**
Bufotes_latastii	-	DQ629599
Bufotes_baturae	-	DQ629604

Capensibufo_rosei	-	HG321482
Capensibufo_tradouwi	-	FN650139
Churamiti_maridadi	-	KY555706
Dendrophryniscus_minutus	-	-
Didynamipus_sjostedti	-	-
Duttaphrynus_atukoralei	-	FJ882835-36
Duttaphrynus_brevirostris	-	FJ882786
Duttaphrynus_crocus	-	FJ882789
Duttaphrynus_dhufarensis	-	FJ882837-38
Duttaphrynus_himalayanus	-	FJ882790
Duttaphrynus_hololius	-	FJ882781
Duttaphrynus_melanostictus	EU712821	FJ882791
Duttaphrynus_parietalis	-	FJ882784
Duttaphrynus_scaber	-	FJ882785
Duttaphrynus_stomaticus	-	FJ882787
Duttaphrynus_stuarti	-	FJ882788
Epidalea_calamita	EU497610	FJ882809
Incilius_alvarius	DQ158351	JN868006
Incilius_coccifer	DQ158366	JN868016
Ingerophrynus_divergens	EU712818	FJ882802
Ingerophrynus_galeatus	DQ158374	FJ882768
Ingerophrynus_macrotis	DQ158388	KY555693
Ingerophrynus_biporcatus	KY555715	-
Ingerophrynus_parvus	KF666331	KY555702
Ingerophrynus_philippinicus	-	-
Bufoides_meghalayanus	-	-
Leptophryne_borbonica	EF107287	FJ882799
Melanophryniscus_klappenbachi	-	-
Melanophryniscus_stelzneri	-	FJ882853
Mertensophryne_micranotis	-	FJ882821
Mertensophryne_uzunguensis	-	FJ882819
Nannophryne_apolobambica	DQ158410	-
Nannophryne_cophotis	DQ158369	-
Nectophryne_afra	-	-
Nectophrynoides_tornieri	EF107329	FJ882815
Nectophrynoides_viviparus	-	FJ882816
Osornophryne_guacamayo	-	-
Pedostibes_hosii	EF107286	FJ882804
Pedostibes_tuberculosus	-	FJ882793
Pelophryne_misera	-	FJ882800
Pelophryne_signata	-	FJ882801
Peltophryne_lemur	DQ158386	-
Phrynoidis_asper	DQ158356	KY555704
Phrynoidis_juxtasper	DQ158385	FJ882805

Poyntonophrynus_dombensis	-	AF463794
Rhaebo_haematiticus	DQ158383	JN868019
Rhinella_nattereri	DQ158380	-
Rhinella_schneideri	DQ158399	FJ882831
Rhinella_marina	-	GU907290
Sabahphrynus_maculatus	-	-
Schismaderma_carens	-	FJ882849-50
Sclerophrys_brauni	EF107331	FJ882822
Sclerophrys_garmani	DQ158375	FJ882823
Strauchbufo_raddei	-	NC_028424
Vandijkophrynus_amatolicus	-	-
Werneria_mertensiana	-	-
Wolterstorffina_parvipalmata	-	FJ882818
Xanthophryne_koynayensis	-	FJ882782
**Outgroup**		
Odontophrynus_occidentalis	AY948934	JX564880
Leptodactylus_melanonotus	AY364224	JX564873
Leptodactylus_ocellatus	DQ158343	-

Megophryidae

Species	16s	COI	BDNF	RAG1	Rhod
Megophrys_sangzhiensis	KX811856	KX812117	KX811961	KX812226	KX812343
Megophrys_spinata	KX811861	KX812121	KX811965	KX812229	KX812345
Megophrys_binlingensis	KX811852	KX812115	KX811970	KX812221	KX812341
Megophrys_jingdongensis	KX811872	KX812131	KX811973	KX812232	KX812350
Megophrys_daweimontis	KX811866	KX812124	KX811968	KX812247	KX812359
Megophrys_wuliangshanensis	KX811881	KX812129	KX811958	KX812258	KX812357
Megophrys_omeimontis	KX811884	KX812136	KX811967	KX812223	KX812347
Megophrys_binchuanensis	KX811848	KX812111	KX811951	KX812218	KX812337
Megophrys_palpebralespinosa	KX811888	KX812137	KX811994	KX812236	KX812363
Megophrys_huangshanensis	KX811819	KX812106	KX811982	KX812215	KX812308
Megophrys_boettgeri	KX811814	KX812104	KX811980	KX812213	KX812305
Megophrys_jinggangensis	KX811840	KX812108	KX811986	KX812242	KX812328
Megophrys_baolongensis	KX811813	KX812093	KX811945	KX812202	KX812318
Megophrys_tuberogranulatus	KX811823	KX812095	KX811979	KX812209	KX812331
Megophrys_wushanensis	KX811838	KX812094	KX811984	KX812203	KX812319
Megophrys_lini	KX811842	KX812110	KX811988	KX812244	KX812394
Megophrys_brachykolos	KX811897	KX812150	KX811985	KX812217	KX812395
Megophrys_minor	KX811896	KX812145	KX812045	KX812253	KX812325
Megophrys_maosonensis	KX811780	KX812080	KX812024	KX812195	KX812375
Megophrys_mangshanensis	KX811790	KX812079	KX812025	KX812194	KX812377
Megophrys_major	KX811769	KX812090	KX812015	KX812186	KX812372
Megophrys_glandulosa	KX811761	KX812074	KX812010	KX812183	KX812298
Megophrys_medogensis	KX811767	KX812082	KX812017	KX812197	KX812301
Megophrys_zhangi	KX811765	KX812084	KX812026	KX812200	KX812303
Megophrys_parva	KX811797	KX812072	KX812006	KX812181	KX812295
Megophrys_montana	KX811927	KX812163	KX811940	KX812281	KX812390
Megophrys_nasuta	KX811921	KX812054	KX811935	KX812171	KX812289
Megophrys_stejnegeri	KX811922	KX812052	KX811936	KX812172	KX812399
Megophrys_ligayae	KX811919	KX812051	KX811933	KX812169	KX812287
Megophrys_wawuensis	KX811902	KX812062	KX812041	KX812271	KX812314
Megophrys_gigantica	KX811898	KX812058	KX811941	KX812270	KX812312
Megophrys_shapingensis	KX811904	KX812060	KX811943	KX812274	KX812316
Megophrys_aceras	KX811925	KX812159	KX812030	KX812262	KX812310
Megophrys_pachyproctus	KX811908	KX812153	KX811998	KX812265	KX812383
Megophrys_flavipunctata	MH647518	MH647536	-	MH647549	-
Megophrys_himalayana	MH647527	-	-	MH647554	-
Megophrys_oreocrypta	MH647521	-	-	MH647547	-
Megophrys_oropedion	KY022317	MH647534	-	KY022361	-
Megophrys_periosa	MH647524	MH647529	-	MH647555	-
Megophrys_serchhipii	KY022323	MH647532	-	KY022366	-
Megophrys_vegrandis	KY022305	MH647530	-	KY022349	-

Megophrys_zunhebotoensis	KY022322	-	-	KY022368	-
Megophrys_ancrae	KY022318	MH647531	-	KY022350	-
Megophrys_robusta	MH647513	MH647535	-	KY022365	-
Megophrys_megacephala	KY022315	MH647533	-	KY022357	-
Ophryophryne_microstoma	KX811914	KX812156	KX812039	KX812277	KX812397
Ophryophryne_hansi	KX811913	KX812155	KX812036	KX812276	KX812396
Borneophrys_edwardinae	KX811918	KX812050	KX811932	KX812168	KX812286
Brachytarsophrys_carinense	KX811811	KX812057	KX811939	KX812268	KX812392
Brachytarsophrys_feae	KX811810	KX812056	KX811938	KX812269	KX812393
Leptolalax_oshanensis	KX811928	KX812166	KX812048	KX812284	KX812402
Leptolalax_ventripunctatus	KX811929	KX812167	KX812049	KX812285	KX812403
Scutiger_boulengeri	KY310750	KY310860	-	KY311026	-
Scutiger_chintingensis	KY310769	KY310878	-	EF397301	-
Scutiger_glandulatus	KY310770	KY310879	-	KY311044	-
Scutiger_mammatus	KY310775	KY310884	-	KY311050	-
Scutiger_nepalensis	KY310776	KY310885	-	KY311051	-
Scutiger_occidentalis	-	KY310901	-	KY311066	-
Scutiger_sikkimensis	KY310802	KY310905	-	KY311073	-
Scutiger_ningshanensis	NC_031426	NC_031426	-	-	-
Scutiger_spinosus	-	KU243055	-	-	-
Scutiger_liupanensis	KX352261	**MW021398**	-	**MW111376**	-
Scutiger_tuberculatus	MF805589	**MW021397**	-	**MW111379**	-
Scutiger_gongshanensis	-	**MW021395**	-	**MW111380**	-
Scutiger_nyingchiensis	KY310796	KU243056	-	**MW111377**	-
Scutiger_wuguanfui	-	KU243061	-	**MW111378**	-
Leptobrachium_bompu	**MW111515**	-	-	-	-
Leptobrachium_bompu	**MW111516**	-	-	-	-
Leptobrachium_hainanense	AB530447	JN700832	-	EF544338	-
Leptobrachium_banae	AB530452	-	-	EF544314	-
Leptobrachium_ngoclinhense	AB530450	-	-	EF544360	-
Leptobrachium__boringii	KX811930	KX812164	KX812046	MH056123	KX812400
Leptobrachium_huashen_	KX811931	KX812165	MH056008	MH056122	MH056143
Leptobrachium_smithi	AB530432	MG935560	-	-	-
Leptobrachium_hasseltii	AB530422	-	-	EF672278	-
Leptobrachium_hendricksoni	AB530418	MG386477	-	-	-
**Outgroup**					
Pelobates_syriacus	AY236807	-	-	KX208696	-
Pelobates_cultripes	AJ871086	AJ871086	-	AY323758	-
Pelobates_varaldii	AY236808	-	-	-	AY341815
Pelodytes_cf._punctatus	NC_020000	NC_020000	JF703235	AY583343	-
Pelodytes_ibericus	JX564882	JX564882	-	KX984226	-
Scaphiopus_hurterii	AY236828	-	-	EF535900	-
Scaphiopus_holbrookii	AB612078	KU985774	AB612074	AB612071	DQ283852
Scaphiopus_couchii	AY236826	-	-	KX208786	AY323738

Agamidae

Species	ND1–ND2	RAG1	R35	ND4	Cytb
Amphibolurus_muricatus	AF128468	-	-	-	-
Chelosania_brunnea	AF128465	JF806190	JF804583	-	-
Ctenophorus_adelaidensis	AF128471	JF806192	JF804602	-	-
Ctenophorus_isolepis	AF375629	JF806193	JF804587	-	-
Diporiphora_nobbi	AY132999	-	-	AY281164	-
Diporiphora_winneckei	AY133012	-	-	-	-
Intellagama_lesueurii	AF128466	AY662581	JF804599	-	-
Moloch_horridus	AF128467	JF806197	JF804595	-	-
Physignathus_cocincinus	U82690	AY662582	HQ876322	-	AB263945
Pogona_vitticeps	AY133026	JF806200	JF804600	NC_006922	NC_006922
Rankinia_diemensis	AF375619	-	-	-	-
Tympanocryptis_lineata	EU727400	-	-	-	-
Tympanocryptis_tetraporophora	DQ529269	-	-	-	-
Chlamydosaurus_kingii	EF090489	JF806191	JF804584	EF090423	NC_009421
Hypsilurus_boydii	AY133013	JF806196	JF804592	-	-
Gowidon_temporalis	AY133002	-	-	-	-
Acanthocercus_atricollis	-	-	JX839080	JX857596	-
Agama_agama	AF128504	EU402825	HQ876321	AF443225	-
Agama_anchietae	GQ242169	-	JX839094	GU128483	AF355566
Xenagama_taylori	NC_008065	KP994938	JX839174	NC_008065	NC_008065
Trapelus_agilis	AF128509	JF806201	JF804606	-	-
Trapelus_mutabilis	-	-	JX839170	GU128501	-
Pseudotrapelus_sinaitus	AF128507	-	JX839168	NC_013603	AB116956
Bufoniceps_laungwalaensis	DQ008214	-	-	-	-
Coryphophylax_subcristatus	-	-	-	-	-
Stellagama_stellio	AF128516	-	JX839166	-	-
Paralaudakia_caucasia	AF172705	-	-	AY053998	KF691617
Paralaudakia_himalayana	AF028676	KJ363481	-	-	-
Paralaudakia_lehmanni	GQ242233	KJ363482	-	KF691670	KF691618
Laudakia_wui	-	-	-	KU926290	-
Laudakia_tuberculata	AF128514	-	-	KU926285	-
Laudakia_nupta	AF128513	-	-	KF691667	-
Laudakia_melanura	-	-	-	KX131009	-
Laudakia_sacra	AF128515	-	-	KU926293	-
Laudakia_papenfussi	-	-	-	KU926288	-
Phrynocephalus_arabicus	KF691651	KJ363507	-	KF691675	-
Phrynocephalus_axillaris	KC551439	KC551397	-	KC551305	KF691635
Phrynocephalus_erythrurus	GQ141231	KJ363485	-	KF691684	KF691632
Phrynocephalus_forsythii	KF691661	KJ363484	-	AY054017	KF691633
Phrynocephalus_frontalis	AY396592	KC551407	-	KC551315	AY053909
Phrynocephalus_guinanensis	KC551454	KC551412	-	KC551320	-

Phrynocephalus_guttatus	GQ242203	KJ363491	-	KF691693	KF691640
Phrynocephalus_helioscopus	KF691658	KJ363502	-	KF691683	KF691630
Phrynocephalus_interscapularis	AF128517	KJ363510	-	KF691671	KF691620
Phrynocephalus_maculatus	KF691650	-	-	KF691674	KF691623
Phrynocephalus_mystaceus	AF128518	GQ242237	JF804596	AY054055	KF691626
Phrynocephalus_reticulatus	KF691652	KJ363503	-	KF691676	KF691624
Phrynocephalus_ornatus	KF691649	KJ363509	-	KF691673	KF691622
Phrynocephalus_persicus	KF691657	KJ363500	-	KF691681	KF691631
Phrynocephalus_przewalskii	AY396586	KJ363495	-	AY054061	AY053941
Phrynocephalus_putjatai	HM629331	KJ363487	-	KF691686	KF691634
Phrynocephalus_raddei	KF691653	KJ363504	-	KF691678	KF691625
Phrynocephalus_rossikowi	-	-	-	MG739301	MG739302
Phrynocephalus_scutellatus	KF691655	KJ363508	-	KF691677	KF691619
Phrynocephalus_strauchi	KF691654	KJ363505	-	KF691679	-
Phrynocephalus_theobaldi	KC551462	KC551419	-	KC551328	-
Phrynocephalus_versicolor	KF691664	KJ363497	-	KF691696	KF691641
Phrynocephalus_vlangalii	EF375649	-	-	EU294095	-
Draco_biaro	AF288277	-	-	-	-
Draco_bimaculatus	AF288241	-	-	-	-
Draco_blanfordii	AF288242	JF806194	JF804589	-	-
Draco_cornutus	AF288244	-	-	-	-
Draco_dussumieri	-	-	-	-	-
Draco_haematopogon	AF288259	-	-	-	-
Draco_lineatus	-	-	-	-	-
Draco_maculatus	AF288248	-	-	-	-
Draco_maximus	AF288231	-	-	-	-
Draco_melanopogon	AF288258	-	-	-	-
Draco_obscurus	AF288250	-	-	-	-
Draco_quinquefasciatus	AF288232	-	-	-	-
Draco_taeniopterus	AF288251	-	-	-	-
Draco_volans	AF288267	-	-	AF443226	-
Ptyctolaemus_collicristatus	AY555837	-	-	-	-
Ptyctolaemus_gularis	**MW111456**	-	-	-	-
Calotes_calotes	AF128482	AY662584	-	-	AB263941
Calotes_ceylonensis	AF128483	-	-	-	-
Calotes_emma	DQ289460	JF806189	JF804581	-	-
Calotes_htunwini	DQ289464	-	-	-	-
Calotes_jerdoni	GQ502783	-	-	-	-
Calotes_medogensis	**MW111455**	-	-	-	-
Calotes_mystaceus	AF128487	-	-	-	-
Calotes_versicolor	DQ289478	-	-	NC_009683	AY572870
Calotes_chincollium	DQ289459	-	-	-	-
Calotes_irawadi	DQ289468	-	-	-	-
Calotes_minor	KT952396	KT952398	-	-	-

Japalura_andersoniana	MK001394	-	MK001445	-	-
Japalura_kumaonensis	MK001396	-	MK001479	-	-
Japalura_tricarinata	AF128478	AY662585	MK001480	-	-
Japalura_variegata	AF128479	-	-	-	-
Pseudocalotes_bapoensis	MK001436	-	MK001453	-	-
Cristidorsa_otai	MK0014101	-	MK001441	-	-
Cristidorsa_planidorsata	MK001400	-	MK001442	-	-
Diploderma_batangensis	MK001412	-	-	-	-
Diploderma_brevipes	MK001429	-	MK001464	-	-
Diploderma_chapaensis	MG214262	-	MK001474	-	-
Diploderma_dymondi	MK001422	-	MK001477	-	-
Diploderma_flaviceps	MK001416	-	MK001472	-	-
Diploderma_iadinum	**MW111458**	-	-	-	-
Diploderma_laeviventris	MK001407	-	MK001466	-	-
Diploderma_luei	MK001433	-	MK001462	-	-
Diploderma_makii	MK001431	-	MK001460	-	-
Diploderma_micangshanensis	MK001424	-	MK001452	-	-
Diploderma_polygonata	MK001427	-	MK001458	-	-
Diploderma_slowinskii	MK001405	-	-	-	-
Diploderma_splendidum	MK001418	-	MK001476	-	-
Diploderma_swinhonis	MK001419	-	MK001456	-	-
Diploderma_varcoae	**MW111459**	-	-	-	-
Diploderma_vela	MK001414	-	MK001448	-	-
Diploderma_yulongense	MK001410	-	MK001450	-	-
Diploderma_yunnanense	MK001409	-	MK001469	-	-
Diploderma_zhaoermii	MK001425	-	MK001467	-	-
Mictopholis_austeniana	**MW111457**	-	-	-	-
Pseudocalotes_brevipes	AF128502	MG599031	-	KY884011	AY572869
Sarada_deccanensis	KT831315	KT831296	KT831279	-	-
Sitana_sivalensis	MG641403	MG641352	MG641310	-	-
Sitana_ponticeriana	KT831326	KT831304	KT831286	-	-
Sitana_spinaecephalus	KT831336	KT831309	KT831290	-	-
Sitana_laticeps	KT831323	KT831302	KT831284	-	-
Sitana_visiri	KT831340	KT831312	KT831293	-	-
Sitana_marudhamneydhal	KX371915	MG641332	KX371918	-	-
Otocryptis_beddomei	MG650165	MG641363	MG641324	-	-
Acanthosaura_armata	NC_014175	-	-	NC_014175	AY572872
Acanthosaura_lepidogaster	AF128499	JF806187	JF804578	-	AY572923
Salea_horsfieldii	AF128490	MG599032	-	-	-
Mantheyus_phuwuanensis	AY555836	FJ356735	-	-	-
Ceratophora_aspera	AF128491	-	-	-	-
Lyriocephalus_scutatus	AF364052	MG599029	-	-	-
Aphaniotis_fusca	AF288228	-	-	-	-
Gonocephalus_grandis	AF128496	-	-	-	AB263939

Bronchocela_cristatella	AF288229	-	-	-	-
Hydrosaurus_amboinensis	NC_014178	JF806195	JF804591	NC_014178	NC_014178
Leiolepis_belliana	U82689	AY662587	HQ876324	-	-
Leiolepis_guttata	NC_014179	-	-	NC_014179	NC_014179
Uromastyx_acanthinura	AB113801	AY988025	-	-	AB474753
Uromastyx_aegyptia	AB113806	-	-	AF443224	AB116942
Saara_hardwickii	AB113803	GU457972	HQ876325	-	AB474757
**Outgroup**					
Brookesia_brygooi	FJ975191	JF806188	JF804580	FJ981800	-
Chamaeleo_calyptratus	AF448744	GU457970	HQ876323	HF570566	-
Phrynosoma_cornutum	DQ385344	FJ356738	-	U66224	AY141087
Phrynosoma_platyrhinos	DQ385354	DQ385419	JF804597	JN809350	EU543746
Uma_scoparia	AF049861	JF806214	JF804608	-	EU543750
Uta_stansburiana	AF049863	DQ385422	JF804610	JX481607	JX481462
Petrosaurus_mearnsi	JN648436	GQ896005	HQ876333	AF210354	GQ272777
Sceloporus_graciosus	AF049860	EU085722	GQ464646	EU085839	KC853847
Crotaphytus_collaris	EU038620	FJ356749	JF804586	EU085839	KC853847
Gambelia_wislizenii	NC_012831	AY662600	HQ876327	KC621497	EU116511
Brachylophus_fasciatus	AF528721	AY988026	JF804579	U66238	KX610572
Dipsosaurus_dorsalis	AF049857	FJ356747	HQ876329	KX579001	KX610606
Sauromalus_ater	U82687	AY662591	JF804603	HM352518	KX610613
Polychrus_marmoratus	NC_012839	FJ356748	HQ876335	NC_012839	MH221669
Leiocephalus_barahonensis	EF591774	-	DQ119622	-	KU710319
Anolis_carolinensis	NC_010972	FJ356739	HQ876334	EU747728.2	EU747728.2
Basiliscus_vittatus	NC_012829	-	-	NC_012829	NC_012829
Corytophanes_cristatus	AF528717	JF806205	JF804585	-	-
Stenocercus_guentheri	DQ080223	HQ876440	HQ876337	-	-
Tropidurus_hispidus	AY625154	AY988013	-	-	KU245065
Uranoscodon_superciliosus	AF528749	JF806215	JF804609	-	KU245082
Enyalioides_laticeps	EU586748	EU586773	JF804590	U66226	-
Morunasaurus_annularis	EU586758	FJ356741	HQ876328	-	-
Liolaemus_bellii	AF099223	HQ876436	HQ876331	-	MH178577
Phymaturus_indistinctus	AY661893	-	-	AY367851.2	MH923556
Chalarodon_madagascariensis	AB266748	FJ356745	JF804582	NC_012836	NC_012836
Oplurus_cuvieri	U82685	AY662601	-	U66225	KY942063
Urostrophus_vautieri	AF528734	HQ876435	HQ876330	KM517830	KT342907
Leiosaurus_catamarcensis	AF528731	JF806207	JF804594	-	KT342892
Pristidactylus_torquatus	-	JF806210	JF804601	-	KT342906
**Species**	**12s-16s**	**C-mos**	**BDNF**		
Amphibolurus_muricatus	-	AF137523	DQ340701		
Chelosania_brunnea	-	DQ340664	JF806006		
Ctenophorus_adelaidensis	-	DQ340692	JF806008		
Ctenophorus_isolepis	-	DQ340671	DQ340715		
Diporiphora_nobbi	-	DQ340661	DQ340702		

Diporiphora_winneckei	-	DQ340680	DQ340725
Intellagama_lesueurii	AB031974-AB031991	DQ340689	DQ340737
Moloch_horridus	-	DQ340697	DQ340746
Physignathus_cocincinus	AF236822-AB031990	AY987991	HQ876215
Pogona_vitticeps	AB166795-AB031992	DQ340691	DQ340739
Rankinia_diemensis	-	DQ340693	DQ340741
Tympanocryptis_lineata	-	DQ340694	DQ340742
Tympanocryptis_tetraporophora	EF081041	DQ340695	DQ340743
Chlamydosaurus_kingii	EF090423-EF090423	DQ340665	DQ340708
Hypsilurus_boydii	-	DQ340682	DQ340727
Gowidon_temporalis	AB031970-AB031987	DQ340662	DQ340703
Acanthocercus_atricollis	JX668130	JX838888	-
Agama_agama	GU133310	AF137530	EU402615
Agama_anchietae	AF355524	JX838905	-
Xenagama_taylori	GU128466	JX838994	-
Trapelus_agilis	HQ901109	-	JF806016
Trapelus_mutabilis	GU128465	-	-
Pseudotrapelus_sinaitus	NC_013603	-	-
Bufoniceps_laungwalaensis	-	-	-
Coryphophylax_subcristatus	EU502966-EU503021	-	-
Stellagama_stellio	GU952115-GU128464	-	-
Paralaudakia_caucasia	AY053643-AY053765	DQ340686	DQ340732
Paralaudakia_himalayana	-	-	KJ363402
Paralaudakia_lehmanni	-	-	-
Laudakia_wui	-	-	-
Laudakia_tuberculata	HM040916-HM921190	-	-
Laudakia_nupta	HQ901100	-	-
Laudakia_melanura	-	-	-
Laudakia_sacra	-	-	-
Laudakia_papenfussi	-	-	-
Phrynocephalus_arabicus	-	-	KJ363434
Phrynocephalus_axillaris	AY053656-AY053779	-	KJ363410
Phrynocephalus_erythrurus	-	-	KJ363406
Phrynocephalus_forsythii	AY053659-	-	KJ363405

	AY053784		
Phrynocephalus_frontalis	-	-	KJ363409
Phrynocephalus_guinanensis	-	-	-
Phrynocephalus_guttatus	AY053678-AY053800	-	KJ363414
Phrynocephalus_helioscopus	AY053682-AY053817	-	KJ363426
Phrynocephalus_interscapularis	-	-	KJ363436
Phrynocephalus_maculatus	-	-	KJ363433
Phrynocephalus_mystaceus	AY053700-AY053822	AF137527	DQ340735
Phrynocephalus_reticulatus	-	-	KJ363428
Phrynocephalus_ornatus	-	-	KJ363435
Phrynocephalus_persicus	-	-	KJ363424
Phrynocephalus_przewalskii	AY053702-AY053828	-	KJ363419
Phrynocephalus_putjatai	-	-	KJ363408
Phrynocephalus_raddei	-	-	KJ363429
Phrynocephalus_rossikowi	-	-	-
Phrynocephalus_scutellatus	-	-	-
Phrynocephalus_strauchi	-	-	KJ363430
Phrynocephalus_theobaldi	-	-	-
Phrynocephalus_versicolor	-	-	KJ363421
Phrynocephalus_vlangalii	AY053749-AY053880	-	AF497714
Draco_biaro	-	-	-
Draco_bimaculatus	-	-	-
Draco_blanfordii	AB023733-AB023751	-	JF806010
Draco_cornutus	AB023729-AB023752	-	-
Draco_dussumieri	AB023734-AB023753	-	-
Draco_haematopogon	AB023736-AB023755	-	-
Draco_lineatus	AB023738-AB023757	-	-
Draco_maculatus	AB023739-AB023758	-	-
Draco_maximus	AB023740-AB023760	-	-
Draco_melanopogon	AB023742-AB023762	-	-
Draco_obscurus	AB023744-	-	-

	AB023764		
Draco_quinquefasciatus	AB023745-<br>AB023766	-	-
Draco_taeniopterus	AB023747-<br>AB023767	-	-
Draco_volans	AB023731-<br>AB023770	-	-
Ptyctolaemus_collicristatus	-	-	-
Ptyctolaemus_gularis	-	-	-
Calotes_calotes	-	-	-
Calotes_ceylonensis	-	-	-
Calotes_emma	-	-	JF806005
Calotes_htunwini	-	-	-
Calotes_jerdoni	-	-	-
Calotes_medogensis	-	-	-
Calotes_mystaceus	-	-	-
Calotes_versicolor	AB183287-<br>AB031981	AF137525	DQ340705
Calotes_chincollium	-	-	-
Calotes_irawadi	-	-	-
Calotes_minor	-	-	-
Japalura_andersoniana	-	MK001486	MK001556
Japalura_kumaonensis	-	-	-
Japalura_tricarinata	-	MK001491	MK001560
Japalura_variegata	-	-	-
Pseudocalotes_bapoensis	-	MK001503	MK001550
Cristidorsa_otai	-	-	-
Cristidorsa_planidorsata	-	-	-
Diploderma_batangensis	-	-	-
Diploderma_brevipes	-	MK001506	MK001540
Diploderma_chapaensis	-	MK001494	MK001526
Diploderma_dymondi	-	MK001513	MK001545
Diploderma_flaviceps	-	MK001512	MK001528
Diploderma_iadinum	-	-	-
Diploderma_laeviventris	-	-	MK001529
Diploderma_luei	-	MK001510	MK001532
Diploderma_makii	-	MK001508	MK001536
Diploderma_micangshanensis	-	-	MK001559
Diploderma_polygonata	-	MK001515	MK001538
Diploderma_slowinskii	-	-	-
Diploderma_splendidum	-	MK001492	MK001522
Diploderma_swinhonis	-	MK001495	MK001534
Diploderma_varcoae	-	MK001493	MK001523
Diploderma_vela	-	MK001499	MK001530

Diploderma_yulongense	-	MK001498	MK001542
Diploderma_yunnanense	-	MK001517	MK001524
Diploderma_zhaoermii	-	MK001500	MK001547
Mictopholis_austeniana	-	-	-
Pseudocalotes_brevipes	-	-	-
Sarada_deccanensis	-	-	-
Sitana_sivalensis	-	-	-
Sitana_ponticeriana	-	-	-
Sitana_spinaecephalus	-	-	-
Sitana_laticeps	-	-	-
Sitana_visiri	-	-	-
Sitana_marudhamneydhal	-	-	-
Otocryptis_beddomei	-	-	-
Acanthosaura_armata	NC_014175	-	-
Acanthosaura_lepidogaster	-	-	JF806003
Salea_horsfieldii	-	-	-
Mantheyus_phuwuanensis	AB023750-AB023772	-	-
Ceratophora_aspera	-	-	-
Lyriocephalus_scutatus	-	-	-
Aphaniotis_fusca	AB023749-AB023771	-	-
Gonocephalus_grandis	AB031966-AB031983	-	-
Bronchocela_cristatella	EU503024	-	-
Hydrosaurus_amboinensis	NC_014178	-	JF806011
Leiolepis_belliana	AB031969-AB031986	FJ984253	HQ876216
Leiolepis_guttata	NC_014179	-	-
Uromastyx_acanthinura	FJ639598	AY987992	AY987979
Uromastyx_aegyptia	AB031977-AB031994	AF137531	-
Saara_hardwickii	FJ639591	-	GU457849
**Outgroup**			
Brookesia_brygooi	HF570400	FJ984306	JF806004
Chamaeleo_calyptratus	HF570443	HF570667	GU457847
Phrynosoma_cornutum	DQ385390	AY987989	KR360458
Phrynosoma_platyrhinos	DQ385401	-	DQ385330
Uma_scoparia	AF194260	-	JF806029
Uta_stansburiana	GQ464578	AF315389	JN648395
Petrosaurus_mearnsi	L41450	-	HQ876221
Sceloporus_graciosus	AF440090	-	EU085928
Crotaphytus_collaris	L41443	AY987985	JF806021
Gambelia_wislizenii	AY217995	AY217842	KP820845

Brachylophus_fasciatus	-	AY987993	JF806018
Dipsosaurus_dorsalis	KC621331	AF148705	GQ853275
Sauromalus_ater	-	HM352537	JF806026
Polychrus_marmoratus	AF338329	AY987983	AY987966
Leiocephalus_barahonensis	U39564.2	DQ119594	HQ876223
Anolis_carolinensis	EU747728.2	-	EU402616
Basiliscus_vittatus	NC_012829	-	-
Corytophanes_cristatus	-	AF315390	JF806020
Stenocercus_guentheri	L41481	-	HQ876224
Tropidurus_hispidus	U39563	AY987984	AY987967
Uranoscodon_superciliosus	AF362543	-	JF806030
Enyalioides_laticeps	KY982512	-	GU457848
Morunasaurus_annularis	-	-	HQ876218
Liolaemus_bellii	AY662069	-	HQ876220
Phymaturus_indistinctus	AY367823	AY367880	-
Chalarodon_madagascariensis	NC_012836	AY987987	JF806019
Oplurus_cuvieri	U39587	EU099681	AY987971
Urostrophus_vautieri	KM517592	-	HQ876219
Leiosaurus_catamarcensis	AF338341	-	JF806022
Pristidactylus_torquatus	KT342933	-	JF806025

Elapidae

Species	Cytb	ND4	12s	16s	C-mos
Acanthophis_praelongus	AY340135	AY340164	EU547112	EU547161	EU546926
Aipysurus_laevis	DQ233922	EF506638	EU547132	DQ233997	EU546945
Aspidomorphus_muelleri	AF217814	EU546999	EU547090	EU547139	EU366448
Austrelaps_superbus	AF217815	EU547030	EU547127	EU547176	EU546940
Brachyurophis_semifasciatus	EU547058	EU547012	EU547107	EU547156	EU546922
Cacophis_squamulosus	EU547052	EU547007	EU547101	EU547150	EU366451
Demansia_papuensis	EU547044	EU547002	EU547093	EU547142	EU546910
Denisonia_devisi	EU547071	EU547023	EU547120	EU547169	EU546933
Drysdalia_coronoides	EU547075	EU547027	EU547124	EU547173	EU546937
Echiopsis_curta	EU547072	EU547024	EU547121	EU547170	EU546934
Elapognathus_coronatus	EU547069	EU547021	EU547118	EU547167	EU546931
Emydocephalus_annulatus	EU547087	EU547038	EU547136	EU547185	EU546947
Ephalophis_greyae	KC014392	KC014466	-	KC014318	-
Furina_diadema	EU547053	EU547008	EU547102	EU547151	EU546917
Hemiaspis_dameli	DQ233952	EU547025	EU547122	EU547171	EU546935
Hoplocephalus_bitorquatus	EU547079	EU547031	EU547128	EU547177	EU546941
Hydrelaps_darwiniensis	EU547084	EU547035	EU547133	EU547182	EU546946
Hydrophis_cyanocinctus	DQ233946	FJ593215	-	DQ234032	FJ587189
Micropechis_ikaheka	AF217826	EU547000	EU547091	EU547140	EU366449
Neelaps_calonotus	EU547060	EF210841	EU547109	EU547158	EU546923
Notechis_scutatus	AF217836	EU547034	U96802	-	EU546944
Oxyuranus_microlepidotus	EU547050	EF210823	EU547099	EU547148	EU366450
Paroplocephalus_atriceps	EU547080	EU547032	EU547129	EU547178	EU546942
Parahydrophis_mertoni	DQ233974	FJ593201	-	DQ234048	FJ587177
Parasuta_monachus	EU547067	EU547019	EU547116	EU547165	EU546929
Pseudechis_australis	AY343091	AY340177	AJ749363	EU547144	EU546912
Pseudonaja_textilis	EU547048	DQ098645	EU547097	EU547146	EU546914
Rhinoplocephalus_bicolor	EU547068	EU547020	EU547117	EU547166	EU546930
Simoselaps_anomalus	EU547061	EU547014	EU547110	EU547159	EU546924
Suta_suta	EU547066	EU547018	EU547115	EU547164	EU366452
Toxicocalamus_preussi	AF217825	EU547001	EU547092	EU547141	EU546909
Tropidechis_carinatus	EU547081	EU547033	EU547130	EU547179	EU546943
Vermicella_intermedia	EU547055	EF210842	EU547104	EU547153	EU546919
Aspidelaps_scutatus	KX694861	-	KX694563	KX694617	KX694796
Bungarus_bungaroides	AJ748689	AJ830218	-	-	-
Bungarus_caeruleus	AJ749305	AJ830220	-	-	-
Bungarus_candidus	AJ749343	AJ830255	JN687932	JN687933	-
Bungarus_ceylonicus	KC347466	KC347501	KC347316	KC347350	KC347390
Bungarus_fasciatus	EU547086	EU547037	EU547135	EU547184	EU366447
Bungarus_flaviceps	AJ749351	AJ830251	-	-	-
Bungarus_multicinctus	AJ749345	NC 011392	EU579522	EF520682	AF435021

Bungarus_niger	AJ749304	AJ830241	-	-	-
Bungarus_sindanus	AJ749346	AJ830242	-	-	-
Bungarus_slowinskii	AJ749306	AJ830250	-	-	-
Calliophis_melanurus	KC347458	KC347502	KC347317	KC347351	KC347391
Calliophis_bivirgata	AF217812	AY058979	U96800	-	AY058934
Calliophis_bibroni	KX573695	-	-	-	-
Calliophis_castoe	KX573696	JQ282155	-	-	-
Calliophis_intestinalis	-	KX130761	-	-	-
Calliophis_nigrescens	-	JQ282156	-	-	-
Cryptophis_nigrescens	EU547070	EU547022	EU547119	EU547168	EU546932
Dendroaspis_angusticeps	JF357936	JF357927	AF544764	JF357945	AF544735
Elapsoidea_semiannulata	AF039260	JF357928	AF544745	JF357946	AF544678
Hemachatus_haemachatus	AF217821	-	U96797	-	-
Hemibungarus_calligaster	EF137411	EF137403	-	-	EF137419
Laticauda_colubrina	AF217834	EU546998	EU547089	EU547138	AY058932
Micruroides_euryxanthus	AF217823	-	Z46433	Z46483	-
Micrurus_surinamensis	EF137415	EF137407	AF544770	AF544799	AF544708
Naja_kaouthia	**MW111477**	**MW111432**	**MW020355**	**MW019917**	AY058938
Naja_atra	**MW111478**	**MW111433**	**MW020356**	**MW019918**	-
Naja_annulata	AY188010	AY058970	-	AY188049	AY187971
Naja_siamensis	AB920242	-	JN687926	JN687927	-
Naja_sumatrana	AB920240	-	JN687928	JN687929	-
Naja_naja	DQ272477	DQ897690	NC_010225	GQ359756	AF435020
Naja_haje	AY611994	GQ359585	GQ359666	AY611811	AY611903
Naja_annulifera	AF155216	GQ359586	GQ359667	GQ359753	-
Naja_nivea	AF217827	AY058983	EU624238	GQ359755	AY058939
Naja_ashei	GQ359493	GQ359575	GQ359656	GQ359742	-
Naja_mossambica	GQ359495	GQ359577	GQ359658	GQ359744	-
Naja_nigricollis	AF399746	DQ897697	EU624237	GQ359754	-
Naja_katiensis	GQ359494	GQ359576	GQ359657	GQ359743	-
Naja_pallida	AF399750	DQ897714	GQ359659	GQ359745	-
Naja_nubiae	AF399752	DQ897718	GQ359660	GQ359746	-
Naja_melanoleuca	-	JF357931	U96801	JF357949	-
Naja_multifasciata	AF217837	AY058985	-	-	AY058941
Ophiophagus_hannah	EF694840	NC 011394	EU921899	NC_011394	AY058940
Sinomicrurus_japonicus	AF217831	AY058971	D31615	-	AY058926
Sinomicrurus_kelloggii	EF137417	EF137409	-	-	EF137424
Sinomicrurus_mcclellandii	EF137418	EF137410	D31616	-	EF137425
Walterinnesia_aegypta	AF217838	AY058988	U96807	-	AY058943
**Outgroup**					
Eryx_miliaris	U69824	AF302942	AF544746	-	AF544683
Boa_constrictor	AB177354	AB177354	AB177354	AB177354	AF471115
Eunectes_notaeus	AM236347	AM236347	AM236347	AM236347	-
Psammophis_lineatus	DQ486428	EU526861	FJ404152	FJ404216	FJ404256

Compsophis_albiventris	AY188011	FJ404351	FJ404149	AY188050	AY187972
Sistrurus_catenatus	AY223610	AF156575	HQ257513	AF259119	JN090134
Crotalus_atrox	AY223608	DQ679856	AF259258	AF057272	JN090135
Crotalus_viridis	AF471066	AF194157	DQ020029	AF259145	AF471135
Vipera_ursinii	-	EF012798	EF012817	-	AF433658
Bitis_arietans	AY235728	EU852304	EU852316	JF357953	-
Trimeresurus_trigonocephalus	KC347488	AY059597	KC347336	KC347374	-
Pareas_carinatus	JQ598940	-	AF544773	AF544802	AF544692
Achalinus_meiguensis	FJ424614	FJ424614	FJ424614	FJ424614	-
Acrochordus_granulatus	AB177879	AB177879	AB177879	AB177879	AF471124
Acrochordus_javanicus	-	HM234055	AF512745	Z46502	HM234058
Anilius_scytale	NC_014343	NC_014343	NC_014343	NC_014343	AF544722
Cylindrophis_maculatus	-	-	KC347320	KC347355	-
Cylindrophis_ruffus	AB179619	AB179619	AB179619	AB179619	AF471133
Cantoria_violacea	EF395897	-	EF395873	EF395848	EF395922
Cerberus_rynchops	EF395901	U49327	AF499289	EF395852	EF395926
Loxocemus_bicolor	AY099993	-	AF512737	AF512737	AY099969
Broghammerus_reticulatus	U69860	-	Z46448	EF545062	AF039472
Xenopeltis_unicolor	NC_007402	NC_007402	NC_007402	NC_007402	DQ465561
Indotyphlops_braminus	NC_010196	NC_010196	NC_010196	NC_010196	AY099980
Lycodon_osmanhilli	-	KC347524	-	KC347364	KC347403
Oligodon_arnensis	KC347464	KC347504	KC347327	KC347365	KC347404
Rhabdophis_subminiatus	JQ598951	JQ687411	AF544776	AF544805	JQ687436
Thamnophis_sirtalis	L33290	AY136243	AF402646	KX694670	DQ902094
Thermophis_zhaoermii	GQ166168	GQ166168	GQ166168	GQ166168	KP777529
Oxyrhabdium_leporinum	MG458755	-	-	-	MG458765
Homoroselaps_lacteus	AF217833	FJ404339	FJ404135	AY611843	AY058931

**Species**	**RAG1**
Acanthophis_praelongus	EU546887
Aipysurus_laevis	EU546906
Aspidomorphus_muelleri	EU366434
Austrelaps_superbus	EU546901
Brachyurophis_semifasciatus	EU546883
Cacophis_squamulosus	EU366440
Demansia_papuensis	EU546871
Denisonia_devisi	EU546894
Drysdalia_coronoides	EU546898
Echiopsis_curta	EU546895
Elapognathus_coronatus	EU546892
Emydocephalus_annulatus	EU546908
Ephalophis_greyae	-
Furina_diadema	EU546878
Hemiaspis_dameli	EU546896
Hoplocephalus_bitorquatus	EU546902

Hydrelaps_darwiniensis	EU546907
Hydrophis_cyanocinctus	FJ587112
Micropechis_ikaheka	EU366435
Neelaps_calonotus	EU546884
Notechis_scutatus	-
Oxyuranus_microlepidotus	EU366439
Paroplocephalus_atriceps	EU546903
Parahydrophis_mertoni	FJ587099
Parasuta_monachus	EU546890
Pseudechis_australis	EU546873
Pseudonaja_textilis	EU546875
Rhinoplocephalus_bicolor	EU546891
Simoselaps_anomalus	EU546885
Suta_suta	EU366436
Toxicocalamus_preussi	EU546870
Tropidechis_carinatus	EU546904
Vermicella_intermedia	EU546880
Aspidelaps_scutatus	-
Bungarus_bungaroides	-
Bungarus_caeruleus	-
Bungarus_candidus	-
Bungarus_ceylonicus	-
Bungarus_fasciatus	EU366438
Bungarus_flaviceps	-
Bungarus_multicinctus	-
Bungarus_niger	-
Bungarus_sindanus	-
Bungarus_slowinskii	-
Calliophis_melanurus	-
Calliophis_bivirgata	-
Calliophis_bibroni	-
Calliophis_castoe	-
Calliophis_intestinalis	-
Calliophis_nigrescens	-
Cryptophis_nigrescens	EU546893
Dendroaspis_angusticeps	-
Elapsoidea_semiannulata	-
Hemachatus_haemachatus	-
Hemibungarus_calligaster	-
Laticauda_colubrina	EU366433
Micruroides_euryxanthus	-
Micrurus_surinamensis	-
Naja_kaouthia	-
Naja_atra	-

Naja_annulata	-
Naja_siamensis	-
Naja_sumatrana	-
Naja_naja	EU366432
Naja_haje	-
Naja_annulifera	-
Naja_nivea	-
Naja_ashei	-
Naja_mossambica	-
Naja_nigricollis	-
Naja_katiensis	-
Naja_pallida	-
Naja_nubiae	-
Naja_melanoleuca	-
Naja_multifasciata	-
Ophiophagus_hannah	-
Sinomicrurus_japonicus	-
Sinomicrurus_kelloggii	-
Sinomicrurus_mcclellandii	-
Walterinnesia_aegypta	-
**Outgroup**	
Eryx_miliaris	AY487393
Boa_constrictor	AY487351
Eunectes_notaeus	AY988063
Psammophis_lineatus	-
Compsophis_albiventris	-
Sistrurus_catenatus	-
Crotalus_atrox	-
Crotalus_viridis	-
Vipera_ursinii	-
Bitis_arietans	EU852328
Trimeresurus_trigonocephalus	-
Pareas_carinatus	-
Achalinus_meiguensis	-
Acrochordus_granulatus	HM234060
Acrochordus_javanicus	HM234061
Anilius_scytale	AY988072
Cylindrophis_maculatus	KC347433
Cylindrophis_ruffus	AY662613
Cantoria_violacea	-
Cerberus_rynchops	EU366441
Loxocemus_bicolor	AY444061
Broghammerus_reticulatus	EU624119
Xenopeltis_unicolor	EU402870

Indotyphlops_braminus	AY662612
Lycodon_osmanhilli	KC347441
Oligodon_arnensis	KC347442
Rhabdophis_subminiatus	-
Thamnophis_sirtalis	-
Thermophis_zhaoermii	-
Oxyrhabdium_leporinum	-
Homoroselaps_lacteus	-

Anguidae

Species	COI	Cytb	ND2	ND4	AKAP9
Abronia_graminea	AB080273	AB080273	AB080273	AB080273	KY712872
Anguis_fragilis	EU443256	EU443256	EU443256	EU443256	KY712869
Barisia_imbricata	-	AF056597	AF085613	AY605116	-
Dopasia_gracilis	**MW021342**	NC030369	NC030369	NC030369	-
Dopasia_hainanensis	**MW021343**	-	-	-	-
Dopasia_harti	NC022700	KY712783	AF085624	KY712810	KY712870
Dopasia_sp	**MW021344**	-	-	-	-
Elgaria_kingii	-	KY712790	KY712769	KY712818	KY712879
Gerrhonotus_parvus	-	KY712787	KY712768	KY712814	KY712875
Hyalosaurus_koellikeri	-	-	AF085621	-	-
Mesaspis_monticola	MH274440	-	-	MG598733	-
Ophisaurus_attenuatus	EU747729	EU747729	EU747729	EU747729	-
Ophisaurus_ventralis	KU986014	KY712784	KY712766	KY712811	KY712871
Pseudopus_apodus	-	KY712782	AF085623	KY712808	JN614168
**Outgroup**					
Anniella_pulchra	KU985941	AF195090	AF085606	AY620747	JN614136
Celestus_enneagrammus	-	-	AF085607	MG598720	JN614144
Diploglossus_pleei	-	-	AF085609	-	-
Ophiodes_striatus	-	-	AF085610	-	-
**Species**	**ATPSb**	**BACH1**	**EXPH5**	**NKTR**	**SELT**
Abronia_graminea	KY712923	KY712981	KY713030	KY713062	KY713114
Anguis_fragilis	KY712920	KY712978	KY713027	KY713059	-
Barisia_imbricata	-	-	-	-	-
Dopasia_gracilis	-	-	-	-	-
Dopasia_hainanensis	-	-	-	-	-
Dopasia_harti	KY712921	KY712979	KY713028	KY713060	KY713112
Dopasia_sp	-	-	-	-	-
Elgaria_kingii	KY712930	KY712988	KY713035	KY713069	KY713122
Gerrhonotus_parvus	KY712926	KY712984	KY713032	KY713065	KY713117
Hyalosaurus_koellikeri	-	-	-	-	-
Mesaspis_monticola	-	-	-	-	-
Ophisaurus_attenuatus	-	-	-	-	-
Ophisaurus_ventralis	KY712922	KY712980	KY713029	KY713061	KY713113
Pseudopus_apodus	KY712919	JN614195	KY713026	KY713058	KY713111
**Outgroup**					
Anniella_pulchra	-	-	-	JN614006	-
Celestus_enneagrammus	-	JN614186	-	JN614010	-
Diploglossus_pleei	-	-	-	-	-
Ophiodes_striatus	-	-	-	-	-
**Species**	**UBN1**				
Abronia_graminea	KY713180				

Anguis_fragilis	KY713177
Barisia_imbricata	-
Dopasia_gracilis	-
Dopasia_hainanensis	-
Dopasia_harti	KY713178
Dopasia_sp	-
Elgaria_kingii	KY713187
Gerrhonotus_parvus	KY713183
Hyalosaurus_koellikeri	-
Mesaspis_monticola	-
Ophisaurus_attenuatus	-
Ophisaurus_ventralis	KY713179
Pseudopus_apodus	JN614070
**Outgroup**	
Anniella_pulchra	JN614047
Celestus_enneagrammus	JN614054
Diploglossus_pleei	-
Ophiodes_striatus	-

Pareidae

Species	Cytb	C-mos	ND4
Pareas_atayal	KJ642116	KJ642198	-
Pareas_carinatus	JF827676	JF827701	JF827652
Pareas_chinensis	**MW111480**	**MW111509**	-
Pareas_chinensis	**MW111481**	**MW111510**	-
Pareas_chinensis	JF827690	JF827716	JF827667
Pareas_nuchalis	-	-	U49311
Pareas_formosensis	KJ642130	KJ642201	-
Pareas_monticola	JF827689	JF827715	JF827666
Pareas_monticola	**MW111482**	**MW111511**	-
Pareas_hamptoni	KJ642153	KJ642205	-
Pareas_iwasakii	KJ642156	KJ642207	-
Pareas_komaii	KJ642182	KJ642210	-
Pareas_macularius	**MW111483**	**MW111512**	-
Pareas_margaritophorus	**MW111484**	**MW111513**	-
Pareas_stanleyi	JN230704	JN230703	JN230705
Aplopeltura_boa	JF827673	JF827696	JF827650
Asthenodipsas_tropidonotus	AY425808	-	-
Asthenodipsas_vertebralis	AY425807	-	-
Asthenodipsas_malaccanus	KX660469	KX660336	KX660597
Asthenodipsas_laevis	KX660468	KX660335	KX660596
**Outgroup**			
Gloydius_blomhoffi	JF827674	JF827697	JF827651
Lycodon_rufozonatum	JF827672	JF827695	JF827649
Eryx_miliaris	U69824	AF544683	AF302942
Boa_constrictor	AB177354	AF471115	AB177354
Psammophis_lineatus	DQ486428	FJ404256	EU526861
Compsophis_albiventris	AY188011	AY187972	FJ404351
Sistrurus_catenatus	AY223610	JN090134	AF156575
Crotalus_atrox	AY223608	JN090135	DQ679856
Achalinus_meiguensis	FJ424614	-	FJ424614
Acrochordus_granulatus	AB177879	AF471124	AB177879
Acrochordus_javanicus	-	HM234058	HM234055
Anilius_scytale	NC_014343	AF544722	NC_014343
Cylindrophis_ruffus	AB179619	AF471133	AB179619
Cantoria_violacea	EF395897	EF395922	-
Cerberus_rynchops	EF395901	EF395926	U49327
Loxocemus_bicolor	AY099993	AY099969	-
Broghammerus_reticulatus	U69860	AF039472	-
Xenopeltis_unicolor	NC_007402	DQ465561	NC_007402
Indotyphlops_braminus	NC_010196	AY099980	NC_010196
Thamnophis_sirtalis	L33290	DQ902094	AY136243

Thermophis_zhaoermii	GQ166168	KP777529	GQ166168
Naja_naja	DQ272477	AF435020	DQ897690
Naja_haje	AY611994	AY611903	GQ359585

Scincidae

**Species**	**12s**	**16s**	**ND2**	**ND4**	**Cytb**
Ablepharus_pannonicus	-	-	AY607280	-	-
Acontias_meleagris	AY169563	HQ180038	AY662553	AY169637	FJ972224
Amphiglossus_reticulatus	AY315490	AY315539	-	-	AY391172
Anomalopus_swansoni	AY169576	AY169613	-	AY169651	-
Asymblepharus_alaicus	-	-	AY607281	-	-
Asymblepharus_nyingchiensis	-	-	**MW111452**	-	-
Asymblepharus_sp	-	-	**MW111453**	-	-
Asymblepharus_sikimmensis	-	-	AY662549	-	-
Asymblepharus_medogensis	-	-	**MW111454**	-	-
Brachymeles_bicolor	AY818761	-	-	-	FJ972227
Brachymeles_bonitae	-	-	JN981990	-	-
Brachymeles_gracilis	AY649103	AY649143	-	-	-
Brachymeles_miriamae	-	-	HQ907436	-	-
Brachymeles_talinis	AY649104	AY649144	-	-	-
Calyptotis_scutirostrum	AY169577	AY308181	AF373265	AY169652	AF373235
Carlia_fusca	AY308331	AY308182	-	FJ379453	-
Chalcides_ocellatus	FJ980091	AY649147	AY662557	-	FJ972228
Chalcides_sexlineatus	AF054532	AF054547	-	-	AF054561
Chalcides_viridanus	AF232630	EU278036	-	-	AF054566
Ctenotus_labillardieri	-	-	-	EU109213	GQ241602
Dasia_grisea	AB028773	AB028784	-	JF498460	-
Dasia_olivacea	AB028772	AB028783	-	-	-
Dasia_vittata	AB028771	AB028782	-	-	-
Egernia_stokesii	-	-	-	JF813095	-
Emoia_atrocostata	JF497856	JF497979	-	JF498461	-
Emoia_caeruleocauda	AY218012	AY217962	JF498109	JF498462	U20454
Emoia_cyanura	AY218018	AY308191	DQ675263	JF498464	U49345
Eugongylus_rufescens	AY169567	AY649152	DQ675253	AY169642	AY217807
Eulamprus_kosciuskoi	DQ915292	DQ915316	-	DQ915340	-
Eulamprus_quoyii	AY169585	AY169622	-	AY169660	-
Eumeces_schneideri	AY315506	AY308213	-	-	-
Eurylepis_taeniolata	-	-	-	-	HM921206
Eutropis_allapallensis	JQ767975	JQ767959	-	-	-
Eutropis_beddomei	JQ767970	JQ767965	-	-	-
Eutropis_bibronii	JQ767979	JQ767963	-	-	-
Eutropis_carinata	JQ767968	JQ767954	-	-	-
Eutropis_clivicola	JQ767978	JQ767956	-	-	-
Eutropis_cumingi	DQ239218	DQ238896	-	-	DQ239137
Eutropis_dissimilis	AY159046	AY159075	-	-	-
Eutropis_indeprensa	AY159047	AY159076	-	-	-
Eutropis_longicaudata	AY070341	AY070359	-	AY169645	DQ239139

Eutropis_macrophthalma	AY159048	AY159077	-	-	-
Eutropis_macularia	AF153557	AF153573	-	AF228556	AF153590
Eutropis_madaraszi	AY159051	AY159080	-	-	-
Eutropis_multicarinata	AY159052	AY159081	-	-	-
Eutropis_multifasciata	AY159054	AF153576	JF498112	JF498466	AY151513
Eutropis_nagarjunensis	JQ767972	JQ767952	-	-	-
Eutropis_quadricarinata	AY159060	AY159089	-	-	-
Eutropis_rudis	AB028779	AB028790	-	-	DQ239135
Eutropis_rugifera	JQ767980	JQ767962	-	-	-
Eutropis_trivittata	JQ767971	JQ767951	-	-	-
Eutropis_tytleri	AY159045	AY159074	-	-	-
Feylinia_polylepis	AY649120	AY649161	AY662556	-	FJ972225
Hemiergis_peronii	HM852467	HM852495	-	AY169665	-
Heremites_auratus	-	-	U71330	-	AY151507
Heremites_vittatus	EU477288	-	-	-	EU443142
Insulasaurus_arborens	JF497863	JF497986	JF498113	JF498467	-
Insulasaurus_victoria	-	JF497989	JF498117	-	-
Insulasaurus_wrighti	JF497867	JF497990	JF498119	JF498471	-
Isopachys_anguinoides	AB028803	AB028815	-	-	-
Lamprolepis_smaragdina	AB028774	AY169605	-	AY169643	AY217803
Larutia_seribuatensis	-	-	HQ907429	-	-
Leptosiaphos_vigintiserierum	AY308409	AY308259	-	-	-
Lerista_neander	EF672796	EF672867	-	EF673008	-
Lipinia_noctua	JF497868	JF497992	JF498120	JF498473	-
Lipinia_pulchella	JF497869	JF497994	JF498121	JF498475	-
Lipinia_vittigera	AB028804	AB028816	-	-	-
Lygisaurus_novaeguineae	AY218014	AY217964	-	AJ290523	AY217810
Lygosoma_albopunctata	AY308414	AY308262	-	-	-
Lygosoma_bowringii	AB028775	AY308263	HQ907430	-	-
Lygosoma_koratense	AB028805	AY308269	-	-	-
Lygosoma_lineolatum	AY308422	AY308271	-	-	-
Lygosoma_punctata	-	-	DQ675265	-	-
Lygosoma_quadrupes	AB028806	AB028818	HQ907432	-	-
Mabuya_mabouya	EU477265	AY070357	JF498123	-	EU443115
Madascincus_melanopleura	AY315481	AY802764	-	-	AY802770
Mesoscincus_schwartzei	AY649117	AY649158	-	-	-
Nannoscincus_mariei	-	EU567924	EU423132	EU423130	EU567832
Oligosoma_aeneum	AF194088	EU567866	EF567164	EU567745	EU567771
Oligosoma_lichenigera	EU567966	EU567870	EU567704	EU567763	EU567829
Ophiomorus_punctatissimus	AY649127	AY649168	AY607279	-	EF581258
Otosaurus_cumingi	JF497873	JF497998	JF498126	JF498477	-
Papuascincus_stanleyanus	JF497877	JF498000	JF498128	JF498479	-
Parvoscincus_steerei	JF497904	JF498032	JF498160	JF498510	-
Parvoscincus_tagapayo	JF497918	JF498046	JF498174	JF498520	-

Plestiodon_barbouri	EU202957	DQ173534	-	-	EU203041
Plestiodon_capito	-	-	-	-	-
Plestiodon_chinensis	JN117724	-	-	-	-
Plestiodon_egregius	AB016606	AB016606	NC_000888	NC_000888	AB016606
Plestiodon_elegans	JN117723	AY649153	-	-	EU203036
Plestiodon_fasciatus	AY308349	AY308200	-	JF498546	DQ241639
Plestiodon_japonicus	EU202993	EU203034	-	-	EU203143
Plestiodon_kishinouyei	-	-	-	-	-
Plestiodon_laticeps	AY218039	AY308205	-	-	EU116510
Plestiodon_latiscutatus	AY308355	AY308206	-	-	EU203035
Plestiodon_marginatus	EU202955	EU202996	-	-	EU203038
Plestiodon_quadrilineatus	JF497945	JF498073	-	JF498547	-
Plestiodon_skiltonianus	AY308364	AY308215	AY662551	-	-
Plestiodon_stimpsonii	AB543279	EU202997	-	-	EU203039
Plestiodon_tamdaoensis	-	-	-	-	-
Plestiodon_tunganus	-	-	-	-	-
Ristella_rurkii	AY308443	AY308292	-	-	-
Scelotes_mirus	AY649130	AY028893	-	AF228558	AY217828
Scincella_assata	JF497946	JF498074	JF498186	JF498548	-
Scincella_cherriei	AB057377	AB057392	-	JF498550	GQ450315
Scincella_doriae	-	-	**MW111451**	-	-
Scincella_huanrenensis	-	-	KU507306	-	-
Scincella_lateralis	AY169598	HM852503	JF498187	AY169673	AY217806
Scincella_potanini	-	-	AY607287	-	-
Scincella_reevesii	JF497949	AY308299	-	JF498551	-
Scincella_rupicola	-	-	AY607284	-	-
Scincella_tsinlingensis	-	-	AY607286	-	-
Scincella_vandenburghi	-	-	KU646826	-	-
Scincus_mitranus	AY649133	AY308303	-	-	AF280131
Sphenomorphus_acutus	JF497950	JF498079	JF498188	JF498552	-
Sphenomorphus_buenloicus	-	HM773218	-	-	-
Sphenomorphus_concinnatus	JF497951	JF498080	JF498190	JF498554	-
Sphenomorphus_cranei	JF497952	JF498082	JF498192	JF498556	-
Sphenomorphus_cyanolaemus	JF497954	JF498084	JF498193	JF498557	-
Sphenomorphus_diwata	JF497956	JF498086	JF498194	JF498558	-
Sphenomorphus_fasciatus	JF497958	JF498088	DQ675240	JF498560	AF373234
Sphenomorphus_indicus	AB028808	AB028820	JF498198	JF498562	-
Sphenomorphus_jobiensis	DQ915291	AY308306	DQ675258	DQ915339	AF151648
Sphenomorphus_leptofasciatus	-	-	AF373263	-	AF373233
Sphenomorphus_maculatus	AY308461	AY308310	JF498199	JF498563	-
Sphenomorphus_maculatus	**MW026138**	**MW024860**	**MW111450**	-	-
Sphenomorphus_maindroni	AY308462	AY308311	-	-	-
Sphenomorphus_melanopogon	AY308463	AY308312	-	-	-
Sphenomorphus_muelleri	AY169599	AY169636	-	AY169674	-

Sphenomorphus_multisquamatus	JF497961	JF498091	JF498200	JF498564	-
Sphenomorphus_praesignis	AB028810	AB028822	-	-	-
Sphenomorphus_sabanus	AY308465	AY308314	JF498201	JF498565	-
Sphenomorphus_scutatus	JF497963	JF498093	JF498202	JF498566	-
Sphenomorphus_simus	AY218017	AY217967	-	-	EU116518
Sphenomorphus_solomonis	DQ915304	AY308316	JF498204	DQ915352	-
Sphenomorphus_stellatus	-	HM773221	-	-	-
Sphenomorphus_variegatus	JF497966	JF498096	JF498206	JF498569	-
Tiliqua_scincoides	AF090187	-	DQ675249	-	-
Trachylepis_perrotetii	DQ239223	AY159114	JF498207	AY169646	DQ239145
Tribolonotus_novaeguineae	AY308472	AY308321	HM229445	-	HM229523
Tropidophorus_baconi	AB222937	AB222953	-	-	-
Tropidophorus_baviensis	AB222942	AB222958	-	-	-
Tropidophorus_beccarii	AB222935	AB222951	-	-	-
Tropidophorus_berdmorei	AB028811	AB028823	-	-	-
Tropidophorus_brookei	AB222933	AB222949	DQ675192	-	-
Tropidophorus_cocincinensis	AB222943	AY308323	-	-	-
Tropidophorus_grayi	AB222941	AB222957	-	-	-
Tropidophorus_hainanus	AB222944	AB222960	-	-	-
Tropidophorus_latiscutatus	AB222934	AB222950	-	-	-
Tropidophorus_matsuii	AB222936	AB222952	-	-	-
Tropidophorus_microlepis	AB222947	AB222963	-	-	-
Tropidophorus_misaminius	AB222948	AB222964	-	-	-
Tropidophorus_murphyi	AB222945	AB222961	-	-	-
Tropidophorus_partelloi	AB222946	AB222962	-	-	-
Tropidophorus_robinsoni	AB222939	AB222955	-	-	-
Tropidophorus_sinicus	AB222938	AB222954	-	-	-
Tropidophorus_thai	AB222940	AB222956	-	-	-
Typhlosaurus_lomiae	DQ249020	HQ180022	AY662554	-	DQ249097
Tytthoscincus_aesculeticola	JF497970	JF498100	JF498208	JF498570	-
Tytthoscincus_atrigularis	JF497972	JF498102	JF498210	JF498572	-
Tytthoscincus_hallieri	JF497973	JF498104	JF498212	JF498574	-
Tytthoscincus_parvus	JF497974	JF498106	JF498214	JF498576	-
**Outgroup**					
Xantusia_bezyi	AY140984	AY140993	EU130270	AY584432	EU116615
Xantusia_vigilis	DQ249024	DQ249035	EU130279	AY584459	EU116656
Lepidophyma_flavimaculatum	NC_008775	DQ249036	NC_008775	AB162908	EU116546
Broadleysaurus_major	AJ416921	AJ416922	AY167396	-	DQ090881
Cordylosaurus_subtessellatus	AY218001	AY217951	AY167393	-	AY217797
Zonosaurus_ornatus	AJ416929	AJ416930	-	-	DQ004409
Smaug_warreni	HQ167133	HQ167244	NC_005962	AB079613	DQ249103
Namazonurus_namaquensis	HQ167104	HQ167215	-	-	EU116507
Cordylus_tropidosternum	HQ167125	HQ167236	-	-	DQ090880
Carphodactylus_laevis	AF090175	GU460142	AY369017	-	AF109565

Delma_borea	-	AY134511	AY134583	-	-
Oedura_tryoni	AF090179	AY583929	EF532898	JQ398487	AY583956
Coleonyx_variegatus	AB114446	AY140999	AB114446	NC_008774	EU116505
Gonatodes_albogularis	GU139932	EF564024	JX041354	-	-
Phyllodactylus_nocticolus	FJ662583	FJ662583	JX041409	FJ662561	-
Gekko_gecko	HM370130	HM370130	AY282753	AY282753	NC_007627
Hemidactylus_brookii	HM595685	HM040938	EU268375	EU268438	GQ375294
Uroplatus_phantasticus	EU596664	GU129013	EF490800	JN038120	EF490773
Paroedura_androyensis	GU128974	GU129000	EF490774	EF536218	EF490748
Dibamus_novaeguineae	-	-	FJ195390	FJ195390	EU116508

**Species**	**C-mos**	**BDNF**	**R35**	**RAG1**
Ablepharus_pannonicus	AF039466	-	-	-
Acontias_meleagris	AY662572	GU457870	HQ876348	AY662639
Amphiglossus_reticulatus	FJ667670	FJ667641	-	-
Anomalopus_swansoni	HQ655196	HQ655159	-	-
Asymblepharus_alaicus	-	-	-	-
Asymblepharus_nyingchiensis	-	-	-	-
Asymblepharus_sp	-	-	-	-
Asymblepharus_sikimmensis	-	-	-	AY662631
Asymblepharus_medogensis	-	-	-	-
Brachymeles_bicolor	AY818797	HQ907281	HQ907689	-
Brachymeles_bonitae	-	HQ907250	HM161057	HM161152
Brachymeles_gracilis	-	HQ907295	JN568499	JN654856
Brachymeles_miriamae	-	HQ907236	HQ907643	-
Brachymeles_talinis	-	HQ907266	HQ907674	-
Calyptotis_scutirostrum	-	-	-	-
Carlia_fusca	-	-	-	-
Chalcides_ocellatus	AY818798	HM160584	HM161058	AY662638
Chalcides_sexlineatus	-	JQ073128	-	JQ073251
Chalcides_viridanus	-	JQ073129	-	JQ073252
Ctenotus_labillardieri	-	-	-	-
Dasia_grisea	-	HQ907226	HQ907631	-
Dasia_olivacea	-	-	-	-
Dasia_vittata	DQ675393	-	-	DQ675338
Egernia_stokesii	HQ655203	HQ655166	-	-
Emoia_atrocostata	-	HQ907222	HQ907627	-
Emoia_caeruleocauda	AY217859	HM160585	HM161059	HM161154
Emoia_cyanura	AY217865	-	JF498338	DQ675343
Eugongylus_rufescens	AY818779	JN654799	JN568485	JN654859
Eulamprus_kosciuskoi	-	-	-	-
Eulamprus_quoyii	-	-	-	-
Eumeces_schneideri	-	HM160586	HM161060	HM161155
Eurylepis_taeniolata	-	HM160587	HM161061	HM161156
Eutropis_allapallensis	JQ767942	-	-	-

Eutropis_beddomei	JQ767946	-	-	-
Eutropis_bibronii	JQ767947	-	-	-
Eutropis_carinata	JQ767941	-	-	-
Eutropis_clivicola	JQ767945	-	-	-
Eutropis_cumingi	DQ238977	-	-	-
Eutropis_dissimilis	-	-	-	-
Eutropis_indeprensa	-	-	-	-
Eutropis_longicaudata	EU366457	-	-	-
Eutropis_macrophthalma	-	-	-	-
Eutropis_macularia	DQ238976	-	-	-
Eutropis_madaraszi	-	-	-	-
Eutropis_multicarinata	DQ675394	-	-	DQ675339
Eutropis_multifasciata	DQ238978	-	JF498340	AY444055
Eutropis_nagarjunensis	JQ767949	-	-	-
Eutropis_quadricarinata	-	-	-	-
Eutropis_rudis	DQ238975	-	-	-
Eutropis_rugifera	-	-	-	-
Eutropis_trivittata	JQ767948	-	-	-
Eutropis_tytleri	-	-	-	-
Feylinia_polylepis	AY662571	GU457871	JN568493	AY662637
Hemiergis_peronii	HQ655206	HQ655171	-	-
Heremites_auratus	-	-	-	AY662629
Heremites_vittatus	-	-	-	-
Insulasaurus_arborens	-	-	JF498341	-
Insulasaurus_victoria	-	-	JF498345	-
Insulasaurus_wrighti	-	-	JF498347	-
Isopachys_anguinoides	-	-	-	-
Lamprolepis_smaragdina	AY217854	HQ655172	HQ907626	-
Larutia_seribuatensis	-	HQ907229	HQ907636	-
Leptosiaphos_vigintiserierum	EU164504	-	-	-
Lerista_neander	HQ655208	HQ655173	-	-
Lipinia_noctua	AF039465	HQ655175	JF498348	-
Lipinia_pulchella	-	HQ907220	HQ907625	-
Lipinia_vittigera	-	-	-	-
Lygisaurus_novaeguineae	AY217861	-	-	-
Lygosoma_albopunctata	-	-	-	-
Lygosoma_bowringii	HQ655212	HQ655177	HQ907637	-
Lygosoma_koratense	-	-	-	-
Lygosoma_lineolatum	-	-	-	-
Lygosoma_punctata	-	-	-	DQ675345
Lygosoma_quadrupes	-	HQ907232	HQ907639	-
Mabuya_mabouya	-	-	JF498350	-
Madascincus_melanopleura	AY802768	HM160578	HM161052	HM161147
Mesoscincus_schwartzei	-	HM160593	HM161067	HM161162

Nannoscincus_mariei	DQ675372	-	-	EU568021
Oligosoma_aeneum	DQ675384	-	-	EU568058
Oligosoma_lichenigera	DQ675385	-	-	EU568108
Ophiomorus_punctatissimus	-	HM160594	HM161068	HM161163
Otosaurus_cumingi	-	-	JF498353	-
Papuascincus_stanleyanus	-	-	JF498355	-
Parvoscincus_steerei	-	-	JF498400	-
Parvoscincus_tagapayo	-	-	JF498402	-
Plestiodon_barbouri	-	HM160605	HM161079	HM161174
Plestiodon_capito	-	HM160608	HM161082	HM161177
Plestiodon_chinensis	-	HM160610	HM161084	HM161179
Plestiodon_egregius	-	HM160618	HM161092	HM161187
Plestiodon_elegans	-	HM160621	HM161095	HM161190
Plestiodon_fasciatus	AY217869	HQ876228	HQ876347	HQ876444
Plestiodon_japonicus	-	HM160629	HM161103	HM161198
Plestiodon_kishinouyei	-	HM160631	HM161105	HM161200
Plestiodon_laticeps	EU116681	EU108357	HM161107	EU108531
Plestiodon_latiscutatus	-	HM160635	HM161109	HM161204
Plestiodon_marginatus	-	HM160640	HM161114	HM161209
Plestiodon_quadrilineatus	-	HQ907223	HM161125	HM161221
Plestiodon_skiltonianus	AF315396	HM160658	HM161131	AY662633
Plestiodon_stimpsonii	-	HM160660	HM161133	HM161229
Plestiodon_tamdaoensis	-	HM160663	HM161136	HM161232
Plestiodon_tunganus	-	HM160666	HM161139	HM161235
Ristella_rurkii	-	-	-	-
Scelotes_mirus	AY217879	JQ073075	-	JQ073196
Scincella_assata	-	-	JF498428	-
Scincella_cherriei	-	-	JF498429	-
Scincella_doriae	-	-	-	-
Scincella_huanrenensis	-	-	-	-
Scincella_lateralis	AY217857	HQ655188	HM161140	HM161236
Scincella_potanini	-	-	-	-
Scincella_reevesii	HQ655222	HQ655189	HQ907634	-
Scincella_rupicola	-	-	-	-
Scincella_tsinlingensis	-	-	-	-
Scincella_vandenburghi	-	-	-	-
Scincus_mitranus	AY818806	-	-	-
Sphenomorphus_acutus	-	-	JF498431	-
Sphenomorphus_buenloicus	-	-	-	-
Sphenomorphus_concinnatus	-	-	JF498432	-
Sphenomorphus_cranei	-	-	JF498434	-
Sphenomorphus_cyanolaemus	-	-	JF498436	-
Sphenomorphus_diwata	-	-	JF498438	-
Sphenomorphus_fasciatus	DQ675380	-	JF498440	DQ675320

Sphenomorphus_indicus	-	-	JF498441	-
Sphenomorphus_jobiensis	DQ675395	HQ655190	-	-
Sphenomorphus_leptofasciatus	AF039464	-	-	-
Sphenomorphus_maculatus	-	-	JF498442	-
Sphenomorphus_maculatus	-	-	-	-
Sphenomorphus_maindroni	-	-	-	-
Sphenomorphus_melanopogon	-	-	-	-
Sphenomorphus_muelleri	-	-	-	-
Sphenomorphus_multisquamatus	-	-	JF498443	-
Sphenomorphus_praesignis	-	-	-	-
Sphenomorphus_sabanus	-	-	JF498444	-
Sphenomorphus_scutatus	-	-	JF498445	-
Sphenomorphus_simus	EU116688	EU108365	-	EU108539
Sphenomorphus_solomonis	HQ655224	HQ655191	JN568487	JN654864
Sphenomorphus_stellatus	-	-	-	-
Sphenomorphus_variegatus	-	-	JF498448	-
Tiliqua_scincoides	AF039462	JN654804	JN568504	JN654866
Trachylepis_perrotetii	DQ238986	HM160591	HM161065	HM161160
Tribolonotus_novaeguineae	HM229567	-	-	-
Tropidophorus_baconi	-	-	-	-
Tropidophorus_baviensis	-	-	-	-
Tropidophorus_beccarii	-	-	-	-
Tropidophorus_berdmorei	-	-	-	-
Tropidophorus_brookei	DQ675397	-	-	-
Tropidophorus_cocincinensis	-	-	-	-
Tropidophorus_grayi	-	-	-	-
Tropidophorus_hainanus	-	-	-	-
Tropidophorus_latiscutatus	-	-	-	-
Tropidophorus_matsuii	-	-	-	-
Tropidophorus_microlepis	-	-	-	-
Tropidophorus_misaminius	-	-	-	-
Tropidophorus_murphyi	-	-	-	-
Tropidophorus_partelloi	-	-	-	-
Tropidophorus_robinsoni	-	-	-	-
Tropidophorus_sinicus	-	-	-	-
Tropidophorus_thai	-	-	-	-
Typhlosaurus_lomiae	DQ249065	-	-	DQ249129
Tytthoscincus_aesculeticola	-	-	JF498452	-
Tytthoscincus_atrigularis	-	-	JF498454	-
Tytthoscincus_hallieri	-	-	JF498455	-
Tytthoscincus_parvus	-	-	JF498456	-
**Outgroup**				
Xantusia_bezyi	EU116786	EU108462	-	EU108636
Xantusia_vigilis	EU116835	EU108491	HQ876351	AY662642

Lepidophyma_flavimaculatum	EU116715	GU457873	HQ876350	GU457995
Broadleysaurus_major	DQ100149	HM160588	HM161062	HM161157
Cordylosaurus_subtessellatus	AY217849	HQ876225	HQ876344	HQ876441
Zonosaurus_ornatus	DQ100120	JN654807	JN568497	JN614040
Smaug_warreni	DQ249072	-	-	-
Namazonurus_namaquensis	AY217848	AY987981	-	EU108528
Cordylus_tropidosternum	DQ100148	-	-	-
Carphodactylus_laevis	EF534905	-	-	EF534781
Delma_borea	AY134547	GU457867	HQ876381	GU457990
Oedura_tryoni	AF090848	-	-	JQ173758
Coleonyx_variegatus	EF534901	EU108352	HQ876371	HQ876448
Gonatodes_albogularis	EF564078	GU457866	HQ876380	EF534797
Phyllodactylus_nocticolus	FJ662518	FJ662489	-	HQ426311
Gekko_gecko	EU366455	EU402614	HQ876378	AY662625
Hemidactylus_brookii	AY863049	HM180358	-	GQ375314
Uroplatus_phantasticus	HQ426592	EU596897	-	EF490747
Paroedura_androyensis	HQ256721	-	-	EF490721
Dibamus_novaeguineae	EF450999	EU108355	DQ119613	GU457986

Gekkonidae

**Species**	**ND2**	**RAG1**	**PDC**
Alsophylax_pipiens	KC151973	KC152020	KC151995
Altiphylax_medogensis	**MW111448**	**MW111362**	**MW111427**
Altiphylax_medogensis	**MW111449**	**MW111363**	**MW111428**
Altiphylax_stolickzai	KJ794404	KJ794381	KJ794394
Altiphylax_levitoni	KC151974	KC152022	KC151997
Calodactylodes_illingworthorum	JX041318	JQ945288	JQ945356
Chondrodactylus_fitzsimonsi	JN393945	EU293645	EU293712
Crossobamon_orientalis	KC151975	KC152023	KC151998
Cyrtodactylus_adleri	-	-	KM255168
Cyrtodactylus_angularis	JX440523	JQ945301	JX440632
Cyrtodactylus_annandalei	JX440524	JX440683	JX440633
Cyrtodactylus_ayeyarwadyensis	JX440526	JX440685	JX440634
Cyrtodactylus_battalensis	KC151983	KC152035	KC152007
Cyrtodactylus_bintangtinggi	JX519494	-	-
Cyrtodactylus_brevidactylus	JX440527	JX440687	JX440636
Cyrtodactylus_cayuensis	**MW111436**	**MW111364**	**MW111421**
Cyrtodactylus_cayuensis	**MW111437**	**MW111365**	**MW111422**
Cyrtodactylus_collegalensis	-	KC735087	KC735092
Cyrtodactylus_chanhomeae	JX440529	JX440688	JX440637
Cyrtodactylus_chrysopylos	JX440531	JX440690	JX440639
Cyrtodactylus_deccanensis	JX440521	JX440681	JX440630
Cyrtodactylus_elok	JQ889180	JX440694	JX440643
Cyrtodactylus_fasciolatus	KM255184	KM255120	KM255143
Cyrtodactylus_feae	JX440536	JX440696	JX440645
Cyrtodactylus_gansi	JX440537	JX440697	JX440646
Cyrtodactylus_gubernatoris	KM255204	KM255123	KM255147
Cyrtodactylus_himalayanus	KM255173	KM255124	KM255149
Cyrtodactylus_hontreensis	JX440539	JX440699	-
Cyrtodactylus_interdigitalis	JQ889181	JX440700	JX440648
Cyrtodactylus_intermedius	JQ889182	JX440701	JX440649
Cyrtodactylus_jarujini	JX440541	JQ945303	JX440651
Cyrtodactylus_jellesmae	JX440542	JX440702	JX440652
Cyrtodactylus_khasiensis	KM255188	KM255127	KM255152
Cyrtodactylus_lawderanus	KM255189	KM255128	KM255153
Cyrtodactylus_loriae	EU268350	EU268289	EU268319
Cyrtodactylus_novaeguineae	JX440547	HQ426274	HQ426185
Cyrtodactylus_paradoxus	JX440549	JX440709	JX440659
Cyrtodactylus_peguensis	GU550727	-	-
Cyrtodactylus_philippinicus	JX440550	JQ945304	JX440660
Cyrtodactylus_pulchellus	JX440552	JX440711	JX440662
Cyrtodactylus_quadrivirgatus	JX440553	JX440712	JX440663

Cyrtodactylus_rubidus	KM255203	-	-
Cyrtodactylus_russelli	JX440555	-	-
Cyrtodactylus_sadlieri	JQ820309	-	-
Cyrtodactylus_semenanjungensis	JQ889177	JX440716	JX440666
Cyrtodactylus_sermowaiensis	JX440558	JX440718	JX440668
Cyrtodactylus_slowinskii	JX440559	JX440719	-
Cyrtodactylus_sp_Timor	JX440560	JX440720	JX440669
Cyrtodactylus_tibetanus	**MW111438**	**MW111366**	**MW111423**
Cyrtodactylus_tibetanus	**MW111439**	-	**MW111424**
Cyrtodactylus_tigroides	JX440562	JX440723	JX440671
Cyrtodactylus_tiomanensis	JX440563	JX440724	JX440672
Cyrtodactylus_triedra	JX440522	JX440682	JX440631
Cyrtodactylus_zhaoermii	**MW111440**	**MW111367**	**MW111425**
Cyrtodactylus_zhaoermii	**MW111441**	**MW111368**	**MW111426**
Cyrtodactylus sp_CHNG	KM255179	-	-
Cyrtodactylus_septentrionalis	-	KM255133	KM255158
Cyrtodactylus chamba	KM255191	KM255134	KM255159
Cyrtodactylus sp._Glow	KM255193	-	-
Cyrtodactylus guwahatiensis	KM255194	KM255135	KM255160
Cyrtodactylus jaintiaensis	KM255195	-	-
Cyrtodactylus sp._Khellong	KM255196	-	-
Cyrtodactylus kazirangaensis	KM255170	KM255136	KM255161
Cyrtodactylus sp._Mizoram	KM255197	-	-
Cyrtodactylus nagalandensis	KM255199	KM255137	KM255162
Cyrtodactylus_montanus	KM255200	KM255138	KM255163
Cyrtodactylus sp._Sikkim	KM255181	KM255139	KM255165
Cyrtodactylus tripuraensis	KM255182	KM255141	KM255166
Cyrtodactylus_oldhami	JX440548	JX440707	JX440657
Cyrtopodion'_aravallense	KJ794406	KJ794372	KJ794385
Cyrtopodion_mansarulus	KJ794414	KJ794383	KJ794396
Cyrtopodion_scabrum	JX041345	HQ426275	HQ426186
Dixonius_vietnamensis	EU054297	EU054281	EU054265
Eublepharis_macularius	JX041350	EF534776	EF534816
Gecko_smithi	JN019056	JN019121	JN019090
Gehyra_mutilata	JN019081	JN019145	JN019113
Gekko_gecko	AF114249	EU054272	EU054256
Gekko_monarchus	JN019078	JN019142	JN019110
Gekko_vittatus	JN019072	JN019137	JN019105
Hemidactylus_anamallensis	-	HM622353	HM622368
Hemidactylus_angulatus	HM559620	HM559686	HM559653
Hemidactylus_aquilonius	EU268373	EU268312	EU268342
Hemidactylus_brasilianus	EU268351	EU268290	EU268320
Hemidactylus_brookii	GQ458051	GQ375313	GQ375307
Hemidactylus_craspedotus	HM559618	HM559684	HM559651

Hemidactylus_depressus	HM559625	HM559691	HM559658
Hemidactylus_fasciatus	EU268371	EU268310	EU268340
Hemidactylus_frenatus	GQ458049	GQ375308	GQ375301
Hemidactylus_garnotii	EU268363	EU268302	EU268332
Hemidactylus_karenorum	EU268362	EU268301	EU268331
Hemidactylus_leschenaulti	HM559636	HM559702	HM559669
Hemidactylus_persicus	EU268377	EU268316	EU268346
Hemidactylus_platyurus	HM559619	HM559685	HM559652
Hemidactylus_platyurus	-	**MW111369**	**MW111429**
Hemiphyllodactylus_aurantiacus	JN393933	JN393977	JN394011
Hemiphyllodactylus_zayuensis	**MW111434**	-	-
Hemiphyllodactylus_zayuensis	**MW111435**	-	-
Hemiphyllodactylus_yunnanensis	JN393949	-	-
Altiphylax_levitoni	KC151974	KC152022	KC151997
Hemidactylus_aaronbaueri	-	HM622352	HM622367
Hemidactylus_bowringii	-	HM622354	HM622369
Hemidactylus_flaviviridis	-	EU268294	EU268324
Hemidactylus_giganteus_2	-	HM622357	HM622372
Hemidactylus_gracilis	-	HM622359	HM622374
Hemidactylus_greeffii	-	EU268308	EU268338
Hemidactylus_haitianus	-	EU268311	EU268341
Hemidactylus_imbricatus	-	EU268292	EU268322
Hemidactylus_mabouia	-	EU268300	EU268330
Hemidactylus_maculatus	-	HM622361	HM622375
Hemidactylus_palaichthus	-	EU268307	EU268337
Hemidactylus_prashadi	-	HM622364	HM622378
Hemidactylus_robustus	-	EU268315	EU268345
Hemidactylus_triedrus	-	HM622365	HM622379
Hemiphyllodactylus_banaensis	KF219783	-	-
Hemiphyllodactylus_bintik	KJ663757	-	-
Hemiphyllodactylus_changningensis	KP732437	-	-
Hemiphyllodactylus_chiangmaiensis	KF219781	-	-
Hemiphyllodactylus_dushanensis	FJ971016	-	-
Hemiphyllodactylus_ganoklonis	JN393950	-	-
Hemiphyllodactylus_harterti	KF219760	-	-
Hemiphyllodactylus_insularis	KF219762	-	-
Hemiphyllodactylus_jinpingensis	FJ971038	-	-
Hemiphyllodactylus_larutensis	KJ663758	-	-
Hemiphyllodactylus_longlingensis	FJ971045	-	-
Hemiphyllodactylus_tehtarik	KF219784	-	-
Hemiphyllodactylus_titiwangsaensis	KF219785	-	-
Hemiphyllodactylus_typus	KF219797	-	-
Homopholis_walbergii	EU054244	EU054220	EU054196
Lepidodactylus_lugubris	JN393944	JX515629	JX515642

Lepidodactylus_orientalis	JN019080	JN019144	JN019112
Lialis_burtonis	-	GU459540	GU459742
Lialis_jicari	AY369025	-	-
Luperosaurus_cumingii	JX515623	JX515637	JX515650
Mediodactylus_russowii	JX440517	JX440678	JX440627
Microgecko_persicus	KJ794409	KJ794375	KJ794388
Nactus_vankampeni	EU054295	EU054279	EU054263
Oedura_marmorata	GU459951	EF534779	EF534819
Phelsuma_inexpectata	JN393939	JN393983	JN394016
Phelsuma_rosagularis	EU423292	HQ426306	HQ426217
**Outgroup**			
Phyllodactylus_xanti	JN393940	EF534807	EF534849
Ptyodactylus_guttatus	JX041426	EU293636	EU293703
Pygopus_nigriceps	JX440518	EF534783	EF534823
Sphaerodactylus_roosevelti	JN393943	EF534785	EF534825
Sphaerodactylus_torrei	JX440519	EF534788	EF534829
Tenuidactylus_caspius	KC151988	KC152039	KC152012
Tropiocolotes_nubicus	KC151991	KC152042	KC152015
Woodworthia_maculata	GU459852	GU459449	GU459651

Viperidae

**Species**	**12s**	**16s**	**Cytb**	**ND4**
Agkistrodon_contortrix	AF259224	AF259117	EU483263	AF156576
Agkistrodon_piscivorus	KX694610	KX694680	DQ523161	DQ523161
Atheris_ceratophora	DQ305410	DQ305433	DQ305456	DQ305474
Atheris_chlorechis	KX694550	KX694618	KX694841	EU624211
Atheris_nitschei	AY223650	AY223663	AF471070	AY223618
Atheris_squamigera	AF544762	EU624279	EU624303	EU624212
Atropoides_picadoi	AF057208	AF057255	AY223583	U41872
Azemiops_feae	KX694579	KX694684	KX694840	AY352808
Bitis_arietans	KX694571	KX694619	KX694845	EU852304
Bitis_gabonica	KX694551	KX694671	KX694846	EU624217
Bitis_nasicornis	DQ305411	DQ305434	DQ305457	DQ305475
Bitis_rubida	EU624251	EU624286	EU624306	EU624219
Bitis_worthingtoni	EU624252	AJ275745	AJ275692	EU624220
Bitis_xeropaga	EU624253	EU624287	EU624307	EU624221
Bothriechis_lateralis	AF057211	AF057258	AY223588	U41873
Bothriechis_nigroviridis	AF057212	AF057259	AY223589	AY223635
Bothriechis_schlegelii	AF057213	AF057260	AY223590	AF292611
Bothrops_ammodytoides	AY223658	AY223671	AY223595	AY223639
Bothrops_asper	AF057218	AF057265	AY223599	U41876
Bothrops_brazili	EU867252	EU867264	AF292597	AF292635
Bothrops_chloromelas	DQ305430	DQ305453	DQ305471	DQ305488
Bothrops_erythromelas	AF057219	AF057266	AY223600	U41877
Bothrops_jararaca	KX694583	EU867266	KX694833	AY122860
Bothrops_pictus	-	-	AF292583	AF292621
Calloselasma_rhodostoma	KX694585	KX694626	AF292569	AY352813
Causus_lichtensteini	KX694560	KX694628	KX694848	-
Causus_resimus	AY223649	AY223662	AY223555	AY223616
Causus_rhombeatus	DQ305409	DQ305432	DQ305455	U41866
Cerastes_cerastes	KX694540	KX694629	AF471028	EU624222
Cerastes_gasperettii	JN870181	AJ275756	AJ275704	-
Cerastes_vipera	-	AJ275757	AJ275705	-
Mixcoatlus_barbouri	HM363639	HM363640	HM363641	HM363642
Cerrophidion_tzotzilorum	JN870182	JN870193	DQ061203	DQ061228
Crotalus_adamanteus	KX694608	KX694635	AF259185	U41880
Crotalus_basiliscus	AF259244	AF259136	AY704845	AY704894
Crotalus_cerastes	AF259235	AF259128	U69773	JN620962
Crotalus_enyo	AF259245	AF259137	-	-
Crotalus_pricei	AF259237	AF259130	-	JN022878
Crotalus_ruber	AF259261	AF259153	-	DQ679838
Crotalus_willardi	KX694609	KX694682	KX694839	JN870209
Daboia_mauritanica	EU624261	EU624295	EU624313	EU624229

Daboia_palaestinae	JN870183	AJ275775	AJ275722	-
Daboia_siamensis	EU913478	NC011391	NC_011391	EU913478
Deinagkistrodon_acutus	KX694593	KX694683	KX694847	AY352811
Echis_borkini	GQ359644	GQ359730	GQ359486	GQ359563
Echis_carinatus	EU852313	EU852319	EU852295	GQ359524
Echis_coloratus	EU852315	EU852321	EU852297	EU624224
Echis_jogeri	GQ359641	GQ359732	GQ359476	GQ359560
Echis_leucogaster	GQ359622	GQ359705	GQ359456	GQ359541
Echis_omanensis	GQ359631	GQ359715	GQ359466	GQ359550
Echis_pyramidum	EU852314	EU852320	EU852296	EU624226
Eristicophis_macmahoni	EU624259	EU624293	AJ275711	EU624227
Garthius_chaseni	AY352791	AY352729	AY352760	AY352825
Gloydius_blomhoffi	AY352780	AY352719	AY352751	AY352814
Gloydius_brevicaudus	AY352781	AY352720	AY352752	AY352815
Gloydius_halys	KY040526	KY040558	KX063802	KX063775
Gloydius_intermedius	KY040524	KY040556	KY040617	KY040638
Gloydius_stejnegeri	KY040536	KY040568	KX063817	KX063790
Gloydius_cognatus	KY040529	KY040561	KY040619	KY040640
Gloydius_liupanensis	EF012814	-	JQ687491	JQ687472
Gloydius_qinglinensis	KY040534	KY040566	KY040623	KY040644
Gloydius_changdaoensis	KY040521	KY040553	KX063821	KX063794
Gloydius_shedaoensis	AF057194	AF057241	AY223566	AY223623
Gloydius_huangi	MK227409	MK227412	MK227415	MK227418
Gloydius_strauchi	KY040543	KY040575	KY040629	KY040650
Gloydius_monticola	KY040550	KY040582	KY040636	JX661243
Gloydius_rubromaculatus	KY040548	KY040580	KY040634	KY040655
Gloydius_angusticep	KY040540	KY040572	KY040626	KY040647
Gloydius_tsushimaensis	JN870186	JN870196	JN870203	JN870211
Gloydius_ussuriensis	AF057193	AF057240	AY223565	AY223622
Hypnale_hypnale	AY352778	AY352717	AY352750	AY352812
Hypnale_nepa	KC347325	KC347362	KC347485	KC347491
Hypnale_zara	KC347326	KC347363	KC347463	KC347513
Lachesis_acrochorda	JN870187	JN870197	JN870204	JN870212
Macrovipera_lebetina	KX694561	KX694652	AJ275713	DQ897729
Macrovipera_schweizeri	EU624262	AJ275768	AJ275715	-
Mixcoatlus_browni	HM363650	HM363651	HM363652	HM363653
Mixcoatlus_melanurus	AF057210	AF057257	AY223587	AY223634
Montivipera_albizona	EU624265	AJ275780	AJ275727	DQ897731
Montivipera_wagneri	JN870188	AJ275778	AJ275725	JN870213
Montivipera_xanthina	EU624268	AJ275777	AJ275724	EU624234
Ophryacus_undulatus	AF057209	AF057256	AY223586	AY223633
Ovophis_monticola	HQ325260	HQ325078	HQ325138	HQ325199
Ovophis_monticola	**MW020347**	**MW020093**	**MW111485**	-
Ovophis_monticola	HQ325306	HQ325119	HQ325179	HQ325241

Ovophis_makazayazaya	HQ325266	HQ325084	HQ325143	HQ325202
Ovophis_makazayazaya	DQ305417	DQ305440	DQ305463	DQ305481
Ovophis_convictus	HQ325264	HQ325082	HQ325129	HQ325190
Ovophis_okinavensis	AF057199	AF057246	AY223573	U41895
Ovophis_tonkinensis	HQ325308	HQ325121	HQ325181	HQ325243
Ovophis_tonkinensis	HQ325255	HQ325073	HQ325133	HQ325194
Ovophis_tonkinensis	HQ325274	HQ325096	HQ325151	HQ325209
Ovophis_zayuensis	HQ325273	HQ325089	HQ325150	HQ325208
Ovophis_zayuensis	**MW020348**	**MW020094**	**MW111486**	**MW111430**
Ovophis_zayuensis	HQ325304	HQ325118	HQ325177	HQ325239
Porthidium_ophryomegas	AF057205	AF057252	AY223580	U41888
Porthidium_yucatanicum	JN870189	JN870198	DQ061215	DQ061244
Proatheris_superciliaris	EU624263	EU624296	AJ275685	EU624230
Protobothrops_cornutus	AY294276	AY294267	AY294272	AY294262
Protobothrops_dabieshanensis	NC_022473	NC_022473	NC_022473	NC_022473
Protobothrops_elegans	AF057201	AF057248	AY223575	U41893
Protobothrops_flavoviridis	AY352792	AY352730	AY223574	AY352826
Protobothrops_himalayanus	**MW133302**	**MW020036**	**MW111487**	**-**
Protobothrops_jerdonii	AY763180	AY763199	HM567474	EU810020
Protobothrops_jerdonii	**MW133303**	**MW020037**	**MW111488**	**-**
Protobothrops_jerdonii	**MW133304**	**MW020038**	KF997884	**-**
Protobothrops_jerdonii	**MW020039**	MW020039	**MW111489**	**-**
Protobothrops_jerdonii	**MW133305**	**MW020040**	**-**	**-**
Protobothrops_jerdonii	**MW133306**	**MW020041**	**MW111490**	**-**
Protobothrops_kaulbacki	DQ666056	DQ666055	DQ666060	DQ666057
Protobothrops_mangshanensis	AY352787	AY352726	AY352758	AY352821
Protobothrops_maolanensis	JN799405	JN799398	JN799401	JN799409
Protobothrops_mucrosquamatus	AY223653	AY223666	AY223577	AY223629
Protobothrops_tokarensis	AF057202	AF057249	AY223576	AY223628
Protobothrops_trungkhanhensis	KT220284	KT220303	KT220323	KT220343
Protobothrops_trungkhanhensis	**MW133307**	**MW020042**	**MW111491**	**-**
Protobothrops_xiangchengensis	AY763188	AY763207	DQ666061	DQ666058
Protobothrops_sieversorum	DQ305414	DQ305437	AY352753	DQ305478
Pseudocerastes_fieldi	EU624264	AJ275769	AJ275716	-
Sistrurus_catenatus	DQ464268	AF259119	AY223610	HQ257759
Sistrurus_miliarius	KX694565	KX694679	EU483385	HQ257760
Trimeresurus_albolabris	AF057195	AF057242	AY223567	U41890
Trimeresurus_andersonii	AY352801	AY352740	-	AY352835
Trimeresurus_popeiorum	AY371753	AY371769	AY371801	AY371837
Trimeresurus_borneensis	AY352783	AY352722	AY352754	AY352817
Trimeresurus_popeiorum	AY371752	AY371778	AY371818	AY371853
Trimeresurus_cantori	AY352802	AY352741	AF171889	AY352836
Trimeresurus_erythrurus	AF517161	AF517174	AF171900	AF517217
Trimeresurus_fasciatus	GQ428492	GQ428466	GQ428475	GQ428482

Trimeresurus_flavomaculatus	AY059535	AY059551	AF171916	AY059584
Trimeresurus_popeiorum	AY059537	AY059553	AY371796	AY059588
Trimeresurus_gracilis	DQ305415	DQ305438	AF171913	AY352823
Trimeresurus_gramineus	AY352793	AY352731	AY352761	AY352827
Trimeresurus_gumprechti	EU443791	EU443792	AY321489	EU443787
Trimeresurus_hageni	AY059536	AY059552	AY059567	AY059585
Trimeresurus_insularis	AY059534	AY352738	AY059568	AY352833
Trimeresurus_kanburiensis	AY289219	AY352737	AY289225	AY289231
Trimeresurus_macrops	AF517163	AF517176	AF517184	AF517219
Trimeresurus_malabaricus	AY059548	AY059564	AY059569	AY059587
Trimeresurus_malcolmi	AY371757	AY371786	AY371832	AY371861
Trimeresurus_mcgregori	AY371756	AY371795	AY371831	AY371858
Trimeresurus_medoensis	AY352797	AY352735	AY352765	AY352831
Trimeresurus_medoensis	**MW020335**	**MW020095**	**MW111479**	**-**
Trimeresurus_popeiorum	AY371737	AY371774	AY371814	AY371839
Trimeresurus_popeiorum	AY059538	AY059554	AY059571	AY059590
Trimeresurus_puniceus	AF517164	AF517177	AF517192	AF517220
Trimeresurus_purpureomaculatus	AF517162	AF517175	AF517188	AF517218
Trimeresurus_sabahi	AY371736	AY371771	AY371815	AY371842
Trimeresurus_schultzei	AY352785	AY352725	AY352756	AY352819
Trimeresurus_septentrionalis	AY059543	AY352724	AY352755	AY352818
Trimeresurus_sichuanensis	HQ850445	HQ850446	HQ850448	HQ850450
Trimeresurus_stejnegeri	AY059539	AY059555	AF171896	AY059593
Trimeresurus_sumatranus	AY371759	AY371788	AY371828	AY371866
Trimeresurus_tibetanus	AY352776	AY352715	AY352749	AY352810
Trimeresurus_trigonocephalus	AY059549	AY059565	AF171890	AY059597
Trimeresurus_truongsonensis	EU443817	EU443818	EU443815	EU443816
Trimeresurus_venustus	AY293931	AY352723	AF171914	AY293930
Trimeresurus_vogeli	AF517170	AF517183	AY059581	AF517225
Trimeresurus_yunnanensis	EU443813	EU443814	EF597523	EF597527
Tropidolaemus_subannulatus	AF057198	AF057245	AY223571	AY223625
Tropidolaemus_wagleri	AF517167	AF517180	AF517191	AF517223
Vipera_ammodytes	EU624266	EU624297	AM944799	EU624232
Vipera_berus	EU543221	DQ186081	DQ186032	DQ897728
Vipera_eriwanensis	KX694555	KX694672	KX694842	FR727021
Vipera_kaznakovi	-	-	AY321093	FR727034
Vipera_latastei	-	-	AY321094	JX649572
Vipera_lotievi	-	-	JN204717	FR727030
Vipera_renardi	-	-	HQ845740	FR727033
Vipera_seoanei	-	AJ275782	DQ186030	FR727035
Vipera_ursinii	-	-	AY311383	FR726981
**Outgroup**				
Achalinus_meiguensis	FJ424614	FJ424614	FJ424614	FJ424614
Achalinus_rufescens	KX694570	KX694613	KX694895	U49319

Acrochordus_granulatus	AB177879	NC_007400	NC_007400	NC_007400
Acrochordus_javanicus	KX694587	KX694614	KX694897	HM234055
Ahaetulla_prasina	KX694556	KX694615	-	-
Ahaetulla_pulverulenta	KC347304	-	KC347454	KC347512
Anilius_scytale	FJ755180	-	FJ755180	FJ755180
Antillophis_parvifrons	KX694557	KX694616	KX694883	FJ416814
Aparallactus_modestus	FJ404130	AY611824	AY612007	FJ404332
Aplopeltura_boa	AF544761	AF544787	-	U49312
Aspidelaps_scutatus	KX694563	KX694617	KX694861	AY058969
Atractaspis_corpulenta	Z46597	AY611837	AY612020	FJ404335
Atractaspis_micropholis	AF544740	AY611823	AY612006	FJ404336
Boa_constrictor	NC_007398	NC_007398	AF471036	NC_007398
Boaedon_fuliginosus	AY122681	AY188079	AF471060	HQ207151
Brachyorrhos_albus	KX694569	KX694674	KX694849	-
Broghammerus_reticulatus	Z46448	-	FJ717481	-
Bungarus_fasciatus	NC_011393	NC_011393	EU579523	EU579523
Calabaria_reinhardtii	Z46464	-	AY099985	AF302943
Calamaria_pavimentata	KX694584	KX694624	KX694890	-
Calamaria_yunnanensis	KX694572	KX694625	KX694891	-
Cantoria_violacea	KX694595	KX694627	KX694852	-
Cerberus_rynchops	KX694596	KX694630	KX694850	U49327
Chrysopelea_ornata	KX694558	KX694631	KX694862	KC347496
Coluber_constrictor	KX694604	KX694632	EU180486	AF138746
Compsophis_infralineatus	KX694601	KX694633	EF203990	FJ404359
Coronella_austriaca	KX694589	KX694634	KX694868	AY487065
Cubophis_cantherigerus	AF158405	AF158475	AF544669	FJ416818
Cylindrophis_ruffus	NC_007401	-	NC_007401	NC_007401
Diadophis_punctatus	AY577015	KX694636	EU193843	EU194025
Dipsas_catesbyi	Z46459	KX694637	KX694856	EF078585
Dromicodryas_bernieri	KX694586	KX694638	DQ979987	FJ404353
Drymarchon_corais	HM565758	KX694639	KX694871	DQ902314
Duberria_lutrix	KX694598	KX694640	KX694892	DQ486308
Eirenis_modestus	AY039143	KX694641	KX694864	AY487072
Erpeton_tentaculatus	KX694592	KX694642	KX694851	-
Erythrolamprus_miliaris	KX694588	KX694643	KX694884	-
Eryx_colubrinus	AF544747	-	U69811	-
Eunectes_notaeus	AM236347	-	AM236347	AM236347
Gonionotophis_brussauxi	FJ404156	KX694644	AY612043	FJ404358
Gonionotophis_capensis	AF544769	KX694645	HQ207116	AF544665
Gonyosoma_oxycephalum	AY122678	KX694646	KX694870	DQ902309
Grayia_ornata	AF158434	AF158503	KX694888	AF544663
Heterodon_platirhinos	AY577019	KX694647	KX694857	AF402659
Homalopsis_buccata	KX694597	KX694690	EF395917	-
Homoroselaps_lacteus	KX694590	KX694686	KX694889	AY058976

Imantodes_cenchoa	KX694602	KX694687	KX694858	NC_013988
Ithycyphus_miniatus	KX694576	KX694648	KX694863	-
Lampropeltis_getula	KX694603	KX694649	KX694866	AF138759
Laticauda_colubrina	U96799	EU547138	EU547040	AY058977
Leioheterodon_madagascariensis	KX694599	AY188061	KX694881	FJ404371
Leptodeira_annulata	GQ457806	GQ457746	EF078516	FJ416787
Liophidium_torquatum	KX694577	KX694650	KX694880	-
Loxocemus_bicolor	Z46456	-	AY099993	-
Lycophidion_capense	FJ404178	AY611893	AY612075	FJ404376
Macropisthodon_rudis	KX694559	KX694651	KX694875	U49326
Malpolon_monspessulanus	KX694541	KX694653	KX694879	FJ404320
Micrurus_surinamensis	AF544770	AF544799	EF137415	EF137407
Mimophis_mahafalensis	KX694543	-	KX694877	DQ486202
Naja_atra	KX694544	KX694689	KX694859	EU921898
Natrix_natrix	KX694573	-	KX694876	AY873716
Nerodia_sipedon	KX694606	KX694654	KX694873	-
Ninia_atrata	GQ457814	KX694675	KX694854	GQ334659
Ophiophagus_hannah	KX694600	KX694688	KX694860	EU921899
Pantherophis_guttatus	AY122814	KX694685	AM236349	AM236349
Pareas_carinatus	AF544773	AF544802	JQ598940	-
Pareas_hamptoni	KX694545	KX694656	KX694896	-
Pituophis_deppei	KX694582	KX694657	KX694867	AF138765
Polemon_acanthias	FJ404138	AY611848	AY612031	FJ404341
Psammophis_elegans	KX694546	KX694659	-	EU526862
Psammophis_mossambicus	KX694547	KX694660	KX694878	DQ486283
Psammophis_subtaeniatus	-	KX694661	DQ486358	DQ486253
Psammophylax_variabilis	AF544774	AY611864	AY612046	EU526859
Pseudonaja_modesta	EU547098	KX694662	EU547049	DQ098490
Pseudoxenodon_bambusicola	KX694575	KX694678	-	-
Pseudoxenodon_karlschmidti	KX694578	KX694676	KX694893	-
Psomophis_joberti	GQ457829	GQ457768	KX694887	-
Ptyas_korros	KX694591	KX694663	KX694869	AY487062
Indotyphlops_braminus	DQ343649	-	DQ343649	NC_010196
Rhachidelus_brazili	JQ598837	JQ598897	KX694885	-
Rhamphiophis_oxyrhynchus	KX694548	KX694664	-	-
Sibynomorphus_mikanii	GQ457832	GQ457771	KX694855	-
Sibynophis_collaris	KX694581	KX694665	KC000121	JN211315
Sinomicrurus_macclellandi	KX694549	KX694666	KX694853	EF137410
Sinonatrix_percarinata	KX694542	KX694667	KX694872	JQ687414
Spalerosophis_diadema	KX694605	KX694668	KX694865	AY487059
Stoliczkia_borneensis	AF544779	AF544808	-	-
Storeria_dekayi	KX694607	KX694669	KX694874	EF417365
Taeniophallus_nicagus	KX694562	KX694677	KX694886	-
Thamnophis_sirtalis	KX694574	KX694670	AF420193	AF420196

Tomodon_dorsatus	GQ457838	GQ457777	KX694882	-
Xenochrophis_flavipunctatus	AF544780	AF544809	-	-
Xenodermus_javanicus	AF544781	AF544810	AY425810	U49320
Xenopeltis_unicolor	NC_007402	NC_007402	NC_007402	NC_007402

Colubridae

**Species**	**12s**	**16s**	**Cytb**	**ND4**	**ND2**
Ahaetulla_fronticincta	-	-	AF471072	-	-
Ahaetulla_nasuta	-	-	KC347453	KC347491	-
Ahaetulla_prasina	KX694556	KX694615	-	-	-
Ahaetulla_pulverulenta	KC347304	KC347339	KC347454	KC347492	-
Amphiesma_stolatum	-	-	JQ687432	JQ687425	JQ687464
Archelaphe_bella	-	-	DQ902134	DQ902316	DQ902248
Atretium_yunnanensis	-	-	GQ281787	JQ687423	JQ687463
Boiga_barnesii	KC347311	KC347345	KC347461	KC347499	-
Boiga_beddomei	KC347312	KC347346	KC347462	KC347500	-
Boiga_ceylonensis	KC347313	KC347347	KC347463	KC347501	-
Boiga_cynodon	Z46468	AF139568	-	-	-
Boiga_forsteni	KC347314	KC347348	KC347464	KC347502	-
Boiga_multomaculata	-	-	FJ710798	-	-
Boiga_trigonata	KC347315	KC347349	KC347465	KC347503	-
Calamaria_pavimentata	KX694584	KX694624	KX694890	-	-
Calamaria_yunnanensis	KX694572	KX694625	KX694891	-	-
Chrysopelea_ornata	KC347318	KC347353	KC347469	KC347506	-
Chrysopelea_taprobanica	KC347319	KC347354	KC347470	KC347507	-
Coelognathus_erythrurus	AY122665	-	DQ902108	DQ902288	DQ902215
Coelognathus_flavolineatus	AY122666	-	DQ902128	U49301	DQ902240
Coelognathus_helena	AY122674	-	DQ902112	DQ902292	DQ902219
Coelognathus_radiatus	AY122677	-	DQ902121	DQ902317	DQ902230
Coelognathus_subradiatus	AY122673	-	DQ902126	DQ902304	DQ902235
Coluber_constrictor	KX694604	KX694632	EU180486	AF138746	AY487002
Dendrelaphis_bifrenalis	KC347321	KC347356	KC347473	KC347509	-
Dendrelaphis_cyanochloris	-	-	**MW111475**	**MW111431**	-
Dendrelaphis_cyanochloris	-	-	KX660454	KX660580	-
Dendrelaphis_caudolineatus	AF544782	AF544811	GQ895864	-	-
Dendrelaphis_formosus	-	-	KX660438	KX660565	-
Dendrelaphis_fuliginosus	-	-	KX660512	KX660638	-
Dendrelaphis_haasi	-	-	KX660493	KX660622	-
Dendrelaphis_marenae	-	-	KX660514	KX660640	-
Dendrelaphis_ngansonensis	-	KX660158	-	KX660561	-
Dendrelaphis_pictus	-	-	JX678825	U49304	-
Dendrelaphis_proarchos	-	-	KX660455	KX660581	-
Dendrelaphis_striatus	-	-	KX660495	KX660624	-
Dendrelaphis_subocularis	-	-	KX660494	KX660623	-
Elaphe_bimaculata	AY122767	-	DQ902104	DQ902283	DQ902210
Elaphe_cantoris	**MW114843**	-	-	-	-
Elaphe_cantoris	AY122769	-	DQ902135	DQ902315	DQ902246
Elaphe_carinata	AY122839	HM439983	DQ902133	DQ902284	DQ902211

Elaphe_climacophora	AY122772	-	DQ902105	DQ902285	DQ902212
Elaphe_davidi	AY122775	-	-	-	-
Elaphe_dione	AF236673	-	DQ902107	DQ902287	DQ902214
Elaphe_hodgsoni	-	-	DQ902136	DQ902318	DQ902247
Elaphe_moellendorffi	AY122786	-	DQ902116	DQ902295	DQ902223
Elaphe_quadrivirgata	AY122793	-	DQ902120	DQ902300	DQ902228
Elaphe_quatuorlineata	AY122796	AF215267	AY486931	AY487067	AY487028
Elaphe_sauromates	AY122797	-	-	-	-
Elaphe_schrenckii	AF236672	-	JQ798790	DQ902302	DQ902233
Elaphe_taeniura	AY122807	HM439981	EF076709	EF076708	EF076707
Gonyosoma_boulengeri	-	-	AF471053	-	-
Gonyosoma_frenatum	-	-	DQ902110	DQ902290	DQ902217
Gonyosoma_jansenii	AY122671	-	DQ902113	DQ902313	DQ902220
Gonyosoma_oxycephalum	AY122678	KX694646	KX694870	DQ902309	DQ902241
Gonyosoma_prasinum	-	-	DQ902119	DQ902299	DQ902227
Hebius_craspedogaster	-	-	JQ687429	JQ687412	JQ687459
Hebius_parallelum	-	-	**MW111476**	-	-
Herpetoreas_burbrinki	-	-	JQ687443	JQ687418	JQ687459
Herpetoreas_platyceps	-	-	**MW111464**	-	-
Hypsiglena_torquata	NC_013992	EU728591	AF471038	U49309	EU728591
Lycodon_alcalai	-	-	KC010344	-	-
Lycodon_aulicus	-	-	HQ735416	-	-
Lycodon_bibonius	-	-	KC010351	-	-
Lycodon_butleri	-	-	KC010353	-	-
Lycodon_carinatus	-	KC347352	KC347468	-	-
Lycodon_cavernicolus	-	-	KJ607890	-	-
Lycodon_fasciatus	-	-	**MW111467**	-	-
Lycodon_fasciatus	-	-	**MW111468**	-	-
Lycodon_septentrionalis	-	-	**MW111469**	-	-
Lycodon_chrysoprateros	-	-	KC010360	-	-
Lycodon_dumerilii	-	-	KC010361	-	-
Lycodon_effraenis	-	-	KC010376	-	-
Lycodon_meridionalis	-	-	**MW111470**	-	-
Lycodon_futsingensis	-	-	KC733209	-	-
Lycodon_gongshan	-	-	KP901024	-	-
Lycodon_jara	-	-	KC010367	-	-
Lycodon_laoensis	Z46455	Z46485	KC010368	-	-
Lycodon_muelleri	-	-	KC010373	-	-
Lycodon_multizonatus	-	-	KF732926	-	-
Lycodon_paucifasciatus	-	EU999213	-	-	-
Lycodon_rufozonatus	-	-	KC733194	-	-
Lycodon_ruhstrati	-	EU999209	KC010381	-	-
Lycodon_stormi	-	-	KC010380	-	-
Lycodon_subcinctus	-	-	KC733203	-	-

Lycodon_synaptor	-	-	**MW111471**	-	-
Lycodon_zawi	-	-	AF471040	-	-
Oligodon_arnensis	KC347327	KC347365	KC347481	KC347518	-
Oligodon_calamarius	KC347328	KC347329	-	-	
Oligodon_ornatus	**MW133293**	**MW090136**	-	-	
Oligodon_chinensis	HM591524	HM591527	-	-	-
Oligodon_cinereus	HM591503	HM591508	AF471033	-	-
Oligodon_cruentatus	HM591517	HM591517	-	-	-
Oligodon_cyclurus	HM591535	HM591536	-	-	-
Oligodon_fasciolatus	**MW133294**	**MW090137**	-	-	-
Oligodon_formosanus	HM591532	HM591533	-	-	-
Oligodon_lacroixi	**MW133295**	**MW090138**	-	-	-
Oligodon_lipipengi	**MW133296**	**MW090139**	-	-	-
Oligodon_maculatus	HM591511	HM591511	-	-	-
Oligodon_modestus	HM591498	-	-	-	-
Oligodon_nagao	**MW133297**	**MW090140**	-	-	-
Oligodon_ocellatus	**MW133298**	**MW090141**	-	-	-
Oligodon_octolineatus	HM591519	HM591519	-	-	-
Oligodon_planiceps	HM591514	HM591514	-	-	-
Oligodon_sp	**MW133299**	**MW090142**	-	-	-
Oligodon_splendidus	HM591510	HM591510	-	-	-
Oligodon_sublineatus	KC347329	KC347367	KC347483	KC347520	-
Oligodon_taeniatus	HM591520	HM591522	-	-	-
Oligodon_theobaldi	HM591516	HM591516	-	-	-
Oligodon_torquatus	HM591513	HM591513	-	-	-
Oligodon_venustus_	HM591500	-	-	-	-
Oreocryptophis_porphyraceus	NC_012770	NC_012770	DQ902118	DQ902298	NC_012770
Pseudoxenodon_bambusicola	JQ598833	JQ598893	-	-	-
Pseudoxenodon_karlschmidti	KX694578	KX694676	KX694893	-	-
Pseudoxenodon_macrops	JQ598835	JQ598895	JQ598949	-	-
Pseudoxenodon_stejnegeri	-	**MW090133**	-	-	-
Ptyas_carinata	-	KX660238	-	-	-
Ptyas_dhumnades	**MW133300**	**MW090134**	-	-	-
Ptyas_fusca	-	KX660202	-	-	-
Ptyas_korros	KX694591	KX694663	KX694869	AY487062	AY487023
Ptyas_luzonensis	-	KX660251	-	-	-
Ptyas_mucosa	AY122828	FJ907950	AF471054	AY487063	AY487024
Ptyas_nigromarginata	**MW133301**	**MW090135**	-	-	-
Rhabdophis_adleri	-	-	**MW111465**	-	-
Rhabdophis_guangdongensis	-	-	KF800930	-	-
Rhabdophis_himalayanus	-	-	-	-	-
Rhabdophis_leonardi	-	-	**MW111472**	-	-
Rhabdophis_nigrocinctus	-	-	**MW111473**	-	-
Rhabdophis_nuchalis	AF236678	-	GQ281786	JQ687413	JQ687454

Rhabdophis_pentasupralabialis	-	-	KF800934	-	-
Rhabdophis_subminiatus	AF544776	AF544805	JQ598951	JQ687411	-
Rhabdophis_swinhonis	-	-	AB842176	-	-
Rhabdophis_tigrinus	AF236679	-	GQ281785	JQ687419	JQ687460
Sibynophis_bistrigatus	-	-	KC000127	-	KC000130
Sibynophis_chinensis	-	-	KC000124	-	KC000131
Sibynophis_collaris	KX694581	KX694665	KC000121	JN211315	KC000133
Sibynophis_triangularis	-	-	KC000123	-	KC000132
Thamnophis_sirtalis	KX694574	KX694670	AF420193	AF420196	DQ995365
Thermophis_baileyi	-	-	EU496919	JN564723	JN559862
Thermophis_zhaoermii	GQ166168	GQ166168	GQ166168	GQ166168	GQ166168
Trachischium_guentheri	-	-	MN306288	-	-
Trachischium_monticola	-	-	MN306290	-	-
Trachischium_tenuiceps	-	-	MN306289	-	-
Xenochrophis_flavipunctatus	AF544780	AF544809	-	-	FJ416748
Xenochrophis_piscator	GQ225679	-	GQ225659	-	-
Xenochrophis_punctulatus	-	-	AF471079	AY487074	AY487035
Xenochrophis_schnurrenbergeri	GQ225678	-	GQ225660	-	-
**Outgroup**					
Achalinus_meiguensis	FJ424614	FJ424614	FJ424614	FJ424614	FJ424614
Achalinus_rufescens	KX694570	KX694613	KX694895	U49319	-
Acrochordus_granulatus	AB177879	NC007400	NC_007400	NC_007400	NC_007400
Acrochordus_javanicus	KX694587	KX694614	KX694897	HM234055	-
Anilius_scytale	FJ755180	NC_014343	FJ755180	FJ755180	NC_014343
Azemiops_feae	KX694579	KX694684	KX694840	AY352808	-
Bitis_arietans	KX694571	KX694619	KX694845	EU852304	JX073288
Boa_constrictor	NC_007398	NC_007398	AF471036	NC_007398	NC_007398
Broghammerus_reticulatus	Z46448	-	FJ717481	-	-
Cantoria_violacea	KX694595	KX694627	KX694852	-	-
Cerberus_rynchops	KX694596	KX694630	KX694850	U49327	-
Crotalus_cerastes	AF259235	AF259128	U69773	JN620962	AY016245
Cylindrophis_ruffus	NC_007401	NC_007401	NC_007401	NC_007401	NC_007401
Deinagkistrodon_acutus	KX694593	KX694683	KX694847	AY352811	DQ343647
Eryx_colubrinus	AF544747	-	U69811	-	-
Eunectes_notaeus	AM236347	-	AM236347	AM236347	AM236347
Indotyphlops_braminus	DQ343649	NC_010196	DQ343649	NC_010196	NC_010196
Loxocemus_bicolor	Z46456	-	AY099993	-	-
Micrurus_surinamensis	AF544770	AF544799	EF137415	EF137407	-
Naja_atra	KX694544	KX694689	KX694859	EU921898	EU921898
Psammophis_mossambicus	KX694547	KX694660	KX694878	DQ486283	-
Sistrurus_catenatus	DQ464268	AF259119	AY223610	HQ257759	GQ359800
Vipera_ammodytes	EU624266	EU624297	AM944799	EU624232	AM944799
Xenopeltis_unicolor	NC_007402	NC_007402	NC_007402	NC_007402	NC_007402

**Species**	**C-mos**

Ahaetulla_fronticincta	AF471161
Ahaetulla_nasuta	KC347377
Ahaetulla_prasina	KX694801
Ahaetulla_pulverulenta	KC347378
Amphiesma_stolatum	JQ687450
Archelaphe_bella	DQ902097
Atretium_yunnanensis	JQ687448
Boiga_barnesii	KC347385
Boiga_beddomei	KC347386
Boiga_ceylonensis	KC347387
Boiga_cynodon	-
Boiga_forsteni	KC347388
Boiga_multomaculata	FJ710797
Boiga_trigonata	KC347389
Calamaria_pavimentata	KX694804
Calamaria_yunnanensis	KX694805
Chrysopelea_ornata	KC347393
Chrysopelea_taprobanica	KC347394
Coelognathus_erythrurus	DQ902067
Coelognathus_flavolineatus	DQ902090
Coelognathus_helena	DQ902071
Coelognathus_radiatus	DQ902079
Coelognathus_subradiatus	DQ902084
Coluber_constrictor	KX694806
Dendrelaphis_bifrenalis	KC347397
Dendrelaphis_cyanochloris	**MW111502**
Dendrelaphis_cyanochloris	KX660320
Dendrelaphis_caudolineatus	GQ895808
Dendrelaphis_formosus	KX660301
Dendrelaphis_fuliginosus	KX660383
Dendrelaphis_haasi	KX660365
Dendrelaphis_marenae	KX660385
Dendrelaphis_ngansonensis	KX660297
Dendrelaphis_pictus	-
Dendrelaphis_proarchos	KX660321
Dendrelaphis_striatus	KX660367
Dendrelaphis_subocularis	KX660366
Elaphe_bimaculata	DQ902062
Elaphe_cantoris	-
Elaphe_cantoris	DQ902095
Elaphe_carinata	DQ902063
Elaphe_climacophora	DQ902064
Elaphe_davidi	-
Elaphe_dione	DQ902066

Elaphe_hodgsoni	DQ902096
Elaphe_moellendorffi	DQ902074
Elaphe_quadrivirgata	DQ902078
Elaphe_quatuorlineata	AY486955
Elaphe_sauromates	-
Elaphe_schrenckii	DQ902082
Elaphe_taeniura	EF076705
Gonyosoma_boulengeri	AF471153
Gonyosoma_frenatum	DQ902069
Gonyosoma_jansenii	DQ902100
Gonyosoma_oxycephalum	KX694808
Gonyosoma_prasinum	DQ902077
Hebius_craspedogaster	JQ687437
Hebius_parallelum	**MW111503**
Herpetoreas_burbrinki	GQ281781
Herpetoreas_platyceps	**MW111514**
Hypsiglena_torquata	AF471159
Lycodon_alcalai	KC010303
Lycodon_aulicus	HQ735418
Lycodon_bibonius	KC010309
Lycodon_butleri	KC010311
Lycodon_carinatus	KC347392
Lycodon_cavernicolus	-
Lycodon_fasciatus	**MW111504**
Lycodon_fasciatus	**MW111505**
Lycodon_septentrionalis	**MW111506**
Lycodon_chrysoprateros	KC010318
Lycodon_dumerilii	KC010319
Lycodon_effraenis	KC010328
Lycodon_meridionalis	**MW111507**
Lycodon_futsingensis	KC733225
Lycodon_gongshan	KP901016
Lycodon_jara	KC010322
Lycodon_laoensis	KC010323
Lycodon_muelleri	KC010326
Lycodon_multizonatus	KF732933
Lycodon_paucifasciatus	-
Lycodon_rufozonatus	KC733211
Lycodon_ruhstrati	KC010332
Lycodon_stormi	KC010331
Lycodon_subcinctus	KC733220
Lycodon_synaptor	**MW111508**
Lycodon_zawi	AF471111
Oligodon_arnensis	KC347405

Oligodon_calamarius	-
Oligodon_ornatus	-
Oligodon_chinensis	-
Oligodon_cinereus	AF471101
Oligodon_cruentatus	-
Oligodon_cyclurus	-
Oligodon_fasciolatus	-
Oligodon_formosanus	-
Oligodon_lacroixi	-
Oligodon_lipipengi	-
Oligodon_maculatus	-
Oligodon_modestus	-
Oligodon_nagao	-
Oligodon_ocellatus	-
Oligodon_octolineatus	-
Oligodon_planiceps	-
Oligodon_sp	-
Oligodon_splendidus	-
Oligodon_sublineatus	KC347407
Oligodon_taeniatus	-
Oligodon_theobaldi	-
Oligodon_torquatus	-
Oligodon_venustus_	-
Oreocryptophis_porphyraceus	DQ902076
Pseudoxenodon_bambusicola	JQ598996
Pseudoxenodon_karlschmidti	KX694816
Pseudoxenodon_macrops	-
Pseudoxenodon_stejnegeri	**MW111492**
Ptyas_carinata	KX660379
Ptyas_dhumnades	**MW111493**
Ptyas_fusca	KX660342
Ptyas_korros	KX694817
Ptyas_luzonensis	KX660392
Ptyas_mucosa	AF471151
Ptyas_nigromarginata	**MW111494**
Rhabdophis_adleri	**MW111495**
Rhabdophis_guangdongensis	KF800920
Rhabdophis_himalayanus	**MW111496**
Rhabdophis_leonardi	**MW111497**
Rhabdophis_nigrocinctus	**MW111498**
Rhabdophis_nuchalis	JQ687438
Rhabdophis_pentasupralabialis	KF800924
Rhabdophis_subminiatus	JQ687436
Rhabdophis_swinhonis	AB861888

Rhabdophis_tigrinus	JQ687444
Sibynophis_bistrigatus	KC000112
Sibynophis_chinensis	KC000113
Sibynophis_collaris	KX694818
Sibynophis_triangularis	KC000116
Thamnophis_sirtalis	DQ902094
Thermophis_baileyi	EU496922
Thermophis_zhaoermii	-
Trachischium_guentheri	**MW111499**
Trachischium_monticola	**MW111501**
Trachischium_tenuiceps	**MW111500**
Xenochrophis_flavipunctatus	AF544714
Xenochrophis_piscator	GQ225669
Xenochrophis_punctulatus	AF471106
Xenochrophis_schnurrenbergeri	GQ225668
**Outgroup**	
Achalinus_meiguensis	-
Achalinus_rufescens	-
Acrochordus_granulatus	KX694800
Acrochordus_javanicus	-
Anilius_scytale	AF544722
Azemiops_feae	KX694774
Bitis_arietans	-
Boa_constrictor	AF471115
Broghammerus_reticulatus	AF544675
Cantoria_violacea	EF395922
Cerberus_rynchops	KX694779
Crotalus_cerastes	JN620894
Cylindrophis_ruffus	AF471133
Deinagkistrodon_acutus	KX694782
Eryx_colubrinus	AF544716
Eunectes_notaeus	KX694783
Indotyphlops_braminus	AY099980
Loxocemus_bicolor	AF544730
Micrurus_surinamensis	EF137422
Naja_atra	KX694797
Psammophis_mossambicus	DQ486185
Sistrurus_catenatus	-
Vipera_ammodytes	-
Xenopeltis_unicolor	AY099977

Data S6.

Time trees with calibration dates indicated. The estimated dates and confidence limits for each corresponding event are presented in **Table S2** and **Table S3**. The red triangles indicate fossil calibrations, and black triangles indicate secondary calibrations (see **Table S5** for a list all calibrations and their citations).

Agamidae

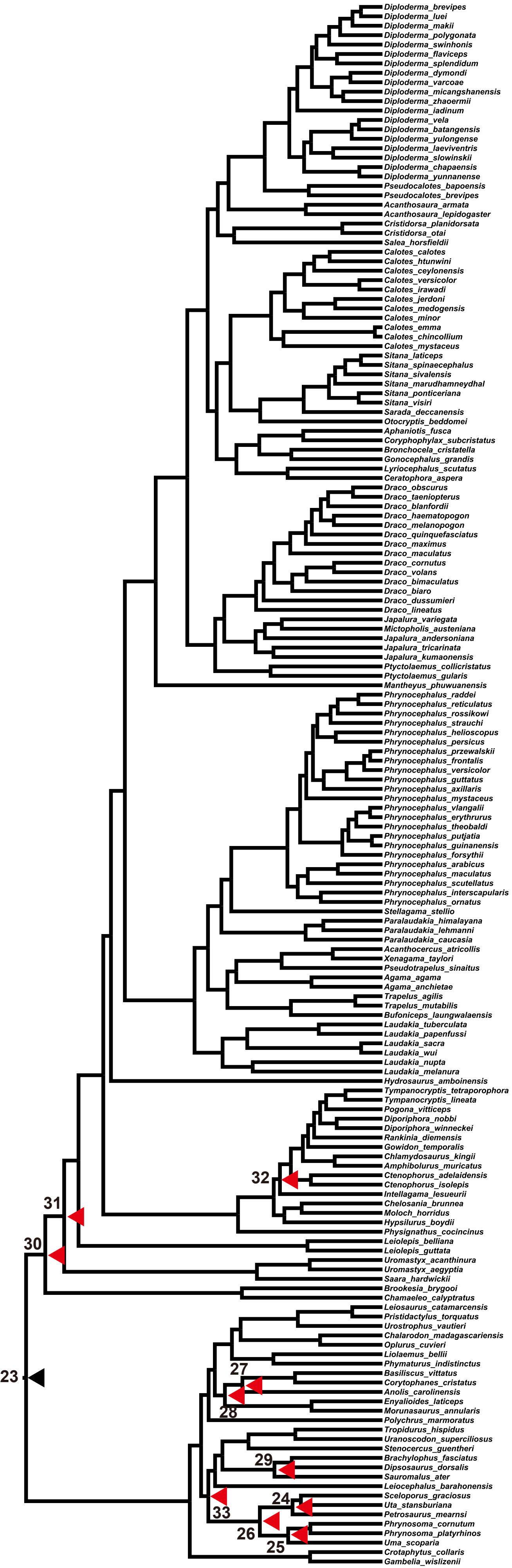
Diploderma_brevipes
Diploderma_luei
Diploderma_makii
Diploderma_polygonata
Diploderma_swinhonis
Diploderma_flaviceps
Diploderma_splendidum
Diploderma_dymondi
Diploderma_varcoae
Diploderma_micangshanensis
Diploderma_zhaoermii
Diploderma_iadinum
Diploderma_vela
Diploderma_batangensis
Diploderma_yulongense
Diploderma_laeviventris
Diploderma_slowinskii
Diploderma_chapaensis
Diploderma_yunnanense
Pseudocalotes_bapoensis
Pseudocalotes_brevipes
Acanthosaura_armata
Acanthosaura_lepidogaster
Cristidorsa_planidorsata
Cristidorsa_otai
Salea_horsfieldii
Calotes_calotes
Calotes_htunwini
Calotes_ceylonensis
Calotes_versicolor
Calotes_irawadi
Calotes_jerdoni
Calotes_medogensis
Calotes_minor
Calotes_emma
Calotes_chincollium
Calotes_mystaceus
Sitana_laticeps
Sitana_spinaecephalus
Sitana_sivalensis
Sitana_marudhamneydhal
Sitana_ponticeriana
Sitana_visiri
Sarada_deccanensis
Otocryptis_beddomei
Aphaniotis_fusca
Coryphophylax_subcristatus
Bronchocela_cristatella
Gonocephalus_grandis
Lyriocephalus_scutatus
Ceratophora_aspera
Draco_obscurus
Draco_taeniopterus
Draco_blanfordii
Draco_haematopogon
Draco_melanopogon
Draco_quinquefasciatus
Draco_maximus
Draco_maculatus
Draco_cornutus
Draco_volans
Draco_bimaculatus
Draco_biaro
Draco_dussumieri
Draco_lineatus
Japalura_variegata
Mictopholis_austeniana
Japalura_andersoniana
Japalura_tricarinata
Japalura_kumaonensis
Ptyctolaemus_collicristatus
Ptyctolaemus_gularis
Mantheyus_phuwuanensis
Phrynocephalus_raddei
Phrynocephalus_reticulatus
Phrynocephalus_rossikowi
Phrynocephalus_strauchi
Phrynocephalus_helioscopus
Phrynocephalus_persicus
Phrynocephalus_przewalskii
Phrynocephalus_frontalis
Phrynocephalus_versicolor
Phrynocephalus_guttatus
Phrynocephalus_axillaris
Phrynocephalus_mystaceus
Phrynocephalus_vlangalii
Phrynocephalus_erythrurus
Phrynocephalus_theobaldi
Phrynocephalus_putjatia
Phrynocephalus_guinanensis
Phrynocephalus_forsythii
Phrynocephalus_arabicus
Phrynocephalus_maculatus
Phrynocephalus_scutellatus
Phrynocephalus_interscapularis
Phrynocephalus_ornatus
Stellagama_stellio
Paralaudakia_himalayana
Paralaudakia_lehmanni
Paralaudakia_caucasia
Acanthocercus_atricollis
Xenagama_taylori
Pseudotrapelus_sinaitus
Agama_agama
Agama_anchietae
Trapelus_agilis
Trapelus_mutabilis
Bufoniceps_laungwalaensis
Laudakia_tuberculata
Laudakia_papenfussi
Laudakia_sacra
Laudakia_wui
Laudakia_nupta
Laudakia_melanura
Hydrosaurus_amboinensis
Tympanocryptis_tetraporophora
Tympanocryptis_lineata
Pogona_vitticeps
Diporiphora_nobbi
Diporiphora_winneckei
Rankinia_diemensis
Gowidon_temporalis
Chlamydosaurus_kingii
Amphibolurus_muricatus
32
Ctenophorus_adelaidensis
Ctenophorus_isolepis
Intellagama_lesueurii
Chelosania_brunnea
Moloch_horridus
Hypsilurus_boydii
Physignathus_cocincinus
31
Leiolepis_belliana
Leiolepis_guttata
30
Uromastyx_acanthinura
Uromastyx_aegyptia
Saara_hardwickii
Brookesia_brygooi
Chamaeleo_calyptratus
Leiosaurus_catamarcensis
Pristidactylus_torquatus
Urostrophus_vautieri
Chalarodon_madagascariensis
Oplurus_cuvieri
Liolaemus_bellii
Phymaturus_indistinctus
23
27
Basiliscus_vittatus
Corytophanes_cristatus
Anolis_carolinensis
28
Enyalioides_laticeps
Morunasaurus_annularis
Polychrus_marmoratus
Tropidurus_hispidus
Uranoscodon_superciliosus
Stenocercus_guentheri
29
Brachylophus_fasciatus
Dipsosaurus_dorsalis
Sauromalus_ater
Leiocephalus_barahonensis
24
Sceloporus_graciosus
Uta_stansburiana
33
Petrosaurus_mearnsi
26
Phrynosoma_cornutum
Phrynosoma_platyrhinos
25
Uma_scoparia
Crotaphytus_collaris
Gambelia_wislizenii

Anguidae

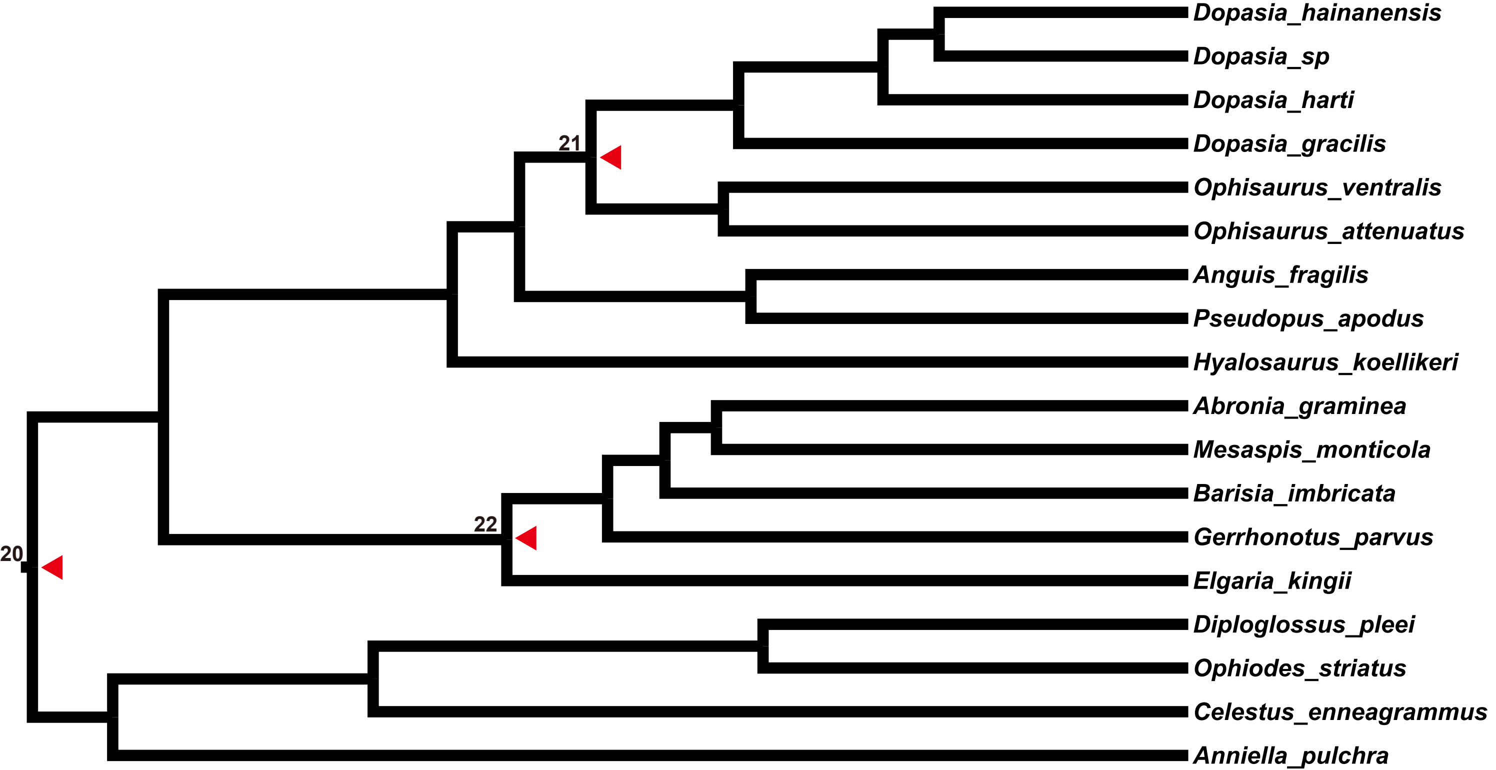

Dopasia_hainanensis
Dopasia_sp
Dopasia_harti
Dopasia_gracilis
Ophisaurus_ventralis
Ophisaurus_attenuatus
Anguis_fragilis
Pseudopus_apodus
Hyalosaurus_koellikeri
Abronia_graminea
Mesaspis_monticola
Barisia_imbricata
Gerrhonotus_parvus
Elgaria_kingii
Diploglossus_pleei
Ophiodes_striatus
Celestus_enneagrammus
Anniella_pulchra
21
22
20

Bufonidae

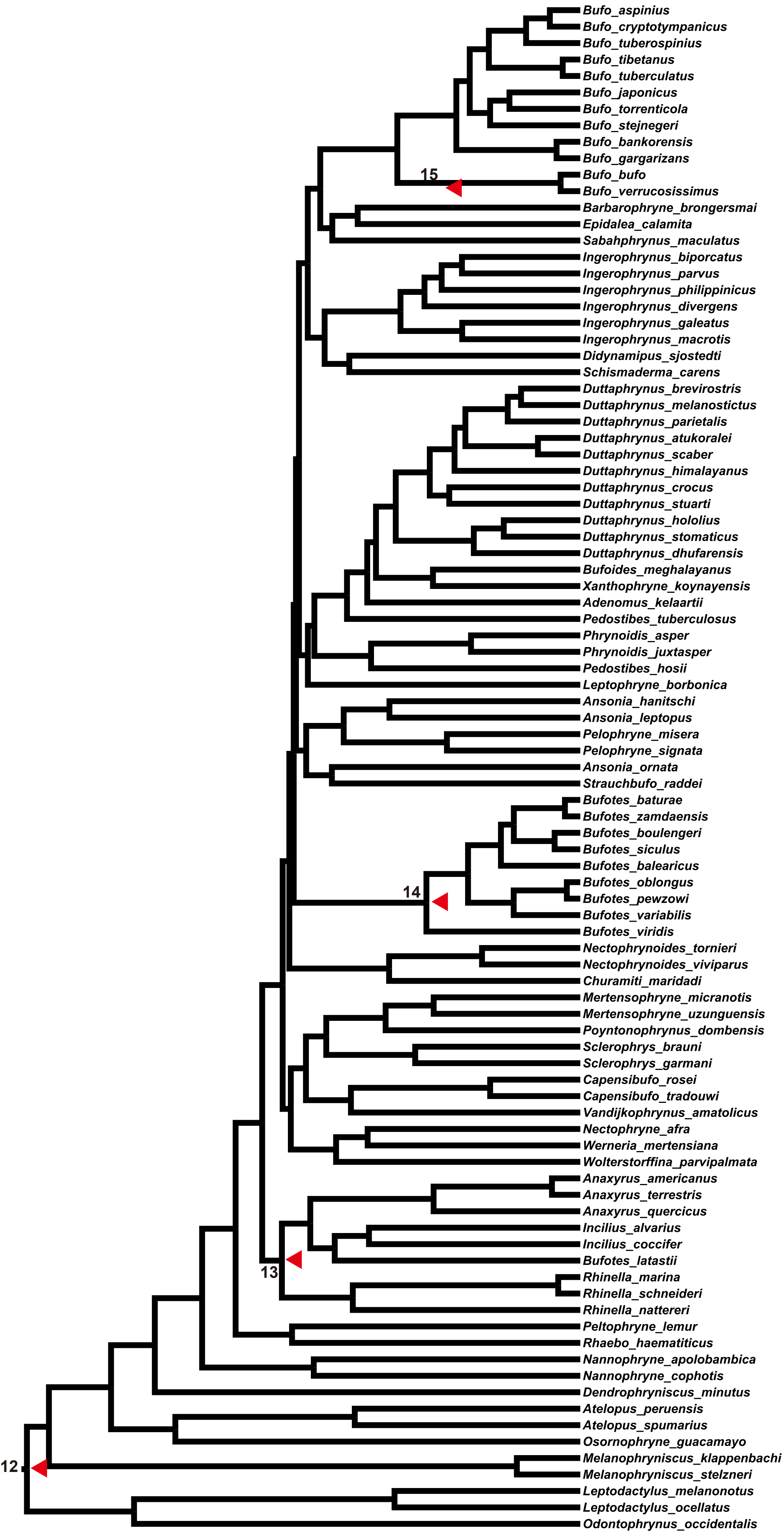
Bufo_aspinius
Bufo_cryptotympanicus
Bufo_tuberospinius
Bufo_tibetanus
Bufo_tuberculatus
Bufo_japonicus
Bufo_torrenticola
Bufo_stejnegeri
Bufo_bankorensis
Bufo_gargarizans
15
Bufo_bufo
Bufo_verrucosissimus
Barbarophryne_brongersmai
Epidalea_calamita
Sabahphrynus_maculatus
Ingerophrynus_biporcatus
Ingerophrynus_parvus
Ingerophrynus_philippinicus
Ingerophrynus_divergens
Ingerophrynus_galeatus
Ingerophrynus_macrotis
Didynamipus_sjostedti
Schismaderma_carens
Duttaphrynus_brevirostris
Duttaphrynus_melanostictus
Duttaphrynus_parietalis
Duttaphrynus_atukoralei
Duttaphrynus_scaber
Duttaphrynus_himalayanus
Duttaphrynus_crocus
Duttaphrynus_stuarti
Duttaphrynus_hololius
Duttaphrynus_stomaticus
Duttaphrynus_dhufarensis
Bufoides_meghalayanus
Xanthophryne_koynayensis
Adenomus_kelaartii
Pedostibes_tuberculosus
Phrynoidis_asper
Phrynoidis_juxtasper
Pedostibes_hosii
Leptophryne_borbonica
Ansonia_hanitschi
Ansonia_leptopus
Pelophryne_misera
Pelophryne_signata
Ansonia_ornata
Strauchbufo_raddei
Bufotes_baturae
Bufotes_zamdaensis
Bufotes_boulengeri
Bufotes_siculus
Bufotes_balearicus
14
Bufotes_oblongus
Bufotes_pewzowi
Bufotes_variabilis
Bufotes_viridis
Nectophrynoides_tornieri
Nectophrynoides_viviparus
Churamiti_maridadi
Mertensophryne_micranotis
Mertensophryne_uzunguensis
Poyntonophrynus_dombensis
Sclerophrys_brauni
Sclerophrys_garmani
Capensibufo_rosei
Capensibufo_tradouwi
Vandijkophrynus_amatolicus
Nectophryne_afra
Werneria_mertensiana
Wolterstorffina_parvipalmata
Anaxyrus_americanus
Anaxyrus_terrestris
Anaxyrus_quercicus
Incilius_alvarius
Incilius_coccifer
Bufotes_latastii
13
Rhinella_marina
Rhinella_schneideri
Rhinella_nattereri
Peltophryne_lemur
Rhaebo_haematiticus
Nannophryne_apolobambica
Nannophryne_cophotis
Dendrophryniscus_minutus
Atelopus_peruensis
Atelopus_spumarius
Osornophryne_guacamayo
12
Melanophryniscus_klappenbachi
Melanophryniscus_stelzneri
Leptodactylus_melanonotus
Leptodactylus_ocellatus
Odontophrynus_occidentalis

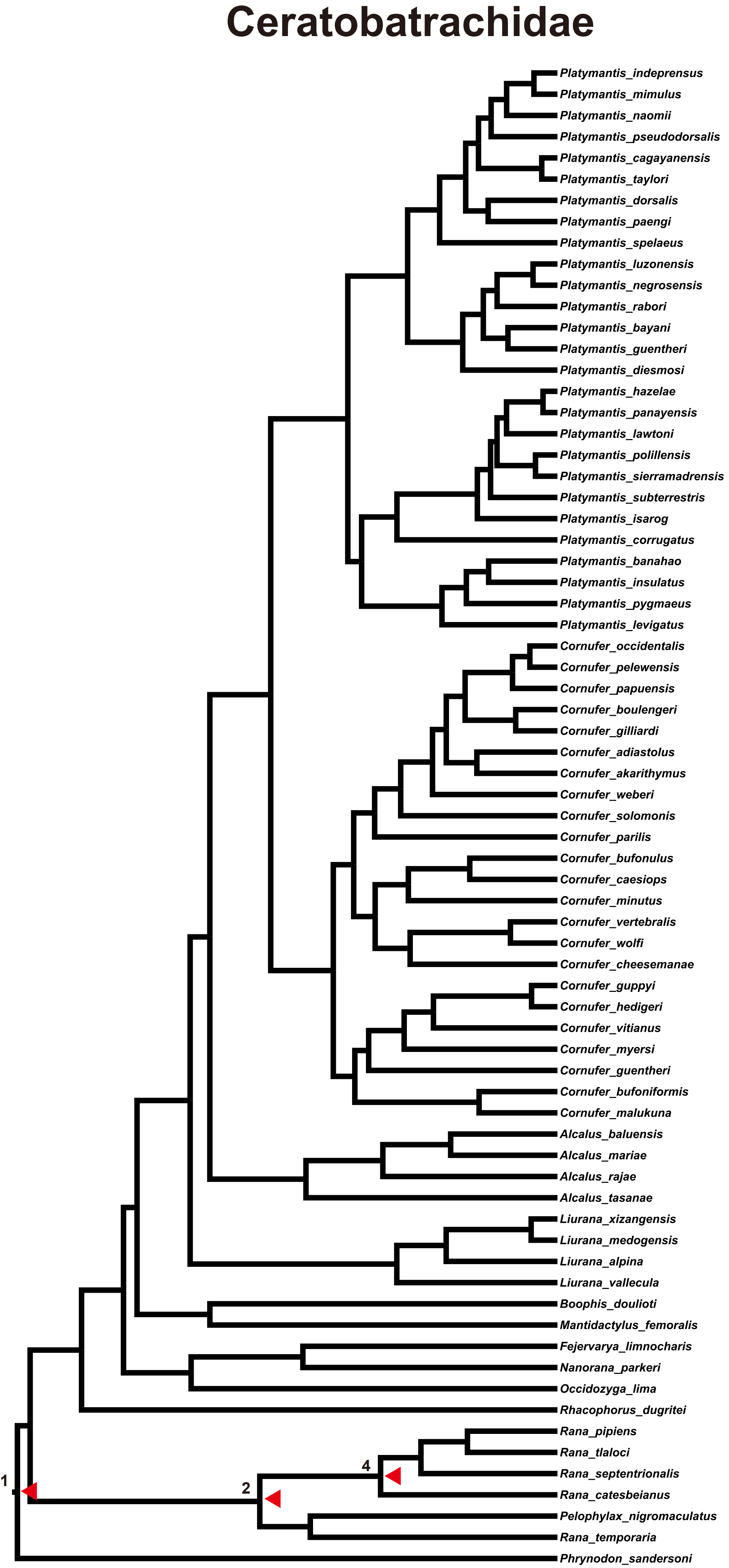
Ceratobatrachidae
Platymantis_indeprensus
Platymantis_mimulus
Platymantis_naomii
Platymantis_pseudodorsalis
Platymantis_cagayanensis
Platymantis_taylori
Platymantis_dorsalis
Platymantis_paengi
Platymantis_spelaeus
Platymantis_luzonensis
Platymantis_negrosensis
Platymantis_rabori
Platymantis_bayani
Platymantis_guentheri
Platymantis_diesmosi
Platymantis_hazelae
Platymantis_panayensis
Platymantis_lawtoni
Platymantis_polillensis
Platymantis_sierramadrensis
Platymantis_subterrestris
Platymantis_isarog
Platymantis_corrugatus
Platymantis_banahao
Platymantis_insulatus
Platymantis_pygmaeus
Platymantis_levigatus
Cornufer_occidentalis
Cornufer_pelewensis
Cornufer_papuensis
Cornufer_boulengeri
Cornufer_gilliardi
Cornufer_adiastolus
Cornufer_akarithymus
Cornufer_weberi
Cornufer_solomonis
Cornufer_parilis
Cornufer_bufonulus
Cornufer_caesiops
Cornufer_minutus
Cornufer_vertebralis
Cornufer_wolfi
Cornufer_cheesemanae
Cornufer_guppyi
Cornufer_hedigeri
Cornufer_vitianus
Cornufer_myersi
Cornufer_guentheri
Cornufer_bufoniformis
Cornufer_malukuna
Alcalus_baluensis
Alcalus_mariae
Alcalus_rajae
Alcalus_tasanae
Liurana_xizangensis
Liurana_medogensis
Liurana_alpina
Liurana_vallecula
Boophis_doulioti
Mantidactylus_femoralis
Fejervarya_limnocharis
Nanorana_parkeri
Occidozyga_lima
Rhacophorus_dugritei
Rana_pipiens
Rana_tlaloci
Rana_septentrionalis
Rana_catesbeianus
Pelophylax_nigromaculatus
Rana_temporaria
Phrynodon_sandersoni
1
2
4

Colubridae

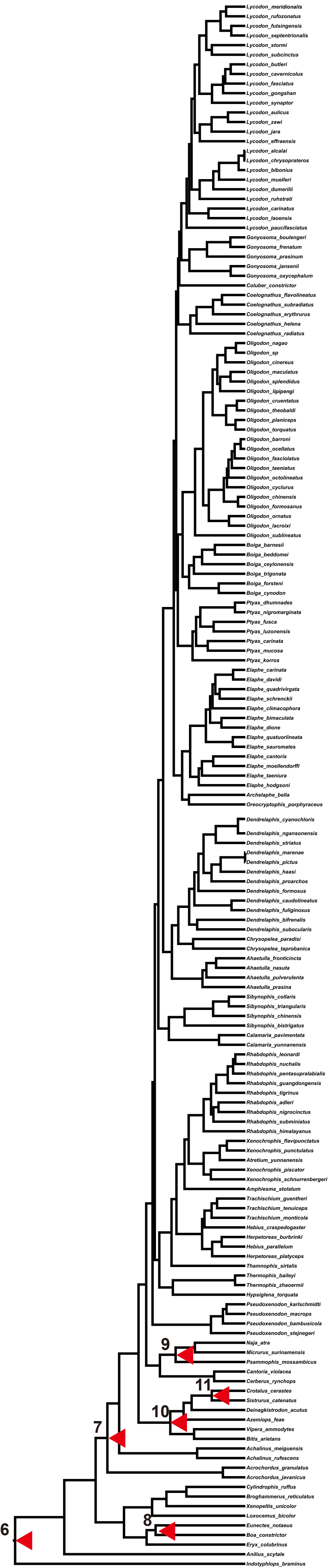

Lycodon_meridionalis
Lycodon_rufozonatus
Lycodon_futsingensis
Lycodon_septentrionalis
Lycodon_stormi
Lycodon_subcinctus
Lycodon_butleri
Lycodon_cavernicolus
Lycodon_fasciatus
Lycodon_gongshan
Lycodon_synaptor
Lycodon_aulicus
Lycodon_zawi
Lycodon_jara
Lycodon_effraensis
Lycodon_alcalai
Lycodon_chrysoprateros
Lycodon_bibonius
Lycodon_muelleri
Lycodon_dumerilii
Lycodon_ruhstrati
Lycodon_carinatus
Lycodon_laoensis
Lycodon_paucifasciatus
Gonyosoma_boulengeri
Gonyosoma_frenatum
Gonyosoma_prasinum
Gonyosoma_jansenii
Gonyosoma_oxycephalum
Coluber_constrictor
Coelognathus_flavolineatus
Coelognathus_subradiatus
Coelognathus_erythrurus
Coelognathus_helena
Coelognathus_radiatus
Oligodon_nagao
Oligodon_sp
Oligodon_cinereus
Oligodon_maculatus
Oligodon_splendidus
Oligodon_lipipengi
Oligodon_cruentatus
Oligodon_theobaldi
Oligodon_planiceps
Oligodon_torquatus
Oligodon_barroni
Oligodon_ocellatus
Oligodon_fasciolatus
Oligodon_taeniatus
Oligodon_octolineatus
Oligodon_cyclurus
Oligodon_chinensis
Oligodon_formosanus
Oligodon_ornatus
Oligodon_lacroixi
Oligodon_sublineatus
Boiga_barnesii
Boiga_beddomei
Boiga_ceylonensis
Boiga_trigonata
Boiga_forsteni
Boiga_cynodon
Ptyas_dhumnades
Ptyas_nigromarginata
Ptyas_fusca
Ptyas_luzonensis
Ptyas_carinata
Ptyas_mucosa
Ptyas_korros
Elaphe_carinata
Elaphe_davidi
Elaphe_quadrivirgata
Elaphe_schrenckii
Elaphe_climacophora
Elaphe_bimaculata
Elaphe_dione
Elaphe_quatuorlineata
Elaphe_sauromates
Elaphe_cantoris
Elaphe_moellendorffi
Elaphe_taeniura
Elaphe_hodgsoni
Archelaphe_bella
Oreocryptophis_porphyraceus
Dendrelaphis_cyanochloris
Dendrelaphis_ngansonensis
Dendrelaphis_striatus
Dendrelaphis_marenae
Dendrelaphis_pictus
Dendrelaphis_haasi
Dendrelaphis_proarchos
Dendrelaphis_formosus
Dendrelaphis_caudolineatus
Dendrelaphis_fuliginosus
Dendrelaphis_bifrenalis
Dendrelaphis_subocularis
Chrysopelea_paradisi
Chrysopelea_taprobanica
Ahaetulla_fronticincta
Ahaetulla_nasuta
Ahaetulla_pulverulenta
Ahaetulla_prasina
Sibynophis_collaris
Sibynophis_triangularis
Sibynophis_chinensis
Sibynophis_bistrigatus
Calamaria_pavimentata
Calamaria_yunnanensis
Rhabdophis_leonardi
Rhabdophis_nuchalis
Rhabdophis_pentasupralabialis
Rhabdophis_guangdongensis
Rhabdophis_tigrinus
Rhabdophis_adleri
Rhabdophis_nigrocinctus
Rhabdophis_subminiatus
Rhabdophis_himalayanus
Xenochrophis_flavipunctatus
Xenochrophis_punctulatus
Atretium_yunnanensis
Xenochrophis_piscator
Xenochrophis_schnurrenbergeri
Amphiesma_stolatum
Trachischium_guentheri
Trachischium_tenuiceps
Trachischium_monticola
Hebius_craspedogaster
Herpetoreas_burbrinki
Hebius_parallelum
Herpetoreas_platyceps
Thamnophis_sirtalis
Thermophis_baileyi
Thermophis_zhaoermii
Hypsiglena_torquata
Pseudoxenodon_karlschmidti
Pseudoxenodon_macrops
Pseudoxenodon_bambusicola
Pseudoxenodon_stejnegeri
9
Naja_atra
Micrurus_surinamensis
Psammophis_mossambicus
Cantoria_violacea
Cerberus_rynchops
11
Crotalus_cerastes
Sistrurus_catenatus
10
Deinagkistrodon_acutus
Azemiops_feae
Vipera_ammodytes
Bitis_arietans
7
Achalinus_meiguensis
Achalinus_rufescens
Acrochordus_granulatus
Acrochordus_javanicus
Cylindrophis_ruffus
Broghammerus_reticulatus
Xenopeltis_unicolor
Loxocemus_bicolor
8
Eunectes_notaeus
Boa_constrictor
Eryx_colubrinus
6
Anilius_scytale
Indotyphlops_braminus

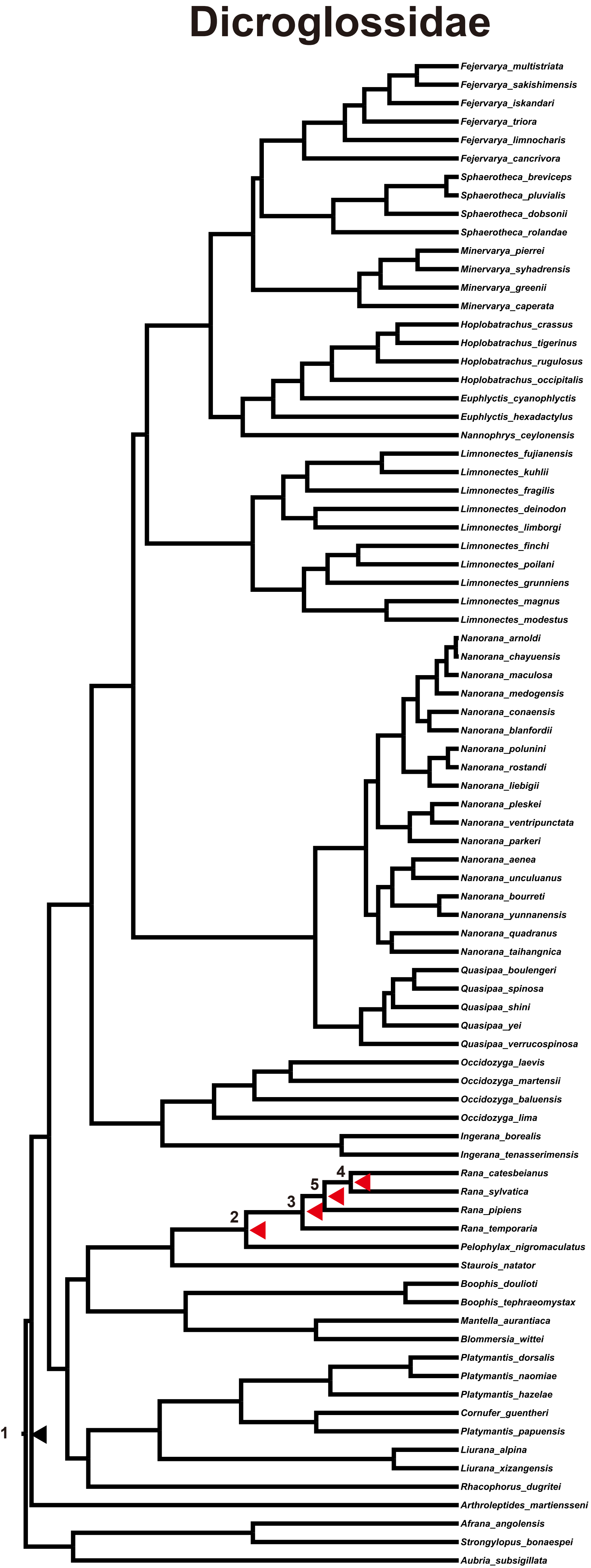
Dicroglossidae
Fejervarya_multistriata
Fejervarya_sakishimensis
Fejervarya_iskandari
Fejervarya_triora
Fejervarya_limnocharis
Fejervarya_cancrivora
Sphaerotheca_breviceps
Sphaerotheca_pluvialis
Sphaerotheca_dobsonii
Sphaerotheca_rolandae
Minervarya_pierrei
Minervarya_syhadrensis
Minervarya_greenii
Minervarya_caperata
Hoplobatrachus_crassus
Hoplobatrachus_tigerinus
Hoplobatrachus_rugulosus
Hoplobatrachus_occipitalis
Euphlyctis_cyanophlyctis
Euphlyctis_hexadactylus
Nannophrys_ceylonensis
Limnonectes_fujianensis
Limnonectes_kuhlii
Limnonectes_fragilis
Limnonectes_deinodon
Limnonectes_limborgi
Limnonectes_finchi
Limnonectes_poilani
Limnonectes_grunniens
Limnonectes_magnus
Limnonectes_modestus
Nanorana_arnoldi
Nanorana_chayuensis
Nanorana_maculosa
Nanorana_medogensis
Nanorana_conaensis
Nanorana_blanfordii
Nanorana_polunini
Nanorana_rostandi
Nanorana_liebigii
Nanorana_pleskei
Nanorana_ventripunctata
Nanorana_parkeri
Nanorana_aenea
Nanorana_unculuanus
Nanorana_bourreti
Nanorana_yunnanensis
Nanorana_quadranus
Nanorana_taihangnica
Quasipaa_boulengeri
Quasipaa_spinosa
Quasipaa_shini
Quasipaa_yei
Quasipaa_verrucospinosa
Occidozyga_laevis
Occidozyga_martensii
Occidozyga_baluensis
Occidozyga_lima
Ingerana_borealis
Ingerana_tenasserimensis
4
5
3
2
Rana_catesbeianus
Rana_sylvatica
Rana_pipiens
Rana_temporaria
Pelophylax_nigromaculatus
Staurois_natator
Boophis_doulioti
Boophis_tephraeomystax
Mantella_aurantiaca
Blommersia_wittei
Platymantis_dorsalis
Platymantis_naomiae
Platymantis_hazelae
Cornufer_guentheri
Platymantis_papuensis
1
Liurana_alpina
Liurana_xizangensis
Rhacophorus_dugritei
Arthroleptides_martiensseni
Afrana_angolensis
Strongylopus_bonaespei
Aubria_subsigillata

Elapidae

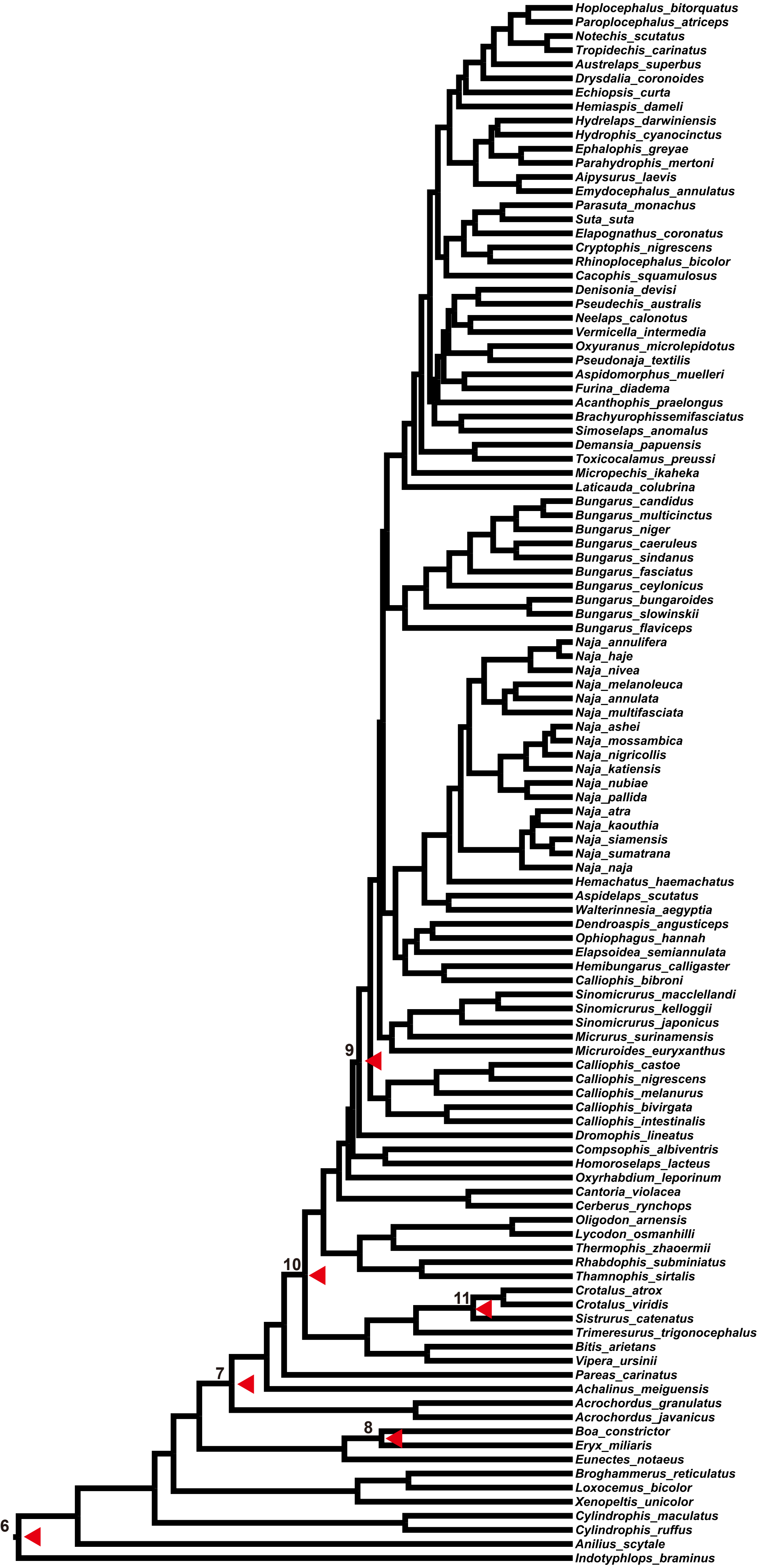
Hoplocephalus_bitorquatus
Paroplocephalus_atriceps
Notechis_scutatus
Tropidechis_carinatus
Austrelaps_superbus
Drysdalia_coronoides
Echiopsis_curta
Hemiaspis_dameli
Hydrelaps_darwiniensis
Hydrophis_cyanocinctus
Ephalophis_greyae
Parahydrophis_mertoni
Aipysurus_laevis
Emydocephalus_annulatus
Parasuta_monachus
Suta_suta
Elapognathus_coronatus
Cryptophis_nigrescens
Rhinoplocephalus_bicolor
Cacophis_squamulosus
Denisonia_devisi
Pseudechis_australis
Neelaps_calonotus
Vermicella_intermedia
Oxyuranus_microlepidotus
Pseudonaja_textilis
Aspidomorphus_muelleri
Furina_diadema
Acanthophis_praelongus
Brachyurophissemifasciatus
Simoselaps_anomalus
Demansia_papuensis
Toxicocalamus_preussi
Micropechis_ikaheka
Laticauda_colubrina
Bungarus_candidus
Bungarus_multicinctus
Bungarus_niger
Bungarus_caeruleus
Bungarus_sindanus
Bungarus_fasciatus
Bungarus_ceylonicus
Bungarus_bungaroides
Bungarus_slowinskii
Bungarus_flaviceps
Naja_annulifera
Naja_haje
Naja_nivea
Naja_melanoleuca
Naja_annulata
Naja_multifasciata
Naja_ashei
Naja_mossambica
Naja_nigricollis
Naja_katiensis
Naja_nubiae
Naja_pallida
Naja_atra
Naja_kaouthia
Naja_siamensis
Naja_sumatrana
Naja_naja
Hemachatus_haemachatus
Aspidelaps_scutatus
Walterinnesia_aegyptia
Dendroaspis_angusticeps
Ophiophagus_hannah
Elapsoidea_semiannulata
Hemibungarus_calligaster
Calliophis_bibroni
Sinomicrurus_macclellandi
Sinomicrurus_kelloggii
Sinomicrurus_japonicus
Micrurus_surinamensis
Micruroides_euryxanthus
9
Calliophis_castoe
Calliophis_nigrescens
Calliophis_melanurus
Calliophis_bivirgata
Calliophis_intestinalis
Dromophis_lineatus
Compsophis_albiventris
Homoroselaps_lacteus
Oxyrhabdium_leporinum
Cantoria_violacea
Cerberus_rynchops
Oligodon_arnensis
Lycodon_osmanhilli
Thermophis_zhaoermii
Rhabdophis_subminiatus
10
Thamnophis_sirtalis
Crotalus_atrox
11
Crotalus_viridis
Sistrurus_catenatus
Trimeresurus_trigonocephalus
Bitis_arietans
Vipera_ursinii
Pareas_carinatus
7
Achalinus_meiguensis
Acrochordus_granulatus
Acrochordus_javanicus
8
Boa_constrictor
Eryx_miliaris
Eunectes_notaeus
Broghammerus_reticulatus
Loxocemus_bicolor
Xenopeltis_unicolor
Cylindrophis_maculatus
Cylindrophis_ruffus
6
Anilius_scytale
Indotyphlops_braminus

Gekkonidae

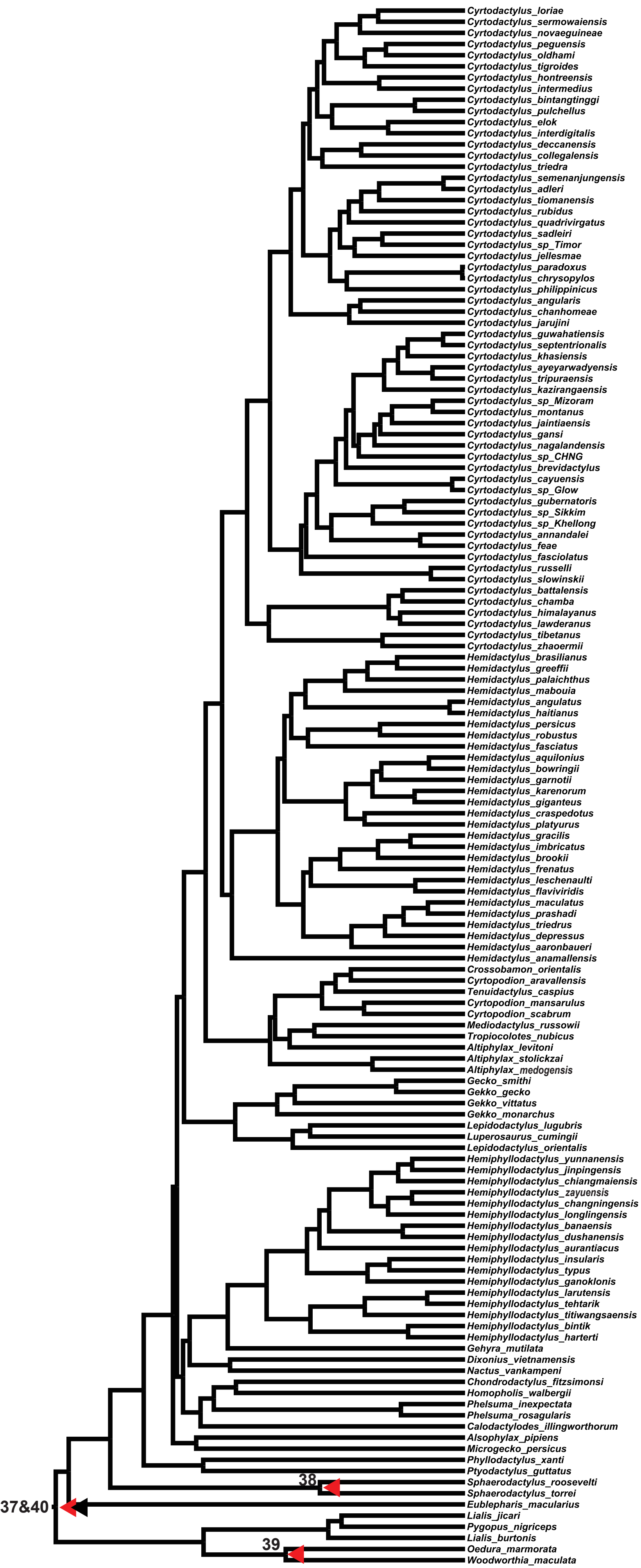

Cyrtodactylus_loriae
Cyrtodactylus_sermowaiensis
Cyrtodactylus_novaeguineae
Cyrtodactylus_peguensis
Cyrtodactylus_oldhami
Cyrtodactylus_tigroides
Cyrtodactylus_hontreensis
Cyrtodactylus_intermedius
Cyrtodactylus_bintangtinggi
Cyrtodactylus_pulchellus
Cyrtodactylus_elok
Cyrtodactylus_interdigitalis
Cyrtodactylus_deccanensis
Cyrtodactylus_collegalensis
Cyrtodactylus_triedra
Cyrtodactylus_semenanjungensis
Cyrtodactylus_adleri
Cyrtodactylus_tiomanensis
Cyrtodactylus_rubidus
Cyrtodactylus_quadrivirgatus
Cyrtodactylus_sadleiri
Cyrtodactylus_sp_Timor
Cyrtodactylus_jellesmae
Cyrtodactylus_paradoxus
Cyrtodactylus_chrysopylos
Cyrtodactylus_philippinicus
Cyrtodactylus_angularis
Cyrtodactylus_chanhomeae
Cyrtodactylus_jarujini
Cyrtodactylus_guwahatiensis
Cyrtodactylus_septentrionalis
Cyrtodactylus_khasiensis
Cyrtodactylus_ayeyarwadyensis
Cyrtodactylus_tripuraensis
Cyrtodactylus_kazirangaensis
Cyrtodactylus_sp_Mizoram
Cyrtodactylus_montanus
Cyrtodactylus_jaintiaensis
Cyrtodactylus_gansi
Cyrtodactylus_nagalandensis
Cyrtodactylus_sp_CHNG
Cyrtodactylus_brevidactylus
Cyrtodactylus_cayuensis
Cyrtodactylus_sp_Glow
Cyrtodactylus_gubernatoris
Cyrtodactylus_sp_Sikkim
Cyrtodactylus_sp_Khellong
Cyrtodactylus_annandalei
Cyrtodactylus_feae
Cyrtodactylus_fasciolatus
Cyrtodactylus_russelli
Cyrtodactylus_slowinskii
Cyrtodactylus_battalensis
Cyrtodactylus_chamba
Cyrtodactylus_himalayanus
Cyrtodactylus_lawderanus
Cyrtodactylus_tibetanus
Cyrtodactylus_zhaoermii
Hemidactylus_brasilianus
Hemidactylus_greeffii
Hemidactylus_palaichthus
Hemidactylus_mabouia
Hemidactylus_angulatus
Hemidactylus_haitianus
Hemidactylus_persicus
Hemidactylus_robustus
Hemidactylus_fasciatus
Hemidactylus_aquilonius
Hemidactylus_bowringii
Hemidactylus_garnotii
Hemidactylus_karenorum
Hemidactylus_giganteus
Hemidactylus_craspedotus
Hemidactylus_platyurus
Hemidactylus_gracilis
Hemidactylus_imbricatus
Hemidactylus_brookii
Hemidactylus_frenatus
Hemidactylus_leschenaulti
Hemidactylus_flaviviridis
Hemidactylus_maculatus
Hemidactylus_prashadi
Hemidactylus_triedrus
Hemidactylus_depressus
Hemidactylus_aaronbaueri
Hemidactylus_anamallensis
Crossobamon_orientalis
Cyrtopodion_aravallensis
Tenuidactylus_caspius
Cyrtopodion_mansarulus
Cyrtopodion_scabrum
Mediodactylus_russowii
Tropiocolotes_nubicus
Altiphylax_levitoni
Altiphylax_stolickzai
Altiphylax_medogensis
Gecko_smithi
Gekko_gecko
Gekko_vittatus
Gekko_monarchus
Lepidodactylus_lugubris
Luperosaurus_cumingii
Lepidodactylus_orientalis
Hemiphyllodactylus_yunnanensis
Hemiphyllodactylus_jinpingensis
Hemiphyllodactylus_chiangmaiensis
Hemiphyllodactylus_zayuensis
Hemiphyllodactylus_changningensis
Hemiphyllodactylus_longlingensis
Hemiphyllodactylus_banaensis
Hemiphyllodactylus_dushanensis
Hemiphyllodactylus_aurantiacus
Hemiphyllodactylus_insularis
Hemiphyllodactylus_typus
Hemiphyllodactylus_ganoklonis
Hemiphyllodactylus_larutensis
Hemiphyllodactylus_tehtarik
Hemiphyllodactylus_titiwangsaensis
Hemiphyllodactylus_bintik
Hemiphyllodactylus_harterti
Gehyra_mutilata
Dixonius_vietnamensis
Nactus_vankampeni
Chondrodactylus_fitzsimonsi
Homopholis_walbergii
Phelsuma_inexpectata
Phelsuma_rosagularis
Calodactylodes_illingworthorum
Alsophylax_pipiens
Microgecko_persicus
Phyllodactylus_xanti
Ptyodactylus_guttatus
38
Sphaerodactylus_roosevelti
Sphaerodactylus_torrei
37&40
Eublepharis_macularius
Lialis_jicari
Pygopus_nigriceps
Lialis_burtonis
39
Oedura_marmorata
Woodworthia_maculata

Megophryidae

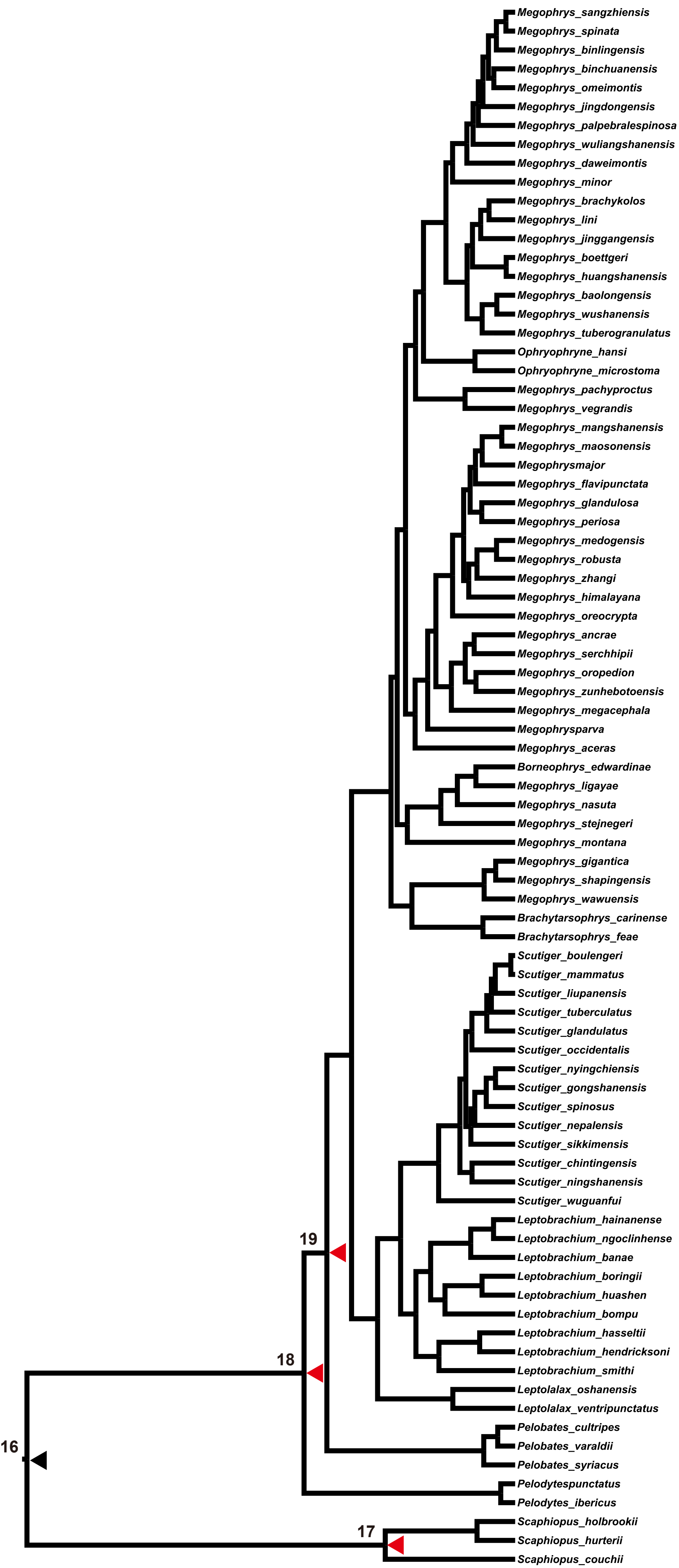

Pareidae

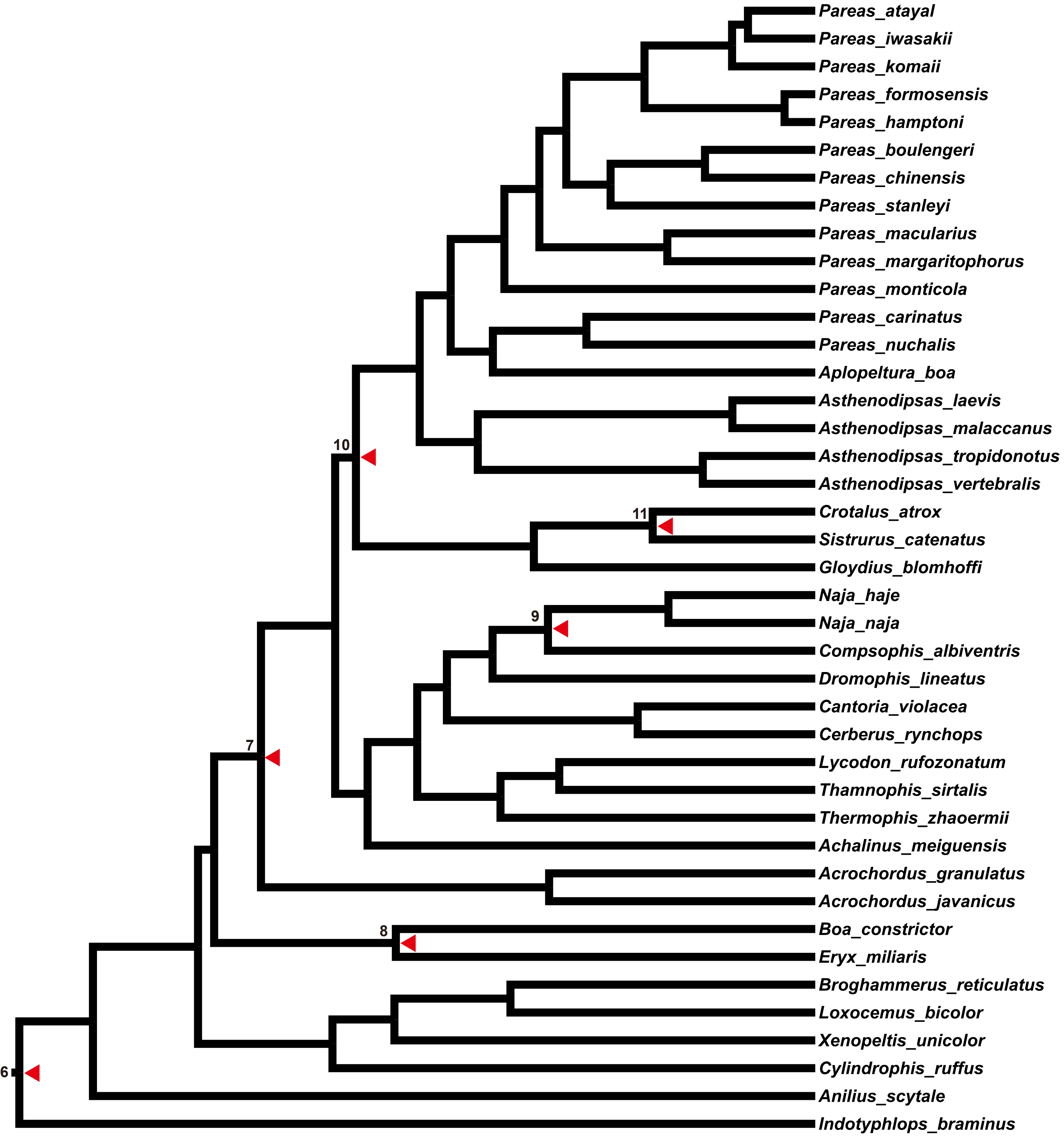

Pareas_atayal
Pareas_iwasakii
Pareas_komaii
Pareas_formosensis
Pareas_hamptoni
Pareas_boulengeri
Pareas_chinensis
Pareas_stanleyi
Pareas_macularius
Pareas_margaritophorus
Pareas_monticola
Pareas_carinatus
Pareas_nuchalis
Aplopeltura_boa
Asthenodipsas_laevis
Asthenodipsas_malaccanus
Asthenodipsas_tropidonotus
Asthenodipsas_vertebralis
Crotalus_atrox
Sistrurus_catenatus
Gloydius_blomhoffi
Naja_haje
Naja_naja
Compsophis_albiventris
Dromophis_lineatus
Cantoria_violacea
Cerberus_rynchops
Lycodon_rufozonatum
Thamnophis_sirtalis
Thermophis_zhaoermii
Achalinus_meiguensis
Acrochordus_granulatus
Acrochordus_javanicus
Boa_constrictor
Eryx_miliaris
Broghammerus_reticulatus
Loxocemus_bicolor
Xenopeltis_unicolor
Cylindrophis_ruffus
Anilius_scytale
Indotyphlops_braminus
10
11
9
7
8
6

Ranidae

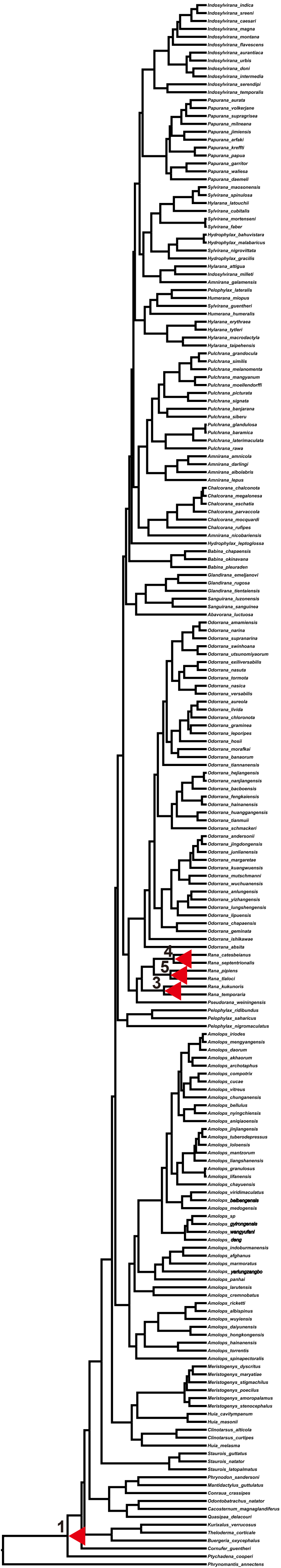

Indosylvirana_indica
Indosylvirana_sreeni
Indosylvirana_caesari
Indosylvirana_magna
Indosylvirana_montana
Indosylvirana_flavescens
Indosylvirana_aurantiaca
Indosylvirana_urbis
Indosylvirana_doni
Indosylvirana_intermedia
Indosylvirana_serendipi
Indosylvirana_temporalis
Papurana_aurata
Papurana_volkerjane
Papurana_supragrisea
Papurana_milneana
Papurana_jimiensis
Papurana_arfaki
Papurana_kreffti
Papurana_papua
Papurana_garritor
Papurana_waliesa
Papurana_daemeli
Sylvirana_maosonensis
Sylvirana_spinulosa
Hylarana_latouchii
Sylvirana_cubitalis
Sylvirana_mortenseni
Sylvirana_faber
Hydrophylax_bahuvistara
Hydrophylax_malabaricus
Sylvirana_nigrovittata
Hydrophylax_gracilis
Hylarana_attigua
Indosylvirana_milleti
Amnirana_galamensis
Pelophylax_lateralis
Humerana_miopus
Sylvirana_guentheri
Humerana_humeralis
Hylarana_erythraea
Hylarana_tytleri
Hylarana_macrodactyla
Hylarana_taipehensis
Pulchrana_grandocula
Pulchrana_similis
Pulchrana_melanomenta
Pulchrana_mangyanum
Pulchrana_moellendorffi
Pulchrana_picturata
Pulchrana_signata
Pulchrana_banjarana
Pulchrana_siberu
Pulchrana_glandulosa
Pulchrana_baramica
Pulchrana_laterimaculata
Pulchrana_rawa
Amnirana_amnicola
Amnirana_darlingi
Amnirana_albolabris
Amnirana_lepus
Chalcorana_chalconota
Chalcorana_megalonesa
Chalcorana_eschatia
Chalcorana_parvaccola
Chalcorana_mocquardi
Chalcorana_rufipes
Amnirana_nicobariensis
Hydrophylax_leptoglossa
Babina_chapaensis
Babina_okinavana
Babina_pleuraden
Glandirana_emeljanovi
Glandirana_rugosa
Glandirana_tientaiensis
Sanguirana_luzonensis
Sanguirana_sanguinea
Abavorana_luctuosa
Odorrana_amamiensis
Odorrana_narina
Odorrana_supranarina
Odorrana_swinhoana
Odorrana_utsunomiyaorum
Odorrana_exiliversabilis
Odorrana_nasuta
Odorrana_tormota
Odorrana_nasica
Odorrana_versabilis
Odorrana_aureola
Odorrana_livida
Odorrana_chloronota
Odorrana_graminea
Odorrana_leporipes
Odorrana_hosii
Odorrana_morafkai
Odorrana_banaorum
Odorrana_tiannanensis
Odorrana_hejiangensis
Odorrana_nanjiangensis
Odorrana_bacboensis
Odorrana_fengkaiensis
Odorrana_hainanensis
Odorrana_huanggangensis
Odorrana_tianmuii
Odorrana_schmackeri
Odorrana_andersonii
Odorrana_jingdongensis
Odorrana_junlianensis
Odorrana_margaretae
Odorrana_kuangwuensis
Odorrana_mutschmanni
Odorrana_wuchuanensis
Odorrana_anlungensis
Odorrana_yizhangensis
Odorrana_lungshengensis
Odorrana_lipuensis
Odorrana_chapaensis
Odorrana_geminata
Odorrana_ishikawae
Odorrana_absita
4
Rana_catesbeianus
Rana_septentrionalis
5
Rana_pipiens
Rana_tlaloci
3
Rana_kukunoris
Rana_temporaria
Pseudorana_weiningensis
Pelophylax_ridibundus
Pelophylax_saharicus
Pelophylax_nigromaculatus
Amolops_iriodes
Amolops_mengyangensis
Amolops_daorum
Amolops_akhaorum
Amolops_archotaphus
Amolops_compotrix
Amolops_cucae
Amolops_vitreus
Amolops_chunganensis
Amolops_bellulus
Amolops_nyingchiensis
Amolops_aniqiaoensis
Amolops_jinjiangensis
Amolops_tuberodepressus
Amolops_loloensis
Amolops_mantzorum
Amolops_liangshanensis
Amolops_granulosus
Amolops_lifanensis
Amolops_chayuensis
Amolops_viridimaculatus
Amolops_beibengensis
Amolops_medogensis
Amolops_sp
Amolops_gyirongensis
Amolops_wangyufani
Amolops_deng
Amolops_indoburmanensis
Amolops_afghanus
Amolops_marmoratus
Amolops_yarlungzangbo
Amolops_panhai
Amolops_larutensis
Amolops_cremnobatus
Amolops_ricketti
Amolops_albispinus
Amolops_wuyiensis
Amolops_daiyunensis
Amolops_hongkongensis
Amolops_hainanensis
Amolops_torrentis
Amolops_spinapectoralis
Meristogenys_dyscritus
Meristogenys_maryatiae
Meristogenys_stigmachilus
Meristogenys_poecilus
Meristogenys_amoropalamus
Meristogenys_stenocephalus
Huia_cavitympanum
Huia_masonii
Clinotarsus_alticola
Clinotarsus_curtipes
Huia_melasma
Staurois_guttatus
Staurois_natator
Staurois_latopalmatus
Phrynodon_sandersoni
Mantidactylus_guttulatus
Conraua_crassipes
Odontobatrachus_natator
Cacosternum_magnaglandiferus
Quasipaa_delacouri
1
Kurixalus_verrucosus
Theloderma_corticale
Buergeria_oxycephalus
Cornufer_guentheri
Ptychadena_cooperi
Phrynomantis_annectens

Rhacophoridae

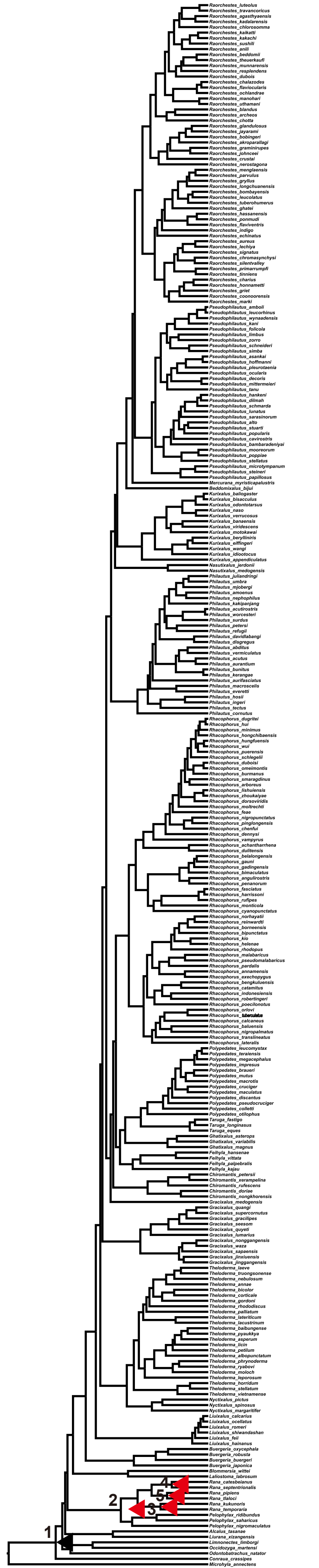

Scincidae

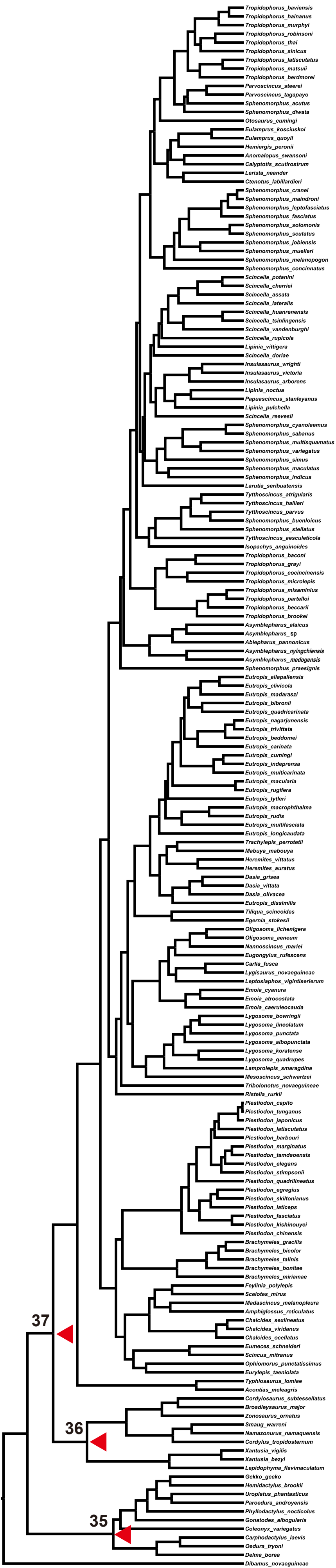

Tropidophorus_baviensis
Tropidophorus_hainanus
Tropidophorus_murphyi
Tropidophorus_robinsoni
Tropidophorus_thai
Tropidophorus_sinicus
Tropidophorus_latiscutatus
Tropidophorus_matsuii
Tropidophorus_berdmorei
Parvoscincus_steerei
Parvoscincus_tagapayo
Sphenomorphus_acutus
Sphenomorphus_diwata
Otosaurus_cumingi
Eulamprus_kosciuskoi
Eulamprus_quoyii
Hemiergis_peronii
Anomalopus_swansoni
Calyptotis_scutirostrum
Lerista_neander
Ctenotus_labillardieri
Sphenomorphus_cranei
Sphenomorphus_maindroni
Sphenomorphus_leptofasciatus
Sphenomorphus_fasciatus
Sphenomorphus_solomonis
Sphenomorphus_scutatus
Sphenomorphus_jobiensis
Sphenomorphus_muelleri
Sphenomorphus_melanopogon
Sphenomorphus_concinnatus
Scincella_potanini
Scincella_cherriei
Scincella_assata
Scincella_lateralis
Scincella_huanrenensis
Scincella_tsinlingensis
Scincella_vandenburghi
Scincella_rupicola
Lipinia_vittigera
Scincella_doriae
Insulasaurus_wrighti
Insulasaurus_victoria
Insulasaurus_arborens
Lipinia_noctua
Papuascincus_stanleyanus
Lipinia_pulchella
Scincella_reevesii
Sphenomorphus_cyanolaemus
Sphenomorphus_sabanus
Sphenomorphus_multisquamatus
Sphenomorphus_variegatus
Sphenomorphus_simus
Sphenomorphus_maculatus
Sphenomorphus_indicus
Larutia_seribuatensis
Tytthoscincus_atrigularis
Tytthoscincus_hallieri
Tytthoscincus_parvus
Sphenomorphus_buenloicus
Sphenomorphus_stellatus
Tytthoscincus_aesculeticola
Isopachys_anguinoides
Tropidophorus_baconi
Tropidophorus_grayi
Tropidophorus_cocincinensis
Tropidophorus_microlepis
Tropidophorus_misaminius
Tropidophorus_partelloi
Tropidophorus_beccarii
Tropidophorus_brookei
Asymblepharus_alaicus
Asymblepharus_sp
Ablepharus_pannonicus
Asymblepharus_nyingchiensis
Asymblepharus_medogensis
Sphenomorphus_praesignis
Eutropis_allapallensis
Eutropis_clivicola
Eutropis_madaraszi
Eutropis_bibronii
Eutropis_quadricarinata
Eutropis_nagarjunensis
Eutropis_trivittata
Eutropis_beddomei
Eutropis_carinata
Eutropis_cumingi
Eutropis_indeprensa
Eutropis_multicarinata
Eutropis_macularia
Eutropis_rugifera
Eutropis_tytleri
Eutropis_macrophthalma
Eutropis_rudis
Eutropis_multifasciata
Eutropis_longicaudata
Trachylepis_perrotetii
Mabuya_mabouya
Heremites_vittatus
Heremites_auratus
Dasia_grisea
Dasia_vittata
Dasia_olivacea
Eutropis_dissimilis
Tiliqua_scincoides
Egernia_stokesii
Oligosoma_lichenigera
Oligosoma_aeneum
Nannoscincus_mariei
Eugongylus_rufescens
Carlia_fusca
Lygisaurus_novaeguineae
Leptosiaphos_vigintiserierum
Emoia_cyanura
Emoia_atrocostata
Emoia_caeruleocauda
Lygosoma_bowringii
Lygosoma_lineolatum
Lygosoma_punctata
Lygosoma_albopunctata
Lygosoma_koratense
Lygosoma_quadrupes
Lamprolepis_smaragdina
Mesoscincus_schwartzei
Tribolonotus_novaeguineae
Ristella_rurkii
Plestiodon_capito
Plestiodon_tunganus
Plestiodon_japonicus
Plestiodon_latiscutatus
Plestiodon_barbouri
Plestiodon_marginatus
Plestiodon_tamdaoensis
Plestiodon_elegans
Plestiodon_stimpsonii
Plestiodon_quadrilineatus
Plestiodon_egregius
Plestiodon_skiltonianus
Plestiodon_laticeps
Plestiodon_fasciatus
Plestiodon_kishinouyei
Plestiodon_chinensis
Brachymeles_gracilis
Brachymeles_bicolor
Brachymeles_talinis
Brachymeles_bonitae
Brachymeles_miriamae
Feylinia_polylepis
Scelotes_mirus
Madascincus_melanopleura
Amphiglossus_reticulatus
Chalcides_sexlineatus
Chalcides_viridanus
Chalcides_ocellatus
Eumeces_schneideri
Scincus_mitranus
Ophiomorus_punctatissimus
Eurylepis_taeniolata
Typhlosaurus_lomiae
Acontias_meleagris
Cordylosaurus_subtessellatus
Broadleysaurus_major
Zonosaurus_ornatus
Smaug_warreni
Namazonurus_namaquensis
Cordylus_tropidosternum
Xantusia_vigilis
Xantusia_bezyi
Lepidophyma_flavimaculatum
Gekko_gecko
Hemidactylus_brookii
Uroplatus_phantasticus
Paroedura_androyensis
Phyllodactylus_nocticolus
Gonatodes_albogularis
Coleonyx_variegatus
Carphodactylus_laevis
Oedura_tryoni
Delma_borea
Dibamus_novaeguineae
37
36
35

Viperidae

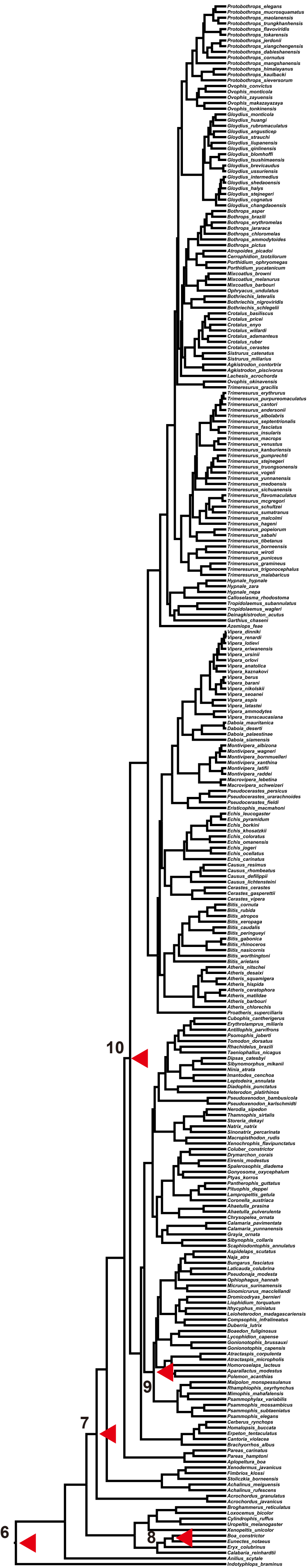
Protobothrops_elegans
Protobothrops_mucrosquamatus
Protobothrops_maolanensis
Protobothrops_trungkhanhensis
Protobothrops_flavoviridis
Protobothrops_tokarensis
Protobothrops_jerdonii
Protobothrops_xiangchengensis
Protobothrops_dabieshanensis
Protobothrops_cornutus
Protobothrops_mangshanensis
Protobothrops_himalayanus
Protobothrops_kaulbacki
Protobothrops_sieversorum
Ovophis_convictus
Ovophis_monticola
Ovophis_zayuensis
Ovophis_makazayazaya
Ovophis_tonkinensis
Gloydius_monticola
Gloydius_huangi
Gloydius_rubromaculatus
Gloydius_angusticep
Gloydius_strauchi
Gloydius_liupanensis
Gloydius_qinlinensis
Gloydius_blomhoffi
Gloydius_tsushimaensis
Gloydius_brevicaudus
Gloydius_ussuriensis
Gloydius_intermedius
Gloydius_shedaoensis
Gloydius_halys
Gloydius_stejnegeri
Gloydius_cognatus
Gloydius_changdaoensis
Bothrops_asper
Bothrops_brazili
Bothrops_erythromelas
Bothrops_jararaca
Bothrops_chloromelas
Bothrops_ammodytoides
Bothrops_pictus
Atropoides_picadoi
Cerrophidion_tzotzilorum
Porthidium_ophryomegas
Porthidium_yucatanicum
Mixcoatlus_browni
Mixcoatlus_melanurus
Mixcoatlus_barbouri
Ophryacus_undulatus
Bothriechis_lateralis
Bothriechis_nigroviridis
Bothriechis_schlegelii
Crotalus_basiliscus
Crotalus_pricei
Crotalus_enyo
Crotalus_willardi
Crotalus_adamanteus
Crotalus_ruber
Crotalus_cerastes
Sistrurus_catenatus
Sistrurus_miliarius
Agkistrodon_contortrix
Agkistrodon_piscivorus
Lachesis_acrochorda
Ovophis_okinavensis
Trimeresurus_gracilis
Trimeresurus_erythrurus
Trimeresurus_purpureomaculatus
Trimeresurus_cantori
Trimeresurus_andersonii
Trimeresurus_albolabris
Trimeresurus_septentrionalis
Trimeresurus_fasciatus
Trimeresurus_insularis
Trimeresurus_macrops
Trimeresurus_venustus
Trimeresurus_kanburiensis
Trimeresurus_gumprechti
Trimeresurus_stejnegeri
Trimeresurus_truongsonensis
Trimeresurus_vogeli
Trimeresurus_yunnanensis
Trimeresurus_medoensis
Trimeresurus_sichuanensis
Trimeresurus_flavomaculatus
Trimeresurus_mcgregori
Trimeresurus_schultzei
Trimeresurus_sumatranus
Trimeresurus_malcolmi
Trimeresurus_hageni
Trimeresurus_popeiorum
Trimeresurus_sabahi
Trimeresurus_tibetanus
Trimeresurus_borneensis
Trimeresurus_wiroti
Trimeresurus_puniceus
Trimeresurus_gramineus
Trimeresurus_trigonocephalus
Trimeresurus_malabaricus
Hypnale_hypnale
Hypnale_zara
Hypnale_nepa
Calloselasma_rhodostoma
Tropidolaemus_subannulatus
Tropidolaemus_wagleri
Deinagkistrodon_acutus
Garthius_chaseni
Azemiops_feae
Vipera_dinniki
Vipera_renardi
Vipera_lotievi
Vipera_eriwanensis
Vipera_ursinii
Vipera_orlovi
Vipera_anatolica
Vipera_kaznakovi
Vipera_berus
Vipera_barani
Vipera_nikolskii
Vipera_seoanei
Vipera_aspis
Vipera_latastei
Vipera_ammodytes
Vipera_transcaucasiana
Daboia_mauritanica
Daboia_deserti
Daboia_palaestinae
Daboia_siamensis
Montivipera_albizona
Montivipera_wagneri
Montivipera_bornmuelleri
Montivipera_xanthina
Montivipera_latifii
Montivipera_raddei
Macrovipera_lebetina
Macrovipera_schweizeri
Pseudocerastes_persicus
Pseudocerastes_urarachnoides
Pseudocerastes_fieldi
Eristicophis_macmahoni
Echis_leucogaster
Echis_pyramidum
Echis_borkini
Echis_khosatzkii
Echis_coloratus
Echis_omanensis
Echis_jogeri
Echis_ocellatus
Echis_carinatus
Causus_resimus
Causus_rhombeatus
Causus_defilippii
Causus_lichtensteini
Cerastes_cerastes
Cerastes_gasperettii
Cerastes_vipera
Bitis_cornuta
Bitis_rubida
Bitis_atropos
Bitis_xeropaga
Bitis_caudalis
Bitis_peringueyi
Bitis_gabonica
Bitis_rhinoceros
Bitis_nasicornis
Bitis_worthingtoni
Bitis_arietans
Atheris_nitschei
Atheris_desaixi
Atheris_squamigera
Atheris_hispida
Atheris_ceratophora
Atheris_matildae
Atheris_barbouri
Atheris_chlorechis
Proatheris_superciliaris
Cubophis_cantherigerus
Erythrolamprus_miliaris
Antillophis_parvifrons
Psomophis_joberti
Tomodon_dorsatus
Rhachidelus_brazili
Taeniophallus_nicagus
Dipsas_catesbyi
Sibynomorphus_mikanii
Ninia_atrata
Imantodes_cenchoa
Leptodeira_annulata
Diadophis_punctatus
Heterodon_platirhinos
Pseudoxenodon_bambusicola
Pseudoxenodon_karlschmidti
Nerodia_sipedon
Thamnophis_sirtalis
Storeria_dekayi
Natrix_natrix
Sinonatrix_percarinata
Macropisthodon_rudis
Xenochrophis_flavipunctatus
Coluber_constrictor
Drymarchon_corais
Eirenis_modestus
Spalerosophis_diadema
Gonyosoma_oxycephalum
Ptyas_korros
Pantherophis_guttatus
Pituophis_deppei
Lampropeltis_getula
Coronella_austriaca
Ahaetulla_prasina
Ahaetulla_pulverulenta
Chrysopelea_ornata
Calamaria_pavimentata
Calamaria_yunnanensis
Grayia_ornata
Sibynophis_collaris
Scaphiodontophis_annulatus
Aspidelaps_scutatus
Naja_atra
Bungarus_fasciatus
Laticauda_colubrina
Pseudonaja_modesta
Ophiophagus_hannah
Micrurus_surinamensis
Sinomicrurus_macclellandi
Dromicodryas_bernieri
Liophidium_torquatum
Ithycyphus_miniatus
Leioheterodon_madagascariensis
Compsophis_infralineatus
Duberria_lutrix
Boaedon_fuliginosus
Lycophidion_capense
Gonionotophis_brussauxi
Gonionotophis_capensis
Atractaspis_corpulenta
Atractaspis_micropholis
Homoroselaps_lacteus
Aparallactus_modestus
Polemon_acanthias
Malpolon_monspessulanus
Rhamphiophis_oxyrhynchus
Mimophis_mahafalensis
Psammophylax_variabilis
Psammophis_mossambicus
Psammophis_subtaeniatus
Psammophis_elegans
Cerberus_rynchops
Homalopsis_buccata
Erpeton_tentaculatus
Cantoria_violacea
Brachyorrhos_albus
Pareas_carinatus
Pareas_hamptoni
Aplopeltura_boa
Xenodermus_javanicus
Fimbrios_klossi
Stoliczkia_borneensis
Achalinus_meiguensis
Achalinus_rufescens
Acrochordus_granulatus
Acrochordus_javanicus
Broghammerus_reticulatus
Loxocemus_bicolor
Cylindrophis_ruffus
Uropeltis_melanogaster
Xenopeltis_unicolor
Boa_constrictor
Eunectes_notaeus
Eryx_colubrinus
Calabaria_reinhardtii
Anilius_scytale
Indotyphlops_braminus
10
9
7
8
6

Data S7.

Origination, peaks and inflection points detected of MDivE and MDisE curves from 1,000 bootstrap pseudoreplicates.

In situ **diversification**	**Bootstrap (BS)**	**Origination time (Ma)**	**Time of the peak (Ma)**	**Inflection point 1 (Ma)**	**Inflection point 2 (Ma)**
	BS1	67	14	11	28
	BS2	67	14	11	24
	BS3	67	14	11	35
	BS4	67	11	35	56
	BS5	67	11	35	56
	BS6	67	11	11	35
	BS7	57	11	35	51
	BS8	57	11	31	56
	BS9	57	11	27	55
	BS10	67	11	11	31
	BS11	67	14	11	28
	BS12	67	14	35	56
	BS13	67	14	35	51
	BS14	67	14	24	56
	BS15	67	14	31	56
	BS16	67	14	35	56
	BS17	67	14	11	29
	BS18	67	14	11	43
	BS19	57	14	28	55
	BS20	67	14	35	56
	BS21	67	14	35	56
	BS22	57	14	11	31
	BS23	57	11	27	52
	BS24	67	11	27	55
	BS25	67	11	31	54
	BS26	67	11	31	51
	BS27	67	11	35	56
	BS28	67	11	35	54
	BS29	67	11	27	51
	BS30	67	11	30	54
	BS31	67	11	11	35
	BS32	67	11	35	56
	BS33	67	11	28	51
	BS34	57	11	35	51
	BS35	67	11	28	55
	BS36	57	11	35	56
	BS37	67	11	29	46
	BS38	67	11	35	56

BS39	67	11	11	35
BS40	67	11	11	35
BS41	67	11	11	35
BS42	67	11	35	56
BS43	67	11	28	55
BS44	67	11	27	51
BS45	67	11	28	51
BS46	67	11	35	56
BS47	67	11	27	51
BS48	67	11	27	55
BS49	67	11	27	51
BS50	57	11	26	51
BS51	67	11	28	51
BS52	67	11	11	28
BS53	67	11	28	51
BS54	67	11	35	56
BS55	67	11	31	54
BS56	57	11	27	55
BS57	67	11	21	51
BS58	67	11	35	56
BS59	67	11	26	43
BS60	67	11	27	52
BS61	57	14	11	28
BS62	67	14	27	55
BS63	67	14	27	52
BS64	57	14	27	55
BS65	57	14	27	51
BS66	67	14	27	51
BS67	54	14	35	56
BS68	67	14	27	51
BS69	57	14	28	46
BS70	67	14	11	27
BS71	67	14	25	43
BS72	67	14	27	51
BS73	67	14	27	55
BS74	67	14	11	35
BS75	57	14	27	51
BS76	57	14	11	28
BS77	67	14	27	55
BS78	67	14	35	56

BS79	67	14	11	29
BS80	57	14	11	35
BS81	67	14	28	51
BS82	67	14	31	54
BS83	57	14	25	35
BS84	67	14	30	51
BS85	67	14	11	27
BS86	57	14	28	55
BS87	67	14	31	54
BS88	67	14	23	51
BS89	67	14	24	51
BS90	57	14	11	35
BS91	54	14	31	54
BS92	54	14	28	51
BS93	57	14	27	55
BS94	67	14	35	56
BS95	67	14	11	27
BS96	67	14	11	28
BS97	54	14	28	55
BS98	67	14	19	31
BS99	67	14	31	56
BS100	54	14	21	51
BS101	54	14	28	56
BS102	57	14	24	56
BS103	67	14	27	51
BS104	67	14	35	51
BS105	57	14	35	51
BS106	67	14	35	51
BS107	67	14	35	56
BS108	67	14	27	55
BS109	67	14	35	51
BS110	67	14	27	55
BS111	67	14	24	54
BS112	57	14	27	51
BS113	54	14	23	45
BS114	67	14	25	35
BS115	67	14	27	55
BS116	67	14	35	56
BS117	67	14	35	51
BS118	67	14	28	51

BS119	67	14	28	51
BS120	57	14	24	54
BS121	67	14	28	55
BS122	67	14	27	51
BS123	67	14	24	54
BS124	57	14	27	52
BS125	67	14	28	55
BS126	67	14	35	51
BS127	67	14	28	55
BS128	67	14	27	55
BS129	57	14	24	54
BS130	67	14	20	35
BS131	67	14	28	51
BS132	67	14	27	55
BS133	67	14	27	55
BS134	67	14	35	51
BS135	57	14	35	56
BS136	67	14	31	54
BS137	67	14	27	54
BS138	67	14	27	52
BS139	67	14	35	56
BS140	67	14	20	43
BS141	67	14	27	52
BS142	57	14	35	54
BS143	67	14	35	56
BS144	67	14	28	51
BS145	67	14	11	35
BS146	67	14	11	27
BS147	67	14	25	35
BS148	67	14	35	56
BS149	67	14	35	56
BS150	67	14	31	54
BS151	67	14	27	55
BS152	67	14	27	51
BS153	67	14	28	55
BS154	67	14	27	55
BS155	67	14	31	54
BS156	67	14	35	56
BS157	67	14	27	55
BS158	57	14	28	51

BS159	57	14	11	35
BS160	67	14	27	55
BS161	57	14	35	51
BS162	67	14	21	57
BS163	54	14	24	35
BS164	54	14	11	35
BS165	67	14	27	51
BS166	57	14	27	52
BS167	57	14	31	54
BS168	67	14	11	28
BS169	67	14	11	35
BS170	67	14	27	51
BS171	67	14	27	55
BS172	67	14	23	51
BS173	54	14	27	55
BS174	54	14	11	35
BS175	67	14	35	56
BS176	57	14	20	42
BS177	54	14	23	51
BS178	67	14	27	55
BS179	54	14	11	35
BS180	67	14	27	51
BS181	54	14	35	54
BS182	67	14	28	55
BS183	67	14	28	51
BS184	67	14	35	45
BS185	67	14	24	54
BS186	67	14	28	51
BS187	67	14	28	55
BS188	67	14	11	46
BS189	67	14	28	54
BS190	67	14	27	52
BS191	67	14	35	54
BS192	67	14	27	55
BS193	67	14	11	35
BS194	67	14	27	52
BS195	67	14	27	51
BS196	57	14	27	51
BS197	67	14	27	55
BS198	67	14	28	55

BS199	67	14	28	47
BS200	67	14	35	54
BS201	67	14	27	55
BS202	67	14	28	51
BS203	67	14	27	51
BS204	57	14	28	51
BS205	67	14	35	56
BS206	57	14	27	52
BS207	67	14	11	30
BS208	67	14	11	27
BS209	67	14	24	54
BS210	67	14	28	54
BS211	67	14	35	54
BS212	57	14	30	51
BS213	67	14	27	55
BS214	67	14	27	55
BS215	67	14	35	56
BS216	67	14	27	52
BS217	67	14	35	54
BS218	67	14	28	55
BS219	67	14	27	51
BS220	67	14	35	56
BS221	57	14	11	30
BS222	57	14	35	54
BS223	67	14	21	54
BS224	67	14	31	51
BS225	67	14	24	51
BS226	67	14	35	54
BS227	67	14	27	51
BS228	67	14	27	52
BS229	54	14	35	56
BS230	67	14	27	51
BS231	57	14	24	54
BS232	67	14	27	51
BS233	67	14	35	56
BS234	67	14	24	42
BS235	67	14	27	55
BS236	67	14	27	56
BS237	54	14	27	41
BS238	67	14	35	56

BS239	67	14	28	55
BS240	57	14	27	38
BS241	67	14	27	55
BS242	67	14	27	55
BS243	67	14	27	51
BS244	54	14	24	54
BS245	67	14	27	55
BS246	57	14	27	51
BS247	67	14	35	56
BS248	57	14	35	56
BS249	57	14	28	51
BS250	67	14	27	55
BS251	67	14	35	56
BS252	57	14	34	51
BS253	67	14	11	35
BS254	67	14	25	35
BS255	67	14	27	54
BS256	67	14	28	55
BS257	54	14	24	54
BS258	67	14	28	55
BS259	67	14	27	55
BS260	67	14	25	35
BS261	67	14	31	54
BS262	67	14	11	35
BS263	67	14	11	35
BS264	57	14	31	54
BS265	67	14	21	56
BS266	67	14	35	54
BS267	67	14	29	55
BS268	57	14	35	51
BS269	67	14	35	56
BS270	67	14	27	55
BS271	57	14	23	55
BS272	67	14	35	56
BS273	67	14	35	51
BS274	67	14	27	54
BS275	67	14	24	54
BS276	67	14	27	55
BS277	67	14	27	51
BS278	67	14	17	53

BS279	67	14	35	56
BS280	67	14	24	54
BS281	54	14	35	56
BS282	57	14	11	35
BS283	67	14	24	54
BS284	67	14	11	35
BS285	67	14	24	54
BS286	54	14	27	51
BS287	67	14	25	35
BS288	67	14	27	55
BS289	57	14	27	51
BS290	67	14	27	55
BS291	54	14	24	42
BS292	57	14	11	35
BS293	67	14	28	46
BS294	67	14	11	27
BS295	67	14	28	55
BS296	67	14	35	54
BS297	67	14	27	52
BS298	67	14	35	56
BS299	67	14	11	30
BS300	67	14	27	51
BS301	57	14	34	51
BS302	67	14	24	56
BS303	67	14	28	51
BS304	67	14	31	54
BS305	67	14	21	51
BS306	67	14	27	51
BS307	57	14	27	55
BS308	57	14	25	35
BS309	67	14	27	51
BS310	67	14	27	52
BS311	67	14	35	51
BS312	57	14	12	28
BS313	67	14	28	54
BS314	67	14	27	58
BS315	67	14	27	55
BS316	67	14	28	51
BS317	67	14	27	51
BS318	54	14	27	52

BS319	67	14	27	54
BS320	67	14	35	56
BS321	67	14	35	56
BS322	67	14	35	56
BS323	67	14	31	54
BS324	67	14	11	27
BS325	67	14	35	54
BS326	67	14	11	35
BS327	67	14	27	51
BS328	57	14	28	51
BS329	57	14	13	35
BS330	67	14	11	30
BS331	67	14	31	54
BS332	57	14	23	51
BS333	67	14	11	35
BS334	67	14	35	51
BS335	67	14	24	55
BS336	67	14	27	51
BS337	67	14	35	51
BS338	67	14	27	55
BS339	67	14	11	31
BS340	57	14	35	56
BS341	67	14	11	27
BS342	57	14	24	51
BS343	67	14	24	54
BS344	67	14	28	51
BS345	67	14	20	35
BS346	67	14	11	35
BS347	67	14	27	51
BS348	67	14	23	51
BS349	67	14	35	56
BS350	67	14	11	35
BS351	67	14	31	54
BS352	67	14	35	54
BS353	67	14	11	40
BS354	67	14	35	56
BS355	67	14	23	46
BS356	67	14	31	51
BS357	57	14	31	54
BS358	57	14	24	54

BS359	67	14	28	55
BS360	67	14	28	55
BS361	67	14	24	54
BS362	67	14	28	55
BS363	67	14	28	55
BS364	67	14	24	49
BS365	57	14	11	27
BS366	67	14	27	51
BS367	67	14	35	51
BS368	67	14	28	51
BS369	67	14	27	55
BS370	67	14	35	54
BS371	67	14	35	56
BS372	54	14	35	51
BS373	67	14	28	55
BS374	67	14	11	35
BS375	67	14	27	52
BS376	67	14	26	51
BS377	67	14	35	56
BS378	57	14	35	56
BS379	67	14	35	56
BS380	67	14	27	55
BS381	57	14	28	51
BS382	54	14	27	51
BS383	67	14	27	45
BS384	57	14	27	51
BS385	67	14	28	42
BS386	67	14	27	52
BS387	67	14	28	51
BS388	67	14	35	56
BS389	54	14	27	55
BS390	67	14	28	55
BS391	57	14	24	56
BS392	67	14	35	56
BS393	54	14	30	54
BS394	67	14	19	51
BS395	67	14	22	35
BS396	67	14	31	54
BS397	67	14	27	55
BS398	67	14	28	55

BS399	67	14	35	51
BS400	67	14	28	55
BS401	57	14	11	35
BS402	67	14	28	54
BS403	57	14	21	51
BS404	67	14	24	56
BS405	67	14	11	28
BS406	67	14	31	56
BS407	67	14	28	55
BS408	67	14	35	56
BS409	67	14	35	51
BS410	54	14	11	35
BS411	67	14	35	51
BS412	67	14	27	55
BS413	67	14	11	28
BS414	57	14	11	35
BS415	67	14	27	55
BS416	67	14	35	56
BS417	57	14	28	51
BS418	57	14	27	52
BS419	67	14	24	54
BS420	67	14	28	55
BS421	67	14	24	54
BS422	67	14	27	41
BS423	54	14	23	51
BS424	67	14	35	51
BS425	67	14	11	27
BS426	57	14	35	51
BS427	57	14	27	55
BS428	67	14	24	35
BS429	67	14	31	51
BS430	57	14	35	54
BS431	67	14	28	51
BS432	57	14	35	54
BS433	67	14	28	51
BS434	67	14	35	54
BS435	57	14	27	51
BS436	67	14	35	51
BS437	57	14	35	54
BS438	57	14	28	55

BS439	67	14	28	51
BS440	67	14	27	51
BS441	57	14	28	51
BS442	57	14	28	51
BS443	67	14	27	56
BS444	67	14	28	55
BS445	67	14	27	52
BS446	67	14	28	55
BS447	54	14	11	28
BS448	54	14	35	56
BS449	67	14	27	55
BS450	67	14	35	54
BS451	67	14	35	56
BS452	67	14	35	51
BS453	67	14	28	51
BS454	57	14	35	56
BS455	57	14	23	51
BS456	57	14	27	51
BS457	67	14	11	35
BS458	57	14	27	55
BS459	67	14	11	28
BS460	67	14	11	28
BS461	57	14	27	55
BS462	67	14	30	56
BS463	67	14	35	51
BS464	67	14	27	51
BS465	67	14	31	54
BS466	67	14	25	35
BS467	67	14	28	55
BS468	67	14	27	55
BS469	67	14	28	51
BS470	67	14	35	56
BS471	57	14	27	51
BS472	57	14	28	51
BS473	67	14	28	51
BS474	67	14	28	58
BS475	67	14	35	56
BS476	67	14	35	56
BS477	57	14	11	27
BS478	67	14	35	45

BS479	67	14	28	55
BS480	67	14	35	56
BS481	67	14	27	51
BS482	67	14	28	55
BS483	67	14	28	51
BS484	54	14	28	51
BS485	67	14	11	35
BS486	57	14	27	51
BS487	57	14	11	35
BS488	67	14	24	51
BS489	67	14	31	51
BS490	67	14	35	56
BS491	57	14	28	55
BS492	67	14	27	55
BS493	67	14	35	56
BS494	67	14	35	56
BS495	57	14	11	35
BS496	67	14	28	55
BS497	67	14	28	55
BS498	54	14	11	28
BS499	67	14	27	55
BS500	67	14	35	56
BS501	67	14	27	55
BS502	67	14	11	28
BS503	67	14	27	55
BS504	67	14	34	51
BS505	67	14	35	51
BS506	67	14	26	43
BS507	54	14	28	51
BS508	54	14	27	55
BS509	67	14	27	51
BS510	67	14	23	43
BS511	57	14	11	27
BS512	67	14	24	56
BS513	67	14	35	51
BS514	57	14	36	55
BS515	67	14	27	51
BS516	67	14	27	51
BS517	67	14	27	51
BS518	67	14	27	51

BS519	67	14	35	51
BS520	67	14	11	35
BS521	67	14	28	55
BS522	67	14	35	56
BS523	67	14	35	51
BS524	67	14	28	42
BS525	67	14	11	35
BS526	57	14	11	35
BS527	57	14	31	51
BS528	67	14	35	56
BS529	54	14	28	55
BS530	67	14	28	51
BS531	67	14	27	52
BS532	67	14	11	35
BS533	57	14	35	56
BS534	67	14	25	35
BS535	67	14	35	54
BS536	67	14	27	55
BS537	67	14	27	55
BS538	67	14	11	28
BS539	57	14	27	55
BS540	57	14	28	51
BS541	54	14	27	51
BS542	57	14	24	42
BS543	67	14	36	55
BS544	67	14	24	51
BS545	67	14	35	56
BS546	57	14	24	54
BS547	67	14	27	55
BS548	67	14	28	51
BS549	67	14	35	56
BS550	67	14	35	56
BS551	67	14	24	56
BS552	67	14	25	49
BS553	67	14	28	51
BS554	67	14	27	55
BS555	67	14	31	54
BS556	67	14	35	56
BS557	52	14	35	56
BS558	67	14	27	51

BS559	67	14	11	35
BS560	67	14	11	31
BS561	67	14	28	51
BS562	54	14	27	51
BS563	67	14	27	51
BS564	57	14	27	51
BS565	67	14	27	55
BS566	67	14	25	51
BS567	57	14	35	54
BS568	67	14	24	54
BS569	67	14	24	43
BS570	67	14	28	55
BS571	67	14	31	51
BS572	67	14	18	51
BS573	67	14	31	56
BS574	67	14	35	54
BS575	67	14	29	51
BS576	67	14	28	55
BS577	67	14	25	51
BS578	67	14	35	56
BS579	67	14	27	51
BS580	67	14	24	56
BS581	67	14	20	35
BS582	57	14	11	28
BS583	67	14	20	55
BS584	67	14	31	51
BS585	67	14	27	51
BS586	54	14	28	51
BS587	67	14	20	35
BS588	57	14	31	54
BS589	67	14	35	51
BS590	67	14	24	54
BS591	67	14	27	51
BS592	57	14	25	35
BS593	57	14	27	52
BS594	67	14	27	55
BS595	67	14	35	54
BS596	67	14	35	56
BS597	67	14	28	55
BS598	67	14	27	51

BS599	67	14	11	35
BS600	67	14	35	56
BS601	67	14	27	55
BS602	57	14	27	55
BS603	57	14	20	51
BS604	67	14	35	56
BS605	67	14	35	56
BS606	67	14	11	27
BS607	67	14	28	51
BS608	54	14	28	51
BS609	67	14	35	56
BS610	67	14	30	54
BS611	67	14	23	51
BS612	67	14	27	55
BS613	57	14	27	55
BS614	67	14	28	51
BS615	67	14	35	45
BS616	57	14	26	55
BS617	67	14	27	55
BS618	67	14	28	51
BS619	67	14	11	27
BS620	67	14	35	51
BS621	67	14	27	51
BS622	67	14	11	31
BS623	67	14	24	43
BS624	67	14	35	54
BS625	67	14	28	51
BS626	67	14	31	56
BS627	67	14	35	54
BS628	67	14	11	27
BS629	67	14	25	35
BS630	67	14	23	45
BS631	57	13	27	51
BS632	67	13	26	43
BS633	67	13	24	51
BS634	67	13	28	51
BS635	67	13	27	56
BS636	67	13	35	54
BS637	67	13	35	51
BS638	67	13	23	51

BS639	67	13	28	51
BS640	67	13	27	55
BS641	67	13	27	55
BS642	67	13	27	55
BS643	67	13	35	56
BS644	57	13	35	54
BS645	57	13	24	51
BS646	67	13	28	55
BS647	54	13	35	51
BS648	67	13	27	52
BS649	67	13	27	58
BS650	54	13	27	55
BS651	67	13	28	51
BS652	67	13	27	55
BS653	67	13	25	35
BS654	67	13	35	56
BS655	67	13	28	51
BS656	67	13	35	56
BS657	67	13	35	51
BS658	67	13	27	55
BS659	67	13	28	55
BS660	57	13	25	35
BS661	67	13	35	56
BS662	67	13	31	55
BS663	67	13	28	58
BS664	67	13	23	42
BS665	67	13	35	51
BS666	67	13	11	35
BS667	67	13	35	56
BS668	67	13	11	27
BS669	57	13	25	35
BS670	67	13	24	54
BS671	67	13	28	51
BS672	67	13	35	51
BS673	67	13	23	43
BS674	67	13	27	51
BS675	67	13	35	51
BS676	67	13	29	55
BS677	67	13	27	55
BS678	67	13	24	56

BS679	67	13	27	52
BS680	67	13	25	35
BS681	67	13	35	54
BS682	67	13	38	56
BS683	54	13	28	55
BS684	67	13	11	28
BS685	57	13	25	51
BS686	67	13	31	51
BS687	67	13	27	51
BS688	57	13	35	54
BS689	67	13	35	56
BS690	67	13	11	35
BS691	67	13	35	56
BS692	67	13	43	54
BS693	67	13	11	35
BS694	57	13	25	43
BS695	57	13	27	55
BS696	67	13	27	55
BS697	67	13	35	56
BS698	57	13	35	51
BS699	67	13	35	56
BS700	67	13	27	51
BS701	57	13	27	55
BS702	54	13	35	56
BS703	54	13	27	38
BS704	67	13	35	56
BS705	67	13	27	52
BS706	67	13	28	55
BS707	67	13	27	55
BS708	67	13	35	56
BS709	67	13	28	54
BS710	57	13	27	55
BS711	54	13	27	51
BS712	67	13	11	35
BS713	67	13	35	51
BS714	67	13	35	56
BS715	67	13	11	35
BS716	67	13	25	35
BS717	67	13	35	54
BS718	67	13	25	35

BS719	67	13	35	51
BS720	67	13	28	51
BS721	67	13	11	27
BS722	67	13	11	35
BS723	67	13	35	56
BS724	67	13	24	54
BS725	57	13	25	35
BS726	67	13	27	55
BS727	67	13	27	55
BS728	57	13	27	51
BS729	67	13	35	51
BS730	67	13	28	51
BS731	57	13	35	56
BS732	67	13	11	27
BS733	67	13	20	35
BS734	67	13	30	54
BS735	54	13	24	54
BS736	57	13	31	54
BS737	57	13	35	54
BS738	67	13	27	51
BS739	67	13	27	55
BS740	67	13	11	35
BS741	57	13	20	42
BS742	67	13	28	51
BS743	67	13	31	54
BS744	67	13	27	51
BS745	57	13	28	51
BS746	67	13	27	55
BS747	67	13	27	58
BS748	67	13	28	51
BS749	67	13	27	55
BS750	54	13	35	56
BS751	57	13	28	51
BS752	54	13	23	55
BS753	67	13	28	55
BS754	67	13	35	54
BS755	67	13	35	56
BS756	67	13	27	51
BS757	57	13	27	51
BS758	67	13	35	51

BS759	67	13	29	46
BS760	67	13	35	56
BS761	67	13	11	31
BS762	57	13	35	54
BS763	67	13	22	35
BS764	67	13	27	55
BS765	67	13	28	55
BS766	67	13	27	55
BS767	67	13	35	56
BS768	67	13	11	35
BS769	67	13	24	56
BS770	67	13	28	55
BS771	67	13	28	55
BS772	67	13	35	51
BS773	67	13	27	51
BS774	57	13	26	55
BS775	67	13	35	56
BS776	67	13	31	51
BS777	57	13	27	51
BS778	67	13	24	56
BS779	67	13	27	52
BS780	67	13	11	43
BS781	67	13	25	43
BS782	67	13	35	54
BS783	54	13	25	35
BS784	67	13	35	54
BS785	57	13	23	51
BS786	57	13	27	55
BS787	67	13	35	51
BS788	67	13	28	55
BS789	67	13	24	35
BS790	54	13	24	35
BS791	67	13	11	30
BS792	67	13	35	56
BS793	54	13	27	51
BS794	57	13	28	54
BS795	67	13	11	28
BS796	57	13	27	55
BS797	67	13	27	55
BS798	67	13	28	55

BS799	67	13	28	55
BS800	67	13	30	51
BS801	67	13	24	56
BS802	67	13	11	35
BS803	67	13	25	43
BS804	57	13	11	35
BS805	67	13	28	55
BS806	67	13	35	56
BS807	57	13	24	51
BS808	67	13	24	54
BS809	67	13	25	35
BS810	67	13	35	51
BS811	67	13	35	54
BS812	67	13	35	51
BS813	57	13	23	45
BS814	54	13	28	55
BS815	67	13	11	27
BS816	67	13	35	56
BS817	67	13	27	51
BS818	67	13	24	35
BS819	67	13	11	30
BS820	57	13	35	56
BS821	67	13	29	55
BS822	57	13	31	54
BS823	67	13	23	45
BS824	57	13	11	35
BS825	67	13	11	27
BS826	67	13	35	54
BS827	67	13	25	35
BS828	67	13	24	54
BS829	67	13	27	51
BS830	67	13	11	35
BS831	67	13	25	55
BS832	67	13	19	35
BS833	67	13	35	54
BS834	67	13	35	54
BS835	54	13	27	55
BS836	67	13	27	55
BS837	67	13	27	55
BS838	57	13	28	51

BS839	67	13	11	31
BS840	57	13	27	52
BS841	67	13	27	55
BS842	67	13	35	56
BS843	67	13	29	43
BS844	67	13	35	54
BS845	57	13	35	51
BS846	67	13	24	56
BS847	67	13	28	51
BS848	67	13	11	28
BS849	67	13	35	56
BS850	57	13	27	58
BS851	67	13	28	55
BS852	67	13	28	55
BS853	67	13	28	51
BS854	67	13	35	54
BS855	54	13	35	56
BS856	67	13	28	55
BS857	67	13	35	56
BS858	67	13	35	51
BS859	67	13	28	55
BS860	67	13	11	31
BS861	67	13	23	55
BS862	67	13	23	42
BS863	57	13	27	55
BS864	67	13	11	35
BS865	54	13	31	51
BS866	67	13	28	51
BS867	67	13	11	28
BS868	67	13	11	31
BS869	57	13	35	54
BS870	67	13	11	28
BS871	57	13	12	35
BS872	67	13	28	51
BS873	57	13	35	56
BS874	57	13	27	51
BS875	67	13	37	55
BS876	57	13	27	55
BS877	54	13	35	54
BS878	67	13	25	51

BS879	54	13	25	51
BS880	67	13	31	51
BS881	67	13	31	54
BS882	67	13	31	51
BS883	67	13	11	27
BS884	67	13	35	56
BS885	67	13	11	35
BS886	67	13	28	55
BS887	57	13	24	54
BS888	57	13	11	35
BS889	54	13	11	27
BS890	67	13	27	51
BS891	67	13	11	35
BS892	67	13	35	56
BS893	57	13	11	30
BS894	54	13	25	40
BS895	67	13	35	56
BS896	54	13	24	56
BS897	67	13	27	52
BS898	54	13	35	56
BS899	67	13	35	54
BS900	67	13	27	55
BS901	67	13	31	51
BS902	57	13	35	54
BS903	57	13	35	51
BS904	67	13	27	45
BS905	67	13	27	55
BS906	54	13	27	51
BS907	67	13	27	51
BS908	57	13	28	54
BS909	67	13	11	27
BS910	67	13	27	51
BS911	54	13	24	54
BS912	54	13	28	55
BS913	54	13	24	55
BS914	67	13	28	55
BS915	67	13	35	54
BS916	67	13	35	56
BS917	67	13	27	55
BS918	67	13	11	27

BS919	67	13	11	35
BS920	67	13	35	51
BS921	67	13	24	56
BS922	54	13	28	51
BS923	67	13	28	51
BS924	67	13	24	56
BS925	54	13	27	55
BS926	67	13	35	56
BS927	57	13	35	56
BS928	57	13	31	51
BS929	67	13	11	28
BS930	67	13	35	51
BS931	67	13	31	54
BS932	57	13	26	43
BS933	67	13	35	51
BS934	67	13	28	51
BS935	54	13	35	54
BS936	67	13	35	56
BS937	67	13	28	55
BS938	57	13	25	35
BS939	67	13	35	56
BS940	67	13	28	51
BS941	67	13	27	51
BS942	57	13	11	30
BS943	67	13	35	56
BS944	67	13	27	55
BS945	57	13	28	51
BS946	67	13	31	51
BS947	57	13	27	52
BS948	67	13	35	51
BS949	67	13	27	52
BS950	67	13	27	55
BS951	67	13	30	54
BS952	67	13	18	43
BS953	67	13	35	54
BS954	67	13	20	43
BS955	67	13	27	51
BS956	67	13	27	55
BS957	57	13	11	35
BS958	67	13	24	54

BS959	57	13	28	54
BS960	67	13	27	58
BS961	67	13	41	51
BS962	67	13	28	51
BS963	67	13	35	51
BS964	67	13	24	35
BS965	67	13	24	51
BS966	67	13	25	35
BS967	54	13	35	56
BS968	57	13	28	51
BS969	57	13	30	56
BS970	67	13	28	55
BS971	67	13	11	35
BS972	67	13	27	51
BS973	57	13	28	51
BS974	67	13	28	55
BS975	67	13	20	35
BS976	67	13	27	51
BS977	67	13	27	51
BS978	67	13	27	52
BS979	67	13	11	27
BS980	57	13	35	56
BS981	57	13	28	51
BS982	67	13	27	52
BS983	67	13	35	56
BS984	57	13	28	51
BS985	67	13	28	55
BS986	67	13	27	45
BS987	67	13	27	51
BS988	67	13	35	56
BS989	67	13	27	55
BS990	67	13	24	56
BS991	67	13	11	35
BS992	57	13	31	51
BS993	67	13	24	51
BS994	57	13	27	51
BS995	67	13	27	51
BS996	67	13	31	56
BS997	67	13	43	56
BS998	67	13	24	54

	BS999	57	13	35	56
	BS1000	67	13	29	55

Dispersal	Bootstrap (BS)	Origination time (Ma)	Time of the peak (Ma)	Inflection point 1 (Ma)	Inflection point 2 (Ma)
	BS1	67	16	11	51
	BS2	67	17	11	35
	BS3	67	14	23	51
	BS4	67	14	11	35
	BS5	67	14	24	54
	BS6	67	14	30	54
	BS7	57	14	11	43
	BS8	67	14	27	52
	BS9	67	13	28	54
	BS10	67	13	11	35
	BS11	67	13	11	56
	BS12	67	13	11	43
	BS13	67	13	11	35
	BS14	67	13	11	35
	BS15	67	13	11	53
	BS16	67	13	11	35
	BS17	67	13	11	55
	BS18	67	13	35	56
	BS19	67	13	31	46
	BS20	67	15	11	27
	BS21	67	15	11	43
	BS22	67	15	11	51
	BS23	57	15	35	51
	BS24	67	15	27	58
	BS25	67	15	11	28
	BS26	67	15	28	55
	BS27	57	15	27	52
	BS28	67	15	24	40
	BS29	67	15	27	45
	BS30	67	15	27	51
	BS31	67	15	28	51
	BS32	57	15	27	58
	BS33	67	15	11	35
	BS34	67	13	20	35
	BS35	67	13	24	56

BS36	67	13	25	35
BS37	57	13	28	51
BS38	67	13	11	35
BS39	67	13	24	42
BS40	67	13	24	51
BS41	67	13	24	54
BS42	67	13	13	35
BS43	67	13	28	51
BS44	67	13	11	28
BS45	67	13	28	55
BS46	67	13	27	55
BS47	57	13	46	56
BS48	67	13	31	56
BS49	57	13	27	55
BS50	67	13	27	55
BS51	67	13	11	35
BS52	67	13	20	40
BS53	67	13	31	51
BS54	67	13	28	51
BS55	67	13	35	54
BS56	67	13	11	27
BS57	67	13	24	42
BS58	67	13	11	27
BS59	57	13	35	56
BS60	67	13	35	56
BS61	67	13	19	51
BS62	67	13	24	51
BS63	67	13	25	35
BS64	67	13	27	38
BS65	67	13	35	54
BS66	67	13	27	45
BS67	67	13	31	46
BS68	67	13	11	35
BS69	67	13	23	51
BS70	67	13	24	35
BS71	67	13	24	46
BS72	67	13	27	56
BS73	67	13	28	51
BS74	67	13	35	54
BS75	67	13	11	35

BS76	67	13	24	56
BS77	67	13	35	56
BS78	67	13	27	38
BS79	67	13	35	56
BS80	67	13	27	52
BS81	67	13	27	51
BS82	67	13	27	55
BS83	67	13	21	46
BS84	67	13	23	46
BS85	67	13	28	51
BS86	67	13	23	45
BS87	67	13	11	27
BS88	57	13	11	28
BS89	67	13	31	51
BS90	67	13	20	55
BS91	67	13	28	55
BS92	67	13	31	56
BS93	67	13	31	54
BS94	67	13	24	55
BS95	67	13	27	55
BS96	67	13	28	55
BS97	67	13	35	51
BS98	67	13	35	56
BS99	67	13	24	42
BS100	67	13	27	55
BS101	67	13	21	51
BS102	67	13	35	56
BS103	57	13	35	51
BS104	67	13	27	55
BS105	67	13	25	43
BS106	67	13	11	31
BS107	67	13	23	45
BS108	67	13	11	35
BS109	67	13	27	51
BS110	67	13	35	59
BS111	67	13	35	56
BS112	67	13	26	51
BS113	67	13	27	55
BS114	67	13	27	52
BS115	67	13	35	54

BS116	67	13	24	56
BS117	57	13	35	56
BS118	67	13	25	56
BS119	67	13	28	51
BS120	67	13	28	51
BS121	67	13	27	54
BS122	67	13	24	54
BS123	57	13	35	56
BS124	67	13	24	54
BS125	67	13	27	55
BS126	67	13	11	35
BS127	67	13	24	51
BS128	67	13	11	35
BS129	67	13	35	51
BS130	67	13	11	31
BS131	67	13	30	54
BS132	67	13	24	56
BS133	67	13	28	58
BS134	67	13	11	31
BS135	67	14	27	38
BS136	67	14	27	55
BS137	67	14	27	55
BS138	67	14	35	54
BS139	67	14	42	59
BS140	67	14	27	55
BS141	67	14	20	51
BS142	67	14	27	51
BS143	67	14	24	53
BS144	67	14	35	51
BS145	67	14	35	56
BS146	67	14	27	58
BS147	67	14	28	55
BS148	67	14	11	28
BS149	67	14	35	56
BS150	67	14	28	54
BS151	67	14	28	51
BS152	67	14	12	27
BS153	67	14	24	54
BS154	67	14	23	51
BS155	67	14	28	55

BS156	67	14	35	51
BS157	67	14	35	59
BS158	67	14	27	51
BS159	54	14	28	55
BS160	67	14	25	35
BS161	67	14	24	51
BS162	67	14	22	35
BS163	57	14	11	35
BS164	67	14	27	51
BS165	67	14	27	56
BS166	67	14	28	51
BS167	57	14	23	51
BS168	57	14	34	53
BS169	54	14	27	55
BS170	67	14	31	54
BS171	67	14	27	54
BS172	67	14	31	51
BS173	67	14	35	54
BS174	67	14	35	56
BS175	67	14	35	56
BS176	67	14	11	29
BS177	67	14	26	46
BS178	67	14	28	55
BS179	67	14	28	38
BS180	67	14	11	35
BS181	67	14	27	51
BS182	67	14	11	35
BS183	57	14	35	56
BS184	57	14	35	54
BS185	57	14	24	56
BS186	57	14	28	55
BS187	67	14	11	35
BS188	57	14	28	51
BS189	67	14	28	51
BS190	67	14	23	51
BS191	67	14	35	56
BS192	67	14	27	55
BS193	67	14	27	55
BS194	57	14	11	35
BS195	67	14	27	55

BS196	67	14	35	54
BS197	67	13	11	28
BS198	67	13	28	55
BS199	57	13	27	56
BS200	67	13	28	51
BS201	67	13	24	45
BS202	57	13	11	27
BS203	67	13	28	55
BS204	67	13	24	51
BS205	67	13	27	55
BS206	67	13	31	51
BS207	67	13	27	38
BS208	57	13	27	55
BS209	67	13	11	27
BS210	67	13	25	51
BS211	67	13	28	51
BS212	67	13	27	51
BS213	57	13	27	55
BS214	54	13	11	35
BS215	67	13	35	51
BS216	67	13	23	55
BS217	67	13	27	55
BS218	67	13	11	35
BS219	67	13	27	58
BS220	67	13	27	55
BS221	57	13	35	56
BS222	67	13	23	43
BS223	57	13	35	56
BS224	67	13	28	51
BS225	57	13	27	55
BS226	54	13	25	35
BS227	67	13	27	52
BS228	57	13	27	38
BS229	67	13	31	51
BS230	67	13	35	54
BS231	67	13	35	54
BS232	67	13	35	56
BS233	67	13	31	54
BS234	67	13	28	56
BS235	57	13	24	54

BS236	57	13	11	31
BS237	67	13	35	56
BS238	57	13	27	55
BS239	67	13	28	51
BS240	67	13	35	51
BS241	67	13	35	54
BS242	67	13	11	42
BS243	57	13	30	54
BS244	67	13	35	56
BS245	67	13	25	56
BS246	67	13	11	35
BS247	67	13	27	38
BS248	67	13	28	55
BS249	67	13	35	56
BS250	67	13	11	42
BS251	67	13	11	35
BS252	57	13	35	54
BS253	67	13	38	55
BS254	57	13	27	51
BS255	67	13	18	51
BS256	67	13	28	55
BS257	67	13	29	54
BS258	67	13	28	55
BS259	67	13	27	55
BS260	67	13	11	35
BS261	67	13	21	57
BS262	57	13	11	43
BS263	67	13	11	35
BS264	67	13	35	54
BS265	67	13	31	54
BS266	57	13	19	35
BS267	67	13	27	52
BS268	67	13	31	54
BS269	67	13	31	54
BS270	67	13	11	31
BS271	67	13	11	43
BS272	67	13	27	51
BS273	67	13	35	56
BS274	67	13	27	55
BS275	67	13	11	31

BS276	67	13	28	51
BS277	67	13	24	54
BS278	57	13	18	28
BS279	67	13	27	55
BS280	67	13	26	43
BS281	54	13	35	51
BS282	67	13	11	28
BS283	67	13	35	51
BS284	67	13	25	35
BS285	67	13	31	54
BS286	67	13	24	43
BS287	67	13	28	51
BS288	67	13	31	56
BS289	57	13	31	51
BS290	67	13	11	27
BS291	57	13	35	51
BS292	67	13	35	56
BS293	57	13	24	54
BS294	67	13	11	28
BS295	67	13	11	27
BS296	57	13	11	35
BS297	67	13	28	51
BS298	67	13	12	35
BS299	67	13	27	38
BS300	67	13	35	51
BS301	67	13	24	56
BS302	67	13	35	51
BS303	57	13	27	52
BS304	67	13	24	56
BS305	67	13	27	51
BS306	67	13	28	51
BS307	67	13	23	51
BS308	67	13	35	56
BS309	67	13	11	27
BS310	67	13	11	35
BS311	67	13	24	53
BS312	67	13	25	51
BS313	67	13	11	27
BS314	67	13	28	55
BS315	67	13	24	35

BS316	67	13	18	51
BS317	67	13	27	55
BS318	67	13	35	54
BS319	67	13	24	49
BS320	54	13	27	51
BS321	57	13	28	51
BS322	57	13	28	51
BS323	67	13	24	54
BS324	67	13	28	51
BS325	67	13	27	51
BS326	57	13	24	49
BS327	67	13	28	51
BS328	54	13	24	46
BS329	67	13	35	56
BS330	67	13	27	51
BS331	67	13	35	54
BS332	67	13	35	54
BS333	67	13	35	54
BS334	67	13	31	54
BS335	67	13	27	45
BS336	67	13	27	56
BS337	67	13	35	54
BS338	67	13	11	35
BS339	67	13	27	51
BS340	67	13	11	28
BS341	67	13	35	56
BS342	67	13	28	51
BS343	67	13	23	51
BS344	67	13	11	35
BS345	57	13	11	27
BS346	67	13	28	51
BS347	67	13	27	51
BS348	67	13	35	56
BS349	57	13	25	35
BS350	67	13	31	56
BS351	67	13	35	56
BS352	67	13	35	56
BS353	67	13	35	56
BS354	67	13	11	35
BS355	67	13	35	54

BS356	67	13	25	55
BS357	67	13	24	51
BS358	67	13	35	56
BS359	67	13	11	27
BS360	67	13	11	35
BS361	67	13	28	51
BS362	67	13	28	55
BS363	54	13	27	53
BS364	57	13	35	59
BS365	67	13	35	54
BS366	67	13	25	35
BS367	54	13	28	55
BS368	57	13	11	28
BS369	67	13	24	54
BS370	67	13	29	54
BS371	67	13	31	54
BS372	67	13	28	54
BS373	67	13	24	56
BS374	67	13	24	54
BS375	67	13	35	56
BS376	67	13	28	51
BS377	57	13	35	45
BS378	67	13	28	51
BS379	67	13	25	35
BS380	67	13	27	51
BS381	67	13	19	51
BS382	67	13	11	35
BS383	67	13	31	51
BS384	67	13	11	35
BS385	67	13	35	56
BS386	67	13	21	51
BS387	57	13	27	51
BS388	67	13	25	55
BS389	67	13	35	56
BS390	67	13	24	46
BS391	67	13	31	56
BS392	67	13	27	38
BS393	57	13	35	51
BS394	57	13	24	56
BS395	67	13	27	55

BS396	67	13	27	58
BS397	57	13	35	56
BS398	67	13	27	51
BS399	57	13	35	51
BS400	67	13	35	54
BS401	67	13	35	56
BS402	67	13	11	28
BS403	67	13	27	51
BS404	67	13	21	54
BS405	57	13	11	27
BS406	67	13	23	43
BS407	67	13	28	54
BS408	67	13	28	51
BS409	67	13	11	40
BS410	67	13	27	51
BS411	57	13	24	54
BS412	67	13	28	55
BS413	67	13	27	51
BS414	67	13	27	55
BS415	67	13	11	28
BS416	67	13	35	51
BS417	67	13	35	56
BS418	67	13	27	51
BS419	67	13	43	56
BS420	67	13	24	56
BS421	67	13	17	46
BS422	67	13	24	55
BS423	67	13	30	51
BS424	52	13	24	51
BS425	67	13	27	55
BS426	67	13	24	55
BS427	67	13	27	45
BS428	67	13	35	54
BS429	67	13	27	45
BS430	67	13	35	54
BS431	67	13	24	51
BS432	67	13	28	56
BS433	67	13	27	56
BS434	67	13	24	56
BS435	67	13	35	51

BS436	67	13	27	55
BS437	67	13	28	51
BS438	67	13	35	54
BS439	67	13	27	52
BS440	57	13	35	56
BS441	67	13	11	46
BS442	67	13	35	51
BS443	67	13	27	52
BS444	67	13	28	55
BS445	67	13	28	51
BS446	67	13	27	55
BS447	67	13	25	35
BS448	67	13	35	56
BS449	67	13	11	35
BS450	67	13	27	51
BS451	67	13	27	58
BS452	67	13	28	55
BS453	67	13	35	56
BS454	67	13	35	56
BS455	57	13	27	55
BS456	57	13	35	56
BS457	67	13	27	55
BS458	67	13	43	59
BS459	67	13	35	54
BS460	57	13	35	51
BS461	67	13	11	35
BS462	67	13	35	51
BS463	67	13	35	56
BS464	67	13	20	38
BS465	67	13	27	55
BS466	57	13	31	56
BS467	57	13	27	52
BS468	67	13	30	54
BS469	57	13	11	27
BS470	67	13	11	27
BS471	67	13	26	46
BS472	57	13	27	55
BS473	67	13	25	35
BS474	67	13	27	55
BS475	67	13	35	56

BS476	67	13	35	56
BS477	67	13	21	46
BS478	57	13	11	27
BS479	67	13	27	51
BS480	67	13	11	28
BS481	67	13	35	54
BS482	67	13	22	35
BS483	67	13	36	51
BS484	67	13	35	54
BS485	67	13	24	43
BS486	57	13	24	54
BS487	67	13	13	35
BS488	67	13	35	54
BS489	67	13	30	51
BS490	67	13	11	27
BS491	67	13	35	56
BS492	67	13	27	55
BS493	67	13	11	27
BS494	67	13	28	51
BS495	67	13	35	51
BS496	57	13	11	35
BS497	67	13	27	51
BS498	67	13	24	35
BS499	67	13	27	51
BS500	67	13	24	56
BS501	67	13	11	35
BS502	67	13	31	54
BS503	57	13	18	51
BS504	67	13	11	27
BS505	67	13	35	56
BS506	57	13	27	45
BS507	57	13	31	51
BS508	67	13	35	54
BS509	57	13	28	58
BS510	67	13	11	31
BS511	67	13	28	46
BS512	67	13	28	51
BS513	67	13	35	54
BS514	67	13	35	54
BS515	57	13	28	55

BS516	57	13	29	54
BS517	67	13	24	39
BS518	67	13	11	35
BS519	67	13	28	51
BS520	67	13	27	52
BS521	67	13	27	55
BS522	67	13	19	43
BS523	67	13	27	41
BS524	67	13	21	56
BS525	67	13	11	31
BS526	67	13	23	51
BS527	57	13	28	51
BS528	67	13	27	51
BS529	67	13	27	51
BS530	54	13	27	55
BS531	67	13	27	51
BS532	67	13	27	38
BS533	67	13	20	35
BS534	67	13	27	58
BS535	57	13	31	54
BS536	67	13	35	56
BS537	67	13	27	51
BS538	67	13	24	54
BS539	67	13	24	51
BS540	57	13	11	46
BS541	67	13	11	28
BS542	67	13	27	55
BS543	67	13	35	54
BS544	67	13	24	54
BS545	57	13	11	51
BS546	67	13	35	51
BS547	67	13	28	51
BS548	67	13	35	56
BS549	67	13	39	51
BS550	67	13	27	51
BS551	67	13	28	43
BS552	57	13	27	52
BS553	67	13	35	51
BS554	67	13	27	51
BS555	67	13	27	55

BS556	67	13	24	56
BS557	67	13	31	46
BS558	67	13	35	56
BS559	67	13	11	35
BS560	67	13	27	51
BS561	67	13	27	51
BS562	67	13	27	51
BS563	67	13	27	51
BS564	67	13	28	51
BS565	67	13	27	55
BS566	67	13	27	55
BS567	67	13	27	51
BS568	67	13	18	51
BS569	67	13	35	51
BS570	67	13	19	55
BS571	67	13	35	56
BS572	67	13	25	35
BS573	67	13	27	51
BS574	67	13	35	51
BS575	67	13	35	56
BS576	67	13	27	55
BS577	67	13	35	56
BS578	67	13	35	51
BS579	67	13	25	35
BS580	67	13	35	56
BS581	67	13	27	38
BS582	57	13	24	35
BS583	67	13	27	51
BS584	67	13	35	56
BS585	67	13	25	51
BS586	57	13	11	28
BS587	67	13	24	56
BS588	57	13	35	56
BS589	67	13	11	28
BS590	67	13	27	51
BS591	57	13	28	55
BS592	67	13	30	54
BS593	67	13	35	56
BS594	67	13	28	55
BS595	67	13	11	31

BS596	57	13	27	51
BS597	67	13	27	51
BS598	67	13	31	51
BS599	67	13	24	54
BS600	67	13	27	55
BS601	67	13	27	55
BS602	67	13	27	52
BS603	67	13	28	51
BS604	67	13	11	35
BS605	67	13	12	35
BS606	67	13	28	51
BS607	54	13	24	56
BS608	57	13	28	51
BS609	67	13	25	51
BS610	67	14	11	27
BS611	67	14	11	27
BS612	67	14	24	40
BS613	57	14	28	55
BS614	67	14	23	55
BS615	67	14	35	54
BS616	67	14	28	51
BS617	67	14	11	27
BS618	57	14	28	51
BS619	67	14	24	54
BS620	67	14	35	56
BS621	67	14	27	55
BS622	67	14	11	35
BS623	57	14	28	51
BS624	67	14	11	35
BS625	67	14	31	51
BS626	67	14	11	31
BS627	67	14	29	45
BS628	67	14	11	28
BS629	67	14	28	55
BS630	67	14	35	51
BS631	67	14	28	51
BS632	57	14	13	35
BS633	67	14	28	45
BS634	67	14	27	55
BS635	67	14	35	56

BS636	67	14	30	56
BS637	67	14	27	55
BS638	67	14	11	27
BS639	54	14	31	54
BS640	67	14	11	35
BS641	67	14	27	51
BS642	57	14	27	51
BS643	67	14	28	51
BS644	67	14	11	43
BS645	67	14	28	51
BS646	67	14	11	28
BS647	67	14	28	51
BS648	67	14	27	51
BS649	67	14	24	54
BS650	67	14	25	35
BS651	67	14	28	55
BS652	67	14	27	55
BS653	67	14	35	56
BS654	57	14	24	54
BS655	67	14	27	51
BS656	57	14	35	56
BS657	67	14	31	51
BS658	67	14	27	51
BS659	57	14	24	55
BS660	57	14	25	55
BS661	57	14	35	56
BS662	67	14	27	51
BS663	67	14	27	52
BS664	57	14	11	35
BS665	57	14	11	31
BS666	67	14	27	51
BS667	67	14	20	40
BS668	67	14	27	55
BS669	67	14	28	55
BS670	67	14	27	51
BS671	57	14	35	54
BS672	67	14	27	52
BS673	67	14	28	51
BS674	57	14	25	43
BS675	67	14	35	56

BS676	67	14	40	52
BS677	67	14	11	28
BS678	67	14	35	56
BS679	67	14	31	51
BS680	67	14	35	56
BS681	67	14	31	56
BS682	67	14	35	51
BS683	67	14	35	56
BS684	67	14	11	28
BS685	67	14	40	56
BS686	67	14	35	56
BS687	67	14	24	56
BS688	67	14	35	56
BS689	67	14	24	51
BS690	67	14	27	51
BS691	67	14	24	55
BS692	57	14	27	55
BS693	67	14	27	52
BS694	67	14	19	38
BS695	57	14	27	55
BS696	67	14	11	28
BS697	67	14	35	56
BS698	57	14	35	51
BS699	67	14	28	51
BS700	67	14	11	35
BS701	67	14	27	51
BS702	67	14	22	35
BS703	67	14	35	56
BS704	57	14	27	55
BS705	67	14	24	56
BS706	67	14	27	51
BS707	67	14	35	56
BS708	67	14	24	56
BS709	67	14	11	27
BS710	67	14	31	54
BS711	57	14	27	52
BS712	67	14	11	28
BS713	67	14	28	55
BS714	67	14	24	54
BS715	67	14	11	28

BS716	67	14	35	56
BS717	67	14	27	51
BS718	67	14	12	35
BS719	67	14	35	56
BS720	67	14	35	51
BS721	67	14	30	54
BS722	67	14	28	51
BS723	67	14	27	55
BS724	67	14	28	55
BS725	67	14	23	43
BS726	67	14	24	51
BS727	67	14	35	51
BS728	67	14	35	54
BS729	67	14	35	56
BS730	67	14	27	55
BS731	67	14	27	55
BS732	67	14	11	27
BS733	54	14	35	54
BS734	54	14	24	51
BS735	67	14	11	27
BS736	67	14	11	35
BS737	54	14	27	51
BS738	67	14	27	55
BS739	67	14	35	56
BS740	67	14	28	55
BS741	67	14	24	54
BS742	67	14	24	56
BS743	67	14	21	56
BS744	57	14	11	31
BS745	67	14	24	54
BS746	67	14	35	54
BS747	67	14	27	55
BS748	57	14	25	55
BS749	67	14	31	51
BS750	57	14	27	55
BS751	67	14	31	55
BS752	67	14	28	51
BS753	67	14	24	56
BS754	54	14	35	56
BS755	67	14	28	54

BS756	67	14	11	35
BS757	67	14	27	51
BS758	57	14	28	51
BS759	57	14	28	51
BS760	67	14	24	55
BS761	67	14	23	51
BS762	67	14	11	35
BS763	67	14	27	58
BS764	67	14	11	27
BS765	67	14	28	55
BS766	67	14	25	55
BS767	67	14	27	45
BS768	67	14	27	55
BS769	67	14	11	28
BS770	67	14	31	51
BS771	67	14	24	56
BS772	67	14	27	55
BS773	67	14	35	56
BS774	67	14	28	51
BS775	67	14	24	54
BS776	67	14	25	51
BS777	67	14	20	35
BS778	67	14	24	54
BS779	67	14	38	56
BS780	67	14	11	35
BS781	67	14	27	58
BS782	67	14	27	58
BS783	67	14	41	51
BS784	67	14	31	54
BS785	67	14	24	51
BS786	67	14	27	45
BS787	57	14	27	51
BS788	67	14	27	51
BS789	67	14	24	55
BS790	67	14	23	51
BS791	67	14	28	55
BS792	67	14	25	35
BS793	67	14	13	35
BS794	67	14	11	35
BS795	67	14	24	40

BS796	67	14	27	54
BS797	67	14	23	51
BS798	67	14	27	41
BS799	67	14	24	56
BS800	54	14	21	51
BS801	67	14	27	38
BS802	67	14	11	35
BS803	57	14	31	51
BS804	67	14	35	56
BS805	67	14	35	54
BS806	67	14	24	55
BS807	57	14	28	51
BS808	67	14	11	31
BS809	67	14	35	54
BS810	57	14	28	51
BS811	67	14	24	54
BS812	57	14	21	51
BS813	67	14	26	43
BS814	67	14	35	56
BS815	67	14	25	35
BS816	57	14	23	55
BS817	67	14	28	51
BS818	67	14	11	35
BS819	67	14	28	51
BS820	67	14	28	51
BS821	67	14	24	51
BS822	67	14	11	27
BS823	67	14	28	55
BS824	67	14	27	55
BS825	67	14	28	55
BS826	57	14	27	51
BS827	57	14	35	54
BS828	67	14	25	35
BS829	67	14	31	56
BS830	67	14	27	55
BS831	67	14	27	52
BS832	57	14	27	51
BS833	67	14	31	51
BS834	67	14	35	56
BS835	57	14	25	35

BS836	67	14	28	51
BS837	67	14	27	55
BS838	67	14	11	35
BS839	67	14	20	53
BS840	67	14	46	56
BS841	67	14	29	55
BS842	57	14	27	52
BS843	67	14	35	51
BS844	67	14	28	51
BS845	67	14	31	54
BS846	67	14	11	27
BS847	67	14	21	51
BS848	67	14	25	56
BS849	67	14	35	51
BS850	57	14	24	51
BS851	67	14	24	56
BS852	67	14	27	56
BS853	67	14	28	55
BS854	67	14	27	56
BS855	67	14	34	51
BS856	67	14	11	27
BS857	67	14	27	55
BS858	67	14	27	52
BS859	67	14	27	55
BS860	57	14	31	51
BS861	67	14	28	55
BS862	67	14	24	56
BS863	67	14	31	51
BS864	67	14	11	28
BS865	67	14	35	45
BS866	57	14	27	55
BS867	57	14	27	51
BS868	67	14	11	35
BS869	67	14	35	56
BS870	67	14	35	56
BS871	67	14	28	51
BS872	57	14	27	51
BS873	67	14	27	55
BS874	57	14	28	43
BS875	67	14	31	54

BS876	67	14	27	45
BS877	67	14	28	51
BS878	57	14	27	55
BS879	67	14	27	51
BS880	57	14	28	54
BS881	67	14	35	56
BS882	57	14	35	56
BS883	67	14	27	51
BS884	67	14	28	51
BS885	67	14	27	55
BS886	57	14	18	51
BS887	67	14	35	56
BS888	57	14	28	51
BS889	67	14	31	54
BS890	67	14	25	43
BS891	67	14	24	54
BS892	57	14	27	51
BS893	67	14	27	55
BS894	67	14	24	54
BS895	57	14	35	51
BS896	57	14	28	51
BS897	67	14	27	45
BS898	67	14	35	51
BS899	67	14	27	45
BS900	67	14	11	35
BS901	67	14	11	28
BS902	67	14	11	42
BS903	67	14	28	46
BS904	67	14	28	55
BS905	67	14	11	28
BS906	54	14	28	54
BS907	67	14	42	54
BS908	67	14	27	51
BS909	67	14	11	35
BS910	67	14	11	31
BS911	67	14	20	35
BS912	67	14	28	51
BS913	67	14	35	51
BS914	67	14	11	35
BS915	67	14	11	35

BS916	67	14	35	51
BS917	67	14	11	27
BS918	67	14	11	31
BS919	67	14	27	38
BS920	57	14	28	42
BS921	67	14	11	35
BS922	54	14	24	54
BS923	67	14	27	51
BS924	67	14	28	51
BS925	67	14	35	54
BS926	67	14	27	56
BS927	67	14	26	46
BS928	67	14	35	56
BS929	67	14	24	45
BS930	67	14	35	56
BS931	67	14	29	51
BS932	67	14	27	55
BS933	67	14	28	55
BS934	67	14	31	49
BS935	67	14	27	51
BS936	67	14	18	45
BS937	67	14	31	56
BS938	67	14	11	28
BS939	57	14	27	41
BS940	67	14	35	56
BS941	67	14	28	58
BS942	67	14	21	51
BS943	67	14	11	35
BS944	67	14	11	35
BS945	67	14	24	43
BS946	67	14	27	55
BS947	67	14	11	51
BS948	67	14	11	27
BS949	67	14	28	51
BS950	67	14	35	54
BS951	67	14	27	55
BS952	67	14	11	35
BS953	57	14	24	51
BS954	57	14	35	56
BS955	67	14	24	56

BS956	67	14	27	55
BS957	67	14	29	51
BS958	67	14	13	35
BS959	67	14	28	55
BS960	57	14	29	56
BS961	67	14	25	35
BS962	67	14	11	28
BS963	57	14	27	55
BS964	67	14	27	38
BS965	67	14	30	54
BS966	67	14	35	54
BS967	57	14	24	51
BS968	54	14	27	51
BS969	67	14	27	51
BS970	67	14	25	51
BS971	57	14	23	55
BS972	67	14	24	56
BS973	67	14	29	55
BS974	67	14	43	56
BS975	67	14	20	40
BS976	67	14	11	35
BS977	57	14	28	55
BS978	67	14	27	38
BS979	67	14	31	51
BS980	57	14	35	56
BS981	67	14	24	51
BS982	57	14	23	55
BS983	67	14	24	56
BS984	67	14	35	56
BS985	67	14	27	56
BS986	57	14	30	51
BS987	67	14	11	31
BS988	67	14	27	51
BS989	67	14	11	27
BS990	67	14	27	56
BS991	67	14	35	56
BS992	67	14	35	56
BS993	67	14	35	51
BS994	57	14	28	55
BS995	67	14	24	54

	BS996	67	14	35	51
	BS997	67	14	28	51
	BS998	67	14	29	51
	BS999	67	14	31	51
	BS1000	67	14	35	56

Data S8.

Information of all Himalayan species in our study. Molecular data collected by our study are highlighted by “This study”. “Strict rule” was applied in the analyses shown in the main text, in which “potential Himalayan species” were excluded. “Relax rule” refers to “potential Himalayan species” included.

Class	Family	Species	Source	Strict rule	Relax rule	Notes
Amphibian	Bufonidae	Bufo gargarizans	Genbank	Not in the Himalaya	Widespread including the Himalaya	No sequences in the Himalaya provided ever, so the Himalayan distribution is problematic
Amphibian	Bufonidae	Bufoides meghalayanus	Genbank	Endemic to the Himalaya	Endemic to the Himalaya	
Amphibian	Bufonidae	Bufotes baturae	Genbank	Endemic to the Himalaya	Endemic to the Himalaya	
Amphibian	Bufonidae	Bufotes latastii	Genbank	Endemic to the Himalaya	Endemic to the Himalaya	
Amphibian	Bufonidae	Bufotes zamdaensis	**This study**	Endemic to the	Endemic to the	

				Himalaya	Himalaya	
Amphibian	Bufonidae	Duttaphrynus himalayanus	**This study**	Endemic to the Himalaya	Endemic to the Himalaya	
Amphibian	Bufonidae	Duttaphrynus melanostictus	Genbank	Widespread including the Himalaya	Widespread including the Himalaya	Combined with other sequences outside the Himalaya
Amphibian	Bufonidae	Duttaphrynus stomaticus	Genbank	Not in the Himalaya	Widespread including the Himalaya	No sequences in the Himalaya provided ever, so the Himalayan distribution is problematic
Amphibian	Bufonidae	Duttaphrynus stuarti	**This study**	Endemic to the Himalaya	Endemic to the Himalaya	
Amphibian	Bufonidae	Ingerophrynus macrotis	Genbank	Widespread	Widespread	Combined with other

				including the Himalaya	including the Himalaya	sequences outside the Himalaya
Amphibian	Ceratobatrachidae	Liurana alpina	Genbank	Endemic to the Himalaya	Endemic to the Himalaya	
Amphibian	Ceratobatrachidae	Liurana medogensis	Genbank	Endemic to the Himalaya	Endemic to the Himalaya	
Amphibian	Ceratobatrachidae	Liurana vallecula	**This study**	Endemic to the Himalaya	Endemic to the Himalaya	
Amphibian	Ceratobatrachidae	Liurana xizangensis	Genbank	Endemic to the Himalaya	Endemic to the Himalaya	
Amphibian	Dicroglossidae	Euphlyctis cyanophlyctis	Genbank	Widespread including the Himalaya	Widespread including the Himalaya	Combined with other sequences outside the Himalaya

Amphibian	Dicroglossidae	Euphlyctis hexadactylus	Genbank	Not in the Himalaya	Widespread including the Himalaya	No sequences in the Himalaya provided ever, so the Himalayan distribution is problematic
Amphibian	Dicroglossidae	Hoplobatrachus crassus	Genbank	Widespread including the Himalaya	Widespread including the Himalaya	Combined with other sequences outside the Himalaya
Amphibian	Dicroglossidae	Hoplobatrachus tigerinus	Genbank	Widespread including the Himalaya	Widespread including the Himalaya	Combined with other sequences outside the Himalaya
Amphibian	Dicroglossidae	Ingerana borealis	Genbank	Endemic to the Himalaya	Endemic to the Himalaya	
Amphibian	Dicroglossidae	Minervarya pierrei	Genbank	Endemic to the	Endemic to the	

				Himalaya	Himalaya	
Amphibian	Dicroglossidae	Minervarya syhadrensis	Genbank	Not in the Himalaya	Widespread including the Himalaya	No sequences in the Himalaya provided ever, so the Himalayan distribution is problematic
Amphibian	Dicroglossidae	Nanorana arnoldi	Genbank	Endemic to the Himalaya	Endemic to the Himalaya	
Amphibian	Dicroglossidae	Nanorana blanfordii	**This study**	Endemic to the Himalaya	Endemic to the Himalaya	
Amphibian	Dicroglossidae	Nanorana chayuensis	**This study**	Endemic to the Himalaya	Endemic to the Himalaya	
Amphibian	Dicroglossidae	Nanorana conaensis	Genbank	Endemic to the Himalaya	Endemic to the Himalaya	

Amphibian	Dicroglossidae	Nanorana liebigii	**This study**	Endemic to the Himalaya	Endemic to the Himalaya	
Amphibian	Dicroglossidae	Nanorana medogensis	Genbank	Endemic to the Himalaya	Endemic to the Himalaya	
Amphibian	Dicroglossidae	Nanorana parkeri	Genbank	Widespread including the Himalaya	Widespread including the Himalaya	
Amphibian	Dicroglossidae	Nanorana polunini	**This study**	Endemic to the Himalaya	Endemic to the Himalaya	
Amphibian	Dicroglossidae	Nanorana rostandi	**This study**	Endemic to the Himalaya	Endemic to the Himalaya	
Amphibian	Dicroglossidae	Occidozyga lima	Genbank	Not in the Himalaya	Widespread including the	No sequences in the Himalaya provided ever, so

					Himalaya	the Himalayan distribution is problematic
Amphibian	Dicroglossidae	Sphaerotheca breviceps	Genbank	Not in the Himalaya	Widespread including the Himalaya	No sequences in the Himalaya provided ever, so the Himalayan distribution is problematic
Amphibian	Dicroglossidae	Sphaerotheca rolandae	Genbank	Widespread including the Himalaya	Widespread including the Himalaya	
Amphibian	Megophryidae	Leptobrachium bompu	**This study**	Endemic to the Himalaya	Endemic to the Himalaya	
Amphibian	Megophryidae	Leptobrachium smithi	Genbank	Not in the Himalaya	Widespread including the	No sequences in the Himalaya provided ever, so

					Himalaya	the Himalayan distribution is problematic
Amphibian	Megophryidae	Megophrys ancrae	Genbank	Endemic to the Himalaya	Endemic to the Himalaya	
Amphibian	Megophryidae	Megophrys flavipunctata	Genbank	Endemic to the Himalaya	Endemic to the Himalaya	
Amphibian	Megophryidae	Megophrys glandulosa	Genbank	Not in the Himalaya	Widespread including the Himalaya	No sequences in the Himalaya provided ever, so the Himalayan distribution is problematic
Amphibian	Megophryidae	Megophrys himalayana	Genbank	Endemic to the Himalaya	Endemic to the Himalaya	
Amphibian	Megophryidae	Megophrys major	Genbank	Widespread	Widespread	Combined with other

				including the Himalaya	including the Himalaya	sequences outside the Himalaya
Amphibian	Megophryidae	Megophrys medogensis	Genbank	Endemic to the Himalaya	Endemic to the Himalaya	
Amphibian	Megophryidae	Megophrys megacephala	Genbank	Endemic to the Himalaya	Endemic to the Himalaya	
Amphibian	Megophryidae	Megophrys oreocrypta	Genbank	Endemic to the Himalaya	Endemic to the Himalaya	
Amphibian	Megophryidae	Megophrys oropedion	Genbank	Endemic to the Himalaya	Endemic to the Himalaya	
Amphibian	Megophryidae	Megophrys pachyproctus	Genbank	Endemic to the Himalaya	Endemic to the Himalaya	

Amphibian	Megophryidae	Megophrys parva	Genbank	Not in the Himalaya	Widespread including the Himalaya	No sequences in the Himalaya provided ever, so the Himalayan distribution is problematic
Amphibian	Megophryidae	Megophrys periosa	Genbank	Endemic to the Himalaya	Endemic to the Himalaya	
Amphibian	Megophryidae	Megophrys robusta	Genbank	Endemic to the Himalaya	Endemic to the Himalaya	
Amphibian	Megophryidae	Megophrys serchhipii	Genbank	Endemic to the Himalaya	Endemic to the Himalaya	
Amphibian	Megophryidae	Megophrys vegrandis	Genbank	Endemic to the Himalaya	Endemic to the Himalaya	
Amphibian	Megophryidae	Megophrys wuliangshanensis	Genbank	Not in the	Widespread	No sequences in the

				Himalaya	including the Himalaya	Himalaya provided ever, so the Himalayan distribution is problematic
Amphibian	Megophryidae	Megophrys zhangi	Genbank	Endemic to the Himalaya	Endemic to the Himalaya	
Amphibian	Megophryidae	Megophrys zunhebotoensis	Genbank	Endemic to the Himalaya	Endemic to the Himalaya	
Amphibian	Megophryidae	Scutiger boulengeri	Genbank	Widespread including the Himalaya	Widespread including the Himalaya	
Amphibian	Megophryidae	Scutiger mammatus	Genbank	Not in the Himalaya	Widespread including the Himalaya	No sequences in the Himalaya provided ever, so the Himalayan distribution

						is problematic
Amphibian	Megophryidae	Scutiger nepalensis	Genbank	Endemic to the Himalaya	Endemic to the Himalaya	
Amphibian	Megophryidae	Scutiger nyingchiensis	**This study**	Endemic to the Himalaya	Endemic to the Himalaya	
Amphibian	Megophryidae	Scutiger occidentalis	Genbank	Endemic to the Himalaya	Endemic to the Himalaya	
Amphibian	Megophryidae	Scutiger sikkimensis	Genbank	Endemic to the Himalaya	Endemic to the Himalaya	
Amphibian	Megophryidae	Scutiger spinosus	Genbank	Endemic to the Himalaya	Endemic to the Himalaya	
Amphibian	Megophryidae	Scutiger wuguanfui	**This study**	Endemic to the	Endemic to the	

				Himalaya	Himalaya
Amphibian	Ranidae	Amolops aniqiaoensis	**This study**	Endemic to the Himalaya	Endemic to the Himalaya
Amphibian	Ranidae	Amolops chayuensis	**This study**	Endemic to the Himalaya	Endemic to the Himalaya
Amphibian	Ranidae	Amolops medogensis	**This study**	Endemic to the Himalaya	Endemic to the Himalaya
Amphibian	Ranidae	Amolops nyingchiensis	**This study**	Endemic to the Himalaya	Endemic to the Himalaya
Amphibian	Ranidae	Amolops beibengensis	**This study**	Endemic to the Himalaya	Endemic to the Himalaya
Amphibian	Ranidae	Amolops deng	**This study**	Endemic to the	Endemic to the

				Himalaya	Himalaya	
Amphibian	Ranidae	Amolops gyirongensis	**This study**	Endemic to the Himalaya	Endemic to the Himalaya	
Amphibian	Ranidae	Amolops sp	**This study**	Endemic to the Himalaya	Endemic to the Himalaya	
Amphibian	Ranidae	Amolops wangyufani	**This study**	Endemic to the Himalaya	Endemic to the Himalaya	
Amphibian	Ranidae	Amolops yarlungzangbo	**This study**	Endemic to the Himalaya	Endemic to the Himalaya	
Amphibian	Ranidae	Amolops viridimaculatus	Genbank	Not in the Himalaya	Widespread including the Himalaya	No sequences in the Himalaya provided ever, so the Himalayan distribution is problematic

Amphibian	Ranidae	Clinotarsus alticola	Genbank	Endemic to the Himalaya	Endemic to the Himalaya	
Amphibian	Ranidae	Humerana humeralis	Genbank	Endemic to the Himalaya	Endemic to the Himalaya	
Amphibian	Ranidae	Hydrophylax leptoglossa	Genbank	Widespread including the Himalaya	Widespread including the Himalaya	Combined with other sequences outside the Himalaya
Amphibian	Ranidae	Hylarana tytleri	Genbank	Widespread including the Himalaya	Widespread including the Himalaya	
Amphibian	Ranidae	Odorrana andersonii	Genbank	Not in the Himalaya	Widespread including the Himalaya	No sequences in the Himalaya provided ever, so the Himalayan distribution

						is problematic
Amphibian	Ranidae	Odorrana chloronota	Genbank	Widespread including the Himalaya	Widespread including the Himalaya	Combined with other sequences outside the Himalaya
Amphibian	Ranidae	Odorrana livida	Genbank	Not in the Himalaya	Widespread including the Himalaya	No sequences in the Himalaya provided ever, so the Himalayan distribution is problematic
Amphibian	Ranidae	Sylvirana nigrovittata	Genbank	Widespread including the Himalaya	Widespread including the Himalaya	Combined with other sequences outside the Himalaya
Amphibian	Rhacophoridae	Chiromantis doriae	Genbank	Not in the Himalaya	Widespread including the	No sequences in the Himalaya provided ever, so

					Himalaya	the Himalayan distribution is problematic
Amphibian	Rhacophoridae	Chiromantis nongkhorensis	Genbank	Not in the Himalaya	Widespread including the Himalaya	No sequences in the Himalaya provided ever, so the Himalayan distribution is problematic
Amphibian	Rhacophoridae	Feihyla vittata	**This study**	Widespread including the Himalaya	Widespread including the Himalaya	Combined with other sequences outside the Himalaya
Amphibian	Rhacophoridae	Gracixalus medogensis	**This study**	Endemic to the Himalaya	Endemic to the Himalaya	
Amphibian	Rhacophoridae	Kurixalus appendiculatus	Genbank	Not in the Himalaya	Widespread including the	No sequences in the Himalaya provided ever, so

					Himalaya	the Himalayan distribution is problematic
Amphibian	Rhacophoridae	Kurixalus naso	**This study**	Endemic to the Himalaya	Endemic to the Himalaya	
Amphibian	Rhacophoridae	Nasutixalus jerdonii	Genbank	Endemic to the Himalaya	Endemic to the Himalaya	
Amphibian	Rhacophoridae	Nasutixalus medogensis	Genbank	Endemic to the Himalaya	Endemic to the Himalaya	
Amphibian	Rhacophoridae	Polypedates leucomystax	Genbank	Not in the Himalaya	Widespread including the Himalaya	No sequences in the Himalaya provided ever, so the Himalayan distribution is problematic
Amphibian	Rhacophoridae	Polypedates maculatus	Genbank	Not in the	Widespread	No sequences in the

				Himalaya	including the Himalaya	Himalaya provided ever, so the Himalayan distribution is problematic
Amphibian	Rhacophoridae	Polypedates megacephalus	**This study**	Widespread including the Himalaya	Widespread including the Himalaya	Combined with other sequences outside the Himalaya
Amphibian	Rhacophoridae	Polypedates teraiensis	Genbank	Endemic to the Himalaya	Endemic to the Himalaya	
Amphibian	Rhacophoridae	Raorchestes parvulus	**This study**	Not in the Himalaya	Widespread including the Himalaya	No sequences in the Himalaya provided ever, so the Himalayan distribution is problematic
Amphibian	Rhacophoridae	Rhacophorus bipunctatus	**This study**	Widespread	Widespread	

				including the Himalaya	including the Himalaya	
Amphibian	Rhacophoridae	Rhacophorus burmanus	**This study**	Widespread including the Himalaya	Widespread including the Himalaya	
Amphibian	Rhacophoridae	Rhacophorus rhodopus	Genbank	Not in the Himalaya	Widespread including the Himalaya	No sequences in the Himalaya provided ever, so the Himalayan distribution is problematic
Amphibian	Rhacophoridae	Rhacophorus smaragdinus	Genbank	Endemic to the Himalaya	Endemic to the Himalaya	
Amphibian	Rhacophoridae	Rhacophorus translineatus	**This study**	Endemic to the Himalaya	Endemic to the Himalaya	

Amphibian	Rhacophoridae	Rhacophorus tuberculatus	**This study**	Endemic to the Himalaya	Endemic to the Himalaya	
Amphibian	Rhacophoridae	Theloderma baibungense	**This study**	Endemic to the Himalaya	Endemic to the Himalaya	
Amphibian	Rhacophoridae	Theloderma moloch	**This study**	Endemic to the Himalaya	Endemic to the Himalaya	
Amphibian	Rhacophoridae	Theloderma pyaukkya	Genbank	Endemic to the Himalaya	Endemic to the Himalaya	
Reptile	Agamidae	Calotes emma	Genbank	Not in the Himalaya	Widespread including the Himalaya	
Reptile	Agamidae	Calotes jerdoni	Genbank	Not in the Himalaya	Widespread including the	No sequences in the Himalaya provided ever, so

					Himalaya	the Himalayan distribution is problematic
Reptile	Agamidae	Calotes medogensis	**This study**	Endemic to the Himalaya	Endemic to the Himalaya	
Reptile	Agamidae	Calotes minor	Genbank	Not in the Himalaya	Widespread including the Himalaya	No sequences in the Himalaya provided ever, so the Himalayan distribution is problematic
Reptile	Agamidae	Calotes mystaceus	Genbank	Not in the Himalaya	Widespread including the Himalaya	
Reptile	Agamidae	Calotes versicolor	Genbank	Not in the Himalaya	Widespread including the	No sequences in the Himalaya provided ever, so

					Himalaya	the Himalayan distribution is problematic
Reptile	Agamidae	Cristidorsa otai	Genbank	Endemic to the Himalaya	Endemic to the Himalaya	
Reptile	Agamidae	Cristidorsa planidorsata	Genbank	Endemic to the Himalaya	Endemic to the Himalaya	
Reptile	Agamidae	Draco maculatus	Genbank	Widespread including the Himalaya	Widespread including the Himalaya	Combined with other sequences outside the Himalaya
Reptile	Agamidae	Japalura andersoniana	Genbank	Endemic to the Himalaya	Endemic to the Himalaya	
Reptile	Agamidae	Japalura kumaonensis	Genbank	Endemic to the Himalaya	Endemic to the Himalaya	

Reptile	Agamidae	Japalura tricarinata	Genbank	Endemic to the Himalaya	Endemic to the Himalaya	
Reptile	Agamidae	Japalura variegata	Genbank	Endemic to the Himalaya	Endemic to the Himalaya	
Reptile	Agamidae	Laudakia melanura	Genbank	Not in the Himalaya	Widespread including the Himalaya	No sequences in the Himalaya provided ever, so the Himalayan distribution is problematic
Reptile	Agamidae	Laudakia papenfussi	Genbank	Endemic to the Himalaya	Endemic to the Himalaya	
Reptile	Agamidae	Laudakia tuberculata	Genbank	Endemic to the Himalaya	Endemic to the Himalaya	
Reptile	Agamidae	Laudakia wui	Genbank	Endemic to the	Endemic to the	

				Himalaya	Himalaya	
Reptile	Agamidae	Paralaudakia himalayana	Genbank	Not in the Himalaya	Widespread including the Himalaya	No sequences in the Himalaya provided ever, so the Himalayan distribution is problematic
Reptile	Agamidae	Phrynocephalus reticulatus	Genbank	Not in the Himalaya	Widespread including the Himalaya	No sequences in the Himalaya provided ever, so the Himalayan distribution is problematic
Reptile	Agamidae	Phrynocephalus theobaldi	Genbank	Endemic to the Himalaya	Endemic to the Himalaya	
Reptile	Agamidae	Pseudocalotes austeniana	**This study**	Endemic to the Himalaya	Endemic to the Himalaya	

Reptile	Agamidae	Ptyctolaemus gularis	**This study**	Endemic to the Himalaya	Endemic to the Himalaya	
Reptile	Agamidae	Sitana ponticeriana	Genbank	Not in the Himalaya	Widespread including the Himalaya	No sequences in the Himalaya provided ever, so the Himalayan distribution is problematic
Reptile	Agamidae	Sitana sivalensis	Genbank	Endemic to the Himalaya	Endemic to the Himalaya	
Reptile	Anguidae	Dopasia gracilis	**This study**	Widespread including the Himalaya	Widespread including the Himalaya	Combined with other sequences outside the Himalaya
Reptile	Colubridae	Ahaetulla nasuta	Genbank	Not in the Himalaya	Widespread including the	No sequences in the Himalaya provided ever, so

					Himalaya	the Himalayan distribution is problematic
Reptile	Colubridae	Ahaetulla prasina	Genbank	Not in the Himalaya	Widespread including the Himalaya	No sequences in the Himalaya provided ever, so the Himalayan distribution is problematic
Reptile	Colubridae	Amphiesma stolatum	Genbank	Not in the Himalaya	Widespread including the Himalaya	No sequences in the Himalaya provided ever, so the Himalayan distribution is problematic
Reptile	Colubridae	Archelaphe bella	Genbank	Not in the Himalaya	Widespread including the Himalaya	No sequences in the Himalaya provided ever, so the Himalayan distribution

						is problematic
Reptile	Colubridae	Boiga forsteni	Genbank	Not in the Himalaya	Widespread including the Himalaya	No sequences in the Himalaya provided ever, so the Himalayan distribution is problematic
Reptile	Colubridae	Boiga trigonata	Genbank	Not in the Himalaya	Widespread including the Himalaya	No sequences in the Himalaya provided ever, so the Himalayan distribution is problematic
Reptile	Colubridae	Calamaria pavimentata	Genbank	Not in the Himalaya	Widespread including the Himalaya	No sequences in the Himalaya provided ever, so the Himalayan distribution is problematic

Reptile	Colubridae	Coelognathus helena	Genbank	Not in the Himalaya	Widespread including the Himalaya	No sequences in the Himalaya provided ever, so the Himalayan distribution is problematic
Reptile	Colubridae	Coelognathus radiatus	Genbank	Not in the Himalaya	Widespread including the Himalaya	No sequences in the Himalaya provided ever, so the Himalayan distribution is problematic
Reptile	Colubridae	Dendrelaphis cyanochloris	**This study**	Widespread including the Himalaya	Widespread including the Himalaya	
Reptile	Colubridae	Dendrelaphis pictus	Genbank	Not in the Himalaya	Widespread including the	No sequences in the Himalaya provided ever, so

					Himalaya	the Himalayan distribution is problematic
Reptile	Colubridae	Elaphe cantoris	**This study**	Endemic to the Himalaya	Endemic to the Himalaya	
Reptile	Colubridae	Elaphe hodgsoni	Genbank	Endemic to the Himalaya	Endemic to the Himalaya	
Reptile	Colubridae	Elaphe taeniura	Genbank	Widespread including the Himalaya	Widespread including the Himalaya	Combined with other sequences outside the Himalaya
Reptile	Colubridae	Fowlea flavipunctatus	Genbank	Not in the Himalaya	Widespread including the Himalaya	No sequences in the Himalaya provided ever, so the Himalayan distribution is problematic

Reptile	Colubridae	Fowlea piscator	Genbank	Widespread including the Himalaya	Widespread including the Himalaya	Combined with other sequences outside the Himalaya
Reptile	Colubridae	Fowlea schnurrenbergeri	Genbank	Widespread including the Himalaya	Widespread including the Himalaya	
Reptile	Colubridae	Gonyosoma frenatum	Genbank	Not in the Himalaya	Widespread including the Himalaya	No sequences in the Himalaya provided ever, so the Himalayan distribution is problematic
Reptile	Colubridae	Gonyosoma prasinum	Genbank	Not in the Himalaya	Widespread including the Himalaya	No sequences in the Himalaya provided ever, so the Himalayan distribution

						is problematic
Reptile	Colubridae	Hebius parallelum	**This study**	Endemic to the Himalaya	Endemic to the Himalaya	
Reptile	Colubridae	Herpetoreas burbrinki	Genbank	Endemic to the Himalaya	Endemic to the Himalaya	
Reptile	Colubridae	Herpetoreas platyceps	**This study**	Endemic to the Himalaya	Endemic to the Himalaya	
Reptile	Colubridae	Lycodon aulicus	Genbank	Not in the Himalaya	Widespread including the Himalaya	No sequences in the Himalaya provided ever, so the Himalayan distribution is problematic
Reptile	Colubridae	Lycodon fasciatus	**This study**	Widespread including the	Widespread including the	

				Himalaya	Himalaya	
Reptile	Colubridae	Lycodon jara	Genbank	Widespread including the Himalaya	Widespread including the Himalaya	
Reptile	Colubridae	Lycodon septentrionalis	**This study**	Widespread including the Himalaya	Widespread including the Himalaya	
Reptile	Colubridae	Lycodon zawi	Genbank	Endemic to the Himalaya	Endemic to the Himalaya	
Reptile	Colubridae	Oligodon cinereus	Genbank	Not in the Himalaya	Widespread including the Himalaya	No sequences in the Himalaya provided ever, so the Himalayan distribution is problematic

Reptile	Colubridae	Oligodon cyclurus	Genbank	Not in the Himalaya	Widespread including the Himalaya	No sequences in the Himalaya provided ever, so the Himalayan distribution is problematic
Reptile	Colubridae	Oligodon lipipengi	**This study**	Endemic to the Himalaya	Endemic to the Himalaya	
Reptile	Colubridae	Oligodon theobaldi	Genbank	Not in the Himalaya	Widespread including the Himalaya	No sequences in the Himalaya provided ever, so the Himalayan distribution is problematic
Reptile	Colubridae	Oligodon torquatus	Genbank	Endemic to the Himalaya	Endemic to the Himalaya	
Reptile	Colubridae	Oreocryptophis porphyraceus	Genbank	Widespread	Widespread	Combined with other

				including the Himalaya	including the Himalaya	sequences outside the Himalaya
Reptile	Colubridae	Pseudoxenodon macrops	Genbank	Widespread including the Himalaya	Widespread including the Himalaya	Combined with other sequences outside the Himalaya
Reptile	Colubridae	Ptyas korros	Genbank	Not in the Himalaya	Widespread including the Himalaya	
Reptile	Colubridae	Ptyas mucosa	Genbank	Widespread including the Himalaya	Widespread including the Himalaya	Combined with other sequences outside the Himalaya
Reptile	Colubridae	Ptyas nigromarginata	**This study**	Widespread including the	Widespread including the	

				Himalaya	Himalaya	
Reptile	Colubridae	Rhabdophis himalayanus	Genbank	Endemic to the Himalaya	Endemic to the Himalaya	
Reptile	Colubridae	Rhabdophis leonardi	**This study**	Widespread including the Himalaya	Widespread including the Himalaya	
Reptile	Colubridae	Rhabdophis nuchalis	Genbank	Not in the Himalaya	Widespread including the Himalaya	No sequences in the Himalaya provided ever, so the Himalayan distribution is problematic
Reptile	Colubridae	Rhabdophis subminiatus	Genbank	Widespread including the Himalaya	Widespread including the Himalaya	Combined with other sequences outside the Himalaya

Reptile	Colubridae	Sibynophis collaris	Genbank	Widespread including the Himalaya	Widespread including the Himalaya	Combined with other sequences outside the Himalaya
Reptile	Colubridae	Trachischium guentheri	**This study**	Endemic to the Himalaya	Endemic to the Himalaya	
Reptile	Colubridae	Trachischium monticola	**This study**	Endemic to the Himalaya	Endemic to the Himalaya	
Reptile	Colubridae	Trachischium tenuiceps	**This study**	Endemic to the Himalaya	Endemic to the Himalaya	
Reptile	Elapidae	Bungarus bungaroides	Genbank	Widespread including the Himalaya	Widespread including the Himalaya	
Reptile	Elapidae	Bungarus caeruleus	Genbank	Widespread	Widespread	

				including the Himalaya	including the Himalaya	
Reptile	Elapidae	Bungarus fasciatus	Genbank	Not in the Himalaya	Widespread including the Himalaya	
Reptile	Elapidae	Bungarus niger	Genbank	Endemic to the Himalaya	Endemic to the Himalaya	
Reptile	Elapidae	Naja kaouthia	**This study**	Not in the Himalaya	Widespread including the Himalaya	No sequences in the Himalaya provided ever, so the Himalayan distribution is problematic
Reptile	Elapidae	Naja naja	Genbank	Widespread including the	Widespread including the	Combined with other sequences outside the

				Himalaya	Himalaya	Himalaya
Reptile	Elapidae	Ophiophagus hannah	Genbank	Widespread including the Himalaya	Widespread including the Himalaya	
Reptile	Elapidae	Sinomicrurus macclellandi	Genbank	Not in the Himalaya	Widespread including the Himalaya	No sequences in the Himalaya provided ever, so the Himalayan distribution is problematic
Reptile	Gekkonidae	Altiphylax levitoni	Genbank	Endemic to the Himalaya	Endemic to the Himalaya	
Reptile	Gekkonidae	Altiphylax medogensis	**This study**	Endemic to the Himalaya	Endemic to the Himalaya	
Reptile	Gekkonidae	Altiphylax stolickzai	Genbank	Widespread	Widespread	

				including the Himalaya	including the Himalaya	
Reptile	Gekkonidae	Cyrtodactylus ayeyarwadyensis	Genbank	Widespread including the Himalaya	Widespread including the Himalaya	Combined with other sequences outside the Himalaya
Reptile	Gekkonidae	Cyrtodactylus battalensis	Genbank	Endemic to the Himalaya	Endemic to the Himalaya	
Reptile	Gekkonidae	Cyrtodactylus cayuensis	**This study**	Endemic to the Himalaya	Endemic to the Himalaya	
Reptile	Gekkonidae	Cyrtodactylus chamba	Genbank	Endemic to the Himalaya	Endemic to the Himalaya	
Reptile	Gekkonidae	Cyrtodactylus fasciolatus	Genbank	Endemic to the Himalaya	Endemic to the Himalaya	

Reptile	Gekkonidae	Cyrtodactylus gubernatoris	Genbank	Endemic to the Himalaya	Endemic to the Himalaya
Reptile	Gekkonidae	Cyrtodactylus guwahatiensis	Genbank	Endemic to the Himalaya	Endemic to the Himalaya
Reptile	Gekkonidae	Cyrtodactylus himalayanus	Genbank	Endemic to the Himalaya	Endemic to the Himalaya
Reptile	Gekkonidae	Cyrtodactylus jaintiaensis	Genbank	Endemic to the Himalaya	Endemic to the Himalaya
Reptile	Gekkonidae	Cyrtodactylus kazirangaensis	Genbank	Endemic to the Himalaya	Endemic to the Himalaya
Reptile	Gekkonidae	Cyrtodactylus khasiensis	Genbank	Endemic to the Himalaya	Endemic to the Himalaya

Reptile	Gekkonidae	Cyrtodactylus lawderanus	Genbank	Endemic to the Himalaya	Endemic to the Himalaya	
Reptile	Gekkonidae	Cyrtodactylus montanus	Genbank	Endemic to the Himalaya	Endemic to the Himalaya	
Reptile	Gekkonidae	Cyrtodactylus nagalandensis	Genbank	Endemic to the Himalaya	Endemic to the Himalaya	
Reptile	Gekkonidae	Cyrtodactylus russelli	Genbank	Endemic to the Himalaya	Endemic to the Himalaya	
Reptile	Gekkonidae	Cyrtodactylus septentrionalis	Genbank	Endemic to the Himalaya	Endemic to the Himalaya	
Reptile	Gekkonidae	Cyrtodactylus sp CHNG	Genbank	Endemic to the Himalaya	Endemic to the Himalaya	Insights into Himalayan biogeography from geckos: A molecular

						phylogeny of Cyrtodactylus (Squamata: Gekkonidae)
Reptile	Gekkonidae	Cyrtodactylus sp Glow	Genbank	Endemic to the Himalaya	Endemic to the Himalaya	Insights into Himalayan biogeography from geckos: A molecular phylogeny of Cyrtodactylus (Squamata: Gekkonidae)
Reptile	Gekkonidae	Cyrtodactylus sp Khellong	Genbank	Endemic to the Himalaya	Endemic to the Himalaya	Insights into Himalayan biogeography from geckos: A molecular phylogeny of Cyrtodactylus (Squamata: Gekkonidae)
Reptile	Gekkonidae	Cyrtodactylus sp Mizoram	Genbank	Endemic to the	Endemic to the	Insights into Himalayan

				Himalaya	Himalaya	biogeography from geckos: A molecular phylogeny of Cyrtodactylus (Squamata: Gekkonidae)
Reptile	Gekkonidae	Cyrtodactylus sp Sikkim	Genbank	Endemic to the Himalaya	Endemic to the Himalaya	Insights into Himalayan biogeography from geckos: A molecular phylogeny of Cyrtodactylus (Squamata: Gekkonidae)
Reptile	Gekkonidae	Cyrtodactylus tibetanus	**This study**	Endemic to the Himalaya	Endemic to the Himalaya	
Reptile	Gekkonidae	Cyrtodactylus tripuraensis	Genbank	Endemic to the Himalaya	Endemic to the Himalaya	

Reptile	Gekkonidae	Cyrtodactylus zhaoermii	**This study**	Endemic to the Himalaya	Endemic to the Himalaya	
Reptile	Gekkonidae	Cyrtopodion aravallensis	Genbank	Endemic to the Himalaya	Endemic to the Himalaya	
Reptile	Gekkonidae	Cyrtopodion mansarulus	Genbank	Endemic to the Himalaya	Endemic to the Himalaya	
Reptile	Gekkonidae	Cyrtopodion scabrum	Genbank	Not in the Himalaya	Widespread including the Himalaya	No sequences in the Himalaya provided ever, so the Himalayan distribution is problematic
Reptile	Gekkonidae	Gekko gecko	Genbank	Not in the Himalaya	Widespread including the Himalaya	No sequences in the Himalaya provided ever, so the Himalayan distribution

						is problematic
Reptile	Gekkonidae	Hemidactylus aquilonius	Genbank	Not in the Himalaya	Widespread including the Himalaya	No sequences in the Himalaya provided ever, so the Himalayan distribution is problematic
Reptile	Gekkonidae	Hemidactylus bowringii	Genbank	Not in the Himalaya	Widespread including the Himalaya	No sequences in the Himalaya provided ever, so the Himalayan distribution is problematic
Reptile	Gekkonidae	Hemidactylus brookii	Genbank	Not in the Himalaya	Widespread including the Himalaya	No sequences in the Himalaya provided ever, so the Himalayan distribution is problematic

Reptile	Gekkonidae	Hemidactylus flaviviridis	Genbank	Widespread including the Himalaya	Widespread including the Himalaya	
Reptile	Gekkonidae	Hemidactylus frenatus	Genbank	Widespread including the Himalaya	Widespread including the Himalaya	Combined with other sequences outside the Himalaya
Reptile	Gekkonidae	Hemidactylus garnotii	Genbank	Not in the Himalaya	Widespread including the Himalaya	No sequences in the Himalaya provided ever, so the Himalayan distribution is problematic
Reptile	Gekkonidae	Hemidactylus imbricatus	Genbank	Widespread including the Himalaya	Widespread including the Himalaya	

Reptile	Gekkonidae	Hemidactylus platyurus	**This study**	Not in the Himalaya	Widespread including the Himalaya	No sequences in the Himalaya provided ever, so the Himalayan distribution is problematic
Reptile	Gekkonidae	Hemiphyllodactylus zayuensis	**This study**	Endemic to the Himalaya	Endemic to the Himalaya	
Reptile	Gekkonidae	Hemiphyllodactylus yunnanensis	Genbank	Not in the Himalaya	Widespread including the Himalaya	No sequences in the Himalaya provided ever, so the Himalayan distribution is problematic
Reptile	Pareidae	Pareas monticola	**This study**	Widespread including the Himalaya	Widespread including the Himalaya	

Reptile	Scincidae	Ablepharus pannonicus	Genbank	Not in the Himalaya	Widespread including the Himalaya	No sequences in the Himalaya provided ever, so the Himalayan distribution is problematic
Reptile	Scincidae	Asymblepharus nyingchiensis	**This study**	Endemic to the Himalaya	Endemic to the Himalaya	
Reptile	Scincidae	Asymblepharus sp	**This study**	Endemic to the Himalaya	Endemic to the Himalaya	
Reptile	Scincidae	Asymblepharus medogensis	**This study**	Endemic to the Himalaya	Endemic to the Himalaya	
Reptile	Scincidae	Eumeces schneiderii	Genbank	Not in the Himalaya	Widespread including the Himalaya	No sequences in the Himalaya provided ever, so the Himalayan distribution

						is problematic
Reptile	Scincidae	Eurylepis taeniolata	Genbank	Not in the Himalaya	Widespread including the Himalaya	No sequences in the Himalaya provided ever, so the Himalayan distribution is problematic
Reptile	Scincidae	Eutropis carinata	Genbank	Widespread including the Himalaya	Widespread including the Himalaya	Combined with other sequences outside the Himalaya
Reptile	Scincidae	Eutropis dissimilis	Genbank	Widespread including the Himalaya	Widespread including the Himalaya	
Reptile	Scincidae	Eutropis macularia	Genbank	Widespread including the	Widespread including the	Combined with other sequences outside the

				Himalaya	Himalaya	Himalaya
Reptile	Scincidae	Eutropis multifasciata	Genbank	Widespread including the Himalaya	Widespread including the Himalaya	Combined with other sequences outside the Himalaya
Reptile	Scincidae	Lygosoma albopunctatum	Genbank	Widespread including the Himalaya	Widespread including the Himalaya	Combined with other sequences outside the Himalaya
Reptile	Scincidae	Lygosoma lineolatum	Genbank	Not in the Himalaya	Widespread including the Himalaya	No sequences in the Himalaya provided ever, so the Himalayan distribution is problematic
Reptile	Scincidae	Lygosoma punctata	Genbank	Not in the Himalaya	Widespread including the	

					Himalaya	
Reptile	Scincidae	Scincella doriae	**This study**	Widespread including the Himalaya	Widespread including the Himalaya	
Reptile	Scincidae	Scincella reevesii	Genbank	Not in the Himalaya	Widespread including the Himalaya	No sequences in the Himalaya provided ever, so the Himalayan distribution is problematic
Reptile	Scincidae	Sphenomorphus indicus	Genbank	Widespread including the Himalaya	Widespread including the Himalaya	Combined with other sequences outside the Himalaya
Reptile	Scincidae	Sphenomorphus maculatus	**This study**	Widespread including the	Widespread including the	

				Himalaya	Himalaya	
Reptile	Viperidae	Azemiops feae	Genbank	Not in the Himalaya	Widespread including the Himalaya	No sequences in the Himalaya provided ever, so the Himalayan distribution is problematic
Reptile	Viperidae	Echis carinatus	Genbank	Not in the Himalaya	Widespread including the Himalaya	No sequences in the Himalaya provided ever, so the Himalayan distribution is problematic
Reptile	Viperidae	Ovophis makazayazaya	Genbank	Not in the Himalaya	Widespread including the Himalaya	No sequences in the Himalaya provided ever, so the Himalayan distribution is problematic

Reptile	Viperidae	Ovophis monticola	**This study**	Widespread including the Himalaya	Widespread including the Himalaya
Reptile	Viperidae	Ovophis zayuensis	**This study**	Endemic to the Himalaya	Endemic to the Himalaya
Reptile	Viperidae	Protobothrops himalayanus	**This study**	Endemic to the Himalaya	Endemic to the Himalaya
Reptile	Viperidae	Protobothrops jerdonii	**This study**	Widespread including the Himalaya	Widespread including the Himalaya
Reptile	Viperidae	Protobothrops kaulbacki	Genbank	Widespread including the Himalaya	Widespread including the Himalaya

Reptile	Viperidae	Protobothrops mucrosquamatus	Genbank	Not in the Himalaya	Widespread including the Himalaya	No sequences in the Himalaya provided ever, so the Himalayan distribution is problematic
Reptile	Viperidae	Trimeresurus albolabris	Genbank	Widespread including the Himalaya	Widespread including the Himalaya	Combined with other sequences outside the Himalaya
Reptile	Viperidae	Trimeresurus erythrurus	Genbank	Not in the Himalaya	Widespread including the Himalaya	No sequences in the Himalaya provided ever, so the Himalayan distribution is problematic
Reptile	Viperidae	Trimeresurus medoensis	**This study**	Endemic to the Himalaya	Endemic to the Himalaya	

Reptile	Viperidae	Trimeresurus popeiorum	Genbank	Widespread including the Himalaya	Widespread including the Himalaya	Combined with other sequences outside the Himalaya
Reptile	Viperidae	Trimeresurus purpureomaculatus	Genbank	Not in the Himalaya	Widespread including the Himalaya	No sequences in the Himalaya provided ever, so the Himalayan distribution is problematic
Reptile	Viperidae	Trimeresurus septentrionalis	Genbank	Endemic to the Himalaya	Endemic to the Himalaya	
Reptile	Viperidae	Trimeresurus stejnegeri	Genbank	Not in the Himalaya	Widespread including the Himalaya	No sequences in the Himalaya provided ever, so the Himalayan distribution is problematic

Reptile	Viperidae	Trimeresurus tibetanus	Genbank	Endemic to the Himalaya	Endemic to the Himalaya	
Reptile	Viperidae	Trimeresurus yunnanensis	Genbank	Not in the Himalaya	Widespread including the Himalaya	No sequences in the Himalaya provided ever, so the Himalayan distribution is problematic

References

1. Xing Y and Ree RH. Uplift-driven diversification in the Hengduan Mountains, a temperate biodiversity hotspot. *Proceedings of the National Academy of Sciences* 2017; **114**: E3444–E34.
2. Sambrook J, Fritsch EF and Maniatis T. *Molecular cloning: a laboratory manual*. New York: Cold spring harbor laboratory press, 1989.
3. Kumar S, Stecher G and Li M *et al*. MEGA X: molecular evolutionary genetics analysis across computing platforms. *Molecular Biology and Evolution* 2018; **35**: 1547–49.
4. Vaidya G, Lohman DJ and Meier R. SequenceMatrix: concatenation software for the fast assembly of multi-gene datasets with character set and codon information. *Cladistics* 2011; **27**: 171–80.
5. Nguyen LT, Schmidt HA and von Haeseler A *et al*. IQ-TREE: a fast and effective stochastic algorithm for estimating maximum-likelihood phylogenies. *Molecular Biology and Evolution* 2014; **32**: 268–74.
6. Trifinopoulos J, Nguyen LT and von Haeseler A *et al*. W-IQ-TREE: a fast online phylogenetic tool for maximum likelihood analysis. *Nucleic Acids Research* 2016; **44**: W232–W5.
7. Kalyaanamoorthy S, Minh BQ and Wong TKF *et al*. ModelFinder: fast model selection for accurate phylogenetic estimates. *Nature Methods* 2017; **14**: 587.
8. Minh BQ, Nguyen MAT and von Haeseler A. Ultrafast approximation for phylogenetic bootstrap. *Molecular Biology and Evolution* 2013; **30**: 1188–95.
9. Hoang DT, Chernomor O and von Haeseler A *et al*. UFBoot2: improving the ultrafast bootstrap approximation. *Molecular Biology and Evolution* 2017; **35**: 518–22.
10. Anisimova M and Gascuel O. Approximate likelihood-ratio test for branches: a fast, accurate, and powerful alternative. *Systematic Biology* 2006; **55**: 539–52.
11. Guindon S, Dufayard JF and Lefort V *et al*. New algorithms and methods to estimate maximum-likelihood phylogenies: assessing the performance of PhyML 3.0. *Systematic Biology* 2010; **59**: 307–21.
12. Wilcox TP, Zwicki DJ and Heath TA *et al*. Phylogenetic relationships of the dwarf boas and a comparison of Bayesian and bootstrap measures of phylogenetic support. *Molecular Phylogenetics and Evolution* 2002; **25**: 361–71.

13. Yang Z. PAML 4: phylogenetic analysis by maximum likelihood. *Molecular Biology and Evolution*2007; **24**: 1586–91.
14. Feng YJ, Blackburn DC and Liang D *et al*. Phylogenomics reveals rapid, simultaneous diversification of three major clades of Gondwanan frogs at the Cretaceous–Paleogene boundary. *Proceedings of the National Academy of Sciences* 2017; **114**: E5864–E70.
15. Zheng Y and Wiens JJ. Combining phylogenomic and supermatrix approaches, and a time-calibrated phylogeny for squamate reptiles (lizards and snakes) based on 52 genes and 4162 species. *Molecular Phylogenetics and Evolution* 2016; **94**: 537–47.
16. Brown JW and Smith SA. The Past Sure is Tense: On Interpreting Phylogenetic Divergence Time Estimates. *Systematic Biology* 2018; **67**: 340–53.
17. R Core Team. *R: A language and environment for statistical computing*. https://www.R-project.org.
18. H. Wickham H. *ggplot2: Elegant Graphics for Data Analysis*. https://ggplot2.tidyverse.org.
19. Boos WR and Kuang Z. Dominant control of the South Asian monsoon by orographic insulation versus plateau heating. *Nature* 2010*;* **463**: 218–23.
20. Ding L, Spicer RA and Yang J *et al*. Quantifying the rise of the Himalaya orogen and implications for the South Asian monsoon. *Geology* 2017*;***45**: 215–8.
21. Spicer RA, Tao S and Valdes PJ *et al*. Why the 'Uplift of the Tibetan Plateau' is a myth. *National Science Review* 2020; 0: 1–19.
22. Matzke NJ. Probabilistic historical biogeography: new models for founder event speciation, imperfect detection, and fossils allow improved accuracy and model-testing. *Frontiers of Biogeography* 2013; 5: 242–8.
23. Yu Y, Harris AJ and Blair C *et al*. RASP (Reconstruct Ancestral State in Phylogenies): a tool for historical biogeography. *Molecular Phylogenetics and Evolution* 2015; **87**: 46–9.
24. Jønsson KA and Holt BG. Islands contribute disproportionately high amounts of evolutionary diversity in passerine birds. *Nature Communications* 2015; **6**: 8538.
25. Klaus S, Morley RJ and Plath M *et al*. Biotic interchange between the Indian subcontinent and mainland Asia through time. *Nature Communications* 2016; **7**: 12132.
26. Székely GJ and Rizzo ML. Hierarchical clustering via joint between-within distances. Extending Ward's minimum variance method. *Journal of Classification* 2005; **22**: 151–83.

27. Rizzo ML and Székely GJ. DISCO analysis: a nonparametric extension of analysis of variance. *Annals of Applied Probability* 2010; **4**: 1034–55.
28. James NA and Matteson DS. ecp: An R package for nonparametric multiple change point analysis of multivariate data. *Journal of Statistical Software* 2014; **62**: 1–25.
29. Suchard MA, Lemey P and Baele G *et al*. Bayesian phylogenetic and phylodynamic data integration using BEAST 1.10. *Virus Evolution* 2018; **4**: vey016.
30. Lanfear R, Frandsen PB and Wright AM *et al*. PartitionFinder 2: New Methods for Selecting Partitioned Models of Evolution for Molecular and Morphological Phylogenetic Analyses. *Molecular Biology & Evolution* 2016; **34**: 772–3.
31. Rambaut A, Drummond AJ and Xie D *et al*. Posterior summarisation in Bayesian phylogenetics using Tracer 1.7. *Systematic Biology* 2018; **5**: 5.
32. Rambaut A and Drummond AJ. *TreeAnnotator* 1.4.5. http://beastbioedacuk/TreeAnnotator.
33. Bossuyt F and Milinkovitch MC. Convergent adaptive radiations in Madagascan and Asian ranid frogs reveal covariation between larval and adult traits. *Proceedings of the National Academy of Sciences* 2000; **97**: 6585–90.
34. Stuart BL, Inger RF and Voris HK. High level of cryptic species diversity revealed by sympatric lineages of Southeast Asian forest frogs. *Biology Letters* 2006; **2**: 470–4.
35. Bossuyt F and Dubois A. A review of the frog genus *Philautus* Gistel, 1848 (Amphibia, Anura, Ranidae, Rhacophorinae). *Zeylanica* 2001; **6**: 1–112.
36. Stuart BL. The phylogenetic problem of *Huia* (Amphibia: Ranidae). *Molecular Phylogenetics and Evolution* 2008; **46**: 49–60.
37. Goebel AM, Donnelly JM and Atz ME. PCR primers and amplification methods for 12S ribosomal DNA, the control region, cytochrome oxidase I, and cytochrome b in bufonids and other frogs, and an overview of PCR primers which have amplified DNA in amphibians successfully. *Molecular Phylogenetic Evolution* 1999; **11**: 163–99.
38. Macey JR, Schulte JA and Larson A *et al*. Phylogenetic relationships of toads in the *Bufo bufo* species group from the eastern escarpment of the Tibetan Plateau: a case of vicariance and dispersal. *Molecular Phylogenetics and Evolution* 1998; **9**: 80–7.

39. Macey JR, Larson A and Ananjeva NB *et al*. Two novel gene orders and the role of light-strand replication in rearrangement of the vertebrate mitochondrial genome. *Molecular Biologyand Evolution* 1997; **14**: 91–104.
40. Che J, Chen HM and Yang JX *et al*. Universal COI primers for DNA barcoding amphibians. *Molecular Ecology Resources* 2012; **12**: 247–58.
41. Li R, Chen W and Tu L *et al*. Rivers as barriers for high elevation amphibians: a phylogeographic analysis of the alpine stream frog of the Hengduan Mountains. *Journal of Zoology* 2009; **277**: 309–16.
42. Burbrink FT, Lawson R and Slowinski JB. Mitochondrial DNA phylogeography of the polytypic North American rat snake (*Elaphe obsoleta*): a critique of the subspecies concept.*Evolution* 2000*;* **54**: 2107–18.
43. Arévalo SK and Davis JW. Sites Mitochondrial-DNA sequence divergence and phylogenetic-relationships among 8 chromosome races of the *Sceloporus grammicus* complex (Phrynosomatidae) in central Mexico. *Systematic Biology* 1994; **43**: 387–418.
44. R Lawson, Slowinski JB and Crother BI *et al*. Phylogeny of the Colubroidea (Serpentes): New evidence from mitochondrial and nuclear genes. *Molecular Phylogenetics and Evolution* 2005; **37**: 581–601.
45. Macey J and Schulte J. Molecular Phylogenetics, tRNA Evolution, and Historical Biogeography in Anguid Lizards and Related Taxonomic Families. *Molecular Phylogenetics and Evolution* 1999; **12**: 250–72.
46. Linkem CW, Diesmos AC and Brown RM. A new species of scincid lizards (Genus: *Sphenomorphus*) from Palawan Island, Philippines. *Herpetologica* 2010; **66**: 67–79.
47. Whiting AS, Bauer AM and Sites JW. Phylogenetic relationships and limb loss in sub-Saharan African scincine lizards (Squamata: Scincidae). *Molecular Phylogenetics and Evolution* 2003; **29**: 582–98.
48. Bauer AM, de Silva A and Greenbaum E *et al*. A new species of day gecko from high elevation in Sri Lanka, with a preliminary phylogeny of Sri Lankan *Cnemaspis* (Reptilia: Squamata: Gekkonidae). Mitteilungen aus dem Museum für Naturkunde in Berlin. *Zoologische Reihe* 2007; **83**: 22–32.

49. Rage JC and RočekZ. Evolution of anuran assemblages in the Tertiary and Quaternary of Europe, in the context of palaeoclimate and palaeogeography. *Amphibia-Reptilia* 2003; **24**: 133–67.
50. Böhme M. The oldest representative of a brown frog [Ranidae] from the Early Miocene of Germany. *Acta Palaeontologica Polonica* 2001; **46**: 119–24.
51. Voorhies M, Holman JA and Xue XX. The Hottell Ranch rhino quarries (basal Ogallala; medial Barstovian), Banner County, Nebraska; Part I; Geologic setting, faunal lists, lower vertebrates. *Rocky Mountain Geology* 1987; **25**: 55–69.
52. Holman JA. Early Miocene anurans from Florida. *Quarterly Journal of the Florida Academy of Sciences* 1965; **28**: 68–82.
53. Holman JA. Additional Miocene anurans from Florida. *Quarterly Journal of the Florida Academy of Sciences* 1967; **30**: 121–40.
54. Hoffstetter R. Un Serpent terrestre dans le Crétacé inférieur du Sahara. A terrestrial snake in the Lower Cretaceous of the Sahara. *Bulletin de la Société Géologique de France* 1960; **7**: 897–902.
55. Colston TJ, Grazziotin FG and Shepard DB *et al*. Molecular systematics and historical biogeography of tree boas (*Corallus* spp.). *Molecular Phylogenetics and Evolution* 2013; **66**: 953–9.
56. Wüster W, Peppin L and Pook CE *et al*. A nesting of vipers: phylogeny and historical biogeography of the Viperidae (Squamata: Serpentes). *Molecular Phylogenetics and Evolution* 2008; **49**: 445–59.
57. Báez AM and Nicoli L. Bufonid toads from the Late Oligocene beds of Salla Bolivia. *Journal of Vertebrate Paleontology* 2004; **24**: 73–9.
58. Sanchíz B. Salientia. In: Wellnhofer P (ed.). *Handbuch der Paläoherpetologie*. Verlag Dr. Friedrich Pfeil, Munich, 1998, 1–275.
59. Rocek Z and Rage JC. Tertiary Anura of Africa, Asia, Europe, North America, and Australia. In: Heatwole HF and Carroll RL (eds.). *Amphibian biology*. Chipping Norton, Australia, Surrey Beatty & Sons, 2000, 1334–89.
60. M. Böhme. The Miocene climatic optimum: Evidence from ectothermic vertebrates of Central Europe. *Palaeogeography, Palaeoclimatology, Palaeoecology* 2003, **195**: 389–401.

61. Mahony S, Foley NM and Biju SD *et al*. Evolutionary History of the Asian Horned Frogs (Megophryinae): Integrative Approaches to Timetree Dating in the Absence of a Fossil Record. *Molecular Biology and Evolution* 2017; **34**: 744–71.
62. Estes R. New fossil pelobatid frogs and a review of the genus *Eopelobates*. *Bulletin of the Museum of Comparative Zoology* 1970; **139**: 293–339.
63. Henrici AC, Baez AM and Grande L. *Aerugoamnis paulus*, new genus and new species (Anura: Anomocoela): first reported anuran from the Early Eocene (Wasatchian) Fossil Butte Member of the Green River Formation, Wyoming. *Annals of Carnegie Museum* 2013; **81**: 295–309.
64. Rocek Z, Wuttke M and Gardner JD *et al*. The Euro-American genus *Eopelobates*, and a re-definition of the family Pelobatidae (Amphibia, Anura). *Palaeobiodiversity and Palaeoenvironments* 2014; **94**: 529–67.
65. Gilmore W. Fossil lizards of North America. *Memoirs of the National Academy of Sciences* 1928; **22**: 1–201.
66. Vasilyan D, Böhme M and Klembara J. First record of fossil *Ophisaurus* (Anguimorpha, Anguidae) from Asia. *Journal of Vertebrate Paleontology* 2016; **36**: e1219739.
67. Scarpetta S. The earliest known occurrence of *Elgaria* (Squamata: Anguidae) and a minimum age for crown Gerrhonotinae: Fossils from the Split Rock Formation, Wyoming, USA. *Palaeontologia Electronica* 2018; **21**: 1–8.
68. Townsend TM, Mulcahy DG and Noonan BP *et al*. Phylogeny of iguanian lizards inferred from 29 nuclear loci, and a comparison of concatenated and species-tree approaches for an ancient, rapid radiation. *Molecular Phylogenetics and Evolution* 2011; **61**: 363–80.
69. Yatkola DA. Mid-Miocene lizards from western Nebraska. *Copeia* 1976; **1976**: 645–54.
70. Smith KT. A diverse new assemblage of late Eocene squamates (Reptilia) from the Chadron Formation of North Dakota, U.S.A. *Palaeontologia Electronica*2006; **9**: 1–44.
71. Iturralde-Vinent MA and MacPhee RDE. Age and paleogeographical origin of Dominican amber. *Science* 1996; **273**: 1850–2.
72. Estes R. The fossil record and early distribution of lizards. In: Rhodin A and Miyata K (eds.). *Advances in Herpetology and Evolutionary Biology. Essays in Honor of Ernest E. Williams*. Museum of Comparative Zoology, 1983, 365–98.

73. Schulte JA and Moreno-Roark F. Live birth among iguanian lizards predates Pliocene−Pleistocene glaciations. *Biology Letters* 2010; **6**: 216–8.
74. Hou LH. New materials of Palaeocene lizards of Anhui. *Certebrata Palasiatica* 1976; **14**: 45–52.
75. Auge M. Revision du lezard Uromastix europeaus (Reptilia, Lacertilia) de l' Oligocene français. Analyse fontionelle de l' appareil masticateur de genre Uromastix et implications paleoecologiques. *Revue de Paléobiologie* 1988; **7**: 317–25.
76. Covacevich J, Couper P and Molnar RE *et al*. Miocene dragons from Riversleigh: new data on the history of the family Agamidae (Reptilia: Squamata) in Australia. *Memoires of the Queensland Museum*1990; **29**: 339–60.
77. Conrad JL and Norell MA. A complete Late Cretaceous iguanian (Squamata, Reptilia) from the Gobi and identification of a new iguanian clade. *American Museum Novitates* 2007; **2007**: 1–47.
78. Bauer AM, Böhme W and Weitschat W. An Early Eocene gecko from Baltic amber and its implications for the evolution of gecko adhesion. *Journal of Zoology* 2005; **265**: 327–32.
79. Estes R. A scincoid lizard from the Cretaceous and Paleocene of Montana. *Breviora* 1969; **331**: 1–9.
80. Bryant LJ. *Non-dinosaurian lower vertebrates across the Cretaceous–Tertiary boundary in northeastern Montana.* Berkeley: University of California Press, 1989, 1–107.
81. Krause DW, Evans SE and Gao KQ. First definitive record of Mesozoic lizards from Madagascar. *Journal of Vertebrate Paleontology* 2003; **23**: 842–56.
82. Agarwal I, Bauer AM and Jackman TR *et al*. Insights into Himalayan biogeography from geckos: A molecular phylogeny of *Cyrtodactylus* (Squamata: Gekkonidae). *Molecular Phylogenetics and Evolution* 2014; **80**: 145–55.
83. Heinicke MP, Greenbaum E and Jackman TR *et al*. Phylogeny of a trans-Wallacean radiation (Squamata, Gekkonidae, *Gehyra*) supports a single early colonization of Australia. *Zoologica Scripta* 2011; **40**: 584–602.
84. Arnold EN and Poinar G. A 100 million year old gecko with sophisticated adhesive toe pads, preserved in amber from Myanmar. *Zootaxa* 2008; **1847**: 62–8.
